# Sterically Controlled C(sp^2^)–H Carboxylation and Formylation: A Complementary Strategy to S_E_Ar Approaches

**DOI:** 10.1021/acs.orglett.5c03474

**Published:** 2025-09-15

**Authors:** Rita de Jesus, Jyotirmoy Dey, Elisa Chakraborty, Manuel van Gemmeren

**Affiliations:** Otto Diels-Institut für Organische Chemie, Christian-Albrechts-Universität zu Kiel, Otto-Hahn-Platz 4, 24118 Kiel, Germany

## Abstract

Carboxyl- and formyl-substituted arenes are key motifs in organic chemistry, typically prepared via electrophilic aromatic substitution (S_E_Ar) reactions. S_E_Ar reactions hold tremendous synthetic value, and one of the key advantages is their predictable regioselectivity. Conversely, certain positions remain out of reach, such as sterically accessible but comparably electron-poor sites. Herein, we describe a nondirected C­(sp^2^)–H carboxylation and formylation of arenes enabled by a dual ligand-based catalyst. By optimizing the steric control of a nondirected olefination of arenes and combining this method with two distinct protocols for the subsequent oxidative cleavage of alkenes, we can generate a complementary set of regioisomeric products. The synthetic utility is demonstrated by a broad scope with suitability for late-stage functionalization.

## Graphical abstract

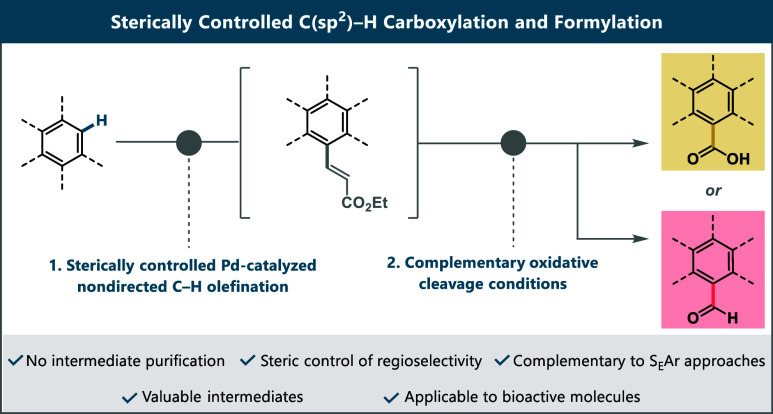

Acyl and carboxyl moieties are among the most prevalent functional groups in nature,^1^ featuring a wide range of bioactive molecules,^2,3^ fragrances, and dyes.^4^ Their alkyl/aryl derivatives are prevalent as precursors for functional materials^5–7^ and as key synthetic intermediates in natural products synthesis.^8,9^ From a retrosynthetic perspective, a common way to synthesize acyl- and carboxyl-substituted arenes is through electrophilic aromatic substitution (S_E_Ar) reactions (Scheme 1, path A).^10–13^ Typical methods to install a carboxyl group employ organolithium or Grignard reagents in combination with CO_2_.^14–19^ Formyl groups are introduced using methodologies such as the Gattermann–Koch^20^ and Vilsmeier–Haack^21^ reactions, which, upon acid-promoted activation, enable the formation of aromatic aldehydes via an S_E_Ar pathway. Recently, various milder protocols have been reported such as biocatalytic approaches^22^ and heterogeneously catalyzed methods using gaseous CO or CO_2_.^23–25^ Transition metal-catalyzed cross-coupling methodologies giving access to the same product classes have also been developed,^18,26,27^ but predominantly remain limited by the reliance on prefunctionalized starting materials.^28,29^ Additionally, transition metal-catalyzed C–H activation as an elegant tool has been used on substrates having coordinating directing groups (DGs) to enable efficient and broadly applicable C–H acylation protocols with regioselectivities controlled by the DG.^30^

**Scheme 1:**
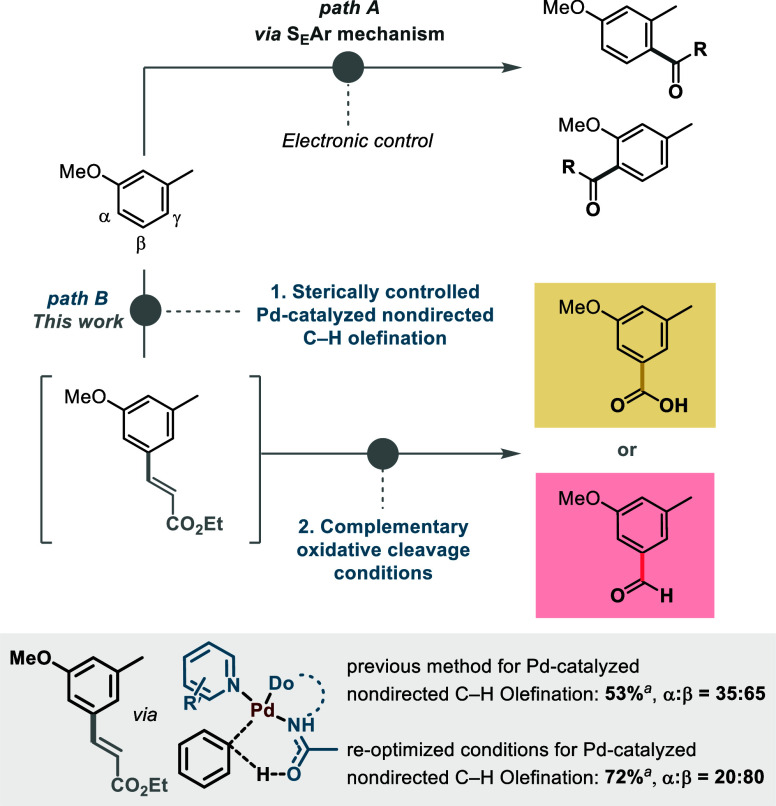
Comparison between the S_E_Ar Pathway (Path A) and our Sterically Controlled Protocol (Path B)^a^

a Yields and regioisomeric ratios given were determined by GC-FID using mesitylene as an internal standard.

Despite these advances, S_E_Ar reactions and related transformations remain among the most extensively investigated and widely utilized reactions in organic chemistry. One key advantage of these reactions is their predictable regioselectivity, which stems directly from the S_E_Ar mechanism. However, this mechanism also implies that certain positions in an arene substrate remain out of reach, such as sterically accessible but comparably electron-poor positions. Recently, arene-limited protocols for the palladium-catalyzed nondirected C–H activation/functionalization of unbiased arene C–H bonds have been developed, which have proven useful for late-stage functionalization.^31–39^ In this context, our group has introduced dual ligand-based Pd catalysts in which monodentate ligands replace the directing group donor atom, allowing steric and electronic tuning while stabilizing the catalyst. The bidentate ligands position the ligands on the metal, facilitate C–H activation via concerted metalation–deprotonation (CMD), and can be tuned (through donor properties, linker length, and bite angle) to optimize sterics and electronics. By combining both ligands, the catalyst system enables efficient nondirected C–H activation while remaining sensitive to both steric and electronic control, with steric influences typically dominating the regioselectivity.^40,41^ Due to the versatility of C=C bonds as linchpins for further transformations, we envisaged a two-step protocol. First, through reoptimization of our dual ligand-catalyzed nondirected olefination of arenes,^34,42^ enhanced steric control was achieved using a novel ligand combination (Scheme 1, path B).

Combining this step with subsequent oxidative cleavage, carboxylic acid or aldehyde moieties can be accessed.^43–46^ Herein, we report a sterically controlled C­(sp^2^)–H carboxylation and formylation, an orthogonal protocol that complements traditional S_E_Ar pathways.^47^ In order to monitor which factors govern the regioselectivity of the olefination step, we chose *m*-cresol ether (**1c**) as a model substrate since its sterically and electronically preferred positions are orthogonal to one another (Scheme 1, paths A and B). Based on our previous mechanistic work, we expected that fine-tuning the steric and electronic properties of both ligands could render the catalyst system less electrophilic. This would enable an even more synchronous C–H activation pathway, thus minimizing electronic influences and allowing steric factors to dominate the regioselectivity.^41^ After extensive optimization of the reaction conditions, we discovered that the combination of phenazine (**ML1**) as a monodentate ligand and an *N*-acylsulfonamide-based bidentate ligand (NASA-ligand, **BL1**) delivers optimal results. Notably, this catalyst system exhibits a substantially higher catalytic activity and enhanced steric control of the regioselectivity compared to those of previous dual ligand-enabled catalysts. Contrary to the S_E_Ar mechanism, which provides functionalization mostly in the γ-position (Scheme 1, path A),^48,49^ our novel dual-ligand system overrides electronic effects, giving functionalization mostly in the sterically unhindered β-position (Scheme 1, path B). Subsequently, two distinct protocols for oxidative cleavage were developed. Carboxylic acids could be obtained by using catalytic osmium tetroxide (OsO_4_) along with oxone as a terminal oxidant (Scheme 2),^44^ whereas aldehydes could be synthesized in a two-step protocol, first converting the alkene to vicinal diols followed by their oxidative cleavage using NaIO_4_ under modified Lemieux–Johnson oxidation (Scheme 3).^43^ With the optimized conditions in hand, we decided to explore the substrate scope to obtain carboxylic acids (Scheme 2) and aldehydes (Scheme 3). **2a** was obtained with *meta* as a major isomer, with suppressed *ortho*-functionalization due to the steric bulkiness of the *tert*-butyl group. Upon oxidative cleavage, **3a** was obtained in good yield. 1,3-Disubstituted arenes afforded products **2b** and **2c** in high yields with remarkable regioselectivity, similar to silyl-protected phenol **2d** that was obtained with improved yield and regioselectivity in comparison to literature reports.^33^ **3b** was obtained in 72% yield as a pure β-isomer, orthogonal to methods that favor electron-rich positions.^50^ Similarly, **3c** and **3d** were obtained as pure regioisomers. **1e** was olefinated in the most sterically accessible position of the arene (**2e**), and upon oxidative cleavage, **3e** was obtained in good yield. To probe the synthetic utility of our protocol, we conducted the olefination of **1e** and subsequent oxidative cleavage on a preparative scale (1.0 mmol scale), showing the robustness of our method.^51^ A combination of steric and electronic factors explains the regioselectivity observed in **2f** and **2g**: the inductive effect of the chlorine in addition to lower steric shielding affords functionalization only at the β-position. Consequently, **3f** and **3g** were obtained as β-products in good yields. Next, we extended our method to a variety of bioactive molecules. Protected phenylalanine could be converted to **2h** in good yields.^52^ Once cleaved oxidatively, **3h** was obtained in good yields with an increase in the *meta* product, illustrating that in some cases the most sterically accessible isomer is preferentially cleaved. Evans-type reagent and blood-glucose lowering agent Nateglinide methyl ester could be converted to **2i**–**j** as regioisomeric mixtures with *meta* as the major isomer, respectively. Compared to our previous studies,^42^ no *ortho* product was formed in the olefination of **1j**, highlighting the high steric sensitivity of our catalyst. **3i**–**j** were obtained without significant change in the regioisomeric ratios. Based on the widespread occurrence and interesting biological properties of biaryls, flurbiprofen methyl ester (**1k**) was tested. Functionalization was observed in previously inaccessible positions (**2k**),^36^ and upon oxidative cleavage, **3k** was obtained in good yield with β as the major isomer. Ketoprofen methyl ester **1l** was converted in a synthetically useful yield (**2l**) with β as the major site of functionalization favored by sterics and statistics. The oxidative cleavage proved to be challenging with this substrate, requiring a slight modification of the reaction conditions to afford **3l** in good yield. As expected based on the high steric sensitivity of our catalyst, **1m** gave **2m** as well as its carboxylic acid derivative **3m** in good yields and as the pure β-isomer, orthogonal to other methods in the literature.^53^ Palonosetron precursor **1n** and thymol ether **1o** were olefinated in the sterically preferred position, giving **2n** and **2o** in moderate to good yields. **3n** and **3o** were obtained as pure regioisomers in the sterically most accessible position, using a modified oxidative cleavage procedure in the case of **3o** (Section 4.3 in the SI). Sonidegib precursor **1p** was functionalized in good yield and mostly in the sterically least hindered position (**2p**), while Gemfibrozil methyl ester **1q** was olefinated to give **2q** in moderate yield, with functionalization mostly in the β-position. Oxidative cleavage afforded **3p** as the β-isomer in good yield, while **3q** was obtained as the pure β-isomer using a modified oxidative cleavage procedure (Section 4.3 in the SI). It is important to note that, contrary to directed methods, nondirected methods usually afford mixtures of regioisomers. However, this can be harnessed as an advantage, for example, in medicinal chemistry research, since the obtained mixtures usually contain major regioisomers that would be challenging to access, typically requiring lengthy synthetic routes.^54,55^ We then turned our attention to the C­(sp^2^)-formylation of arenes (Scheme 3). Product **4a** was obtained in good yields with an improved *meta* ratio as a result of sterics. Functionalization mostly in the least sterically shielded position afforded **4b**–**g** in good yields, showing the robustness of our catalyst. Late-stage formylation delivered molecules **4h**–**j** as regioisomeric mixtures, yet to the best of our knowledge, there is no other method in the literature to access these products. **4k** and **4l** were predominantly functionalized in the β-position similarly to their carboxylic acid counterparts (**3k** and **3l**). **4m**–**o** were obtained as single isomers providing access to a new chemical space. **4p** was formylated similarly to its carboxylic acid counterpart (**3p**) in the sterically unhindered position of the trisubstituted arene. **4q** was obtained in high yields with selectivity similar to that of **3q**. Based on the obtained results, we can see that the high steric sensitivity of our catalyst typically overrides electronic effects, rendering the protocol complementary to approaches via an S_E_Ar mechanism.

**Scheme 2:**
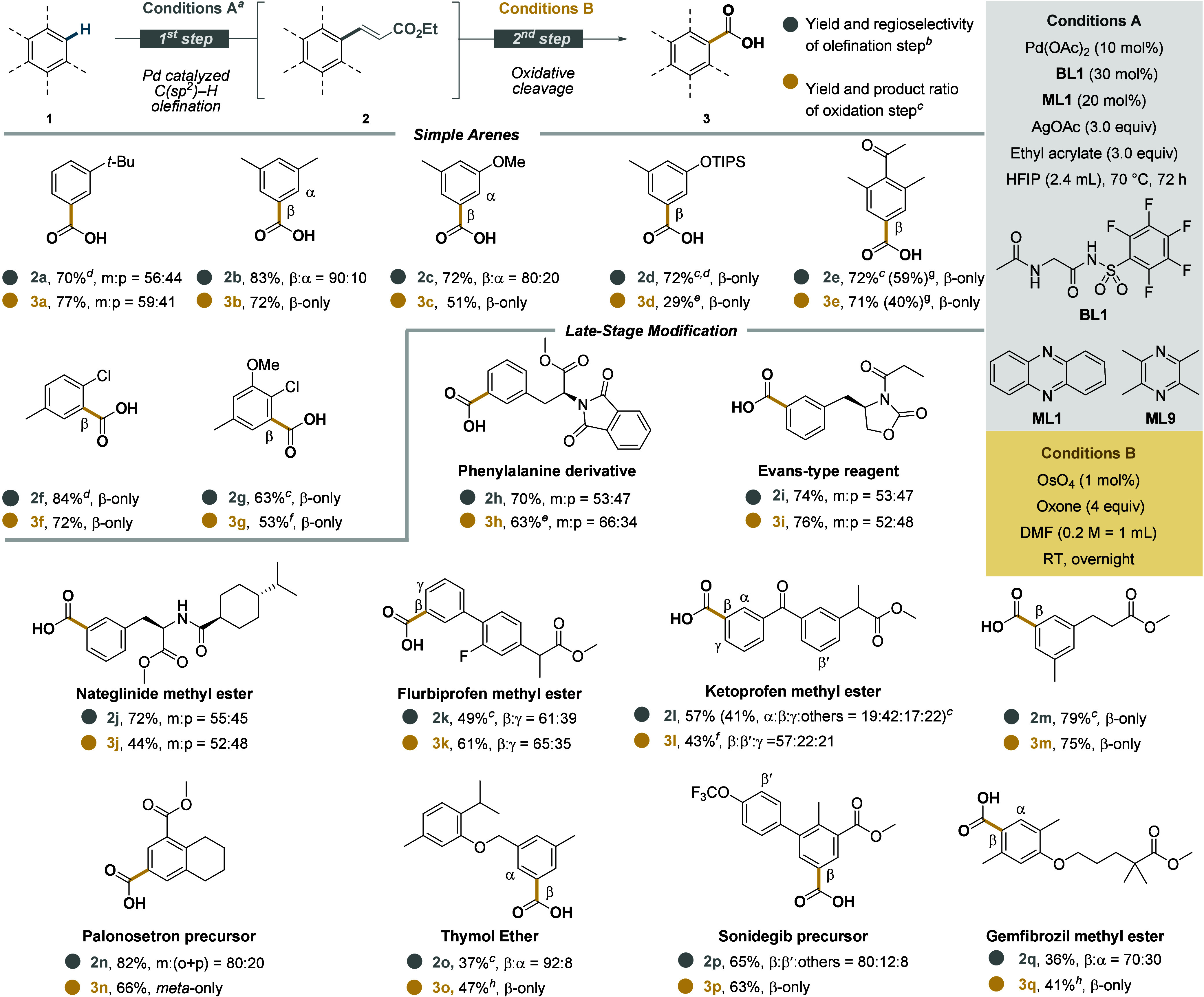
Substrate Scopes for the Synthesis of Carboxylic Acids^a^

a Reactions conducted on a 0.2 mmol scale (the olefination was performed on a 0.2 mmol scale and the crude product underwent oxidative cleavage without intermediate purification). *^b^* Yields and regioisomeric ratios were determined by ^1^H NMR using CH_2_Br_2_ as an internal standard. *^c^* Isolated yields. *^d^* Olefination step with **ML9** (20 mol%) at 70 °C for 48 h. *^e^* Isolated after conversion to the methyl ester. *^f^* OsO_4_ (2 mol %), oxone (6 equiv), 48 h at room temperature. *^g^* 1.0 mmol scale. *^h^* The oxidation was conducted stepwise, synthesizing **4o** and **4q** first (cf. Scheme 3), followed by further oxidation with KMnO_4_.

**Scheme 3:**
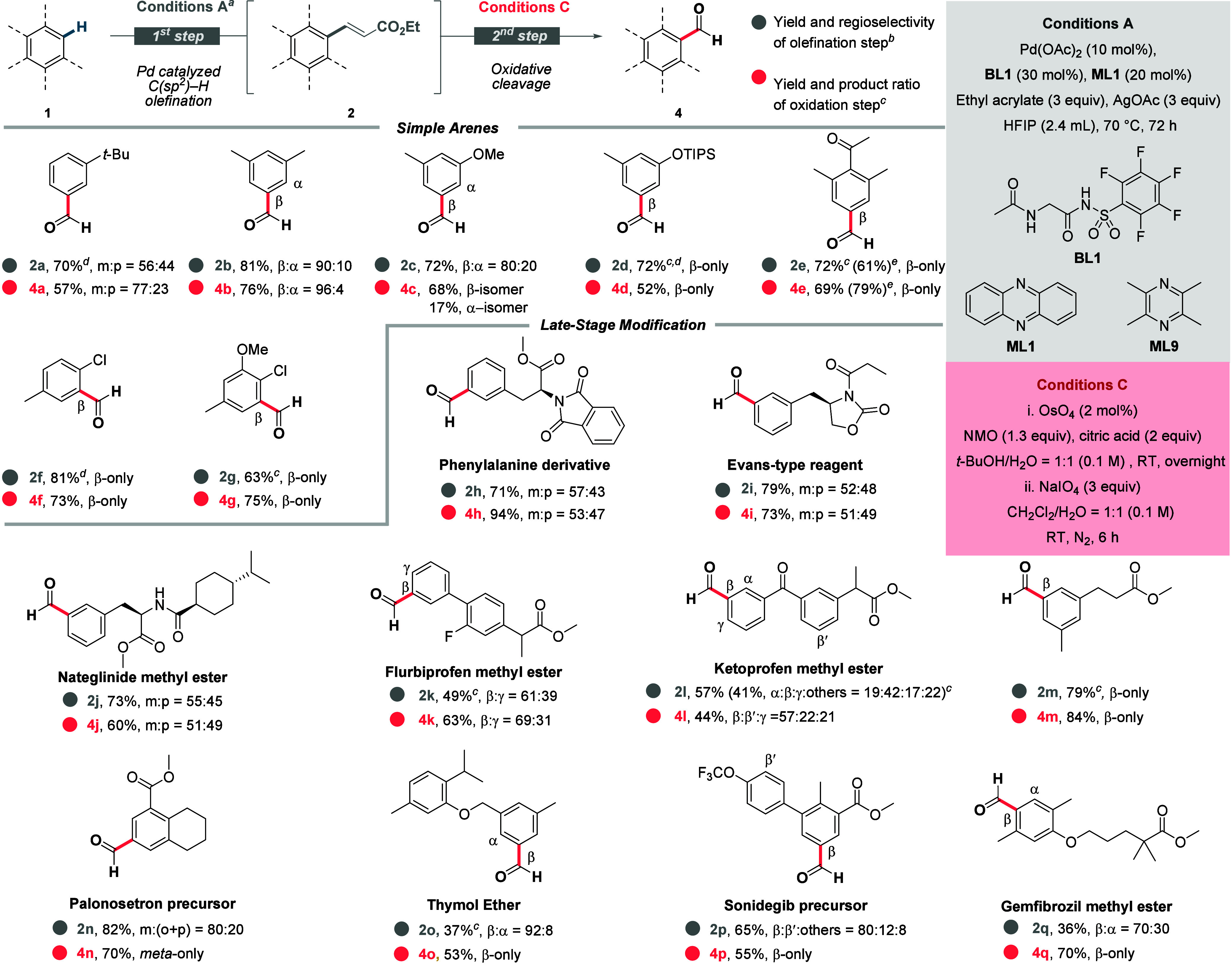
Substrate Scopes for the Synthesis of Aldehydes^a^

a Reactions conducted on a 0.2 mmol scale (the olefination was performed on a 0.2 mmol scale and the crude product underwent oxidative cleavage without intermediate purification). *^b^* Yields and regioisomeric ratios were determined by ^1^H NMR using CH_2_Br_2_ as an internal standard. *^c^* Isolated yields. *^d^* Olefination step with **ML9** (20 mol%) at 70 °C for 48 h; 1.0 mmol scale.

In this work, we disclose an efficient strategy to install formyl and carboxyl groups with sterically controlled regioselectivity, thereby complementing the synthetic repertoire provided by the classical S_E_Ar mechanism. Using a novel dual ligand-based Pd catalyst for nondirected arene C–H olefination, directly followed by one of two divergent oxidative cleavage protocols without an intermediate purification step, gives access to carboxylic acids and aldehydes. The sterically favored products were obtained in moderate to high yields. The protocol was found to feature a broad functional group tolerance and is compatible with bioactive compounds and advanced synthetic intermediates. These features are expected to prove highly useful when combined with traditional arene functionalization, enabling the late-stage generation of regioisomeric product libraries, thereby obviating the need for de novo syntheses or lengthy synthetic sequences to access the target molecules. We thus anticipate that our protocol will serve as a valuable tool enabling the efficient exploration of new chemical space.

## Supplementary Material



## Data Availability

The data underlying this study are available in the published article and its Supporting Information.

## Notes

Experimental procedures and characterization of the compounds obtained (PDF)
