# Supplementary material for: Sterically Controlled C(sp2)–H Carboxylation and Formylation: A Complementary Strategy to SEAr Approaches
Source: Org Lett. 2025 Sep 15;27(38):10849–54. doi: 10.1021/acs.orglett.5c03474 (PMC12481572; doi:10.1021/acs.orglett.5c03474)

Sterically Controlled C(sp^2)–H Carboxylation and Formylation: A Complementary Strategy to S$_E$Ar Approaches

Rita de Jesus,[†,‡] Jyotirmoy Dey,[†,‡] Elisa Chakraborty,[†,‡] Manuel van Gemmeren*,[‡]

[‡] Otto-Diels-Institut für organische Chemie, Otto-Hahn-Platz 4, 24098 Kiel, Germany

[†] These authors contributed equally to this work

* vangemmeren@oc.uni-kiel.de

Table of Contents

1 General Experimental Methods 2
2 Optimization of the Reaction Conditions 4
3 Preparation of Starting Materials and Ligands 15
3.1 Synthesis of Starting Materials 15
3.2 Synthesis of Ligand 26
4 Scope of the Reaction 28
4.1 General Procedures 28
4.2 Characterization of Olefinated Compounds 30
4.3 Characterization of Carboxylic Acids 38
4.4 Characterization of Aldehydes 55
4.5 Scale-up reactions 72
5 Bibliography 74
6 NMR Spectra 77

1 General Experimental Methods

Solvents, Reagents and Techniques

Unless otherwise noted, all reactions were carried out in oven-dried glassware (120 °C). Reaction temperatures refer to the temperature of the aluminum-block surrounding the reaction vessel.

Commercially available chemicals were obtained from ABCR, Acros Organics, BLD-pharm, Alfa Aesar, Fluorochem, Sigma Aldrich, or TCI Europe and used as received. The AgOAc utilized during these studies was purchased from ABCR. A strong dependency of the reaction on the quality and morphology of the AgOAc was observed, leading to varying results with AgOAc purchased from other suppliers. The AgOAc was stored in a glove box and batches for short term use were extracted as required.

HFIP was purchased from fluorochem and directly used it in the reaction. Additional anhydrous solvents (<50 ppm water) were purchased from Fisher Scientific and stored over molecular sieves under inert atmosphere.

Pre-stirring of all the catalyst components in HFIP at room temperature for 10 minutes was found to be crucial to obtain the optimal yield and regioselectivity.

Chromatography

Analytical thin layer chromatography (TLC) was performed on silica gel ALUGRAM Xtra SIL G/UV_{254} plates (Macherey-Nagel) or aluminum oxide 150 F_{254}, neutral plates (Merck). Compounds were visualized by ultraviolet light (254 nm or 366 nm) or by staining with $KMnO_4$ (1 g $KMnO_4$, 6 g K_2CO_3 and 0.1 g KOH in 100 mL of H_2O) or bromocresol green (40 mg bromocresol green in 100 mL EtOH; addition of 0.1M (aq.) NaOH until the blue color appears in the solution) or dinitrophenylhydrazine (DNP) (12 g of 2,4- dinitrophenylhydrazine, 60 mL of conc. sulfuric acid, and 80 mL of water in 200mL of 95% ethanol). Flash chromatography was performed on silica gel 60M (0.04-0.063 mm) or aluminum oxide (aluminum oxide 90, neutral, activity level 1). Positive overpressure was applied. Automated flash chromatography was performed on a Biotage Isolera One system. Compounds were detected by a UV-detector.

Nuclear Magnetic Resonance (NMR) Spectroscopy

^{1}H, ^{13}C, and ^{19}F NMR spectra were recorded at room temperature on a Bruker AvanceNeo 500 or a Bruker Avance 600 device. Chemical shifts (δ) are given relative to tetramethylsilane (TMS) and referenced to residual solvent signals as tabulated by Fulmer et al.[1]Chemical shifts are given with two decimal numbers (^{1}H) or one decimal number (^{13}C, ^{19}F). Data is reported in the following order: Chemical shift (multiplicity [s = singlet, d = doublet, t = triplet, q = quartet, quint = quintet, hept = septet, m = multiplet, br = broad signal], coupling constant (*J* [Hz]) and number of H-atoms). For the spectra of regioisomeric mixtures signals clearly assigned to a particular regioisomer are labelled with a superscript at the integration. The number of protons in such cases refers to the number of protons of the respective isomer. The absence of such an index indicates that the signals of all observed regioisomers overlap, the integration given corresponds to the number of protons in each isomer.

Mixtures of regioisomers were characterized using two-dimensional NMR spectroscopy. By measuring correlation spectroscopy (COSY), heteronuclear single quantum coherence (HSQC), and heteronuclear multiple bond correlation (HMBC) spectra, the proton signals of each regioisomer in the mixture of a respective compound were unambiguously assigned

Exceptions where the assignment of very minor isomers could not be achieved due to weak signal intensity are labelled accordingly.

The ^{13}C-NMR spectra of mixtures are reported as observed. Due to the low signal intensity and potentially an overlap of signals, the number of signals can deviate from the hypothetical value, however, the signals of the major components are clearly recognizable in all cases and correspond to the literature values whenever the respective compounds are literature known. All NMR-spectra were processed using MestReNova.

Infrared spectroscopy (IR) and melting points

IR-spectroscopy was performed on a Perkin Elmer ATR spectrometer. Samples were measured neat. Melting points were determined on a Büchi instrument with automated temperature control. The temperature was raised by 2 °C/ min during measurements.

Mass Spectrometry (MS)

High resolution mass spectra (HRMS) were recorded on a Jeol AccuTOF using electron impact (EI) or a ThermoFisher Orbitrap spectrometer using electron spray ionization (ESI).

Gas Chromatography with Flame Ionization Detection (GC-FID)

GC-FID analysis was performed using an Agilent Technologies 7890B or 8860 instrument with an HP5 column (30 m, 0.32mm × 0.25 µm) and nitrogen as carrier gas.

Gas Chromatography with Mass Spectrometry (GC-MS)

GC-MS was performed on an Agilent Technologies 8890 system coupled to an Agilent Technologies 5977B mass detector (EI) and an HP-5MS column (30 m, 0.32mm × 0.25 µm). Helium was used as carrier gas.

2 Optimization of the Reaction Conditions

General procedure for the reactions carried out during the optimization of the reaction conditions:

An oven dried 10 mL Schlenk tube was charged with a Pd-source, an amino acid-derived ligand, a pyridine ligand, a Ag-source, and HFIP. The reaction vessel was tightly sealed and was pre-stirred at room temperature for 10 minutes. After the pre-stirring 1-methoxy-3-methylbenzene (12.2 mg, 0.100 mmol) and ethyl acrylate were added and the reaction mixture was placed into the inside circle of a preheated aluminum block with a tightly fitting recess on a magnetic stirrer and stirred (1000 rpm stirring speed) at the indicated temperature for the indicated reaction time.

After completion of the reaction time, the reaction mixture was allowed to cool to room temperature. 1,3,5-trimethylbenzene (12.0 mg, 0.100 mmol) was added and the reaction mixture was further diluted with ethyl acetate (4 mL). After homogenizing the reaction mixture, an aliquot (400 µL) of the resulting solution was filtered over a short silica column using ethyl acetate as the eluent and the resulting sample was subjected to GC-FID analysis. All yields and ratios given during the optimization studies were determined by GC-FID analysis of the crude reaction mixture using 1,3,5-trimethylbenzene as an internal standard.

Scheme S2.1: Screening of bidentate ligands (BL)

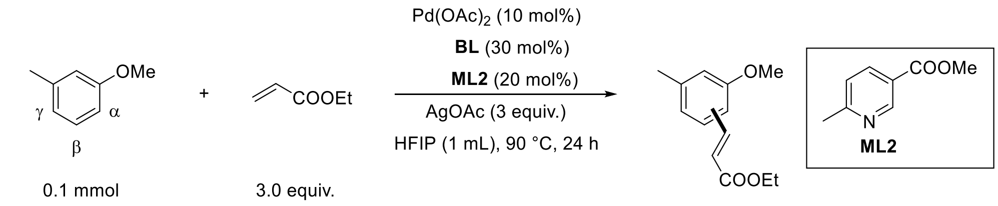

Entry	Bidentate ligand (BL)	Conv.(%)	Yield (%)	α:β:γ
1	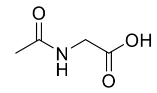 BL2	87	53	35:65:0
2	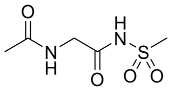 BL3	73	54	35:65:0
3	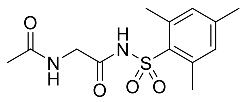 BL4	67	51	60:40:0
4	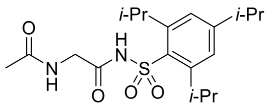 BL5	57	43	50:50:0
5	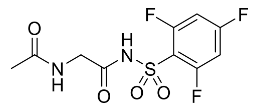 BL6	67	54	30:70:0
6	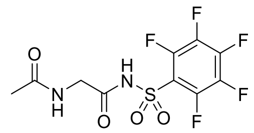 BL1	75	61	28:72:0

Scheme S2.2: Screening of bidentate ligands (BL)

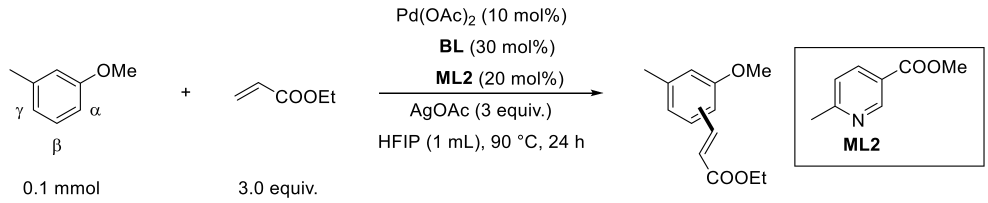

Entry	Bidentate ligand (BL)	Conv.(%)	Yield (%)	α:β:γ
7	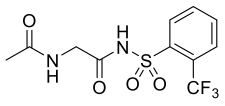 BL7	68	15	40:60:0
8	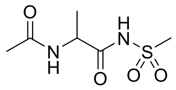 BL8	81	23	39:61:0
9	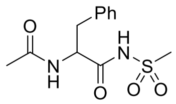 BL9	68	12	66:34:0
10	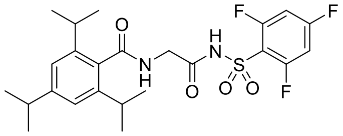 BL10	18	5	47:30:22
11	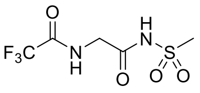 BL11	85	44	82:9:9

Scheme S2.3: Screening of monodentate ligands (ML)

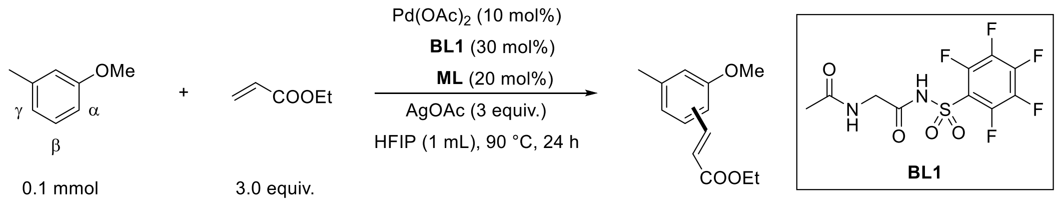

Entry	Monodentate ligand (ML)	Conv.(%)	Yield (%)	α:β:γ
1	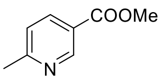 ML2	78	59	28:72:0
2	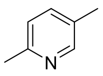 ML3	65	56	31:69:0
3	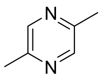 ML4	91	63	28:72:0
4	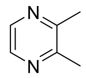 ML5	90	62	26:74:0
5	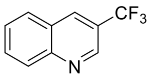 ML6	63	44	27:73:0
6	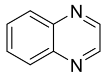 ML7	92	51	23:77:0
7	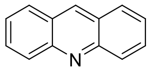 ML8	51	39	42:58:0
8	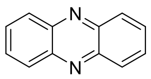 ML1	78	61	23:77:0

Scheme S2.4: Screening of monodentate ligands (ML)

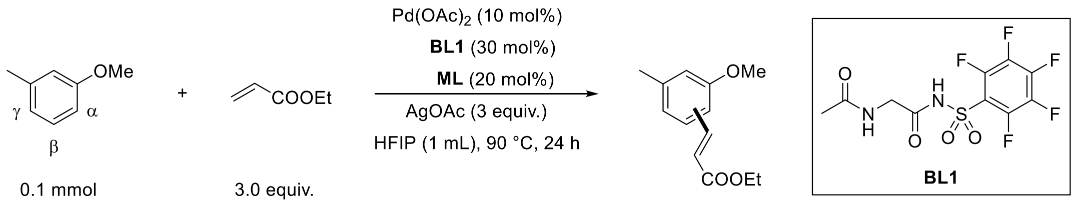

Entry	Monodentate ligand (ML)	Conv.(%)	Yield (%)	α:β:γ
9	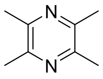 ML9	83	71	28:72:0
10	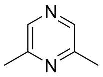 ML10	91	42	32:68:0
11	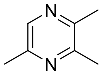 ML11	91	60	27:73:0
12	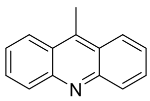 ML12	45	25	39:61:0
13	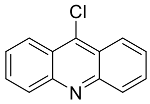 ML13	45	28	37:63:0
14	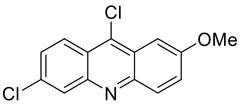 ML14	61	42	27:73:0
15	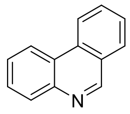 ML15	59	47	27:73:0

Scheme S2.5: Additional bidentate ligand screening with **ML1**

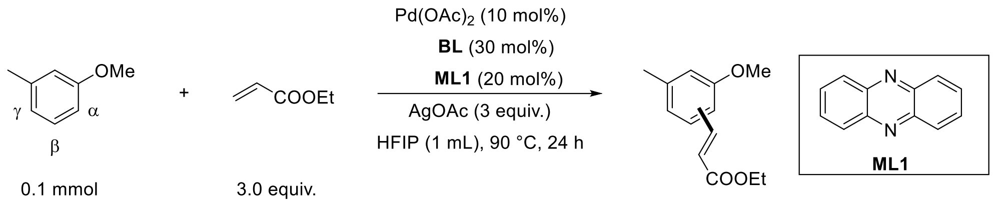

Entry	Bidentate ligand (BL)	Conv.(%)	Yield (%)	α:β:γ
1	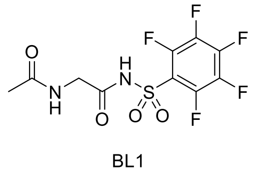 BL1	80	62	23:77:0
2	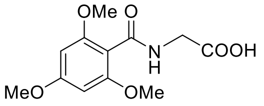 BL12	59	52	23:77:0
3	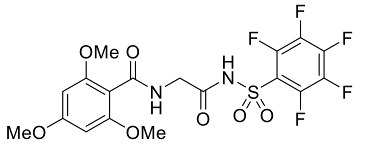 BL13	56	48	23:77:0

Scheme S2.6: Initial temperature and time screening with the most effective monodentate ligands **ML1** and **ML9**

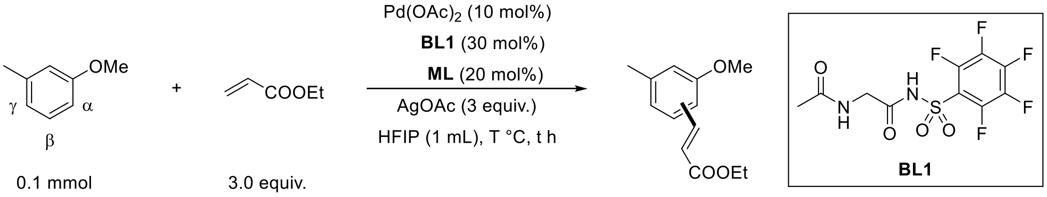

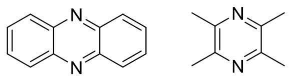

ML1 **ML9**

Entry	ML/ T (°C)/ t (h)	Conv.(%)	Yield (%)	α:β:γ
1	**ML1**/ 90/ 24	78	62	22:78:0
2	**ML1**/ 100/ 18	73	58	22:78:0
3	**ML1**/ 80/ 38	61	50	21:79:0
4	**ML9**/ 90/ 24	80	68	29:71:0
5	**ML9**/ 100/ 18	76	70	29:71:0
6	**ML9**/ 80/ 38	77	64	29:71:0

Scheme S2.7: Initial screening of solvent mixtures with the best performing monodentate ligands **ML1** and **ML9**

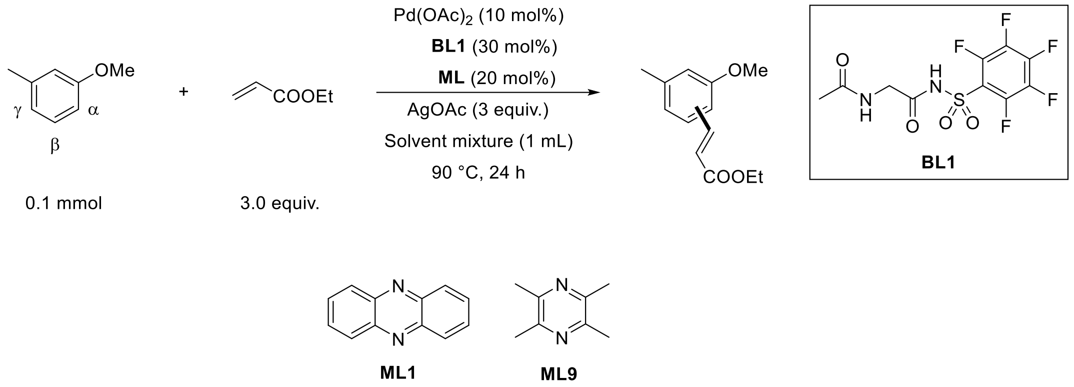

Entry	ML/ Solvent mixture = x:y	Conv.(%)	Yield (%)	α:β:γ
1	**ML1**/ HFIP (ref.)	78	62	22:78:0
2	**ML1**/ HFIP:DCE = 1:1	46	41	22:78:0
3	**ML1**/ HFIP:DCE = 1:9	32	27	23:77:0
4	**ML9**/ HFIP (ref.)	77	65	29:71:0
5	**ML9**/ HFIP:DCE = 1:1	34	29	25:75:0
6	**ML9**/ HFIP:DCE = 1:9	30	27	21:79:0

Scheme S2.8: Additional temperature and time screening with **ML9**

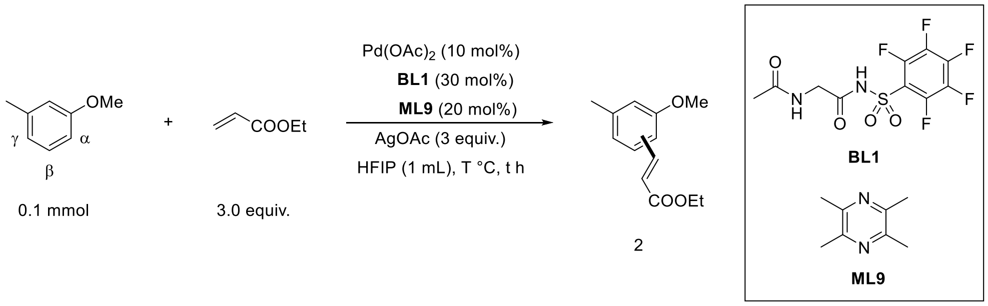

Entry	T (°C)/ t (h)	Conv.(%)	Yield (%)	α:β:γ
1	100/ 18	83	69	29:71:0
2	110/ 18	77	71	27:73:0
3	120/ 18	76	62	29:71:0
4	130/ 18	73	59	28:72:0
5	70/ 48	86	80	30:70:0
6	90/ 48	82	68	28:72:0
7	100/ 48	79	71	28:72:0
8	110/ 48	78	70	28:72:0
9	120 / 48	77	69	27:73:0

Scheme S2. 9: Additional solvent mixture and time screening with **ML9**

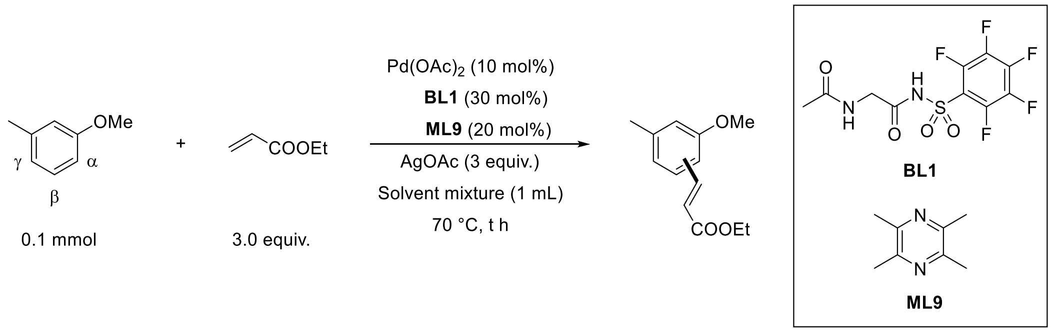

Entry	solvent mixture (2:8)/ t (h)	Conv.(%)	Yield (%)	α:β:γ
1	HFIP (ref.)/ 48	86	80	29:71:0
2	HFIP:DCE/ 48	48	43	21:79:0
3	HFIP:DCE/ 72	57	52	21:79:0
4	TFE:$CHCl_3$/ 48	36	31	18:82:0
5	TFE:$CHCl_3$/ 72	39	33	19.81:0

Scheme S2.10: Temperature screening with **ML9** in HFIP:DCE mixture

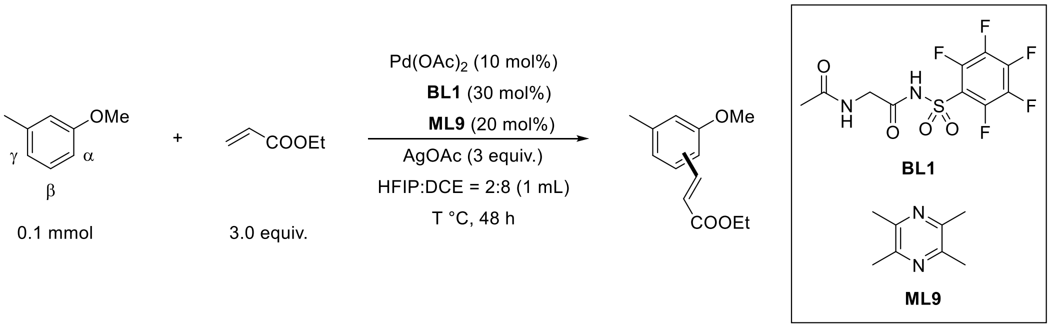

Entry	T (°C)	Conv.(%)	Yield (%)	α:β:γ
1	70	48	43	21:79:0
2	80	55	52	21:79:0
3	90	45	41	20:80:0
4	100	42	34	20:80:0

In case of **ML9,** after using different solvent mixtures in an above-mentioned ratio, the reaction became more regioselective but the overall reactivity decreased in comparison with HFIP as a single solvent. Hence, further screenings were conducted with **ML1**.

Scheme S2.11: Additional temperature and time screening with **ML1**

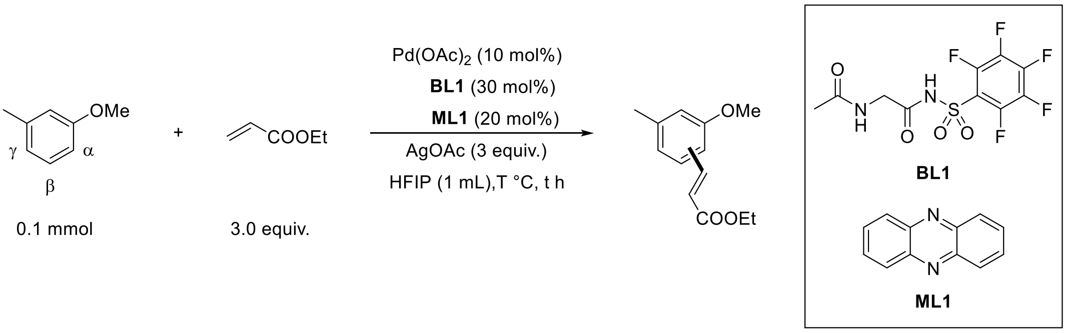

Entry	T (°C)/ t (h)	Conv.(%)	Yield (%)	α:β:γ
1	70/ 48	81	65	22:78:0
2	70/ 72	82	70	22:78:0
3	80/ 48	75	61	20:80:0
4	80/ 72	77	68	21:79:0
5	90/ 24	78	62	21:79:0
6	90/ 48	80	60	20:80:0
7	100/ 48	77	55	21:79:0
8	110/ 48	73	58	21:79:0

Scheme S2.12: Time screening with **ML1** in HFIP:DCE mixture

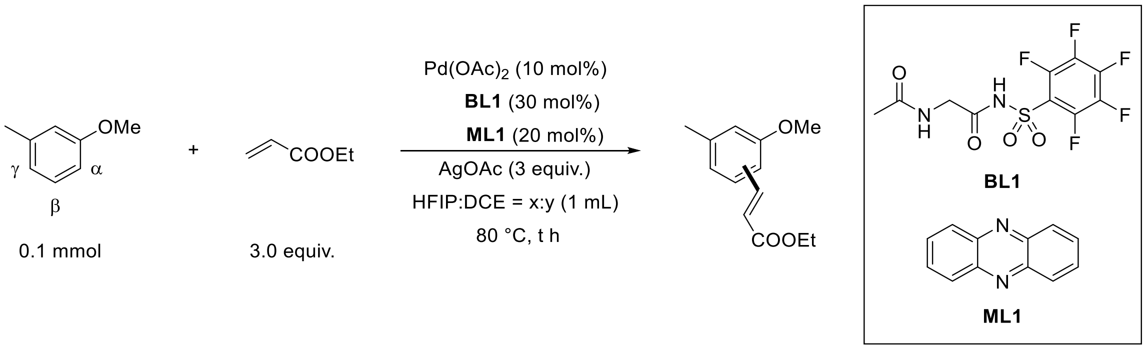

Entry	x:y / t (h)	Conv.(%)	Yield (%)	α:β:γ
1	8:2 / 48	57	49	22:78:0
2	8:2 / 72	65	53	22:78:0
3	1:1 / 48	45	40	21:79:0
4	1:1/ 72	50	42	21:79:0

Scheme S2.13: Concentration screening

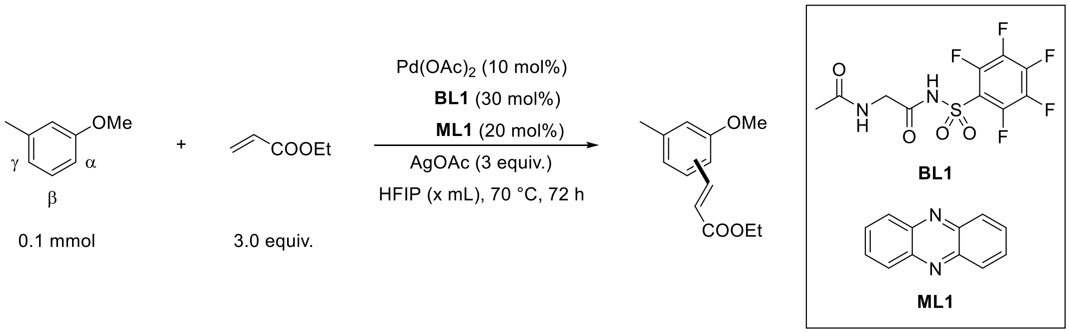

Entry	x mL	Conv.(%)	Yield (%)	α:β:γ
1	0.5	84	60	32:68:0
2	0.8	75	58	25:75:0
3	1	82	66	22:78:0
4	1.2	84	71	21:79:0
5	1.4	81	71	22:78:0
6	1.6	80	68	22:78:0
7	1.8	78	70	22:78:0

Scheme S2.14: Catalyst to ligand ratio screening

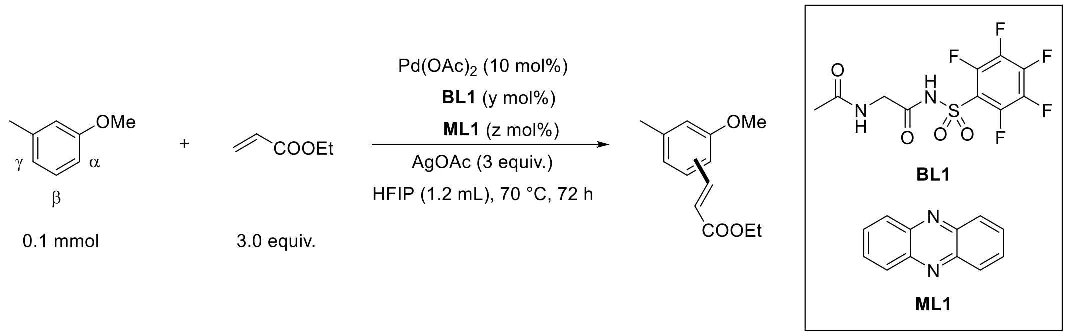

Entry	10:y:z	Conv.(%)	Yield (%)	α:β:γ
1	10:30:20	84	71	21:79:0
2	10:20.20	86	70	29:71:0
3	10:20:30	83	61	34:66:0
4	10:40:30	81	62	22:78:0

Scheme S2.15: Amount of AgOAc screening

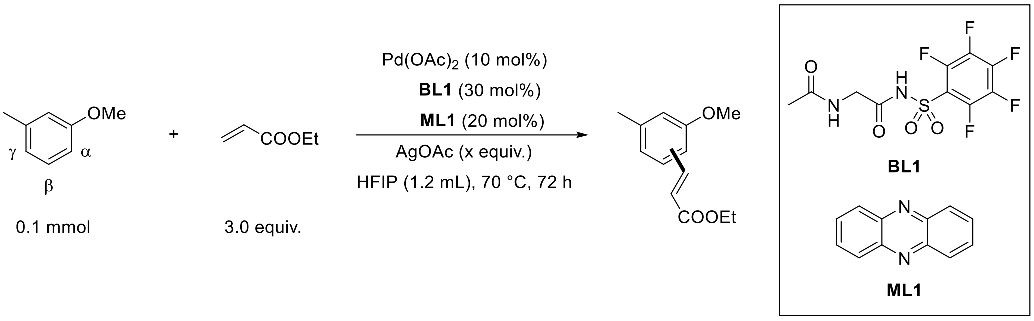

Entry	x equiv.	Conv.(%)	Yield (%)	α:β:γ
1	3	84	71	21:79:0
2	2.5	78	66	24:76:0

Scheme S2.16: Screening of Pd-source

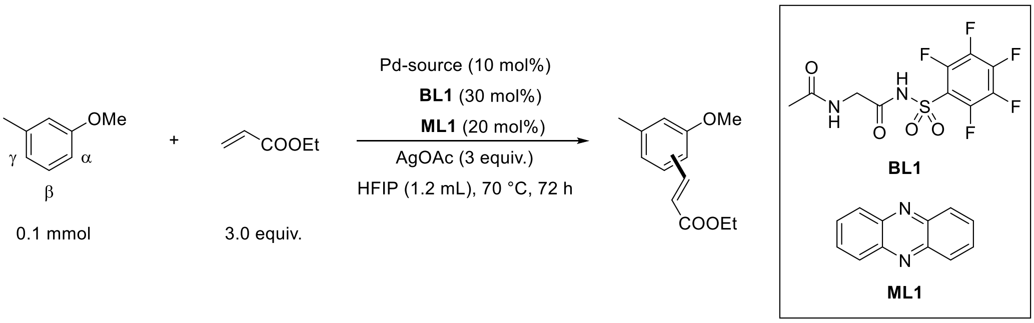

Entry	Pd-source	Conv.(%)	Yield (%)	α:β:γ
1	$Pd(OAc)_2$	83	73	21:79:0
2	$Pd(TFA)_2$	90	46	19:81:0
3	$Pd(CH_3CN)_2Cl_2$	83	62	22:78:0

Scheme S2.17: Catalyst loading screening

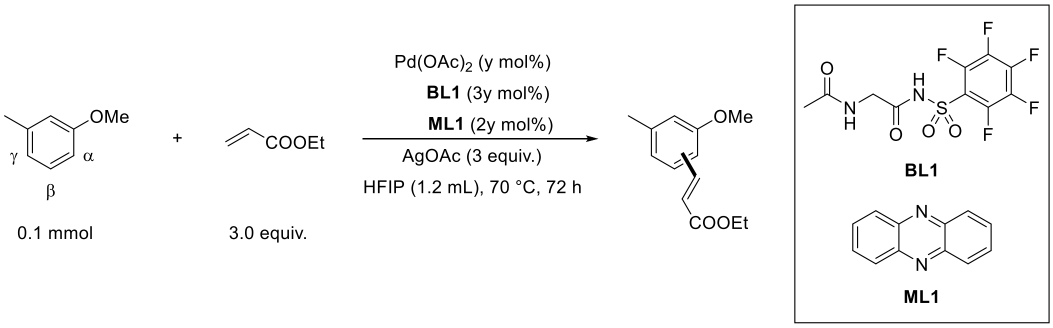

Entry	y mol%	Conv.(%)	Yield (%)	α:β:γ
1	10	84	71	21:79:0
2	5	70	62	29:71:0

3 Preparation of Starting Materials and Ligands

3.1 Synthesis of Starting Materials

General Procedure A: Methyl esterification of the following carboxylic acids

The following compounds were synthesized as previously reported by our group with a slight modification.[2] In a 100 mL round bottom flask, MeI (0.620 mL, 1.42 g, 10.0 mmol, 5.0 equiv) was added to a solution of the corresponding carboxylic acid (2.00 mmol,1.0 equiv) and K_2CO_3 (1.105 g, 8.000 mmol, 4.0 equiv) dissolved in acetone (20 mL, 0.1 M) at room temperature. After stirring the reaction mixture at room temperature for 16 h, saturated $NaHCO_3$ solution (20 mL) was added and the resulting mixture was extracted with EtOAc (3 x 30 mL). The combined organic phases were filtered through Na_2SO_4, concentrated *in vacuo* and the remaining residue was purified by silica gel column chromatography.

Triisopropyl(m-tolyloxy)silane (1d)

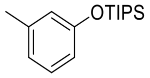

Using a modified literature procedure,[3] in a 100 mL round bottom flask, *m*-cresol (0.310 mL, 324 mg, 3.00 mmol), and triethylamine (1.08 mL, 788 mg, 7.80 mmol, 2.6 equiv) were dissolved in CH_2Cl_2 (30 mL, 0.1 M). Once the reaction mixture was cooled to 0 °C, triisopropylsilyl chloride (0.840 mL, 752 mg, 3.90 mmol, 1.3 equiv) was added dropwise to the solution and then allowed to warm to room temperature in the next 18h. Afterwards the reaction mixture was concentrated *in vacuo.* The target compound **1d** was obtained as a colorless liquid (603 mg, 2.28 mmol, 76%) via silica gel column chromatography using pentane as the eluent. The observed analytical data are in accordance with the ones reported in literature.[3]

^{1}H NMR (500 MHz, $CDCl_3$) δ 7.09 (t, *J* = 7.8 Hz, 1H), 6.77 – 6.73 (m, 1H), 6.72 – 6.70 (m, 1H), 6.70 – 6.67 (m, 1H), 2.30 (brs, 3H), 1.32 – 1.20 (m, 3H), 1.11 (d, *J* = 7.3 Hz, 18H) ppm.

^{13}C NMR (126 MHz, $CDCl_3$) δ 156.1, 139.4, 129.1, 121.9, 120.8, 116.9, 21.5, 18.1, 12.8 ppm.

HRMS (ESI-pos) m/z: Calcd for $C_{16}H_{29}OSi$ $[M+H]^+$ 265.1982, Found 265.1978.

(*S*)-methyl 2-(1,3-dioxoisoindolin-2-yl)-3-phenylpropanoate (1h)

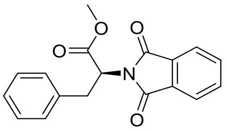

Using a modified literature procedure,[4] in a 100 mL round bottom flask, MeI (0.220 mL, 502 mg, 3.54 mmol,1.23 equiv) was added to a solution of (*S*)-methyl 2-(1,3-dioxoisoindolin-2-yl)-3-phenylpropanoate (851 mg, 2.88 mmol) and K_2CO_3 (529 mg, 3.83 mmol, 1.3 equiv) dissolved in dimethylformamide (30 mL, 0.1 M) at room temperature. After stirring at room temperature for 18 h, the reaction mixture was diluted with CH_2Cl_2 (10 mL) and washed with H_2O (4 x 30 mL). The organic phase was dried over $MgSO_4$, filtered, and concentrated *in vacuo*. The target compound **1h** was obtained as a colorless solid (706 mg, 2.28 mmol, 79%) via silica gel column chromatography using gradient elution (pentane:EtOAc = 70:30 up to pentane: EtOAc = 50:50 as the eluent). The observed analytical data are in accordance with the ones reported in literature.[5]

^{1}H NMR (500 MHz, $CDCl_3$) δ 7.81 – 7.74 (m, 2H), 7.71 – 7.64 (m, 2H), 7.25 – 7.07 (m, 5H), 5.16 (dd, *J* = 11.3, 5.2 Hz, 1H), 3.78 (s, 3H), 3.65 – 3.43 (m, 2H) ppm.

^{13}C NMR (126 MHz, $CDCl_3$) δ 169.5, 167.6, 136.8, 134.2, 131.7, 129.0, 128.7, 127.0, 123.6, 53.4, 53.0, 34.8 ppm.

HRMS (ESI-pos) m/z: Calcd for $C_{18}H_{16}NO_4$ $[M+H]^+$ 310.1074, Found 310.1063.

(*R*)-4-benzyl-3-propionyloxazolidin-2-one (1i)

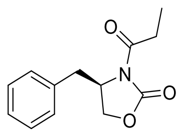

Using a modified literature procedure,[6] a solution of (*R*)-4-benzyl-oxazolidin-2-one (531 mg, 3 mmol) under nitrogen in THF (20 mL, 0.1 M) was cooled to –78 °C using a dry-ice/acetone bath. *n*-BuLi (1.44 mL, 2.5 M, 998 mg, 3.60 mmol, 1.2 equiv) was slowly added dropwise to the solution. Once the addition was complete, the mixture was stirred for 30 min, followed by dropwise addition of propionyl chloride (0.288 mL, 305 mg, 3.30 mmol, 1.1 equiv). The reaction mixture was stirred for 1 h at 0 °C and was then allowed to slowly warm to room temperature over the next 20 h. Subsequently, the reaction was quenched with saturated NH_4Cl aqueous solution, and extracted with EtOAc (3 x 20 mL). The combined organic phases were dried over $MgSO_4$, filtered, and concentrated under reduced pressure. The target compound **1i** was obtained as a colorless solid (574 mg, 2.46 mmol, 82 %) via silica gel column chromatography using gradient elution (pentane:EtOAc = 95:5 up to pentane:EtOAc = 80:20 as the eluent). The observed analytical data are in accordance with the ones reported in literature.[7]

^{1}H NMR (500 MHz, $CDCl_3$) δ 7.36 – 7.31 (m, 2H), 7.30 – 7.25 (m, 1H), 7.23 – 7.19 (m, 2H), 4.71 – 4.64 (m, 1H), 4.23 – 4.14 (m, 2H), 3.30 (dd, *J* = 13.4, 3.3 Hz, 1H), 3.05 – 2.86 (m, 2H), 2.77 (dd, *J* = 13.4, 9.6 Hz, 1H), 1.20 (t, *J* = 7.4 Hz, 3H) ppm.

^{13}C NMR (126 MHz, $CDCl_3$) δ 174.2, 153.6, 135.5, 129.5, 129.1, 127.5, 66.3, 55.3, 38.0, 29.3, 8.4 ppm.

HRMS (ESI-pos) m/z: Calcd for $C_{13}H_{16}O_3N$ $[M+H]^+$ 234.1125, Found 234.1121.

(*R*)-methyl 2-((1*s*,4*S*)-4-isopropylcyclohexanecarboxamido)-3-phenylpropanoate (1j)

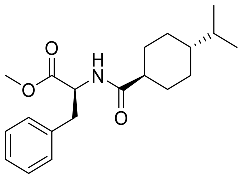

Using a modified literature procedure,[4] in a 100 mL round bottom flask, MeI (0.150 mL, 340 mg, 2.41 mmol,1.23 equiv) was added to a solution of nateglinide (622 mg, 1.96 mmol) and K_2CO_3 (358 mg, 2.59 mmol, 1.32 equiv) dissolved in dimethylformamide (20 mL, 0.1 M) at room temperature. After stirring at room temperature for 18 h, the reaction mixture was diluted with CH_2Cl_2 (10 mL) and washed with H_2O (4 x 20 mL). The organic phase was dried over $MgSO_4$, filtered, and concentrated *in vacuo*. The target compound **1j** was obtained as a colorless solid (562 mg, 1.70 mmol, 87%) via silica gel column chromatography using cyclohexane:EtOAc = 60:40 as the eluent. The observed analytical data are in accordance with the ones reported in literature.[8]

^{1}H NMR (500 MHz, $CDCl_3$) δ 7.32 – 7.22 (m, 3H), 7.10 – 7.05 (m, 2H), 5.87 (d, *J* = 7.7 Hz, 1H), 4.92 – 4.84 (m, 1H), 3.73 (s, 3H), 3.23 – 3.04 (m, 2H), 2.06 – 1.95 (m, 1H), 1.93 – 1.82 (m, 2H), 1.81 – 1.73 (m, 2H), 1.50 – 1.31 (m, 3H), 1.12 – 0.90 (m, 3H), 0.85 (d, *J* = 6.9 Hz, 6H) ppm.

^{13}C NMR (126 MHz, $CDCl_3$) δ 175.7, 172.4, 136.0, 129.5, 128.7, 127.2, 52.8, 52.4, 45.6, 43.4, 38.0, 32.9, 29.9, 29.6, 29.1, 29.0, 19.9 ppm.

HRMS (ESI-pos) m/z: Calcd for $C_{20}H_{30}O_3N$ $[M+H]^+$ 332.2220, Found 332.2212.

Methyl 2-(2-fluoro-[1,1'-biphenyl]-4-yl)propanoate (1k)

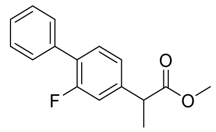

Using a modified literature procedure,[4] in a 100 mL round bottom flask, MeI (0.220 mL, 502 mg, 3.54 mmol,1.18 equiv) was added to a solution of methyl 2-(2-fluoro-[1,1'-biphenyl]-4-yl)propanoate (731 mg, 3.00 mmol) and K_2CO_3 (497 mg, 3.60 mmol, 1.2 equiv) dissolved in dimethylformamide (30 mL, 0.1 M) at room temperature. After stirring at room temperature for 18 h, the reaction mixture was diluted with CH_2Cl_2 (10 mL) and washed with H_2O (4 x 30 mL). The organic phase was dried over $MgSO_4$, filtered, and concentrated *in vacuo*. The target compound **1k** was obtained as a colorless solid (693 mg, 2.68 mmol, 89%) via silica gel column chromatography using gradient elution (pentane:EtOAc = 100:0 up to pentane:EtOAc = 80:20 as the eluent). The observed analytical data are in accordance with the ones reported in literature.[9]

^{1}H NMR (500 MHz, CDCl$_3$) δ 7.57 – 7.52 (m, 2H), 7.47 – 7.34 (m, 4H), 7.17 – 7.10 (m, 2H), 3.77 (q, *J* = 7.1 Hz, 1H), 3.71 (s, 3H), 1.55 (d, *J* = 7.2 Hz, 3H) ppm.

^{13}C NMR (126 MHz, CDCl$_3$) δ 174.6, 159.8 (d, *J* = 248.3 Hz), 141.9 (d, *J* = 7.7 Hz), 135.6, 131.0 (d, *J* = 3.9 Hz), 129.1 (d, *J* = 3.1 Hz), 128.6, 127.8, 123.7 (d, *J* = 3.3 Hz), 115.4 (d, *J* = 23.7 Hz), 52.4, 45.1, 18.6 ppm.

^{19}F NMR (471 MHz, CDCl$_3$) δ –117.6 ppm.

HRMS (ESI-pos) m/z: Calcd for $C_{16}H_{16}O_2F$ $[M+H]^+$ 259.1129, Found 259.1125.

Methyl 2-(3-benzoylphenyl)propanoate (1l):

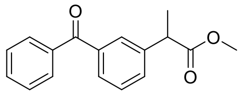

Following the general procedure A using 2-(3-benzoylphenyl)propanoic acid (508 mg, 2.00 mmol) and purification via silica gel column chromatography using pentane:EtOAc = 80:20 as the eluent, the target compound **1l** was obtained as colorless liquid (510 mg, 1.90 mmol, 95%). The observed analytical data are in accordance with the ones reported in literature.[10]

^{1}H NMR (600 MHz, $CDCl_3$) δ 7.80 (dd, J = 8.4, 1.3 Hz, 2H), 7.75 (t, 1H), 7.72 – 7.64 (m, 1H), 7.63 – 7.57 (m, 1H), 7.57 – 7.52 (m, 1H), 7.51 – 7.47 (m, 2H), 7.46 – 7.42 (m, 1H), 3.81 (q, J = 7.2 Hz, 1H), 3.68 (s, 3H), 1.54 (d, J = 7.2 Hz, 3H) ppm.

^{13}C NMR (151 MHz, $CDCl_3$) δ 196.6, 174.7, 141.0, 138.1, 137.6, 132.6, 131.6, 130.2, 129.4, 129.2, 128.7, 128.4, 52.3, 45.4, 18.6 ppm.

HRMS (ESI-pos) m/z: Calcd for $C_{17}H_{17}O_3$ $[M+H]^+$ 269.1172, Found 269.1170.

Methyl 3-(*m*-tolyl)propanoate (1m):

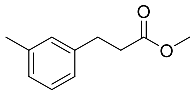

Following the general procedure A using 3-(*m*-tolyl)propanoic acid (328 mg, 2.00 mmol) and purification via silica gel column chromatography using pentane:EtOAc = 96:4 as the eluent, the target compound **1m** was obtained as colorless liquid (250 mg, 1.40 mmol, 70%). The observed analytical data are in accordance with the ones reported in literature.[11]

^{1}H NMR (500 MHz, $CDCl_3$) δ 7.18 (t, *J* = 7.8 Hz, 1H), 7.07 – 6.90 (m, 3H), 3.68 (s, 3H), 2.96 – 2.85 (t, 2H), 2.66 – 2.58 (t, 2H), 2.33 (s, 3H) ppm.

^{13}C NMR (126 MHz, $CDCl_3$) δ 173.6, 140.6, 138.2, 129.2, 128.5, 127.2, 125.4, 51.7, 35.9, 31.0, 21.5 ppm.

HRMS (ESI-pos) m/z: Calcd for $C_{11}H_{15}O_2$ $[M+H]^+$ 179.1067, Found 179.1067.

Methyl 5,6,7,8-tetrahydronaphthalene-1-carboxylate (1n):

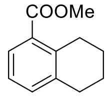

Following the general procedure A using 5,6,7,8-tetrahydronaphthalene-1-carboxylic acid (352 mg, 2.00 mmol) and purification via silica gel column chromatography using pentane:EtOAc = 94:6 as the eluent, the target compound **1n** was obtained as colorless liquid (354 mg, 1.86 mmol, 93%). The observed analytical data are in accordance with the ones reported in literature.[12]

^{1}H NMR (600 MHz, $CDCl_3$) δ 7.64 (m, 1H), 7.22 – 7.19 (m, 1H), 7.16 – 7.08 (m, 1H), 3.87 (s, 3H), 3.11 – 2.96 (m, 2H), 2.85 – 2.69 (m, 2H), 1.88 – 1.69 (m, 4H) ppm.

^{13}C NMR (151 MHz, $CDCl_3$) δ 168.7, 138.7, 138.5, 133.2, 130.4, 127.9, 125.1, 51.9, 30.3, 27.9, 23.2, 22.6 ppm.

HRMS (ESI-pos) m/z: Calcd for $C_{12}H_{15}O_2$ $[M+H]^+$ 191.1067, Found 191.1063.

Methyl 2-methyl-4'-(trifluoromethoxy)-[1,1'-biphenyl]-3-carboxylate (1p):

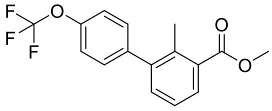

Following the general procedure A using 2-methyl-4'-(trifluoromethoxy)-[1,1'-biphenyl]-3-carboxylic acid (592 mg, 2.00 mmol) and purification via silica gel column chromatography using pentane:EtOAc = 98:2 as the eluent, the target compound **1p** was obtained as colorless liquid (590 mg, 1.90 mmol, 95%). The observed analytical data are in accordance with the ones reported in literature.[13]

^{1}H NMR (500 MHz, Acetone) δ 7.81 (dd, *J* = 7.6, 0.4 Hz, 1H), 7.50 – 7.45 (m, 2H), 7.45 – 7.33 (m, 4H), 3.88 (s, 3H), 2.37 (s, 3H) ppm.

^{13}C NMR (126 MHz, Acetone) δ 168.9, 149.2, 143.1, 141.6, 136.9, 133.9, 132.9, 132.0, 130.3, 126.5, 121.7, 52.3, 18.5 ppm.

^{19}F NMR (471 MHz, Acetone) δ –57.5 ppm.

HRMS (ESI-pos) m/z: Calcd for $C_{16}H_{14}O_3F_3$ $[M+H]^+$ 311.0890, Found 311.0885.

Methyl 5-(2,5-dimethylphenoxy)-2,2-dimethylpentanoate (1q):

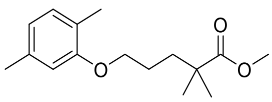

Following the general procedure A using 5-(2,5-dimethylphenoxy)-2,2-dimethylpentanoic acid (500 mg, 2.00 mmol) and purification via silica gel column chromatography using pentane:EtOAc = 96:4 as the eluent, the target compound **1q** was obtained as colorless liquid (505 mg, 1.91 mmol, 95%). The observed analytical data are in accordance with the ones reported in literature.[14]

^{1}H NMR (600 MHz, $CDCl_3$) δ 7.00 (dd, J = 7.5, 0.9 Hz, 1H), 6.66 (dd, J = 7.4, 0.9 Hz, 1H), 6.61 (s, 1H), 3.98 – 3.86 (m, 2H), 3.67 (s, 3H), 2.31 (s, 3H), 2.18 (s, 3H), 1.73 (dd, J = 3.4, 1.8 Hz, 4H), 1.22 (s, 6H) ppm.

^{13}C NMR (151 MHz, $CDCl_3$) δ 178.5, 157.1, 136.6, 130.4, 123.7, 120.8, 112.1, 68.0, 51.9, 42.2, 37.3, 25.3, 21.5, 15.9 ppm.

HRMS (ESI-pos) m/z: Calcd for $C_{16}H_{28}O_3N$ $[M+NH_4]^+$ 282.2064, Found 282.2054.

3.2 Synthesis of Ligand

2-acetamido-*N*-((perfluorophenyl)sulfonyl)acetamide (BL1):

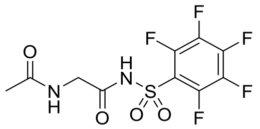

Compound **(BL1)** was synthesized as previously reported by our group with slight modification.[15] DMAP (978 mg, 8.00 mmol, 2.0 equiv) was added to a suspension of EDC·HCl (920 mg, 4.80 mmol, 1.2 equiv) in dry CH_2Cl_2 (10 mL, 0.4 M). The mixture was stirred at room temperature until all the solids dissolved. The mixture was cooled to 0 °C and *N*-acetylglycine (469 mg, 4.00 mmol) was added, followed by the 2,3,4,5,6-pentafluorobenzenesulfonamide (1.19 g, 4.80 mmol, 1.2 equiv). The reaction mixture was stirred for further 1 h at 0 °C and was then allowed to slowly warm to room temperature over the next 19 h. After removing all organic solvents *in vacuo,* the aqueous phase was acidified with conc. aq. HCl to reach a pH value of 1 and extracted with EtOAc (4 x 20 mL). The combined organic phases were dried over Na_2SO_4, filtered, and concentrated under reduced pressure. The crude product was purified via silica gel column chromatography using CH_2Cl_2:MeOH:HCOOH = 96:4:0.5 as the eluent and the target compound **BL1** was obtained as colorless solid (790 mg, 2.28 mmol, 57%).

^{1}H NMR (500 MHz, DMSO) δ 8.11 (t, *J* = 5.7 Hz, 1H), 3.79 (d, *J* = 5.7 Hz, 2H), 1.80 (s, 3H) ppm.

^{13}C NMR (126 MHz, DMSO) δ 169.9, 169.7, 148.8, 145.0, 143.6, 138.4, 136.3, 124.2, 42.4, 22.1 ppm.

^{19}F NMR (471 MHz, DMSO) δ –136.4 (d, *J* = 24.0 Hz), –145.2, –159.7 (t, *J* = 21.9 Hz) ppm.

HRMS (ESI-neg) m/z: Calcd for $C_{10}H_6O_4N_2F_5S_1$ $[M-H]^-$ 344.9974, Found 344.9973.

IR (cm^{-1}): 3371, 3006, 2768, 1726, 1622, 1541, 1520, 1503, 1480, 1434, 1385, 1376, 1300, 1263, 1192, 1156, 1150, 1034, 994, 885, 867, 764.

Melting Point: Sublimation started at 176 °C.

2,4,6-trimethoxy-N-(2-oxo-2-(perfluorophenylsulfonamido)ethyl)benzamide (BL13):

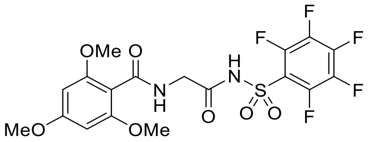

Following the reported literature procedure by our group,[15] using 2-(2,4,6-trimethoxybenzamido)acetic acid **(BL12)** (540 mg, 2.00 mmol) and 2,3,4,5,6-pentafluorobenzenesulfonamide (595 mg, 2.40 mmol, 1.2 equiv), the target compound **BL13** was purified by silica gel column chromatography using CH_2Cl_2:MeOH:HCOOH = 97:3:0.5 as the eluent and obtained as a colorless solid (590 mg, 1.18 mmol, 59%).

^{1}H NMR (500 MHz, DMSO) δ 8.06 (t, *J* = 5.6 Hz, 1H), 6.21 (s, 2H), 3.89 (d, *J* = 5.7 Hz, 2H), 3.78 (s, 3H), 3.68 (s, 6H) ppm.

^{13}C NMR (126 MHz, DMSO) δ 169.3, 164.9, 161.4, 157.8, 145.7, 143.6, 138.3, 136.4, 133.2, 123.1, 108.7, 90.9, 55.7, 55.4, 42.8 ppm.

^{19}F NMR (471 MHz, DMSO) δ –136.3 (d, *J* = 26.1 Hz), –145.3, –159.7 (t, *J* = 21.9 Hz) ppm.

HRMS (ESI-neg) m/z: Calcd for $C_{18}H_{14}O_7N_2F_5S_1$ $[M-H]^-$ 497.0447, Found 497.0449.

IR (cm^{-1}): 3369, 1709, 1635, 1591, 1523, 1495, 1468, 1206.

Melting Point: 160 °C (decomp.)

4 Scope of the Reaction

4.1 General Procedures

General procedure B: Olefination of arenes

An oven dried 10 mL Schlenk tube was charged with $Pd(OAc)_2$ (4.6 mg, 20 µmol, 10 mol%), 2-acetamido-*N*-((perfluorophenyl)sulfonyl)acetamide (**BL1**) (20.8 mg, 60.0 µmol, 30 mol%), Phenazine (**ML1**) (7.2 mg, 40 µmol, 20 mol%), AgOAc (100.2 mg, 0.6000 mmol, 3.0 equiv) and HFIP (2 mL). The reaction mixture was stirred at room temperature for 10 minutes. The arene substrate (0.200 mmol) and ethyl acrylate (60.0 mg, 0.600 mmol, 3 equiv) were added to the reaction mixture, followed by the remaining HFIP (0.4 mL). The reaction vessel was tightly sealed and placed into the inside circle of an aluminum block (preheated to 70 °C) with a tightly fitting recess on a magnetic stirrer. The reaction mixture was stirred with 1000 rpm at this temperature for 72 h. After completion of the reaction, the mixture was allowed to cool to room temperature, filtered through a pad of Celite®, and eluted into a 100 mL round-bottom flask with CH_2Cl_2 (40 mL). All volatiles were removed under reduced pressure. If the corresponding olefinated product was literature unknown, the residue was purified by silica-gel column chromatography.

General procedure C: Olefination of arenes and isolation as the corresponding carboxylic acid

An oven dried 10 mL Schlenk tube was charged with $Pd(OAc)_2$ (4.6 mg, 20 µmol, 10 mol%), 2-acetamido-*N*-((perfluorophenyl)sulfonyl)acetamide (**BL1**) (20.8 mg, 60.0 µmol mmol, 30 mol%), Phenazine (**ML1**) (7.2 mg, 40 µmol, 20 mol%), AgOAc (100.2 mg, 0.6000 mmol, 3.0 equiv) and HFIP (2 mL). The reaction mixture was stirred at room temperature for 10 minutes. The arene substrate (0.200 mmol) and ethyl acrylate (60.0 mg, 0.600 mmol, 3 equiv) were added to the reaction mixture, followed by the remaining HFIP (0.4 mL). The reaction vessel was tightly sealed and placed into the inside circle of an aluminum block (preheated to 70 °C) with a tightly fitting recess on a magnetic stirrer. The reaction mixture was stirred with 1000 rpm at this temperature for 72 h. After completion of the reaction, the mixture was allowed to cool to room temperature, filtered through a pad of Celite® and eluted into a 100 mL round-bottom flask with CH_2Cl_2 (40 mL). All volatiles were removed under reduced pressure. CH_2Br_2 was added as an internal standard and the yield and regioisomeric ratios of corresponding olefinated product were determined by ^{1}H-NMR spectroscopy. If the corresponding olefinated product had previous literature precedence and/or was identified as a single regioisomer in the crude ^{1}H-NMR spectrum, the residue was used in the next step without further purification. The sample from the NMR tube was transferred back into the same flask and concentrated *in vacuo*. Following a modified literature procedure,[16] the crude mixture from the previous step was dissolved in DMF (1 mL, 0.2 M). OsO_4 (63 µL, 2.0 µmol, 1.0 mol%, 2.5 (w/w)% in *t*-BuOH) and Oxone® (492 mg, 0.800 mmol, 4.0 equiv) were added and the mixture was stirred at room temperature for 18 h. Na_2SO_3 (152 mg, 1.20 mmol, 6.0 equiv) and H_2O (20 mL) were added to reduce the remaining Os (VIII) and the resulting mixture was stirred at room temperature for one hour. EtOAc (30 mL) was added, the resulting mixture was transferred into a separatory funnel and the phases were separated. The organic phase was washed with 1 N HCl (2 x 10 mL) and sat. NaCl solution (3 x 10 mL). The organic phase was dried over Na_2SO_4, filtered, and concentrated under reduced pressure. The crude product was purified via silica gel column chromatography. The isolated yield for the oxidative cleavage was calculated based on the ^{1}H-NMR yield of the previous olefination step.

General procedure D: Olefination of arenes and isolation as the corresponding aldehyde

An oven dried 10 mL Schlenk tube was charged with $Pd(OAc)_2$ (4.6 mg, 20 µmol, 10 mol%), 2-acetamido-*N*-((perfluorophenyl)sulfonyl)acetamide (**BL1**) (20.8 mg, 60.0 µmol, 30 mol%), Phenazine (**ML1**) (7.2 mg, 40 µmol, 20 mol%), AgOAc (100.2 mg, 0.6000 mmol, 3.0 equiv) and HFIP (2 mL). The reaction mixture was stirred at room temperature for 10 minutes. The arene substrate (0.200 mmol) and ethyl acrylate (60.0 mg, 0.600 mmol, 3 equiv) were added to the reaction mixture, followed by the remaining HFIP (0.4 mL). The reaction vessel was tightly sealed and placed into the inside circle of an aluminum block (preheated to 70 °C) with a tightly fitting recess on a magnetic stirrer. The reaction mixture was stirred with 1000 rpm at this temperature for 72 h. After completion of the reaction, the mixture was allowed to cool to room temperature, filtered through a pad of Celite® and eluted into a 100 mL round-bottom flask with CH_2Cl_2 (40 mL). All volatiles were removed under reduced pressure. CH_2Br_2 was added as an internal standard and the yield and regioisomeric ratios of corresponding olefinated product were determined by ^{1}H-NMR spectroscopy. If the corresponding olefinated product had previous literature precedence and/or was identified as a single regioisomer in the crude ^{1}H-NMR spectrum, the residue was used in the next step without further purification. The sample from the NMR tube was transferred back into the same flask and concentrated *in vacuo*. The crude mixture from the previous step was dissolved in *t*-BuOH (1 mL). OsO_4 (126 µL, 4.00 µmol, 2.00 mol%, 2.50 (w/w)% in *t*-BuOH), 4-Methylmorpholine *N*-oxide (30.5 mg, 0.260 mmol, 1.3 equiv) and citric acid (76.9 mg, 0.400 mmol, 2.0 equiv) were added to the reaction mixture, followed by distilled H_2O (1 mL). The mixture was stirred at room temperature for 18 h. Na_2SO_3 (152 mg, 1.20 mmol, 6.0 equiv) and H_2O (20 mL) were added to reduce the remaining Os (VIII) and the resulting mixture was stirred at room temperature for one hour. The aqueous phase was extracted with CH_2Cl_2 (3 x 20 mL). The combined organic phases were dried over Na_2SO_4, filtered, and concentrated under reduced pressure. The residue was used in the next step without further purification.

The crude mixture from the previous step was dissolved in CH_2Cl_2 (1 mL). $NaIO_4$ (128.4 mg, 0.6000 mmol, 3.0 equiv) was added and followed by H_2O (1 mL). The mixture was stirred at room temperature under N_2 atmosphere for 6 h or until full conversion of the starting material was observed by TLC. The reaction mixture was filtered through Na_2SO_4 or $MgSO_4$, eluted with CH_2Cl_2 (40 mL), concentrated under reduced pressure, and purified via silica gel column chromatography. The isolated yield of the oxidative cleavage was calculated based on the ^{1}H-NMR yield of previous olefination step.

4.2 Characterization of Olefinated Compounds

(*E*)-ethyl 3-(3-methyl-5-((triisopropylsilyl)oxy)phenyl)acrylate (2d)

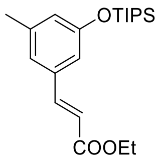

Following a slightly modified general procedure B, using $Pd(OAc)_2$ (4.6 mg, 20 µmol, 10 mol%), 2-acetamido-*N*-((perfluorophenyl)sulfonyl)acetamide (**BL1**) (20.8 mg, 60.0 µmol, 30 mol%), 2,3,5,6-tetramethylpyrazine (**ML9**) (5.5 mg, 40 µmol, 20 mol%), AgOAc (100.2 mg, 0.6000 mmol, 3.0 equiv), HFIP (2.4 mL) and triisopropyl(m-tolyloxy)silane (53.1 mg, 0.201 mmol) as substrate at 70°C for 48 h**.** The target compound **2d** was obtained as a colorless oil (52.7 mg, 72%, β as a single regioisomer). The product **2d** was purified by silica gel column chromatography using gradient elution (pentane:Et_2O = 98:2 up to pentane:Et_2O = 91:9 as the eluent). The observed analytical data are in accordance with the ones reported in literature.[17]

^{1}H NMR (600 MHz, $CDCl_3$) δ 7.59 (d, *J* = 16.0 Hz, 1H), 6.92 (m, 1H), 6.84 (m, 1H), 6.73 (m, 1H), 6.36 (d, *J* = 16.0 Hz, 1H), 4.26 (q, *J* = 7.1 Hz, 2H), 2.30 (brs, 3H), 1.33 (t, *J* = 7.1 Hz, 3H), 1.30 – 1.21 (sept, *J* = 7.8 Hz, 3H), 1.10 (d, *J* = 7.6 Hz, 18H) ppm.

^{13}C NMR (151 MHz, $CDCl_3$) δ 167.2, 156.5, 144.9, 139.9, 135.6, 122.9, 122.1, 118.1, 116.3, 60.6, 21.4, 18.0, 14.5, 12.8 ppm.

HRMS (ESI-pos) m/z: Calcd for $C_{21}H_{38}NO_3Si$ $[M+NH_4]^+$ 380.2616**,** Found 380.2605.

(*E*)-ethyl 3-(4-acetyl-3,5-dimethylphenyl)acrylate (2e):

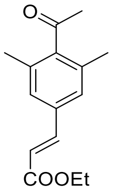

Following the general procedure B and using 1-(2,6-dimethylphenyl)ethanone (29.6 mg, 0.200 mmol) as substrate, the target compound **2e** was obtained as a colorless oil (35.4 mg, 72%, β as a single regioisomer). The product **2e** was purified by silica gel column chromatography using pentane:EtOAc = 96:4 as the eluent.

^{1}H NMR (600 MHz, $CDCl_3$) δ 7.59 (d, *J* = 16.0 Hz, 1H), 7.18 (d, *J* = 0.6 Hz, 2H), 6.42 (d, *J* = 16.0 Hz, 1H), 4.26 (q, *J* = 7.1 Hz, 2H), 2.47 (s, 3H), 2.26 (s, 6H), 1.33 (t, *J* = 7.1 Hz, 3H) ppm.

^{13}C NMR (151 MHz, $CDCl_3$) δ 208.0, 167.2, 144.4, 144.1, 134.9, 133.4, 127.8, 119.2, 60.9, 32.3, 19.5, 14.6 ppm.

HRMS (ESI-pos) m/z: Calcd for $C_{15}H_{19}O_3$ $[M+H]^+$ 247.1329, Found 247.1325.

IR (cm^{-1}): 2980, 2924,2871, 1697,1635, 1445,1353,1322.

(*E*)-ethyl 3-(2-chloro-3-methoxy-5-methylphenyl)acrylate (2g):

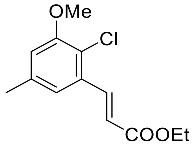

Following the general procedure B and using 1-chloro-2-methoxy-4-methylbenzene (31.3 mg, 0.200 mmol) as substrate, the target compound **2g** was obtained as a colorless oil (32.0 mg, 63%, β as a single regioisomer). The product **2g** was purified by silica gel column chromatography using pentane:EtOAc = 99:1 as the eluent.

^{1}H NMR (600 MHz, $CDCl_3$) δ 8.10 (d, *J* = 16.0 Hz, 1H), 7.04 (dd, *J* = 1.3, 0.6 Hz, 1H), 6.80 – 6.69 (m, 1H), 6.40 (d, *J* = 16.0 Hz, 1H), 4.27 (q, *J* = 7.1 Hz, 2H), 3.89 (s, 3H), 2.35 (s, 3H), 1.34 (t, *J* = 7.1 Hz, 3H) ppm.

^{13}C NMR (151 MHz, $CDCl_3$) δ 166.7, 155.3, 140.9, 137.4, 133.8, 121.2, 120.7, 120.0, 114.2, 60.8, 56.4, 21.6, 14.4 ppm.

HRMS (ESI-pos) m/z: Calcd for $C_{13}H_{16}O_3Cl$ $[M+H]^+$ 255.0783, Found 255.0776.

IR (cm^{-1}): 2981, 1713, 1637, 1575, 1454, 1275.

(*E*)-ethyl 3-(2'-fluoro-4'-(1-methoxy-1-oxopropan-2-yl)-[1,1'-biphenyl]-3-yl)acrylate (2k-β) and (*E*)-ethyl 3-(2'-fluoro-4'-(1-methoxy-1-oxopropan-2-yl)-[1,1'-biphenyl]-4-yl)acrylate (2k-γ)

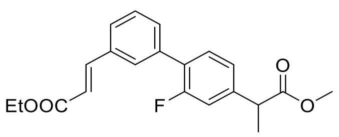

2k-β

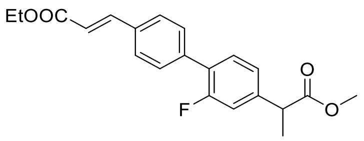

2k-γ

Following the general procedure B and using methyl 2-(2-fluoro-[1,1'-biphenyl]-4-yl)propanoate (51.7 mg, 0.200 mmol) as substrate, the target compound **2k** was obtained as a colourless oil (35.2 mg, 49%, β:γ = 61:39). The product **2k** was purified by silica gel column chromatography using gradient elution (pentane:Et_2O = 95:5 up to pentane:Et_2O = 85:15 as the eluent).

^{1}H NMR (600 MHz, $CDCl_3$) δ 7.75 – 7.70 (m, 1H$^{\beta}$+1H$^{\gamma}$), 7.68 – 7.67 (m, 1H$^{\beta}$), 7.60 – 7.58 (m, 2H$^{\gamma}$), 7.57 – 7.56 (m, 2H$^{\gamma}$), 7.56 – 7.52 (m, 1H$^{\beta}$), 7.52 – 7.51 (m, 1H$^{\beta}$), 7.47 – 7.43 (m, 1H$^{\beta}$), 7.42 – 7.36 (m, 1H$^{\beta}$+1H$^{\gamma}$), 7.18 – 7.14 (m, 1H$^{\beta}$+1H$^{\gamma}$), 7.15 – 7.12 (m, 1H$^{\beta}$+1H$^{\gamma}$), 6.50 – 6.45 (m, 1H$^{\beta}$+1H$^{\gamma}$), 4.30 – 4.25 (m, 2H$^{\beta}$+2H$^{\gamma}$), 3.80 – 3.74 (m, 1H$^{\beta}$+1H$^{\gamma}$), 3.72 – 3.69 (m, 3H$^{\beta}$+3H$^{\gamma}$), 1.55 – 1.52 (m, 3H$^{\beta}$+3H$^{\gamma}$), 1.37 – 1.32 (m, 3H$^{\beta}$+3H$^{\gamma}$) ppm.

^{13}C NMR (151 MHz, $CDCl_3$) δ 174.5, 167.0(8), 167.0(2), 160.7 – 158.8 (m), 144.4, 144.1, 142.6 – 142.4 (m), 137.5, 136.3, 134.8, 133.9, 130.8(2), 130.7(9), 130.7(0), 129.5, 129.1, 128.7, 128.3, 127.3, 127.1, 123.8, 118.9, 118.6, 115.6 – 115.3 (m), 60.7, 52.4, 45.0, 18.5, 14.4 ppm.

^{19}F NMR (471 MHz, $CDCl_3$) δ –117.1, –117.4 ppm.

HRMS (ESI-pos) m/z: Calcd for $C_{21}H_{22}FO_4$ $[M+H]^+$ 357.1497, Found 357.1487.

(*E*)-ethyl 3-(3-(3-(1-methoxy-1-oxopropan-2-yl)benzoyl)phenyl)acrylate (2l-β), (*E*)-ethyl 3-(4-(3-(1-methoxy-1-oxopropan-2-yl)benzoyl)phenyl)acrylate (2l-γ) and (*E*)-ethyl 3-(3-benzoyl-5-(1-methoxy-1-oxopropan-2-yl)phenyl)acrylate (2l-β') :

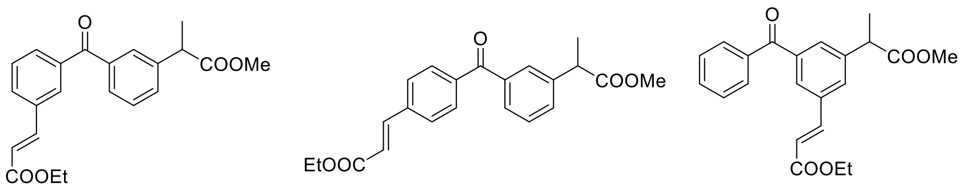

2l-β **2l-γ** **2l-β'**

Following the general procedure B and using methyl 2-(3-benzoylphenyl)propanoate (53.6 mg, 0.200 mmol) as substrate, the target compound **2l** was obtained as a yellowish oil (36.4 mg, 50%). The product **2l** was purified by silica gel column chromatography using pentane:EtOAc = 92:8 as the eluent. Due to the partial overlap of some signals in the ^{1}H NMR spectra of **2l**, it was not possible to assign all protons from each regioisomers unambiguously. Hence, the product **2l** was repurified by silica gel column chromatography using pentane:EtOAc = 85:15 as the eluent. A major fraction containing predominantly a mixture of **2l-β**, **2l-γ,** and **2l-β'** was isolated as an inseparable mixture of grease and *cis* configured isomers in the form of a colorless oil (26.1 mg containing 18.4 mg, 25% of *trans* olefinated product with β:γ:β' = 60:25:15, 3.7 mg, 5% of *cis* olefinated product[1]). Another minor fraction containing predominantly a mixture of **2l-α** and **2l-α'** was isolated as an inseparable mixture of grease in the form of a colorless oil (9.3 mg containing 7.8 mg, 11% of *trans* olefinated product with α:α' = 66:34). Accordingly, the overall yield was calculated to be 41% with α:β:γ:others = 19:42:17:22[2].

Characterization of the major fraction:

^{1}H NMR (600 MHz, C_6D_6) δ 7.94 – 7.91 (m, $1H^{\beta}$), 7.90 – 7.88 (m, $1H^{\gamma}$), 7.85 – 7.83 (m, $1H^{\beta'}$), 7.77 – 7.74 (m, $1H^{\beta}$), 7.73 – 7.68 (m, $1H^{\beta}$+$1H^{\gamma}$+$1H^{\beta'}$+$2H^{\beta'}$), 7.64 – 7.60 (m, $1H^{\beta}$+$1H^{\beta'}$), 7.58 (d, *J* = 0.4 Hz, $2H^{\gamma}$), 7.52 (m, $1H^{\beta}$+$1H^{\gamma}$), 7.49 – 7.47 (m, $1H^{\beta'}$), 7.38 – 7.35 (m, $1H^{\beta'}$), 7.33 – 7.30 (m, $1H^{\gamma}$), 7.30 – 7.27 (m, $1H^{\beta}$+$1H^{\gamma}$), 7.10 – 7.06 (m, $1H^{\beta}$), 7.04 – 6.96 (m, $1H^{\beta}$+$1H^{\gamma}$+$2H^{\gamma}$+$2H^{\beta'}$), 6.88 (dd, $1H^{\beta}$), 6.38 (m, $1H^{\beta}$+$1H^{\gamma}$+$1H^{\beta'}$), 5.86 – 5.70 (m, $5H^{cis}$), 4.14 – 4.04 (m, $2H^{\beta}$+$2H^{\gamma}$+$2H^{\beta'}$), 3.50 – 3.43 (m, $1H^{\beta}$+$1H^{\gamma}$), 3.43 – 3.38 (m, $1H^{\beta'}$), 3.27 (s, $3H^{\beta}$), 3.24 (s, $3H^{\gamma}$), 3.23 (s, $3H^{\beta'}$), 1.31 (m, $3H^{\beta}$+$3H^{\gamma}$), 1.27 (d, *J* = 7.2 Hz, $3H^{\beta'}$), 1.07 – 0.99 (m, $3H^{\beta}$+$3H^{\gamma}$+$3H^{\beta'}$) ppm.

^{13}C NMR (151 MHz, C_6D_6) δ 194.5, 173.6, 165.8(4), 165.7(8), 143.1, 142.9, 142.9, 141.3, 139.0, 138.6, 138.3, 138.0, 137.9, 137.8, 137.4, 135.1, 134.6, 132.2, 131.3, 131.1, 131.0(3), 131.0, 130.4, 130.3, 129.9, 129.7, 129.6, 129.4, 129.3(6), 129.2, 128.8, 128.7, 128.6, 128.5, 128.4, 128.2, 124.4, 124.3, 120.7, 120.0, 119.8, 119.7, 60.2, 60.1, 51.3, 51.2, 45.1(2), 45.0(9), 44.9, 18.3(1), 18.2(7), 18.2, 14.0, 14.0 ppm.

---

[1] Only the characteristic *cis* olefinic proton (with *J* = 12-13 Hz) of each regioisomer was assigned. Due to extensive overlap and the weak intensity of several signals, unambiguous assignment of the remaining signals belonging to *cis* olefinated regioisomers was not possible.

[2] *Cis* isomers were not considered in calculating the regioisomer distribution, since it was assumed all regioisomers isomerize to *cis* in a similar degree and the individual *cis*-regioisomers could not be reliably assigned.

(*E*)-ethyl 3-(2-(3-(1-methoxy-1-oxopropan-2-yl)benzoyl)phenyl)acrylate (2l-α) and (*E*)-ethyl 3-(4-benzoyl-2-(1-methoxy-1-oxopropan-2-yl)phenyl)acrylate (2l-α') :

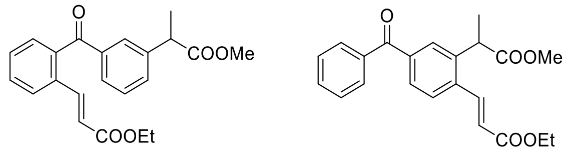

2l-α **2l-α'**

Note: The regioisomeric assignment of **2l-α′** was based on the observation that the proton signal at 3.33 ppm exhibited only a single HMBC correlation to the carbon bearing a proton at 7.03 ppm. Furthermore, the alternative regioisomeric position placing the olefin between the alkyl and carbonyl substituents is sterically substantially more hindered hindered compared to the α'-position**.**

Characterization of the minor fraction:

^{1}H NMR (600 MHz, C_6D_6) δ 8.23 (d, *J* = 15.9 Hz, 1H$^{\alpha}$), 8.19 (d, *J* = 15.9 Hz, 1H$^{\alpha'}$), 7.94 (dd, *J* = 2.1, 1.5 Hz, 1H$^{\alpha}$), 7.76 – 7.65 (m, 2H$^{\alpha'}$), 7.47 (m, 1H$^{\alpha}$), 7.30 – 7.26 (m, 1H$^{\alpha}$+3H$^{\alpha'}$), 7.24 (m, 1H$^{\alpha'}$), 7.07 – 7.05 (m, 1H$^{\alpha}$), 7.05 – 7.02 (m, 1H$^{\alpha'}$), 6.95 – 6.91(m, 1H$^{\alpha}$+2H$^{\alpha'}$), 6.91 (d, *J* = 7.7 Hz, 1H$^{\alpha}$), 6.87 – 6.84 (m 1H$^{\alpha}$), 6.40 (m, 1H$^{\alpha}$+1H$^{\alpha'}$), 3.95 (m, 2H$^{\alpha}$+2H$^{\alpha'}$), 3.40 (q, *J* = 7.2 Hz, 1H$^{\alpha}$), 3.35 (q, *J* = 7.1 Hz, 1H$^{\alpha'}$), 3.23 (s, 3H$^{\alpha}$), 3.23 (s, 3H$^{\alpha'}$), 1.27 (d, *J* = 7.2 Hz, 3H$^{\alpha}$), 1.24 (d, *J* = 7.2 Hz, 3H$^{\alpha'}$), 0.90 (m, 3H$^{\alpha}$+3H$^{\alpha'}$) ppm.

^{13}C NMR (151 MHz, C_6D_6) δ 196.3, 196.2, 173.9, 173.7, 165.9, 142.2, 141.9, 141.8, 141.4, 140.3, 139.7, 138.4, 137.9, 134.7, 133.4, 133.2, 132.3, 130.7, 130.5, 129.7(4), 129.6(6), 129.6(0), 129.5(7), 129.0, 128.9, 128.7, 127.5, 121.7, 121.5, 60.4, 51.6(5), 51.5(8), 45.4, 45.2, 18.6, 18.5, 14.4, 14.2 ppm.

HRMS (ESI-pos) m/z: Calcd for $C_{22}H_{23}O_5$ $[M+H]^+$ 367.1540, Found 367.1533.

IR (cm^{-1}): 2982, 1735,1717, 1660,1638.

(*E*)-ethyl 3-(3-(3-methoxy-3-oxopropyl)-5-methylphenyl)acrylate (2m):

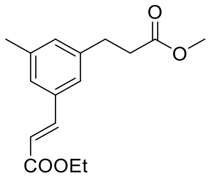

Following the general procedure B and using Methyl 3-(m-tolyl)propanoate (35.6 mg, 0.200 mmol) as substrate, the target compound **2m** was obtained as a colorless oil (43.6 mg, 79%, β as a single regioisomer). The product **2m** was purified by silica gel column chromatography using pentane:EtOAc = 96:4 as the eluent.

^{1}H NMR (600 MHz, $CDCl_3$) δ 7.62 (d, *J* = 16.0 Hz, 1H), 7.22 – 7.17 (m, 1H), 7.18 – 7.12 (m, 1H), 7.07 – 6.96 (m, 1H), 6.41 (d, *J* = 16.0 Hz, 1H), 4.25 (q, *J* = 7.1 Hz, 2H), 3.67 (s, 3H), 2.92 (t, *J* = 7.8 Hz, 2H), 2.62 (t, *J* = 7.4 Hz, 2H), 2.34 (s, 3H), 1.33 (t, *J* = 7.1 Hz, 3H) ppm.

^{13}C NMR (151 MHz, $CDCl_3$) δ 173.3, 167.2, 144.8, 141.3, 138.9, 134.8, 131.3, 126.9, 125.3, 118.2, 60.6, 51.8, 35.6, 30.8, 21.4, 14.5 ppm.

HRMS (ESI-pos) m/z: Calcd for $C_{16}H_{21}O_4$ $[M+H]^+$ 277.1434, Found 277.1430.

IR (cm^{-1}): 2981, 1735, 1707, 1636, 1600, 1437, 1366, 1259.

(*E*)-ethyl 3-(3-((2-isopropyl-5-methylphenoxy)methyl)-5-methylphenyl)acrylate (2o-β) and (*E*)-ethyl 3-(2-((2-isopropyl-5-methylphenoxy)methyl)-4-methylphenyl)acrylate (2o-α)

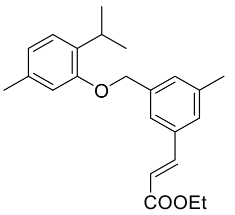

2o-β

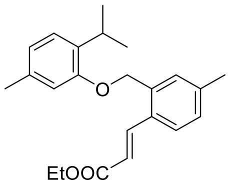

2o-α

Following the general procedure B and using 1-isopropyl-4-methyl-2-((3-methylbenzyl)oxy)benzene (50.9 mg, 0.200 mmol) as substrate, the target compound **2o** was obtained as a colorless oil (26.3 mg, 37%, β:α = 92:8). The product **2o** was purified by silica gel column chromatography using gradient elution (pentane:Et_2O = 98:2 up to pentane:Et_2O = 96:4 as the eluent).

^{1}H NMR (600 MHz, $CDCl_3$) δ 7.99 (d, *J* = 15.8 Hz, $1H^{\alpha}$), 7.69 (d, *J* = 16.0 Hz, $1H^{\beta}$), 7.57 (d, *J* = 8.0 Hz, $1H^{\alpha}$), 7.44 – 7.41 (m, $1H^{\beta}$), 7.37 – 7.35 (m, $1H^{\alpha}$), 7.33 – 7.30 (m, $1H^{\beta}$), 7.30 – 7.28 (m, $1H^{\beta}$), 7.21 – 7.18 (m, $1H^{\alpha}$), 7.16 – 7.12 (m, $1H^{\beta}+1H^{\alpha}$), 6.83 – 6.77 (m, $1H^{\beta}+1H^{\alpha}$), 6.76 – 6.73 (m, $1H^{\beta}+1H^{\alpha}$), 6.46 (d, *J* = 16.0 Hz, $1H^{\beta}$), 6.39 (d, *J* = 15.8 Hz, $1H^{\alpha}$), 5.14 – 5.13 (m, $2H^{\alpha}$), 5.06 – 5.03 (m, $2H^{\beta}$), 4.28 (q, *J* = 7.1 Hz, $2H^{\beta}$), 4.24 (q, *J* = 7.1 Hz, $2H^{\alpha}$), 3.38 (hept, *J* = 6.9 Hz, $1H^{\beta}$), 3.34 – 3.27 (m, $1H^{\alpha}$), 2.43 – 2.39 (m, $3H^{\beta}$), 2.36 – 2.35 (m, $3H^{\alpha}$), 2.34 – 2.33 (m, $3H^{\beta}$), 1.35 (t, *J* = 7.1 Hz, $3H^{\beta}$), 1.30 (t, *J* = 7.1 Hz, $3H^{\alpha}$), 1.25 (d, *J* = 6.9 Hz, $6H^{\beta}$), 1.19 (d, *J* = 6.9 Hz, $6H^{\alpha}$) ppm.

^{13}C NMR (151 MHz, $CDCl_3$) δ 167.2, 167.0, 155.8, 155.7, 144.7, 141.3, 138.9, 138.5, 136.5, 136.4, 134.8, 134.5, 130.8, 130.0, 129.9, 129.3, 128.1, 126.9, 126.1, 124.2, 121.8, 119.5, 118.5, 112.9(1), 112.8(6), 69.8, 68.2, 60.6(2), 60.5(7), 26.7, 26.5, 23.0(0), 22.9(6), 21.6, 21.5, 14.5, 14.4 ppm.

HRMS (ESI-pos) m/z: Calcd for $C_{23}H_{32}NO_3$ $[M+NH_4]^+$ 370.2377**,** Found 370.2365.

4.3 Characterization of Carboxylic Acids

3-(*tert*-butyl)benzoic acid (3a-*meta*) and 4-(*tert*-butyl)benzoic acid (3a-*para*):

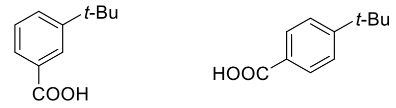

3a-*meta* **3a-*para***

Following a slightly modified general procedure C, using $Pd(OAc)_2$ (4.6 mg, 20 µmol, 10 mol%), 2-acetamido-*N*-((perfluorophenyl)sulfonyl)acetamide (**BL1**) (20.8 mg, 60.0 µmol, 30 mol%), 2,3,5,6-tetramethylpyrazine (**ML9**) (5.5 mg, 40 µmol, 20 mol%), AgOAc (100.2 mg, 0.6000 mmol, 3.0 equiv), HFIP (2.4 mL), and *tert*-butylbenzene (26.8 mg, 0.200 mmol) as substrate at 70°C for 48 h. ^{1}H-NMR yield and regioisomeric ratios of the intermediate olefinated compound **2a** were determined as 70%, m:p = 56:44 respectively (additionally 17% of di-olefinated product were observed). The assignment of **2a-*meta*** and **2a-*para*** from the crude reaction mixture was achieved by comparison with the literature.[18] From the subsequent step, the target compound **3a** was purified by silica gel column chromatography using gradient elution (pentane:EtOAc:HCOOH = 100:0:1 up to pentane:EtOAc:HCOOH = 98:1:1 as the eluent) and obtained as a colorless crystalline solid (19.2 mg, 77%, m:p = 58:42). The analytical data of **3a-*meta*** and **3a-*para*** were in accordance to the ones reported in literature.[19, 20]

^{1}H NMR (500 MHz, $CDCl_3$) δ 8.16 (s, $1H^m$), 8.05 (d, *J* = 8.4 Hz, $2H^p$), 7.94 (dd, *J* = 7.7, 1.2 Hz, $1H^m$), 7.66 (dd, *J* = 7.7, 1.6 Hz, $1H^m$), 7.50 (d, *J* = 7.8 Hz, $2H^p$), 7.41 (t, *J* = 7.8 Hz, $1H^m$), 1.37 (s, $9H^m$), 1.36 (s, $9H^p$) ppm.

^{13}C NMR (126 MHz, $CDCl_3$) δ 172.2, 171.9, 157.7, 151.8, 131.1, 130.3, 129.1, 128.4, 127.6, 127.2, 126.6, 125.6, 35.4, 35.0, 31.4, 31.3 ppm.

HRMS (ESI-neg) m/z: Calcd for $C_{11}H_{13}O_2$ $[M-H]^-$ 177.0921, Found 177.0913.

3,5-dimethylbenzoic acid (3b):

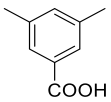

Following the general procedure C and using *m*-xylene (21.2 mg, 0.200 mmol) as substrate. ^{1}H-NMR yield and regioisomeric ratios of the intermediate olefinated compound **2b** were determined as 83%, β:α = 90:10 respectively. The assignment of **2b-α** and **2b-β** from the crude reaction mixture were achieved by comparison with the literature.[21] From the subsequent step, the target compound **3b** was purified by silica gel column chromatography using pentane:EtOAc:HCOOH = 96:4:0.5 as the eluent and obtained as a colorless crystalline solid (18.0 mg, 72%, β as a single regioisomer). The analytical data of **3b** were in accordance to the ones reported in literature.[22]

^{1}H NMR (500 MHz, $CDCl_3$) δ 7.73 (s, 2H), 7.24 (s, 1H), 2.38 (s, 6H) ppm.

^{13}C NMR (126 MHz, $CDCl_3$) δ 172.0, 138.2, 135.4, 129.1, 127.9, 21.1 ppm.

HRMS (ESI-neg) m/z: Calcd for $C_9H_9O_2$ $[M-H]^-$ 149.0608, Found 149.0596.

3-methoxy-5-methylbenzoic acid (3c):

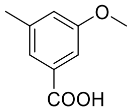

Following the general procedure C and using 1-methoxy-3-methylbenzene (24.4 mg, 0.200 mmol) as substrate. ^{1}H-NMR yield and regioisomeric ratios of the intermediate olefinated compound **2c** were determined as 72%, β:α = 80:20 respectively. The assignment of **2c-α** and **2c-β** from the crude reaction mixture were achieved by comparison with the literature.[23, 24] From the subsequent step, the target compound **3c** was purified by silica gel column chromatography using pentane:EtOAc:HCOOH = 95:5:0.5 as the eluent and obtained as a colorless crystalline solid (12.3 mg, 51%, β as a single regioisomer). The analytical data of **3c** were in accordance to the ones reported in literature.[25]

^{1}H NMR (500 MHz, $CDCl_3$) δ 7.57 – 7.53 (m, 1H), 7.45 – 7.41 (m, 1H), 7.00 – 6.94 (m, 1H), 3.85 (s, 3H), 2.39 (s, 3H) ppm.

^{13}C NMR (126 MHz, $CDCl_3$) δ 171.9, 159.6, 139.8, 130.3, 123.5, 121.2, 111.5, 55.4, 21.4 ppm.

Methyl 3-methyl-5-((triisopropylsilyl)oxy)benzoate (3d)

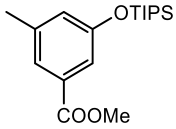

Following a slightly modified general procedure C, using $Pd(OAc)_2$ (4.6 mg, 20 μmol, 10 mol%), 2-acetamido-N-((perfluorophenyl)sulfonyl)acetamide (**BL1**) (20.8 mg, 60.0 μmol, 30 mol%), 2,3,5,6-tetramethylpyrazine (**ML9**) (5.5 mg, 40 μmol, 20 mol%), AgOAc (100.2 mg, 0.6000 mmol, 3.0 equiv), HFIP (2.4 mL), and triisopropyl(m-tolyloxy)silane (**1d**) (53.1 mg, 0.201 mmol) as substrate at 70°C for 48 h. ^{1}H-NMR yield and regioisomeric ratios of the intermediate olefinated compound **2d** were determined as 79%, β as a single regioisomer respectively. From the subsequent step, once washes were performed and the organic phase concentrated under reduced pressure, the crude mixture was dissolved in acetone (5 mL). K_2CO_3 (185 mg, 1.34 mmol, 6.7 equiv) and MeI (0.25 mL, 568 mg, 4.0 mmol, 20 equiv) were added. The mixture was stirred at room temperature for 16 h followed by removal of all volatiles under reduced pressure.[2] The target compound **3d** was purified by silica gel column chromatography using gradient elution (pentane:Et_2O = 100:0 up to pentane:Et_2O = 99:1 as the eluent) and obtained as a light yellow oil (14.9 mg, 29%, β as a single regioisomer).

^{1}H NMR (600 MHz, $CDCl_3$) δ 7.45 – 7.43 (m, 1H), 7.34 – 7.32 (m, 1H), 6.89 – 6.88 (m, 1H), 3.89 (s, 3H), 2.33 (brs, 3H), 1.27 (sept, *J* = 7.4 Hz, 3H), 1.10 (d, *J* = 7.5 Hz, 18H) ppm.

^{13}C NMR (151 MHz, $CDCl_3$) δ 167.3, 156.1, 139.6, 131.2, 125.5, 123.1, 118.0, 52.2, 21.4, 18.1, 12.8 ppm.

HRMS (ESI-pos) m/z: Calcd for $C_{18}H_{31}O_3Si$ $[M+H]^+$ 323.2037, Found 323.2027.

IR (cm^{-1}): 2946, 2867, 2893, 1725, 1594, 1325.

4-acetyl-3,5-dimethylbenzoic acid (3e):

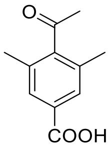

Following the general procedure C and using 1-(2,6-dimethylphenyl)ethanone (29.6 mg, 0.200 mmol) as substrate. ^{1}H-NMR yield and regioisomeric ratios of the intermediate olefinated compound **2e** were determined as 75%, β as a single regioisomer respectively. From the subsequent step, the target compound **3e** was purified by silica gel column chromatography using pentane:EtOAc:HCOOH = 88:12:0.5 as the eluent and obtained as a colorless solid (20.5 mg, 71%, β as a single regioisomer). The analytical data of **3e** were in accordance to the ones reported in literature.[26]

^{1}H NMR (500 MHz, $CDCl_3$) δ 7.77 (s, 2H), 2.50 (s, 3H), 2.31 (s, 6H) ppm.

^{13}C NMR (126 MHz, $CDCl_3$) δ 207.5, 170.3, 147.3, 132.8, 129.6, 129.0, 31.7, 19.0 ppm.

HRMS (ESI-neg) m/z: Calcd for $C_{11}H_{11}O_3$ $[M-H]^-$ 191.0714, Found 191.0704.

2-chloro-5-methylbenzoic acid (3f):

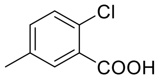

Following the modified general procedure C, using $Pd(OAc)_2$ (4.6 mg, 20 μmol, 10 mol%), 2-acetamido-*N*-((perfluorophenyl)sulfonyl)acetamide (**BL1**) (20.8 mg, 60.0 μmol, 30 mol%), 2,3,5,6-tetramethylpyrazine (**ML9**) (5.5 mg, 40 μmol, 20 mol%), AgOAc (100.2 mg, 0.6000 mmol, 3.0 equiv), HFIP (2.4 mL), and 1-chloro-4-methylbenzene (25.3 mg, 0.200 mmol) as substrate at 70°C for 48 h. ^{1}H-NMR yield and regioisomeric ratios of the intermediate olefinated compound **2f** were determined as 84%, β as a single regioisomer respectively (additionally 10% of di-olefinated product were observed). The assignment of **2f** from the crude reaction mixture was achieved by comparison with the report from our group.[18] From the subsequent step, the target compound **3f** was purified by silica gel column chromatography using pentane:EtOAc:HCOOH = 96:4:0.5 as the eluent and obtained as a colorless crystalline solid (20.5 mg, 72%, β as a single regioisomer). The spectral data of **3f** were in accordance to the ones reported in literature.[27]

^{1}H NMR (500 MHz, $CDCl_3$) δ 7.85 – 7.81 (m, 1H), 7.37 (d, *J* = 8.2 Hz, 1H), 7.32 – 7.26 (m, 1H), 2.38 (s, 3H) ppm.

^{13}C NMR (126 MHz, $CDCl_3$) δ 170.7, 136.8, 134.4, 132.9, 131.6, 131.2, 128.0, 20.7 ppm.

HRMS (ESI-neg) m/z: Calcd for $C_8H_6O_2Cl$ $[M-H]^-$ 169.0051, Found 169.0053.

2-chloro-3-methoxy-5-methylbenzoic acid (3g):

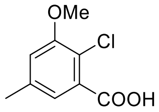

Following the modified general procedure C and using 1-chloro-2-methoxy-4-methylbenzene (31.3 mg, 0.200 mmol) as substrate. ^{1}H-NMR yield and regioisomeric ratios of the intermediate olefinated compound **2g** were determined as 66%, β as a single regioisomer respectively. From the subsequent step using OsO_4 (126 μL, 4.00 μmol, 2.00 mol%, 2.50 (w/w)% in *t*-BuOH), Oxone® (738 mg, 1.20 mmol, 6.0 equiv), and DMF (1 mL, 0.2 M) at room temperature for 48 h, the target compound **3g** was purified by silica gel column chromatography using pentane:EtOAc:HCOOH = 97:3:1.5 as the eluent and obtained as a colorless solid (14.0 mg, 53%, β as a single regioisomer).

^{1}H NMR (500 MHz, $CDCl_3$) δ 7.36 (d, *J* = 1.2 Hz, 1H), 6.93 (d, *J* = 1.5 Hz, 1H), 3.92 (s, 3H), 2.38 (s, 3H) ppm.

^{13}C NMR (126 MHz, $CDCl_3$) δ 170.1, 155.6, 137.5, 129.8, 123.9, 119.9, 116.6, 56.5, 21.3 ppm.

HRMS (ESI-neg) m/z: Calcd for $C_9H_8O_3Cl$ $[M-H]^-$ 199.0168, Found 199.0157.

IR (cm^{-1}): 2981, 1737, 1713, 1365.

Melting Point: 140 °C (decomp.)

(*S*)-methyl 3-(2-(1,3-dioxoisoindolin-2-yl)-3-methoxy-3-oxopropyl)benzoate (3h-*meta*) and (*S*)-methyl 4-(2-(1,3-dioxoisoindolin-2-yl)-3-methoxy-3-oxopropyl)benzoate (3h-*para*)

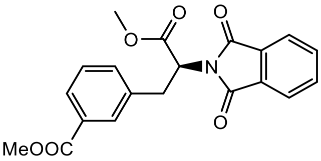

3h-*meta*

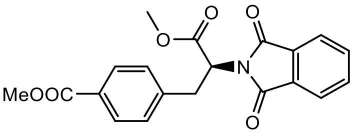

3h-*para*

Following the general procedure C and using (*S*)-methyl 2-(1,3-dioxoisoindolin-2-yl)-3-phenylpropanoate (**1h**) (61.9 mg, 0.200 mmol) as substrate. ^{1}H-NMR yield and regioisomeric ratios of the intermediate olefinated compound **2h** were determined as 70%, m:p = 53:47 respectively. The assignment of **2h** from the crude reaction mixture was achieved by comparison with the literature.[28] From the subsequent step, once washes were performed and the organic phase concentrated under reduced pressure, the crude mixture was dissolved in acetone (5 mL). K_2CO_3 (185 mg, 1.34 mmol, 6.7 equiv) and MeI (0.25 mL, 568 mg, 4.0 mmol, 20 equiv) were added. The mixture was stirred at room temperature for 16 h followed by removal of all volatiles under reduced pressure.[2] The target compound **3h** was purified by silica gel column chromatography using gradient elution (pentane:EtOAc = 95:5 up to pentane: EtOAc = 80:20 as the eluent) and obtained as a yellow oil (32.3 mg, 63%, m:p = 66:34). The spectral data of **3h** were in accordance to the ones reported in literature.[29]

^{1}H NMR (600 MHz, $CDCl_3$) δ 7.87 – 7.85 (m, $2H^p$), 7.85 – 7.84 (m, $1H^m$), 7.82 (ddd, *J* = 9.2, 2.9, 1.4 Hz, $1H^m$), 7.79 – 7.75 (m, $2H^m$+$2H^p$), 7.71 – 7.67 (m, $2H^m$+$2H^p$), 7.35 (ddd, *J* = 9.0, 3.0, 1.4 Hz, $1H^m$), 7.28 – 7.25 (m, $1H^m$), 7.25 – 7.23 (m, $2H^p$), 5.19 – 5.13 (m, $1H^m$+$1H^p$), 3.84 (s, $3H^p$), 3.82 (s, $3H^m$), 3.78 (s, $3H^m$+$3H^p$), 3.68 – 3.55 (m, $2H^m$+$2H^p$) ppm.

^{13}C NMR (151 MHz, $CDCl_3$) δ 169.2(2), 169.1(6), 167.5, 166.9(4), 166.8(7), 142.2, 137.3, 134.4, 134.3, 133.5, 131.6(4), 131.5(6), 130.6(0), 130.2, 130.0(1), 129.0(5), 128.9(7), 128.8, 128.4, 123.7(0), 123.6(8), 53.2, 53.1(4), 53.1(0), 52.9, 52.2, 52.1, 34.8, 34.6 ppm.

HRMS (ESI-pos) m/z: Calcd for $C_{20}H_{21}N_2O_6$ $[M+NH_4]^+$ 385.1394, Found 385.1383.

(*R*)-3-((2-oxo-3-propionyloxazolidin-4-yl)methyl)benzoic acid (3i-*meta*) and (*R*)-4-((2-oxo-3-propionyloxazolidin-4-yl)methyl)benzoic acid (3i-*para*)

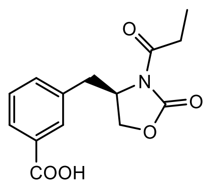

3i-*meta*

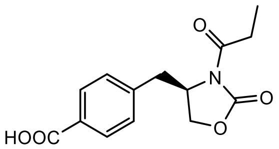

3i-*para*

Following the general procedure C and using (*R*)-4-benzyl-3-propionyloxazolidin-2-one (**1i**) (46.7 mg, 0.200 mmol) as substrate. ^{1}H-NMR yield and regioisomeric ratios of the intermediate olefinated compound **2i** were determined as 74%, m:p = 53:47 respectively. The assignment of **2i** from the crude reaction mixture was achieved by comparison with the report from our group.[30] From the subsequent step, the target compound **3i** was purified by silica gel column chromatography using gradient elution (pentane:Et_2O:HCOOH = 64:35:1 up to pentane:Et_2O:HCOOH = 54:45:1 as the eluent) and obtained as a light brown solid (31.2 mg, 76%, m:p = 52:48).

^{1}H NMR (600 MHz, $CDCl_3$) δ 8.07 (d, *J* = 7.6 Hz, 2H^p), 8.02 (dt, *J* = 6.7, 1.9 Hz, 1H^m), 7.95 (brs, 1H^m), 7.48 – 7.44 (m, 2H^m), 7.33 (d, *J* = 8.0 Hz, 2H^p), 4.75 – 4.70 (m, 1H^m+1H^p), 4.27 – 4.23 (m, 1H^m+1H^p), 4.17 – 4.13 (m, 1H^m+1H^p), 3.38 – 3.32 (m, 1H^m+1H^p), 3.03 – 2.87 (m, 3H^m+3H^p), 1.22 – 1.18 (m, 3H^m+3H^p) ppm.

^{13}C NMR (151 MHz, $CDCl_3$) δ 174.4(0), 174.3(6), 171.8, 171.7, 153.5(2), 153.4(7), 141.8, 136.0, 135.0, 131.0(7), 130.9(7), 130.0(5), 129.7, 129.4(0), 129.3(7), 128.5, 66.3(2), 66.2(8), 55.0, 38.2, 37.7, 29.3, 8.4 ppm.

HRMS (ESI-pos) m/z: Calcd for $C_{14}H_{19}N_2O_5$ $[M+NH_4]^+$ 295.1289, Found 295.1276.

3-((*R*)-2-((1*r*,4*R*)-4-isopropylcyclohexanecarboxamido)-3-methoxy-3-oxopropyl)benzoic acid (3j-*meta*) and 4-((*R*)-2-((1*r*,4*R*)-4-isopropylcyclohexanecarboxamido)-3-methoxy-3-oxopropyl)benzoic acid (3j-*para*)

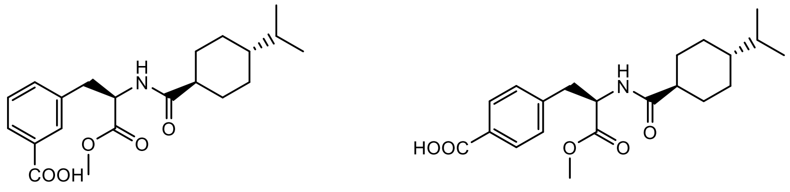

3j-*meta* 3j-*para*

Following the general procedure C and using (*R*)-methyl 2-((1s,4S)-4-isopropylcyclohexanecarboxamido)-3-phenylpropanoate (**1j**) (66.3 mg, 0.200 mmol) as substrate. ^{1}H-NMR yield and regioisomeric ratios of the intermediate olefinated compound **2j** were determined as 72%, m:p = 55:45 respectively. The assignment of **2j** from the crude reaction mixture was achieved by comparison with the report from our group.[30] From the subsequent step, the target compound **3j** was purified by silica gel column chromatography using gradient elution (pentane:EtOAc:HCOOH = 74:25:1 up to pentane:EtOAc:HCOOH = 69:30:1 as the eluent). Additionally, a mixed fraction containing **3j** was re-purified by silica gel column chromatography using pentane:EtOAc HCOOH = 79:20:1 as the eluent. The overall yield was calculated to be 44% with m:p = 52:48 obtaining **3j** as a light brown solid.

^{1}H NMR (600 MHz, CD_3OD) δ 7.95 – 7.93 (m, 2H^p), 7.90 – 7.87 (m, 2H^m), 7.45 (m, 1H^m), 7.41 – 7.38 (m, 1H^m), 7.32 – 7.30 (m, 2H^p), 4.86 (brs, 2H), 4.72 – 4.66 (m, 1H^m+1H^p), 3.71 – 3.70 (m, 3H^m+3H^p), 3.27 – 3.22 (m, 1H^m+1H^p), 3.05 – 3.00 (m, 1H^m+1H^p), 2.13 – 2.07 (m, 1H^m+1H^p), 1.82 – 1.71 (m, 3H^m+3H^p), 1.70 – 1.61 (m, 1H^m+1H^p), 1.42 – 1.23 (m, 3H^m+3H^p), 1.04 – 0.95 (m, 3H^m+3H^p), 0.87 – 0.85 (m, 6H^m+6H^p) ppm.

^{13}C NMR (151 MHz, CD_3OD) δ 179.1(4), 179.1(1), 173.4, 169.7, 144.0, 138.9, 134.9, 132.0, 131.7, 130.8, 130.4, 129.6, 129.2, 54.7, 54.5, 52.7, 46.2, 44.7, 38.3, 38.1, 34.1, 30.8(2), 30.7(8), 30.5(4), 30.4(9), 30.1(0), 30.0(8), 30.0(3), 20.1 ppm.

HRMS (ESI-pos) m/z: Calcd for $C_{21}H_{33}N_2O_5$ $[M+NH_4]^+$ 393.2384, Found 393.2365.

2'-fluoro-4'-(1-methoxy-1-oxopropan-2-yl)-[1,1'-biphenyl]-3-carboxylic acid (3k-β) and 2'-fluoro-4'-(1-methoxy-1-oxopropan-2-yl)-[1,1'-biphenyl]-4-carboxylic acid (3k-γ)

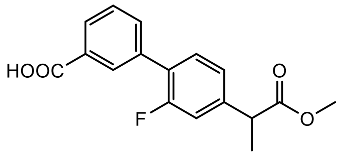

3k-β

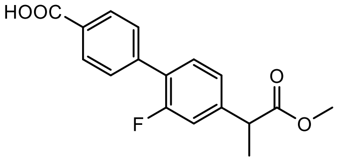

3k-γ

Following the general procedure C and using methyl 2-(2-fluoro-[1,1'-biphenyl]-4-yl)propanoate methyl 2-(1,3-dioxoisoindolin-2-yl)-3-phenylpropanoate (**1k**) (51.7 mg, 0.200 mmol) as substrate. ^{1}H-NMR yield and regioisomeric ratios of the intermediate olefinated compound **2k** were determined as 62%, β:γ = 62:38 respectively. From the subsequent step, the target compound **3k** was purified by silica gel column chromatography using gradient elution (pentane:EtOAc:HCOOH = 94:5:1 up to pentane:EtOAc:HCOOH = 89:10:1 as the eluent). Due to coelution of **3k** and **ML1** during column chromatography, all fractions containing the desired product were transferred into a flask and concentrated under reduced pressure. EtOAc (10 mL) was added to the flask and transferred into a separatory funnel. The organic phase was acidified with 1N HCl until reaching pH 2 and washed. This procedure was done three times to fully remove **ML1**. The organic phase was dried over $MgSO_4$, filtered, and concentrated under reduced pressure, obtaining **3k** as a white solid (22.9 mg, 61%, β:γ = 65:35).

^{1}H NMR (600 MHz, $CDCl_3$) δ 8.31 – 8.27 (m, 1H^{β}), 8.20 – 8.17 (m, 2H^{γ}), 8.13 – 8.11 (m, 1H^{β}), 7.81 – 7.78 (m, 1H^{β}), 7.67 – 7.63 (m, 2H^{γ}), 7.57 – 7.52 (m, 1H^{β}), 7.45 – 7.41 (m, 1H^{β}+1H^{γ}), 7.21 – 7.14 (m, 2H^{β}+2H^{γ}), 3.78 (q, *J* = 7.2 Hz, 1H^{β}+1H^{γ}), 3.71 (s, 3H^{β}+3H^{γ}), 1.55 (d, *J* = 7.2 Hz, 3H^{β}+3H^{γ}) ppm.

^{13}C NMR (151 MHz, $CDCl_3$) δ 174.5(3), 174.4(7), 172.0(5), 172.0(2), 159.8 (d, *J* = 248.6 Hz), 143.1 (d, *J* = 7.7 Hz), 142.7 (d, *J* = 7.6 Hz), 141.1, 136.1, 134.4, 130.9, 130.7, 130.5, 129.9, 129.5, 129.2, 128.8, 128.6, 126.8 (d, *J* = 13.3 Hz), 123.9, 115.8 – 115.4 (m), 52.4, 45.1, 18.5 ppm.

^{19}F NMR (471 MHz, $CDCl_3$) δ –116.9, –117.5 ppm.

HRMS (ESI-neg) m/z: Calcd for $[M-H]^-$ 301.0871, Found 301.0880.

3-(3-(1-methoxy-1-oxopropan-2-yl)benzoyl)benzoic acid (3l-β), 3-benzoyl-5-(1-methoxy-1-oxopropan-2-yl)benzoic acid (3l-β') and 4-(3-(1-methoxy-1-oxopropan-2-yl)benzoyl)benzoic acid (3l-γ):

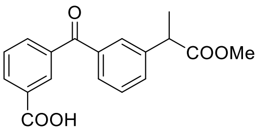

3l-β

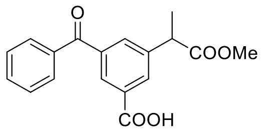

3l-β′

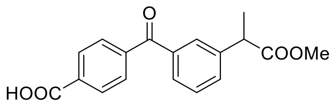

3l-γ

Following a modified general procedure C and using methyl 2-(3-benzoylphenyl)propanoate (53.6 mg, 0.200 mmol) as substrate. The ^{1}H-NMR yield of the intermediate olefinated compound **2l** was determined as 57%. The regioisomeric ratios could not be determined from the crude reaction mixture due to signal overlap. From the subsequent step using OsO_4 (126 µL, 4.00 µmol, 2.00 mol%, 2.50 (w/w)% in *t*-BuOH), Oxone® (738 mg, 1.20 mmol, 6.0 equiv), and DMF (1 mL, 0.2 M) at room temperature for 48 h, the target compound **3l** was purified by silica gel column chromatography using pentane:EtOAc:HCOOH = 85:15:1.0 as the eluent and obtained as a yellowish solid (15.5 mg, 43%, β:β':γ = 57:22:21).

^{1}H NMR (600 MHz, $CDCl_3$) δ 8.49 (t, *J* = 1.8 Hz, 1H$^{\beta}$), 8.38 (t, *J* = 1.6 Hz, 1H$^{\beta'}$), 8.33 (dd, *J* = 7.6, 1.2 Hz, 1H$^{\beta}$), 8.26 (t, *J* = 1.8 Hz, 1H$^{\beta'}$), 8.22 (d, *J* = 8.0 Hz, 2H$^{\gamma}$), 8.07 (dd, *J* = 7.7, 1.2 Hz, 1H$^{\beta}$), 8.02 (t, *J* = 1.7 Hz, 1H$^{\beta'}$), 7.86 (d, *J* = 8.0 Hz, 2H$^{\gamma}$), 7.85 – 7.79 (m, 2H$^{\beta'}$), 7.78 – 7.76 (m, 1H$^{\beta}$+1H$^{\gamma}$), 7.69 – 7.66 (m, 1H$^{\beta}$+1H$^{\gamma}$), 7.64 – 7.60 (m, 1H$^{\beta}$+1H$^{\beta'}$), 7.59 – 7.56 (m, 1H$^{\beta}$+1H$^{\gamma}$), 7.54 – 7.50 (m, 2H$^{\beta'}$), 7.50 – 7.44 (m, 1H$^{\beta}$+1H$^{\gamma}$), 3.90 (q, *J* = 7.2 Hz, 1H$^{\beta'}$), 3.86 – 3.77 (m, 1H$^{\beta}$+1H$^{\gamma}$), 3.70 (s, 3H$^{\beta'}$), 3.69 (s, 3H$^{\beta}$+3H$^{\gamma}$), 1.59 (d, *J* = 7.2 Hz, 3H$^{\beta'}$), 1.55 (d, *J* = 7.2 Hz, 3H$^{\beta}$+3H$^{\gamma}$) ppm.

^{13}C NMR (151 MHz, $CDCl_3$) δ 195.7, 195.4, 195.3, 174.5(2), 174.4(7), 174.0, 170.6, 170.4, 141.9, 141.6, 141.1, 138.4, 137.9, 137.2, 137.1, 136.8, 134.9, 134.1, 133.8, 133.0, 132.9, 132.4, 132.2, 132.0, 131.7, 130.5, 130.1(2), 130.0(9), 129.9, 129.8, 129.6, 129.3, 129.2, 129.1, 129.0, 128.8(4), 128.8(1), 128.7(8), 128.5(4), 128.4(9), 52.4, 52.2, 45.3, 45.1, 18.5(0), 18.4(7), 18.4 ppm.

HRMS (ESI-pos) m/z: Calcd for $C_{18}H_{17}O_5$ $[M+H]^+$ 313.1071, Found 313.1063.

IR (cm^{-1}): 3680, 2981, 2951, 1731, 1697, 1662, 1600, 1435.

Melting Point: >200 °C (decomp.)

3-(3-methoxy-3-oxopropyl)-5-methylbenzoic acid (3m):

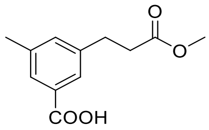

Following the general procedure C and using methyl 3-(m-tolyl)propanoate (35.6 mg, 0.200 mmol) as substrate. ^{1}H-NMR yield and regioisomeric ratios of the intermediate olefinated compound **2m** were determined as 82%, β:α = 97:3 respectively. From the subsequent step, the target compound **3m** was purified by silica gel column chromatography using pentane:EtOAc:HCOOH = 95:5:0.5 as the eluent and obtained as a colorless solid (27.3 mg, 75%, β as a single regioisomer).

^{1}H NMR (500 MHz, $CDCl_3$) δ 7.79 – 7.77 (m, 1H), 7.77 – 7.73 (m, 1H), 7.28 – 7.26 (m, 1H), 3.68 (s, 3H), 2.98 (t, *J* = 7.8 Hz, 2H), 2.66 (t, *J* = 7.4 Hz, 2H), 2.39 (s, 3H) ppm.

^{13}C NMR (126 MHz, $CDCl_3$) δ 173.1, 171.8, 140.9, 138.5, 134.7, 129.5, 128.8, 127.1, 51.7, 35.5, 30.6, 21.2 ppm.

HRMS (ESI-neg) m/z: Calcd for $C_{12}H_{13}O_4$ $[M-H]^-$ 221.0808, Found 221.0814.

IR (cm^{-1}): 2952, 2863, 1735, 1688, 1606, 1437, 1415, 1366, 1304.

Melting Point: 97 °C.

4-(methoxycarbonyl)-5,6,7,8-tetrahydronaphthalene-2-carboxylic acid (3n):

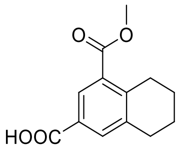

Following the general procedure C and using methyl 5,6,7,8-tetrahydronaphthalene-1-carboxylate (38.0 mg, 0.200 mmol) as substrate. ^{1}H-NMR yield and regioisomeric ratios of the intermediate olefinated compound **2n** were determined as 82%, m:(o+p) = 80:20 respectively. The assignments of **2n-*meta*** and mixture of **2n-*ortho* and 2n-*para*** from the crude reaction mixture were achieved by comparison with the report from our group.[30] From the subsequent step, the target compound **3n** was purified by silica gel column chromatography using pentane:EtOAc:HCOOH = 92:8:0.5 as the eluent and obtained as a colorless crystalline solid (25.3 mg, 66%, *meta* as a single regioisomer).

^{1}H NMR (600 MHz, $CDCl_3$) δ 8.37 (d, *J* = 1.8 Hz, 1H), 8.04 – 7.86 (m, 1H), 3.91 (s, 3H), 3.18 – 3.07 (m, 2H), 2.88 (t, *J* = 5.7 Hz, 2H), 1.92 – 1.72 (m, 4H) ppm.

^{13}C NMR (151 MHz, $CDCl_3$) δ 171.7, 167.8, 145.3, 139.2, 134.4, 130.8, 129.7, 126.2, 52.2, 30.3, 28.3, 22.8, 22.3 ppm.

HRMS (ESI-neg) m/z: Calcd for $C_{13}H_{13}O_4$ $[M-H]^-$ 233.0819, Found 233.0808.

IR (cm^{-1}): 2938, 2866, 1721, 1688, 1605, 1573, 1431, 1331.

Melting Point: 169 °C.

3-((2-isopropyl-5-methylphenoxy)methyl)-5-methylbenzoic acid (3o)

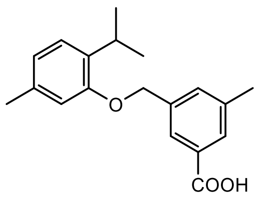

Following a modified literature procedure,[31] in a 10 mL round bottom flask, **4o** (16.4 mg, 0.060 mmol, 1 equiv) dissolved in acetone-water (5 mL, 4:1 v/v) was added $KMnO_4$ (20.9 mg, 0.13 mmol, 2.2 equiv). The reaction was stirred for 18 h at room temperature. Acetone was removed *in vacuo*, the reaction mixture was filtered and the filtrate was acidified with 1N HCl. Subsequently the aqueous phase was extracted with CH_2Cl_2 (3 x 20 mL). The combined organic phases were dried over $MgSO_4$, filtered, and concentrated under reduced pressure. The target compound **3o** was obtained as a colorless crystalline solid (16.5 mg, 47%, β as a single regioisomer).

^{1}H NMR (600 MHz, $CDCl_3$) δ 8.00 – 7.97 (m, 1H), 7.90 – 7.87 (m, 1H), 7.53 – 7.51 (m, 1H), 7.13 (d, J = 7.7 Hz, 1H), 6.79 (d, 1H), 6.74 – 6.73 (m, 1H), 5.09 – 5.08 (m, 2H), 3.37 (hept, J = 6.9 Hz, 1H), 2.45 (s, 3H), 2.32 (s, 3H), 1.23 (d, J = 6.9 Hz, 6H) ppm.

^{13}C NMR (151 MHz, $CDCl_3$) δ 170.6, 155.8, 138.9, 138.3, 136.5, 134.6, 133.3, 130.3, 129.4, 126.2(2), 126.1(9), 121.8, 112.9, 69.6, 26.7, 23.0, 21.5(1), 21.4(9) ppm.

HRMS (ESI-neg) m/z: Calcd for $C_{19}H_{22}O_3$ $[M-H]^-$ 297.1485, Found 297.1497.

IR (cm^{-1}): 2954, 2917, 1689, 1240.

Melting point: sublimated.

5-(methoxycarbonyl)-6-methyl-4'-(trifluoromethoxy)-[1,1'-biphenyl]-3-carboxylic acid (3p)

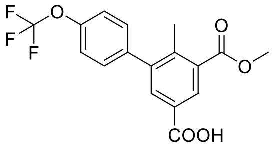

Following the general procedure C and using methyl 2-methyl-4'-(trifluoromethoxy)-[1,1'-biphenyl]-3-carboxylate (62.0 mg, 0.200 mmol) as substrate. ^{1}H-NMR yield and regioisomeric ratios of the intermediate olefinated compound **2p** were determined as 65%, β:β':others = 80:12:8 respectively. The assignment of **2p** from the crude reaction mixture was achieved by comparison with the report from our group.[30] From the subsequent step, the target compound **3p** was purified by silica gel column chromatography using pentane:EtOAc:HCOOH = 95:5:0.5 as the eluent and obtained as a colorless solid (29.0 mg, 63%, β as a single regioisomer).

^{1}H NMR (500 MHz, $CDCl_3$) δ 8.56 (d, *J* = 1.5 Hz, 1H), 8.07 (d, *J* = 1.5 Hz, 1H), 7.40 – 7.29 (m, 4H), 3.96 (s, 3H), 2.49 (s, 3H) ppm.

^{13}C NMR (126 MHz, $CDCl_3$) δ 170.8, 167.6, 148.8, 143.3, 143.0, 138.9, 134.2, 132.0, 131.2, 130.7, 126.6, 120.9, 52.4, 19.0 ppm.

^{19}F NMR (471 MHz, $CDCl_3$) δ –58.3 ppm.

HRMS (ESI-neg) m/z: Calcd for $C_{17}H_{12}O_5F_3$ $[M-H]^-$ 353.0631, Found 353.0642.

IR (cm^{-1}): 3680, 2967, 2844, 1736, 1700, 1432.

Melting Point: 213 °C.

4-((5-methoxy-4,4-dimethyl-5-oxopentyl)oxy)-2,5-dimethylbenzoic acid (3q):

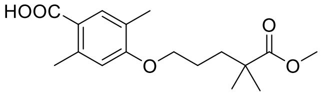

Following a modified literature procedure,[31] a sample of **4q** (7.50 mg, 0.0255 mmol, 1 equiv) obtained through the procedure described in the next section, was dissolved in acetone-water (2.5 mL, 4:1 v/v) and $KMnO_4$ was added (56.8 mg, 0.0360 mmol, 1.42 equiv). The reaction was stirred for 7 h at room temperature. Acetone was removed under reduced pressure, the reaction mixture was filtered, and the filtrate was acidified with 1N HCl. The aqueous phase was extracted with CH_2Cl_2 (5 x 20 mL) and the combined organic phases were dried over $MgSO_4$, filtered, and concentrated under reduced pressure. The target compound **3q** was obtained as a light brown solid in 59% yield (4.6 mg, β as a single regioisomer). This corresponds to an overall yield of 41% from olefin to carboxylic acid when considering the yield of the aldehyde synthesis described in the following section.

^{1}H NMR (600 MHz, $CDCl_3$) δ 7.90 – 7.85 (m, 1H), 6.63 – 6.60 (m, 1H), 3.99 (t, *J* = 6.0 Hz, 2H), 3.67 (s, 3H), 2.62 (s, 3H), 2.20 (s, 3H), 1.80 – 1.69 (m, 4H), 1.23 (s, 6H) ppm.

^{13}C NMR (151 MHz, $CDCl_3$) δ 178.4, 172.3, 160.8, 142.0, 134.3, 124.2, 119.5, 113.8, 68.2, 51.9, 42.2, 37.1, 25.3, 25.1, 22.7, 15.7 ppm.

HRMS (ESI-neg) m/z: Calcd for $C_{17}H_{23}O_5$ $[M-H]^-$ 307.1540, Found 307.1544.

IR (cm^{-1}): 2953, 1841, 1631, 1538, 1464.

4.4 Characterization of Aldehydes

3-(*tert*-butyl)benzaldehyde (4a-*meta*) and 4-(*tert*-butyl)benzaldehyde (4a-*para*)

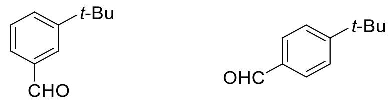

4a-*meta* **4a-*para***

Following a slightly modified general procedure D, using $Pd(OAc)_2$ (4.6 mg, 20 μmol, 10 mol%), 2-acetamido-N-((perfluorophenyl)sulfonyl)acetamide (**BL1**) (20.8 mg, 60.0 μmol, 30 mol%), 2,3,5,6-tetramethylpyrazine (**ML9**) (5.5 mg, 40 μmol, 20 mol%), AgOAc (100.2 mg, 0.6000 mmol, 3.0 equiv), HFIP (2.4 mL), and tert-butylbenzene (26.8 mg, 0.200 mmol) as substrate at 70°C for 48 h. ^{1}H-NMR yield and regioisomeric ratios of the intermediate olefinated compound **2a** were determined as 70%, m:p = 57:43 respectively (additionally 17% of di-olefinated product were observed). The assignment of **2a-*meta*** and **2a-*para*** from the crude reaction mixture were achieved by comparison with the literature.[18] From the subsequent step, the target compound **4a** was purified by silica gel column chromatography using gradient elution (pentane:Et_2O= 100:0 up to pentane:Et_2O= 99:1 as the eluent) and obtained as a colorless oil (12.9 mg, 57**%,** m:p = 77:23).[3]The spectral data of **4a** were in accordance to the ones reported in literature.[32, 33]

^{1}H NMR (600 MHz, CDCl$_3$) δ 10.02 (s, 1H^m), 9.98 (s, 1H^p), 7.93 – 7.90 (m, 1H^m), 7.84 – 7.80 (m, 2H^p), 7.71 – 7.66 (m, 2H^m), 7.57 – 7.54 (m, 2H^p), 7.49 – 7.45 (m, 1H^m), 1.37 (s, 9H^m), 1.36 (s, 9H^p) ppm.

^{13}C NMR (151 MHz, CDCl$_3$) δ 193.0, 192.2, 158.6, 152.4, 136.5, 134.2, 131.9, 129.8, 128.9, 127.6, 126.5, 126.1, 35.5, 35.0, 31.3, 31.2 ppm.

[3] Since the compound is volatile, solvent should be carefully removed by rotary evaporation.

2,4-dimethylbenzaldehyde (4b-α) and 3,5-dimethylbenzaldehyde (4b-β):

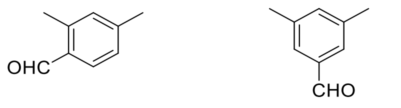

4b-α 4b-β

Following the general procedure D and using *m*-xylene (21.2 mg, 0.200 mmol) as substrate. ^{1}H-NMR yield and regioisomeric ratios of the intermediate olefinated compound **2b** were determined as 81%, β:α = 90:10 respectively. The assignment of **2b-α** and **2b-β** from the crude reaction mixture were achieved by comparison with the literature.[21] From the subsequent step, the target compound **4b** was purified by silica gel column chromatography using pentane:EtOAc = 98:2 as the eluent and obtained as a colorless oil (17.4 mg containing 16.5 mg, 76% of **4b**, β:α = 96:4). Since the compound is volatile, the residual solvents could not be removed completely by rotary evaporation to avoid complete loss of **4b**. The spectral data of **4b** were in accordance to the ones reported in literature.[34]

^{1}H NMR (500 MHz, $CDCl_3$) δ 10.21 (s, 1H^α), 9.95 (s, 1H^β), 7.69 (d, *J* = 7.7 Hz, 1H^α), 7.52 – 7.46 (m, 2H^β), 7.28 – 7.26 (m, 1H^β), 7.16 (d, *J* = 7.8 Hz, 1H^α), 7.07 (s, 1H^α), 2.64 (s, 3H^α), 2.39 (s, 6H^β), 2.37 (s, 3H^α) ppm.

^{13}C NMR (126 MHz, $CDCl_3$) δ 193.2, 139.2, 137.0, 136.6, 128.0, 21.5 ppm.

HRMS (ESI-pos) m/z: Calcd for $C_9H_{11}O$ [M+H]$^+$ 135.0804, Found 135.0803.

2-methoxy-4-methylbenzaldehyde (4c-α) and 3-methoxy-5-methylbenzaldehyde (4c-β):

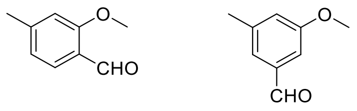

4c-α **4c-β**

Following the general procedure D and using 1-methoxy-3-methylbenzene (24.4 mg, 0.200 mmol) as substrate. ^{1}H-NMR yield and regioisomeric ratios of the intermediate olefinated compound **2c** were determined as 72%, β:α = 80:20 respectively. The assignment of **2c-α** and **2c-β** from the crude reaction mixture were achieved by comparison with the literature.[23, 24] In the subsequent step, the product **4c** was purified by silica gel column chromatography using pentane:EtOAc = 98:2 as the eluent. The target compound **4c-β** was obtained as a colorless oil (14.3 mg, 66%). Additionally, a fraction containing the minor isomer **4c-α** as an inseparable mixture with grease and additional **4c-β** was isolated in form of a colorless oil (6.1 mg containing 3.7 mg, 17% of **4c-α** and 0.4 mg, 2% of **4c-β**). Accordingly, the overall yield was calculated to be 85% with β:α = 80:20. The spectral data of **4c** were in accordance to the ones reported in literature.[35, 36]

Characterization of the β-isomer:

^{1}H NMR (500 MHz, $CDCl_3$) δ 9.93 (s, 1H), 7.30 – 7.27 (m, 1H), 7.22 – 7.18 (m, 1H), 7.02 – 6.96 (m, 1H), 3.85 (s, 3H), 2.40 (s, 3H) ppm.

^{13}C NMR (126 MHz, $CDCl_3$) δ 192.5, 160.3, 140.5, 137.9, 124.6, 122.3, 109.6, 55.6, 21.4 ppm.

Characterization of the α-isomer from the mixture:

^{1}H NMR (500 MHz, $CDCl_3$) δ 10.40 (s, 1H), 7.73 (d, J = 7.8 Hz, 1H), 6.88 – 6.81 (m, 1H), 6.78 (s, 1H), 3.92 (s, 3H), 2.41 (s, 3H) ppm.

^{13}C NMR (126 MHz, $CDCl_3$) δ 189.5, 161.9, 147.4, 128.6, 122.7, 121.7, 112.2, 55.5, 22.3 ppm.

HRMS (ESI-pos) m/z: Calcd for $C_9H_{11}O_2$ $[M+H]^+$ 151.0750, Found 151.0749.

3-methyl-5-((triisopropylsilyl)oxy)benzaldehyde (4d)

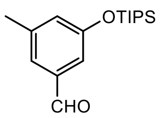

Following a slightly modified general procedure D, using $Pd(OAc)_2$ (4.6 mg, 20 μmol, 10 mol%), 2-acetamido-N-((perfluorophenyl)sulfonyl)acetamide (**BL1**) (20.8 mg, 60.0 μmol, 30 mol%), 2,3,5,6-tetramethylpyrazine (**ML9**) (5.5 mg, 40 μmol, 20 mol%), AgOAc (100.2 mg, 0.6000 mmol, 3.0 equiv), HFIP (2.4 mL), and triisopropyl(m-tolyloxy)silane (**1d**) (53.1 mg, 0.201 mmol) as substrate at 70°C for 48 h. ^{1}H-NMR yield and regioisomeric ratios of the intermediate olefinated compound **2d** were determined as 80%, β as a single regioisomer respectively. From the subsequent step, the target compound **4d** was purified by silica gel column chromatography using gradient elution (pentane:Et_2O = 100:0 up to pentane:Et_2O = 95:5 as the eluent) and obtained as a light yellow oil (24.3 mg, 52%, β as a single regioisomer). The spectral data of **4d** were in accordance to the ones reported in literature.[37]

^{1}H NMR (600 MHz, $CDCl_3$) δ 9.90 (s, 1H), 7.27 – 7.26 (m, 1H), 7.17 – 7.15 (m, 1H), 6.97 – 6.95 (m, 1H), 2.37 (brs, 3H), 1.28 (sept, *J* = 7.7 Hz, 3H), 1.10 (d, *J* = 7.6 Hz, 18H) ppm.

^{13}C NMR (151 MHz, $CDCl_3$) δ 192.5, 156.8, 140.4, 137.9, 127.2, 124.1, 117.0, 21.3, 18.0, 12.8 ppm.

HRMS (ESI-pos) m/z: Calcd for $C_{17}H_{32}NO_2Si$ $[M+NH_4]^+$ 310.2197, Found 310.2199.

4-acetyl-3,5-dimethylbenzaldehyde (4e):

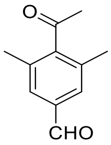

Following the general procedure D and using 1-(2,6-dimethylphenyl)ethanone (29.6 mg, 0.200 mmol) as substrate. ^{1}H-NMR yield and regioisomeric ratios of the intermediate olefinated compound **2e** were determined as 73%, β as a single regioisomer respectively. From the subsequent step, the target compound **4e** was purified by silica gel column chromatography using pentane:EtOAc = 97:3 as the eluent and obtained as a colorless solid (17.7 mg, 69%, β as a single regioisomer). The spectral data of **4e** were in accordance to the ones reported in literature.[38]

^{1}H NMR (500 MHz, CDCl$_3$) δ 9.96 (s, 1H), 7.54 (t, *J* = 0.7 Hz, 2H), 2.50 (s, 3H), 2.32 (s, 6H) ppm.

^{13}C NMR (126 MHz, CDCl$_3$) δ 207.1, 192.0, 147.9, 136.2, 133.4, 129.2, 31.7, 19.0 ppm.

HRMS (ESI-pos) m/z: Calcd for $C_{11}H_{13}O_2$ $[M+H]^+$ 177.0910, Found 177.0910.

2-chloro-5-methylbenzaldehyde (4f-β) and 2-chloro-5-methylisophthalaldehyde (4f-di):

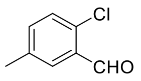

4f-β

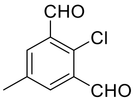

4f-di

Following a modified general procedure D, using $Pd(OAc)_2$ (4.6 mg, 20 µmol, 10 mol%), 2-acetamido-*N*-((perfluorophenyl)sulfonyl)acetamide (**BL1**) (20.8 mg, 60.0 µmol, 30 mol%), 2,3,5,6-tetramethylpyrazine (**ML9**) (5.5 mg, 40 µmol, 20 mol%), AgOAc (100.2 mg, 0.6000 mmol, 3.0 equiv), HFIP (2.4 mL), and 1-chloro-4-methylbenzene (25.3 mg, 0.200 mmol) as substrate at 70°C for 48 h. ^{1}H-NMR yield and regioisomeric ratios of the intermediate olefinated compound **2f** were determined as 81%, β as a single regioisomer respectively (additionally 10% of di-olefinated product were observed). The assignment of **2f** from the crude reaction mixture was achieved by comparison with the report from our group.[18] From the subsequent step, the target compound **4f-β** was purified by silica gel column chromatography using pentane:Et_2O = 99:1 as the eluent and obtained as a colorless oil (18.4 mg, 73%, β as a single regioisomer). Additionally, the di-product **4f-di** was purified by silica gel column chromatography using pentane:Et_2O = 90:10 as the eluent and obtained as colorless solid (3,1 mg, 85%). The spectral data of **4f** were in accordance to the ones reported in literature.[39]

Characterization of the β-isomer:

^{1}H NMR (600 MHz, $CDCl_3$) δ 10.45 (s, 1H), 7.76 – 7.68 (m, 1H), 7.33 (d, *J* = 1.2 Hz, 2H), 2.38 (s, 3H) ppm.

^{13}C NMR (151 MHz, $CDCl_3$) δ 190.4, 137.8, 136.4, 135.4, 132.4, 130.7, 130.0, 21.1 ppm.

HRMS (ESI-pos) m/z: Calcd for C_8H_8OCl $[M+H]^+$ 155.0258, Found 155.0257.

Characterization of the di-product:

^{1}H NMR (500 MHz, $CDCl_3$) δ 10.57 (s, 2H), 7.97 (d, *J* = 0.7 Hz, 2H), 2.44 (s, 3H) ppm.

^{13}C NMR (126 MHz, $CDCl_3$) δ 189.1, 138.5, 138.4, 135.6, 133.5, 21.1 ppm.

HRMS (ESI-pos) m/z: Calcd for $C_9H_8O_2Cl$ $[M+H]^+$ 183.0207, Found 183.0206.

2-chloro-3-methoxy-5-methylbenzaldehyde (4g):

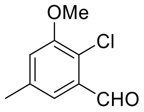

Following the general procedure D and using 1-chloro-2-methoxy-4-methylbenzene (31.3 mg, 0.200 mmol) as substrate. ^{1}H-NMR yield and regioisomeric ratios of the intermediate olefinated compound **2g** were determined as 64%, β as a single regioisomer respectively. From the subsequent step, the target compound **4g** was purified by silica gel column chromatography using pentane:EtOAc = 97:3 as the eluent and obtained as a colorless solid (17.8 mg, 75%, β as a single regioisomer). The spectral data of **4g** were in accordance to the ones reported in literature.[40]

^{1}H NMR (500 MHz, $CDCl_3$) δ 10.50 (s, 1H), 7.41 – 7.29 (m, 1H), 6.98 (d, *J* = 2.0 Hz, 1H), 3.94 (s, 3H), 2.39 (s, 3H) ppm.

^{13}C NMR (126 MHz, $CDCl_3$) δ 190.3, 155.2, 137.9, 133.1, 123.9, 121.0, 118.1, 56.5, 21.3 ppm.

HRMS (ESI-pos) m/z: Calcd for $C_9H_{10}O_2Cl$ $[M+H]^+$ 185.0364, Found 185.0364.

(*S*)-methyl 2-(1,3-dioxoisoindolin-2-yl)-3-(3-formylphenyl)propanoate (4h-*meta*) and (*S*)-methyl 2-(1,3-dioxoisoindolin-2-yl)-3-(4-formylphenyl)propanoate (4h-*para*)

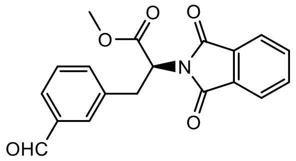

4h-*meta*

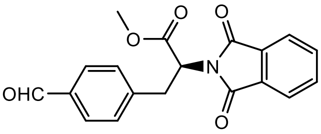

4h-*para*

Following the general procedure D and using (*S*)-methyl 2-(1,3-dioxoisoindolin-2-yl)-3-phenylpropanoate (**1h**) (61.9 mg, 0.200 mmol) as substrate. ^{1}H-NMR yield and regioisomeric ratios of the intermediate olefinated compound **2h** were determined as 71%, m:p = 57:43 respectively. The assignment of **2h** from the crude reaction mixture was achieved by comparison with the literature.[28] From the subsequent step, the target compound **4h** was purified by silica gel column chromatography using gradient elution (pentane:EtOAc = 85:15 up to pentane: EtOAc = 80:20 as the eluent) followed by a repurification by silica gel column chromatography using gradient elution (cyclohexane:EtOAc= 90:10 up to cyclohexane:EtOAc = 85:15 as the eluent), obtaining **4h** as a colorless oil (44.8 mg, 94%, m:p= 53:47).

^{1}H NMR (500 MHz, CDCl$_3$) δ 9.88(5) (s, 1H^p), 9.87(5) (s, 1H^m), 7.80 – 7.75 (m, 2H^m+2H^p), 7.72 – 7.65 (m, 4H^m+4H^p), 7.45 (ddd, *J* = 9.2, 3.2, 1.6 Hz, 1H^m), 7.39 – 7.32 (m, 1H^m+2H^p), 5.21 – 5.15 (m, 1H^m+1H^p), 3.77 (brs, 3H^m+3H^p), 3.71 – 3.57 (m, 2H^m+2H^p) ppm.

^{13}C NMR (126 MHz, CDCl$_3$) δ 192.1, 191.9, 169.1, 169.0, 167.5, 167.4, 144.1, 138.1, 136.8, 135.3, 135.0, 134.4, 131.5(2), 131.4(9), 130.4, 130.1, 129.7, 129.4, 128.3, 123.7, 53.2, 53.1, 53.0, 52.8, 35.0, 34.6 ppm.

HRMS (ESI-pos) m/z: Calcd for $C_{19}H_{16}NO_5$ $[M+H]^+$ 338.1023, Found 338.1018.

(*R*)-3-((2-oxo-3-propionyloxazolidin-4-yl)methyl)benzaldehyde (4i-*meta*) and (*R*)-4-((2-oxo-3-propionyloxazolidin-4-yl)methyl)benzaldehyde (4i-*para*)

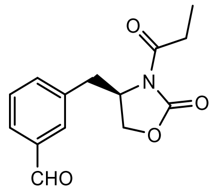

4i-*meta*

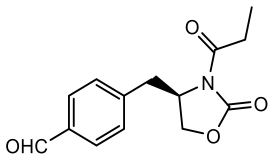

4i-*para*

Following the general procedure D and using (*R*)-4-benzyl-3-propionyloxazolidin-2-one (**1i**) (46.7 mg, 0.200 mmol) as substrate. ^{1}H-NMR yield and regioisomeric ratios of the intermediate olefinated compound **2i** were determined as 79%, m:p = 52:48 respectively. The assignment of **2i** from the crude reaction mixture was achieved by comparison with the report from our group.[30] From the subsequent step, the target compound **4i** was purified by silica gel column chromatography using gradient elution (pentane:Et_2O = 50:50 up to pentane:Et_2O= 35:65 as the eluent) and obtained as a colorless oil along with the presence of cyclohexane (32.6 mg containing 7% of cyclohexane corresponding to 30.3 mg of pure product). Accordingly, the yield was calculated to be 73%, m:p = 51:49.

^{1}H NMR (600 MHz, $CDCl_3$) δ 9.99 (s, 1H^m), 9.98 (s, 1H^p), 7.86 – 7.83 (m, 2H^p), 7.78 (ddd, *J* = 9.0, 3.1, 1.6 Hz, 1H^m), 7.72 (brs, 1H^m), 7.53 – 7.50 (m, 1H^m), 7.48 (ddd, *J* = 9.0, 3.1, 1.4 Hz, 1H^m), 7.39 – 7.37 (m, 2H^p), 4.73 – 4.68 (m, 1H^m+1H^p), 4.26 – 4.22 (m, 1H^m+1H^p), 4.15 – 4.12 (m, 1H^m+1H^p), 3.37 – 3.33 (m, 1H^m+1H^p), 3.01 – 2.87 (m, 3H^m+3H^p), 1.20 – 1.17 (m, 3H^m+3H^p) ppm.

^{13}C NMR (151 MHz, $CDCl_3$) δ 192.0, 191.7, 174.2, 153.4, 153.3, 142.5, 137.0, 136.7, 135.7, 135.5, 130.4, 130.2, 130.0, 129.8, 129.3, 66.2, 54.9(5), 54.9(0), 38.3, 37.8, 29.3, 8.4 ppm.

HRMS (ESI-pos) m/z: Calcd for $C_{14}H_{19}N_2O4$ $[M+NH_4]^+$ 279.1339, Found 279.1333.

(*R*)-methyl 3-(3-formylphenyl)-2-((1r,4R)-4-isopropylcyclohexanecarboxamido) propanoate (4j-*meta*) and (R)-methyl 3-(4-formylphenyl)-2-((1r,4R)-4-isopropylcyclohexanecarboxamido)propanoate (4j-*para*)

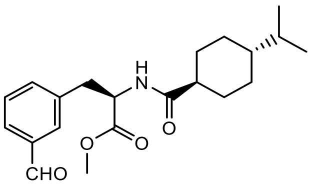

4j-*meta*

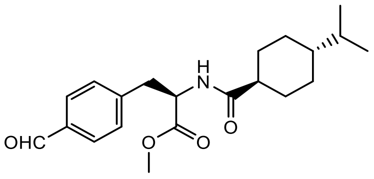

4j-*para*

Following the general procedure D and using (*R*)-methyl 2-((1s,4S)-4-isopropylcyclohexanecarboxamido)-3-phenylpropanoate (**1j**) (66.3 mg, 0.200 mmol) as substrate. ^{1}H-NMR yield and regioisomeric ratios of the intermediate olefinated compound **2j** were determined as 73%, m:p = 55:45 respectively. The assignment of **2j** from the crude reaction mixture was achieved by comparison with the report from our group.[30] From the subsequent step, the target compound **4j** was purified by silica gel column chromatography using gradient elution (pentane:Et_2O = 55:45 up to pentane:Et_2O = 45:55 as the eluent) and obtained as a colorless oil(31.6 mg, 60 %, m:p= 51:49).

^{1}H NMR (600 MHz, $CDCl_3$) δ 9.97 (brs, $1H^{m}+1H^{p}$), 7.81 – 7.79 (m, $2H^{p}$), 7.75 (ddd, *J* = 9.0, 2.8, 1.4 Hz, $1H^{m}$), 7.60 (brs, $1H^{m}$), 7.47 – 7.44 (m, $1H^{m}$), 7.37 (ddd, *J* = 9.0, 2.8, 1.4 Hz, $1H^{m}$), 7.26 – 7.24 (m, $2H^{p}$), 5.97 – 5.95 (m, $1H^{m}+1H^{p}$), 4.94 – 4.89 (m, $1H^{m}+1H^{p}$), 3.74 (m, $3H^{m}+3H^{p}$), 3.30 – 3.25 (m, $1H^{m}+1H^{p}$), 3.17 – 3.11 (m, $1H^{m}+1H^{p}$), 2.04 – 1.98 (m, $1H^{m}+1H^{p}$), 1.89 – 1.81 (m, $2H^{m}+2H^{p}$), 1.79 – 1.74 (m, $2H^{m}+2H^{p}$), 1.41 – 1.34 (m, $3H^{m}+3H^{p}$), 1.07 – 1.00 (m, $1H^{m}+1H^{p}$), 1.00 – 0.91 (m, $2H^{m}+2H^{p}$), 0.85 – 0.82 (m, $6H^{m}+6H^{p}$) ppm.

^{13}C NMR (151 MHz, $CDCl_3$) δ 192.2, 192.0, 175.8, 175.8, 172.1, 172.0, 143.5, 137.4, 136.7, 135.5(4), 135.4(5), 130.3, 130.2, 130.0, 129.3, 129.0, 52.8, 52.7, 52.6, 45.6, 43.3, 38.2, 37.8, 32.9, 30.0, 29.9, 29.6(0), 29.5(8), 29.0(7), 28.9(8), 19.8.

HRMS (ESI-pos) m/z: Calcd for $C_{21}H_{33}N_2O_4$ $[M+NH_4]^+$ 377.2435, Found 377.2426.

Methyl 2-(2-fluoro-3'-formyl-[1,1'-biphenyl]-4-yl)propanoate (4k-β) and Methyl 2-(2-fluoro-4'-formyl-[1,1'-biphenyl]-4-yl)propanoate (4k-γ)

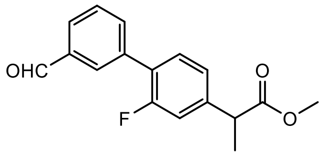

4k-β

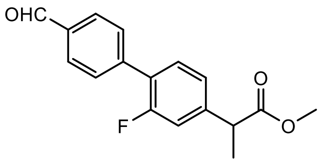

4k-γ

Following the general procedure D and using methyl 2-(2-fluoro-[1,1'-biphenyl]-4-yl)propanoate (51.7 mg, 0.200 mmol) as substrate. ^{1}H-NMR yield and regioisomeric ratios of the intermediate olefinated compound **2k** were determined as 59%, β:γ = 60:40 From the subsequent step, the target compound **4k** was purified by silica gel column chromatography using gradient elution (cyclohexane:Et_2O = 100:0 up to cyclohexane:Et_2O = 80:20 as the eluent). In one fraction pure β-isomer was obtained as a colorless oil (11.2 mg, 33%, β as a single regioisomer) and in a second fraction a mixture of β- and γ-isomer was obtained as a colorless oil (10.1 mg, 30%, β:γ =35:65). The overall yield was calculated to be 63%, β:γ= 69:31.

Characterization of the β-isomer:

^{1}H NMR (600 MHz, $CDCl_3$) δ 10.07 (s, 1H), 8.05 – 8.03 (m, 1H), 7.90 – 7.87 (m, 1H), 7.82 – 7.80 (m, 1H), 7.63 – 7.59 (m, 1H), 7.44 – 7.41 (m, 1H), 7.20 – 7.18 (m, 1H), 7.17 – 7.14 (m, 1H), 3.78 (q, *J* = 7.2 Hz, 1H), 3.71 (s, 3H), 1.55 (d, *J* = 7.2 Hz, 3H) ppm.

^{13}C NMR (151 MHz, $CDCl_3$) δ 192.3, 174.4, 159.8 (d, *J* = 249.0 Hz), 142.9 (d, *J* = 7.8 Hz), 136.8, 136.6, 135.0, 130.8 (d, *J* = 3.7 Hz), 130.4, 129.3, 128.9, 126.5 (d, *J* = 13.3 Hz), 124.0, 115.6 (d, *J* = 23.6 Hz), 52.4, 45.1, 18.6 ppm.

^{19}F NMR (471 MHz, $CDCl_3$) δ –117.5 ppm.

Characterization of the β- and γ-isomer mixture:

^{1}H NMR (600 MHz, $CDCl_3$) δ 10.07 (s, $1H^{\beta}$), 10.06 (s, $1H^{\gamma}$), 8.05 – 8.03 (m, $1H^{\beta}$), 7.96 – 7.94 (m, $2H^{\gamma}$), 7.90 – 7.87 (m, $1H^{\beta}$), 7.82 – 7.80 (m, $1H^{\beta}$), 7.72 – 7.69 (m, $2H^{\gamma}$), 7.63 – 7.59 (m, $1H^{\beta}$), 7.44 – 7.41 (m, $1H^{\beta}+1H^{\gamma}$), 7.20 – 7.18 (m, $1H^{\beta}+1H^{\gamma}$), 7.17 – 7.14 (m, $1H^{\beta}+1H^{\gamma}$), 3.78 (q, *J* = 7.2 Hz, $1H^{\beta}+1H^{\gamma}$), 3.71 (s, $3H^{\beta}+3H^{\gamma}$), 1.55 (d, *J* = 7.2 Hz, $3H^{\beta}+3H^{\gamma}$) ppm.

^{13}C NMR (151 MHz, $CDCl_3$) δ 192.3, 192.0, 174.4(3), 174.3(8), 159.8 (d, *J* = 250.1 Hz), 143.3 (d, *J* = 7.7 Hz), 142.9 (d, *J* = 7.9 Hz), 141.8, 136.8, 136.6, 135.5, 135.0, 130.8, 130.4, 130.0, 129.7, 129.3, 128.9, 126.6 (d, *J* = 13.3 Hz), 126.5 (d), 124.0, 115.9 – 115.4 (m), 52.4, 45.1, 18.5 ppm.

^{19}F NMR (471 MHz, $CDCl_3$) δ –116.8, –117.5 ppm.

HRMS (ESI-pos) m/z: Calcd for $C_{17}H_{19}FNO_3$ $[M+NH_4]^+$ 304.1344, Found 304.1335.

Methyl 2-(3-(3-formylbenzoyl)phenyl)propanoate (4l-β), methyl 2-(3-benzoyl-5-formylphenyl)propanoate (4l-β') and methyl 2-(3-(4-formylbenzoyl)phenyl)propanoate (4l-γ):

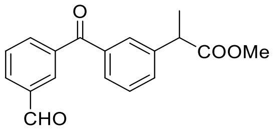

4l-β

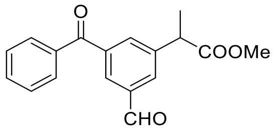

4l-β′

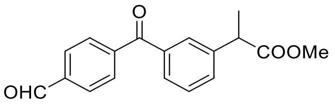

4l-γ

Following the general procedure D and using methyl 2-(3-benzoylphenyl)propanoate (53.6 mg, 0.200 mmol) as substrate. The ^{1}H-NMR yield of the intermediate olefinated compound **2l** was determined as 57%. The regioisomeric ratios from the crude reaction mixture could not b e determined due to signal overlap. From the subsequent step, the target compound **4l** was purified by silica gel column chromatography using pentane:EtOAc = 90:10 as the eluent and obtained as a yellowish oil (16.2 mg containing 14.9 mg, 44% of 4l, β:β':γ = 57:22:21 and 1.3 mg of silicone grease).

^{1}H NMR (600 MHz, $CDCl_3$) δ 10.14 (s, 1H$^{\gamma}$), 10.10 (s, 1H$^{\beta}$), 10.08 (s, 1H$^{\beta'}$), 8.31 – 8.23 (m, 1H$^{\beta}$), 8.16 (t, J = 1.6 Hz, 1H$^{\beta'}$), 8.14 – 8.11 (m, 1H$^{\beta}$), 8.10 – 8.07 (m, 1H$^{\beta}$), 8.07 – 8.05 (m, 1H$^{\beta'}$), 8.04 – 8.02 (m, 1H$^{\beta'}$), 8.02 – 8.00 (m, 2H$^{\gamma}$), 7.95 – 7.90 (m, 2H$^{\gamma}$), 7.84 – 7.78 (m, 2H$^{\beta'}$), 7.78 – 7.74 (m, 1H$^{\beta}$+1H$^{\gamma}$), 7.72 – 7.69 (m, 1H$^{\beta}$), 7.69 – 7.66 (m, 1H$^{\beta}$+1H$^{\gamma}$), 7.66 – 7.62 (m, 1H$^{\beta'}$), 7.60 – 7.56 (m, 1H$^{\beta}$+1H$^{\gamma}$), 7.55 – 7.50 (m, 2H$^{\beta'}$), 7.50 – 7.45 (m, 1H$^{\beta}$+1H$^{\gamma}$), 3.91 (q, J = 7.2 Hz, 1H$^{\beta'}$), 3.87 – 3.78 (m, 1H$^{\beta}$+1H$^{\gamma}$), 3.70 (s, 3H$^{\beta'}$), 3.69 – 3.68 (m, 3H$^{\beta}$+3H$^{\gamma}$), 1.60 (d, J = 7.2 Hz, 3H$^{\beta'}$), 1.57 – 1.52 (m, 3H$^{\beta}$+3H$^{\gamma}$) ppm.

^{13}C NMR (151 MHz, $CDCl_3$) δ 195.9, 195.6, 192.0, 191.7, 191.6, 174.8, 174.3, 142.8, 142.5, 141.5, 139.2, 138.9, 138.8, 137.5, 137.4, 137.1, 137.0, 136.7, 135.8, 135.1, 133.4, 133.2, 132.6, 132.5, 132.2, 131.7, 130.7, 130.6, 130.4, 129.9, 129.7, 129.6, 129.5(4), 129.4(6), 129.3, 129.2(0), 129.1(7), 129.0, 128.9, 52.8, 52.6, 45.6, 45.4, 18.9, 18.8 ppm.

HRMS (ESI-pos) m/z: Calcd for $C_{18}H_{17}O_4$ $[M+H]^+$ 297.1121, Found 297.1117.

IR (cm^{-1}): 2981, 295, 2845, 1733, 1701, 1660, 1597, 1434.

Methyl 3-(3-formyl-5-methylphenyl)propanoate (4m):

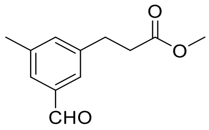

Following the general procedure D and using Methyl 3-(m-tolyl)propanoate (35.6 mg, 0.200 mmol) as substrate. ^{1}H-NMR yield and regioisomeric ratios of the intermediate olefinated compound **2m** were determined as 82%, β:α = 97:3 respectively. From the subsequent step, the target compound **4m** was purified by silica gel column chromatography using pentane:EtOAc = 96:4 as the eluent and obtained as an orange oil (28.4 mg, 84%, β as a single regioisomer).

^{1}H NMR (500 MHz, $CDCl_3$) δ 9.96 (s, 1H), 7.69 – 7.40 (m, 2H), 7.32 – 7.28 (m, 1H), 3.67 (s, 3H), 2.99 (t, *J* = 7.7 Hz, 2H), 2.66 (t, *J* = 7.3 Hz, 2H), 2.41 (s, 3H) ppm.

^{13}C NMR (126 MHz, $CDCl_3$) δ 192.9, 173.4, 141.9, 139.5, 137.2, 135.9, 128.9, 127.1, 52.1, 35.7, 30.9, 21.5 ppm.

HRMS (ESI-pos) m/z: Calcd for $C_{12}H_{18}O_3N$ $[M+NH_4]^+$ 224.1281, Found 224.1275.

IR (cm^{-1}): 2951, 2845, 2730, 1734, 1695, 1598, 1436, 1365, 1275, 1259.

Methyl 3-formyl-5,6,7,8-tetrahydronaphthalene-1-carboxylate (4n):

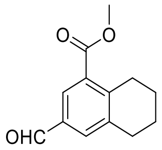

Following the general procedure C and using methyl 5,6,7,8-tetrahydronaphthalene-1-carboxylate (38.0 mg, 0.200 mmol) as substrate. ^{1}H-NMR yield and regioisomeric ratios of the intermediate olefinated compound **2n** were determined as 82%, m:(o+p) = 80:20 respectively. The assignments of **2n-*meta*** and the mixture of **2n-*ortho* and 2n-*para*** from the crude reaction mixture were achieved by comparison with the report from our group.[30] From the subsequent step, the target compound **4n** was purified by silica gel column chromatography using pentane:EtOAc = 97:3 as the eluent and obtained as a colorless crystalline oil (24.9 mg, 70%, *meta* as a single regioisomer).

^{1}H NMR (600 MHz, $CDCl_3$) δ 9.96 (s, 1H), 8.15 (d, *J* = 1.9 Hz, 1H), 7.78 – 7.67 (m, 1H), 3.91 (s, 3H), 3.24 – 3.04 (m, 2H), 2.95 – 2.84 (m, 2H), 1.91 – 1.76 (m, 4H) ppm.

^{13}C NMR (151 MHz, $CDCl_3$) δ 191.7, 167.6, 146.2, 139.8, 133.5, 133.2, 131.2, 129.7, 52.3, 30.3, 28.5, 22.8, 22.2 ppm.

HRMS (ESI-pos) m/z: Calcd for $C_{13}H_{15}O_3$ $[M+H]^+$ 219.1016, Found 219.1008.

IR (cm^{-1}): 2937, 2861, 1722, 1692, 1602, 1434.

3-((2-isopropyl-5-methylphenoxy)methyl)-5-methylbenzaldehyde (4o)

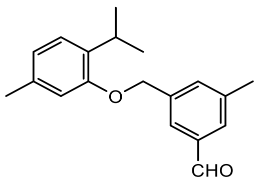

Following a slightly modified general procedure D and using 1-isopropyl-4-methyl-2-((3-methylbenzyl)oxy)benzene (50.9 mg, 0.200 mmol) as substrate. ^{1}H-NMR yield and regioisomeric ratios of the intermediate olefinated compound **2o** were determined as 59%, β:α = 92:8 respectively. To obtain full conversion of the starting material, after stirring the crude mixture for 6 h under room temperature with $NaIO_4$ (128.4 mg, 0.6000 mmol, 3.0 equiv), in CH_2Cl_2 (1 mL) and H_2O (1 mL), the reaction mixture was stirred overnight. After 24 h stirring, a second batch of $NaIO_4$ (128.4 mg, 0.6000 mmol, 3.0 equiv) and H_2O (1mL) was added and the mixture was further stirred for 18 h. Subsequently, the reaction mixture was stirred under air for 8 h. The mixture was filtered through $MgSO_4$, eluted with CH_2Cl_2 (40 mL), and concentrated under reduced pressure. The target compound **4o** was purified by silica gel column chromatography using gradient elution (pentane:Et_2O = 98:2 up to pentane:Et_2O= 96:4 as the eluent) and obtained as a colorless oil (17.6 mg, 53%, β as a single regioisomer).

^{1}H NMR (600 MHz, $CDCl_3$) δ 10.02 (s, 1H), 7.78 – 7.75 (m, 1H), 7.67 – 7.64 (m, 1H), 7.55 – 7.53 (m, 1H), 7.16 – 7.13 (m, 1H), 6.81 – 6.78 (m, 1H), 6.74 – 6.73 (m, 1H), 5.11 – 5.10 (m, 2H), 3.37 (hept, *J* = 6.9 Hz, 1H), 2.48 – 2.46 (m, 3H), 2.33 – 2.32 (m, 3H), 1.24 (d, *J* = 6.9 Hz, 6H) ppm.

^{13}C NMR (151 MHz, $CDCl_3$) δ 192.5, 155.7, 139.4, 138.9, 136.9, 136.6, 134.5, 134.0, 129.6, 126.2, 125.9, 121.9, 112.9, 69.5, 26.7, 23.0, 21.5, 21.4 ppm.

HRMS (ESI-pos) m/z: Calcd for $C_{19}H_{26}NO_2$ $[M+NH_4]^+$ 300.1958, Found 300.1947.

IR (cm^{-1}): 2959, 2922, 2868, 2726, 1698, 1607, 1253.

Methyl 5-formyl-2-methyl-4'-(trifluoromethoxy)-[1,1'-biphenyl]-3-carboxylate (4p):

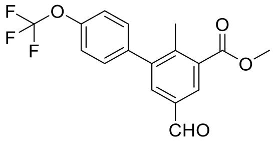

Following the general procedure D and using methyl 2-methyl-4'-(trifluoromethoxy)-[1,1'-biphenyl]-3-carboxylate (62.0 mg, 0.200 mmol) as substrate. ^{1}H-NMR yield and regioisomeric ratios of the intermediate olefinated compound **2p** were determined as 65%, β:β′:others = 80:12:8 respectively. The assignment of **2p** from the crude reaction mixture was achieved by comparison with the report from our group.[30] From the subsequent step, the target compound **4p** was purified by silica gel column chromatography using pentane:EtOAc = 96:4 as the eluent and obtained as a colorless oil (24.1 mg, 55%, β as a single regioisomer).

^{1}H NMR (500 MHz, $CDCl_3$) δ 10.03 (s, 1H), 8.40 – 8.21 (m, 1H), 7.85 (d, *J* = 1.9 Hz, 1H), 7.34 – 7.29 (m, 4H), 3.96 (s, 3H), 2.50 (s, 3H) ppm.

^{13}C NMR (151 MHz, $CDCl_3$) δ 191.1, 167.6, 149.0, 144.2, 143.6, 138.9, 133.9, 133.3, 132.5, 131.3, 130.8, 121.1, 52.6, 19.3 ppm.

^{19}F NMR (471 MHz, $CDCl_3$) δ –58.3 ppm.

HRMS (ESI-pos) m/z: Calcd for $C_{17}H_{14}O_4F_3$ $[M+H]^+$ 339.0839, Found 339.0833.

IR (cm^{-1}): 2955, 1726, 1697, 1600, 1508, 1437.

Methyl 5-(4-formyl-2,5-dimethylphenoxy)-2,2-dimethylpentanoate (4q):

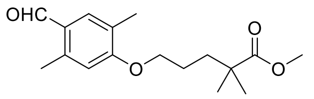

Following the general procedure D and using methyl 5-(2,5-dimethylphenoxy)-2,2-dimethylpentanoate (52.9 mg, 0.200 mmol) as substrate. ^{1}H-NMR yield and regioisomeric ratios of the intermediate olefinated compound **2q** were determined as 36%, β:α = 70:30 respectively. The assignment of **2q** from the crude reaction mixture was achieved by comparison with the report from our group.[30] From the subsequent step, the target compound **4q** was purified by silica gel column chromatography using pentane:Et_2O = 90:10 as the eluent and obtained as a colorless oil (14.7 mg, 70%, β as a single regioisomer).

^{1}H NMR (500 MHz, $CDCl_3$) δ 10.11 (s, 1H), 7.58 (s, 1H), 6.60 (s, 1H), 4.01 (t, *J* = 6.0 Hz, 2H), 3.67 (s, 3H), 2.63 (s, 3H), 2.21 (s, 3H), 1.83 – 1.74 (m, 2H), 1.75 – 1.66 (m, 2H), 1.23 (s, 6H) ppm.

^{13}C NMR (151 MHz, $CDCl_3$) δ 191.4, 178.3, 161.5, 141.3, 134.3, 127.1, 124.9, 113.4, 68.4, 51.9, 42.2, 37.1,29.8, 25.3, 25.1, 19.7, 15.7 ppm.

HRMS (ESI-pos) m/z: Calcd for $C_{17}H_{25}O_4$ $[M+H]^+$ 293.1747 Found 293.1744.

IR (cm^{-1}): 2923, 1826, 1671, 1568, 1463.

4.5 Scale-up reactions

Olefination of 1-(2,6-dimethylphenyl)ethanone and isolation as the corresponding carboxylic acid (4-acetyl-3,5-dimethylbenzoic acid, 3e)

An oven dried 150 mL Schlenk tube was charged with $Pd(OAc)_2$ (22.5 mg, 0.100 mmol, 0.100 equiv), 2-acetamido-*N*-((perfluorophenyl)sulfonyl)acetamide (**BL1**) (103.8 mg, 0.3 mmol, 0.3 equiv), Phenazine (**ML1**) (36.0 mg, 0.2 mmol, 0.2 equiv), AgOAc (498 mg, 3 mmol, 3.0 equiv) and HFIP (10 mL). The reaction mixture was stirred at room temperature for 10 minutes. 1-(2,6-dimethylphenyl)ethanone (148.2 mg, 1.0 mmol, 1 equiv) and ethyl acrylate (300 mg, 3 mmol, 3 equiv) were added to the reaction mixture, followed by the remaining HFIP (2 mL). The reaction vessel was tightly sealed and placed into the inside circle of an aluminum block (preheated to 70 °C) with a tightly fitting recess on a magnetic stirrer. The reaction mixture was stirred with 500 rpm at this temperature for 72 h.[4] After completion of the reaction, the mixture was allowed to cool to room temperature, filtered through a pad of Celite®, and eluted into a 250 mL round-bottom flask with CH_2Cl_2 (100 mL). All volatiles were removed under reduced pressure and CH_2Br_2 was added as an internal standard. ^{1}H-NMR yield and regioisomeric ratios of the intermediate olefinated compound **2e** were determined as 59%, β as a single regioisomer respectively; and the residue was used in the next step without further purification. The sample from the NMR tube was transferred back into the same flask and concentrated under reduced pressure. Following a modified literature procedure, the crude mixture from the previous step was dissolved in DMF (5 mL, 0.2 M). OsO_4 (0.315 mL, 0.01 mmol, 0.01 equiv, 2.5 (w/w)% in *t*-BuOH) and Oxone® (2.46 g, 4.0 mmol, 4.0 equiv) were added and the mixture was stirred at room temperature for 18 h. Na_2SO_3 (755 mg, 6.0 mmol, 6.0 equiv) and H_2O (20 mL) were added to reduce the remaining Os (VIII) and the resulting mixture was stirred at room temperature for one hour. EtOAc (30 mL) was added, the resulting mixture was transferred into a separatory funnel and the phases were separated. The organic phase was washed with 1 N HCl (2 x 10 mL) and sat. NaCl solution (3 x 10 mL). The organic phase was dried over Na_2SO_4, filtered, concentrated under reduced pressure and CH_2Br_2 was added as an internal standard. ^{1}H-NMR yield and regioisomeric ratios of compound **3e** were determined as 72%, β as a single regioisomer respectively. The target compound **3e** was purified by silica gel column chromatography using gradient elution (pentane:EtOAc:HCOOH = 89:10:1 up to pentane:EtOAc:HCOOH = 84:15:1 as the eluent). Due to coelution of **3e** and **ML1** during column chromatography and decomposition, all fractions containing the desired product were transferred into a flask and concentrated under reduced pressure. CH_2Cl_2 (10 mL) was added to the flask and transferred into a separatory funnel. The organic phase was acidified with 1N HCl until reaching pH 2 and washed. This procedure was done four times to fully remove **ML1**. The organic phase was dried over $MgSO_4$, filtered, and concentrated under reduced pressure, obtaining **3e** as a colorless solid (45.1 mg, 40%, β as a single regioisomer).[5] The analytical data of **3e** were in accordance to the ones reported in literature[26] and to the one reported on a 0.2 mmol scale (see Section 4.3). The isolated yield of the oxidative cleavage was calculated based on the ^{1}H-NMR yield of previous olefination step.

---

[4] To ensure consistent results, the temperature of the aluminium block was rechecked with a second thermometer. The rotation per minute was readjusted in comparison to General Procedure B to have good mixing effects.

[5] The yield of the oxidative cleavage was calculated based on the ^{1}H-NMR yield of previous olefination step. Decomposition was observed during purification leading to lower isolated yield in comparison to the yield determined by ^{1}H NMR spectroscopy.

Olefination of 1-(2,6-dimethylphenyl)ethanone and isolation as the corresponding aldehyde (4-acetyl-3,5-dimethylbenzaldehyde , 4e):

An oven dried 150 mL Schlenk tube was charged with $Pd(OAc)_2$ (22.5 mg, 0.100 mmol, 0.100 equiv), 2-acetamido-*N*-((perfluorophenyl)sulfonyl)acetamide (**BL1**) (103.8 mg, 0.3 mmol, 0.3 equiv), Phenazine (**ML1**) (36.0 mg, 0.2 mmol, 0.2 equiv), AgOAc (498 mg, 3 mmol, 3.0 equiv) and HFIP (10 mL). The reaction mixture was stirred at room temperature for 10 minutes. 1-(2,6-dimethylphenyl)ethanone (148.2 mg, 1.0 mmol, 1 equiv) and ethyl acrylate (300 mg, 3 mmol, 3 equiv) were added to the reaction mixture, followed by the remaining HFIP (2 mL). The reaction vessel was tightly sealed and placed into the inside circle of an aluminum block (preheated to 70 °C) with a tightly fitting recess on a magnetic stirrer. The reaction mixture was stirred with 500 rpm at this temperature for 72 h.[6] After completion of the reaction, the mixture was allowed to cool to room temperature, filtered through a pad of Celite®, and eluted into a 250 mL round-bottom flask with CH_2Cl_2 (100 mL). All volatiles were removed under reduced pressure and CH_2Br_2 was added as an internal standard. ^{1}H-NMR yield and regioisomeric ratios of the intermediate olefinated compound **2e** were determined as 61%, β as a single regioisomer respectively; and the residue was used in the next step without further purification. The sample from the NMR tube was transferred back into the same flask and concentrated under reduced pressure. The crude mixture from the previous step was dissolved in *t*-BuOH (5 mL). OsO_4 (0.635 mL, 0.02 mmol, 0.02 equiv, 2.50 (w/w)% in *t*-BuOH), 4-Methylmorpholine *N*-oxide (152 mg, 1.30 mmol, 1.3 equiv) and citric acid (384 mg, 2.00 mmol, 2.0 equiv) were added to the reaction mixture, followed by distilled H_2O (5 mL). The mixture was stirred at room temperature for 18 h. Na_2SO_3 (755 mg, 6.0 mmol, 6.0 equiv) and H_2O (20 mL) were added to reduce the remaining Os (VIII) and the resulting mixture was stirred at room temperature for one hour. The aqueous phase was extracted with CH_2Cl_2 (4 x 20 mL). The combined organic phases were dried over Na_2SO_4, filtered, and concentrated under reduced pressure. The residue was used in the next step without further purification.

The crude mixture from the previous step was dissolved in CH_2Cl_2 (5 mL). $NaIO_4$ (642 mg, 3 mmol, 3.0 equiv) was added and followed by H_2O (5 mL). The mixture was stirred at room temperature under N_2 atmosphere for 10 h. The reaction mixture was filtered through $MgSO_4$, eluted with CH_2Cl_2 (40 mL) and concentrated under reduced pressure. The target compound **4e** was purified by silica gel column chromatography using gradient elution (pentane:EtOAc = 98:2 up to pentane:EtOAc = 97:3 as the eluent) and obtained as a colorless solid (85.4 mg, 79%, β as a single regioisomer). The spectral data of **4e** were in accordance to the ones reported in literature[38] and to the one reported on a 0.2 mmol scale (see Section 4.4). The isolated yield of the oxidative cleavage was calculated based on the ^{1}H-NMR yield of previous olefination step.

[6] To ensure consistent results, the temperature of the aluminium block was rechecked with a second thermometer. The rotation per minute was readjusted in comparison to General Procedure B to have good mixing effects.

5 Bibliography

1. G. R. Fulmer, A. J. M. Miller, N. H. Sherden, H. E. Gottlieb, A. Nudelman, B. M. Stoltz, J. E. Bercaw and K. I. Goldberg. NMR Chemical Shifts of Trace Impurities: Common Laboratory Solvents, Organics, and Gases in Deuterated Solvents Relevant to the Organometallic Chemist. *Organometallics*, **2010**, *29*, 2176-2179.
2. K. K. Ghosh, A. Uttry, A. Koldemir, M. Ong and M. van Gemmeren. Direct β-C(sp3)–H Acetoxylation of Aliphatic Carboxylic Acids. *Org. Lett.*, **2019**, *21*, 7154-7157.
3. K. D. Collins, A. Rühling, F. Lied and F. Glorius. Rapid Assessment of Protecting-Group Stability by Using a Robustness Screen. *Chem. Eur. J.*, **2014**, *20*, 3800-3805.
4. A. Bayer, M. M. Endeshaw and O. R. Gautun. Asymmetric Hetero-Diels−Alder Reactions of N-Sulfinyl Dienophiles Using Chiral Bis(oxazoline)−Copper(II) and −Zinc(II) Triflates. *J. Org. Chem.*, **2004**, *69*, 7198-7205.
5. R. Genka and S. Arimitsu. Redox-economical synthesis of α-substituted α-N-phthaloyl amino aldehydes using Fukuyama reduction. *Tetrahedron Lett.*, **2024**, *137*, 154938.
6. J. D. S. A. Boyd, I. Gunawardana, I. C. Jacobson, Y. Lehuerou, M. L. Lupher, Jr. M. Mclaughlin, S. Miller, A. Thomas, E. Thorsett, R. Xu, M. Yanik, Gan Zhang, 2005019200A2, 2004.
7. A. D. Fotiadou and A. L. Zografos. Accessing the Structural Diversity of Pyridone Alkaloids: Concise Total Synthesis of Rac-Citridone A. *Org. Lett.*, **2011**, *13*, 4592-4595.
8. Y. Zheng, Y. Zhao, S. Tao, X. Li, X. Cheng, G. Jiang and X. Wan. Green Esterification of Carboxylic Acids Promoted by tert-Butyl Nitrite. *Eur. J. Org. Chem.*, **2021**, *2021*, 2713-2718.
9. W.-D. Li, Y. Wu, S.-J. Li, Y.-Q. Jiang, Y.-L. Li, Y. Lan and J.-B. Xia. Boryl Radical Activation of Benzylic C–OH Bond: Cross-Electrophile Coupling of Free Alcohols and CO2 via Photoredox Catalysis. *J. Am. Chem. Soc.*, **2022**, *144*, 8551-8559.
10. C. Yuan, L. Zhu, R. Zeng, Y. Lan and Y. Zhao. Ruthenium(II)-Catalyzed C−H Difluoromethylation of Ketoximes: Tuning the Regioselectivity from the meta to the para Position. *Angew. Chem., Int. Ed.*, **2018**, *57*, 1277-1281.
11. S.-Q. Chen, X.-R. Li, C.-J. Li, J. Fan, Z.-W. Liu and X.-Y. Shi. Aldehyde as a Traceless Directing Group for Regioselective C–H Alkylation Catalyzed by Rhodium(III) in Air. *Org. Lett.*, **2020**, *22*, 1259-1264.
12. J. Dhankhar, M. D. Hofer, A. Linden and I. Čorić. Site-Selective C−H Arylation of Diverse Arenes Ortho to Small Alkyl Groups. *Angew. Chem., Int. Ed.*, **2022**, *61*, e202205470.
13. B. S. Takale, R. R. Thakore, S. Handa, F. Gallou, J. Reilly and B. H. Lipshutz. A new, substituted palladacycle for ppm level Pd-catalyzed Suzuki–Miyaura cross couplings in water. *Chem. Sci.*, **2019**, *10*, 8825-8831.
14. L. Niu, J. Liu, H. Yi, S. Wang, X.-A. Liang, A. K. Singh, C.-W. Chiang and A. Lei. Visible-Light-Induced External Oxidant-Free Oxidative Phosphonylation of C(sp2)–H Bonds. *ACS Catal.*, **2017**, *7*, 7412-7416.
15. M. Farizyan, A. Mondal, S. Mal, F. Deufel and M. van Gemmeren. Palladium-Catalyzed Nondirected Late-Stage C–H Deuteration of Arenes. *J. Am. Chem. Soc.*, **2021**, *143*, 16370-16376.
16. B. R. Travis, R. S. Narayan and B. Borhan. Osmium Tetroxide-Promoted Catalytic Oxidative Cleavage of Olefins: An Organometallic Ozonolysis. *J. Am. Chem. Soc.*, **2002**, *124*, 3824-3825.
17. G. Meng, J.-L. Yan, N. Chekshin, D. A. Strassfeld and J.-Q. Yu. Ligand-Controlled Nondirected meta- or para-C–H Olefination of Silyl-Protected Phenols. *ACS Catal.*, **2024**, *14*, 12806-12813.
18. H. Chen, P. Wedi, T. Meyer, G. Tavakoli and M. van Gemmeren. Dual Ligand-Enabled Nondirected C−H Olefination of Arenes. *Angew. Chem., Int. Ed.*, **2018**, *57*, 2497-2501.
19. Y. Ichikawa, M. Hiramatsu, Y. Mita, M. Makishima, Y. Matsumoto, Y. Masumoto, A. Muranaka, M. Uchiyama, Y. Hashimoto and M. Ishikawa. meta-Non-flat substituents: a

novel molecular design to improve aqueous solubility in small molecule drug discovery. *Org. Biomol. Chem.*, **2021**, *19*, 446-456.
20. N. Tada, K. Hattori, T. Nobuta, T. Miura and A. Itoh. Facile aerobic photooxidation of methyl group in the aromatic nucleus in the presence of an organocatalyst under VIS irradiation. *Green Chem.*, **2011**, *13*, 1669-1671.
21. K. Naksomboon, C. Valderas, M. Gómez-Martínez, Y. Álvarez-Casao and M. Á. Fernández-Ibáñez. S,O-Ligand-Promoted Palladium-Catalyzed C–H Functionalization Reactions of Nondirected Arenes. *ACS Catal.*, **2017**, *7*, 6342-6346.
22. X.-Y. Wang, Z.-P. Shang, G.-F. Zha, X.-Q. Chen, S. N. A. Bukhari and H.-L. Qin. [Ru(bpy)3]Cl2-catalyzed aerobic oxidative cleavage β-diketones to carboxylic acids under visible light irradiation. *Tetrahedron Lett.*, **2016**, *57*, 5628-5631.
23. H. T. Kim, E. Kang, M. Kim and J. M. Joo. Synthesis of Bidentate Nitrogen Ligands by Rh-Catalyzed C–H Annulation and Their Application to Pd-Catalyzed Aerobic C–H Alkenylation. *Org. Lett.*, **2021**, *23*, 3657-3662.
24. S. J. Yun, J. Kim, E. Kang, H. Jung, H. T. Kim, M. Kim and J. M. Joo. Nondirected Pd-Catalyzed C–H Perdeuteration and meta-Selective Alkenylation of Arenes Enabled by Pyrazolopyridone Ligands. *ACS Catal.*, **2023**, *13*, 4042-4052.
25. A. Gevorgyan, K. H. Hopmann and A. Bayer. Formal C−H Carboxylation of Unactivated Arenes. *Chem. Eur. J.*, **2020**, *26*, 6064-6069.
26. J. f. Al-Ka'bi, J. A. Farooqi, P. H. Gore, A. M. G. Nassar, E. F. Saad, E. L. Short and D. N. Waters. Protiodeacylation of 4-substituted 1-acetyl-2,6-dimethylbenzenes in sulphuric acid: kinetics and mechanism. *J. Chem. Soc.*, **1988**, 943-949.
27. A. Sidduri, J. W. Tilley, J. P. Lou, L. Chen, G. Kaplan, F. Mennona, R. Campbell, R. Guthrie, T.-N. Huang, K. Rowan, V. Schwinge and L. M. Renzetti. N-Aroyl-l-Phenylalanine Derivatives as VCAM/VLA-4 Antagonists. *Bioorg. Med. Chem. Lett.*, **2002**, *12*, 2479-2482.
28. P. Wang, P. Verma, G. Xia, J. Shi, J. X. Qiao, S. Tao, P. T. W. Cheng, M. A. Poss, M. E. Farmer, K.-S. Yeung and J.-Q. Yu. Ligand-accelerated non-directed C–H functionalization of arenes. *Nature*, **2017**, *551*, 489-493.
29. G. Chen, Z. Zhuang, G.-C. Li, T. G. Saint-Denis, Y. Hsiao, C. L. Joe and J.-Q. Yu. Ligand-Enabled β-C–H Arylation of α-Amino Acids Without Installing Exogenous Directing Groups. *Angew. Chem., Int. Ed.*, **2017**, *56*, 1506-1509.
30. C. Santiago, H. Chen, A. Mondal and M. van Gemmeren. Dual Ligand-Enabled Late-Stage Fujiwara–Moritani Reactions. *Synlett*, **2021**, *33*, 357-360.
31. K. Ghosh and I. Saha. Triphenylamine-based simple chemosensor for selective fluorometric detection of fluoride, acetate and dihydrogenphosphate ions in different solvents. *J. Inclusion Phenom. Macrocyclic Chem.*, **2011**, *70*, 97-107.
32. M. Austin, O. J. Egan, R. Tully and A. C. Pratt. Quinoline synthesis: scope and regiochemistry of photocyclisation of substituted benzylidenecyclopentanone O-alkyl and O-acetyloximes. *Org. Biomol. Chem.*, **2007**, *5*, 3778-3786.
33. Y. Imada, Y. Okada, K. Noguchi and K. Chiba. Selective Functionalization of Styrenes with Oxygen Using Different Electrode Materials: Olefin Cleavage and Synthesis of Tetrahydrofuran Derivatives. *Angew. Chem., Int. Ed.*, **2019**, *58*, 125-129.
34. Š. Možina, S. Stavber and J. Iskra. Dual Catalysis for the Aerobic Oxidation of Benzyl Alcohols – Nitric Acid and Fluorinated Alcohol. *Eur. J. Org. Chem.*, **2017**, *2017*, 448-452.
35. A. Kumari, M. Suresh and R. B. Singh. Total synthesis of the proposed structure of Anti-TMV active tabasesquiterpene A. *Tetrahedron*, **2021**, *92*, 132282.
36. N. Tibrewal, T. E. Downey, S. G. Van Lanen, E. Ul Sharif, G. A. O'Doherty and J. Rohr. Roles of the Synergistic Reductive O-Methyltransferase GilM and of O-Methyltransferase GilMT in the Gilvocarcin Biosynthetic Pathway. *J. Am. Chem. Soc.*, **2012**, *134*, 12402-12405.
37. F. M. R. Clark, K. Kira, S. Hirota, H. Azuma, T. Nagakura, T. Horizoe, K. Tabata, K. Kusano, T. Omae, A. Inoue, WIPO (PCT), 2007111212A1, 2007.
38. H. Shen, B. Yang, J. Lin, M. Zheng and H. Jiang. Selective Electrochemical Benzylic C(sp3)−H Oxidations in Fluoroalcohols. *ChemistrySelect*, **2023**, *8*, e202302073.

39. D. E. Anderson, M. B. Kim, J. T. Moore, T. E. O'Brien, N. A. Sorto, C. I. Grove, L. L. Lackner, J. B. Ames and J. T. Shaw. Comparison of Small Molecule Inhibitors of the Bacterial Cell Division Protein FtsZ and Identification of a Reliable Cross-Species Inhibitor. *ACS Chem. Biol.*, **2012**, *7*, 1918-1928.
40. S. Perveen, S. Zhang, L. Wang, P. Song, Y. Ouyang, J. Jiao, X.-H. Duan and P. Li. Synthesis of Axially Chiral Biaryls via Enantioselective Ullmann Coupling of ortho-Chlorinated Aryl Aldehydes Enabled by a Chiral 2,2′-Bipyridine Ligand. *Angew. Chem., Int. Ed.*, **2022**, *61*, e202212108.

6 NMR Spectra

Triisopropyl(m-tolyloxy)silane (1d):

^{1}H NMR spectrum (500 MHz) in $CDCl_3$

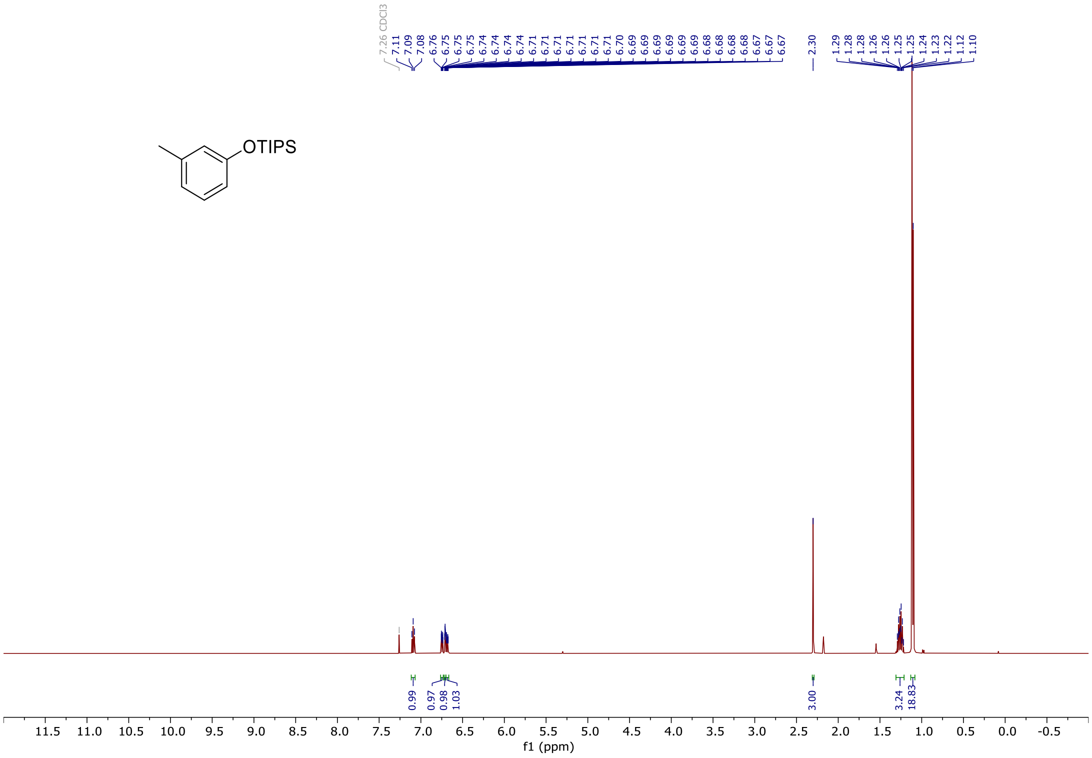

^{13}C NMR spectrum (126 MHz) in $CDCl_3$

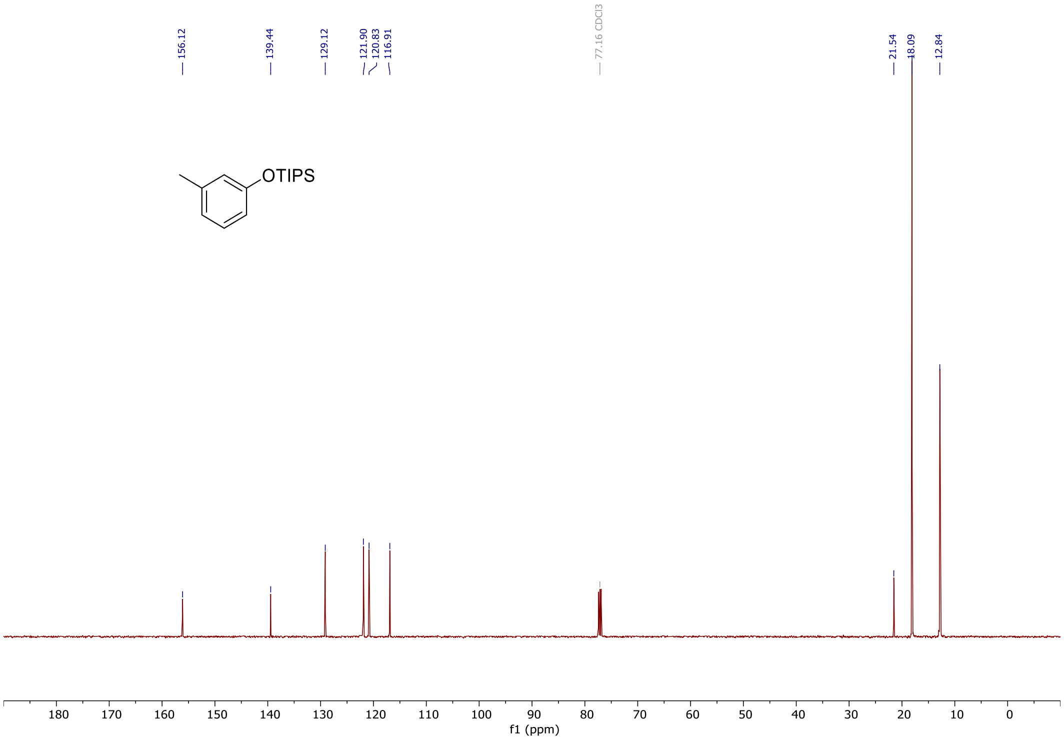

Methyl 2-(1,3-dioxoisoindolin-2-yl)-3-phenylpropanoate (1h):

^{1}H NMR spectrum (500 MHz) in $CDCl_3$

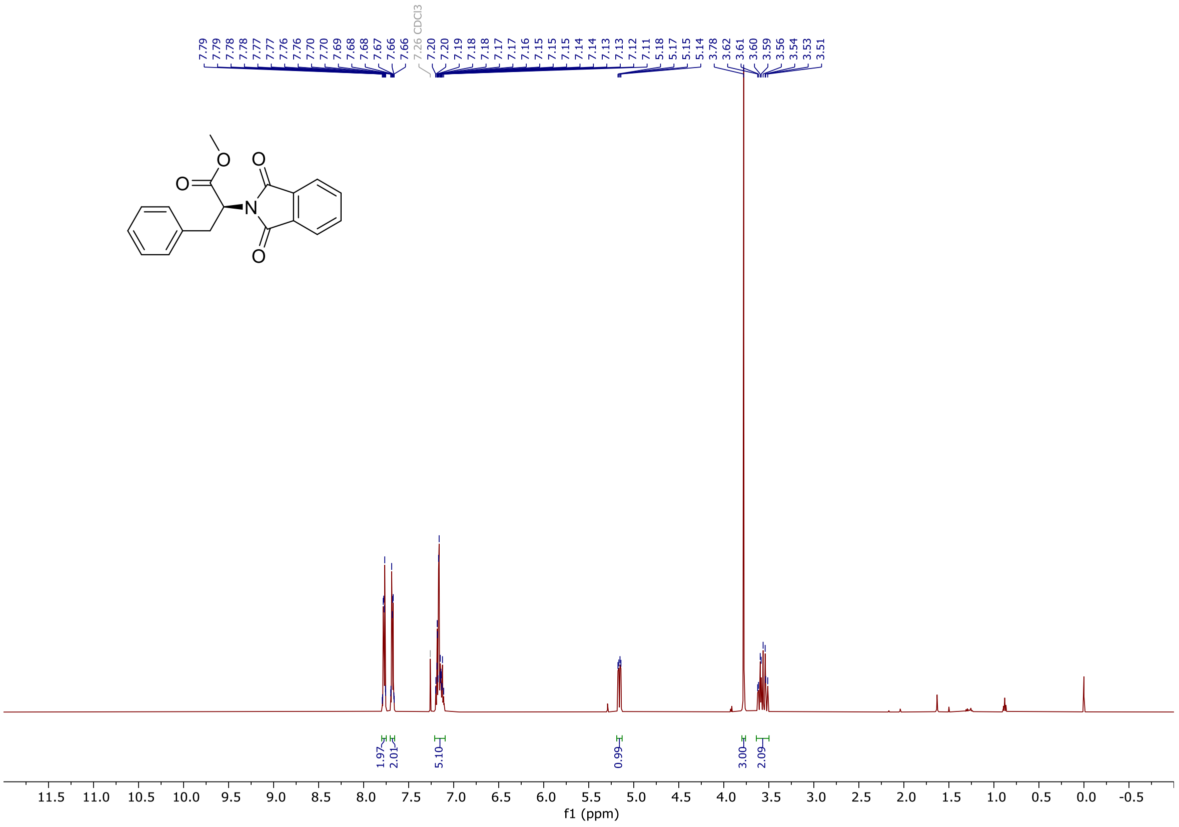

^{13}C NMR spectrum (126 MHz) in $CDCl_3$

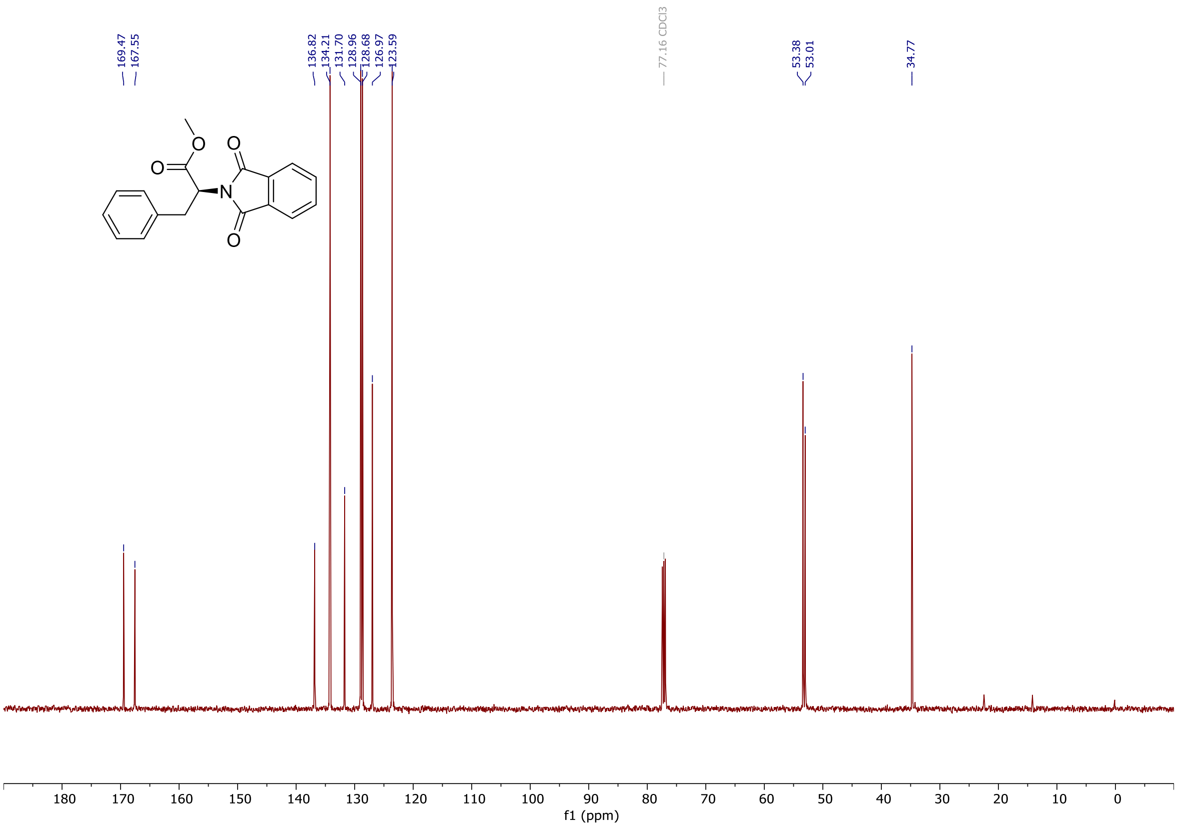

Ethyl (S)-4-benzyl-3-propionyloxazolidin-2-one (1i):

^{1}H NMR spectrum (500 MHz) in $CDCl_3$

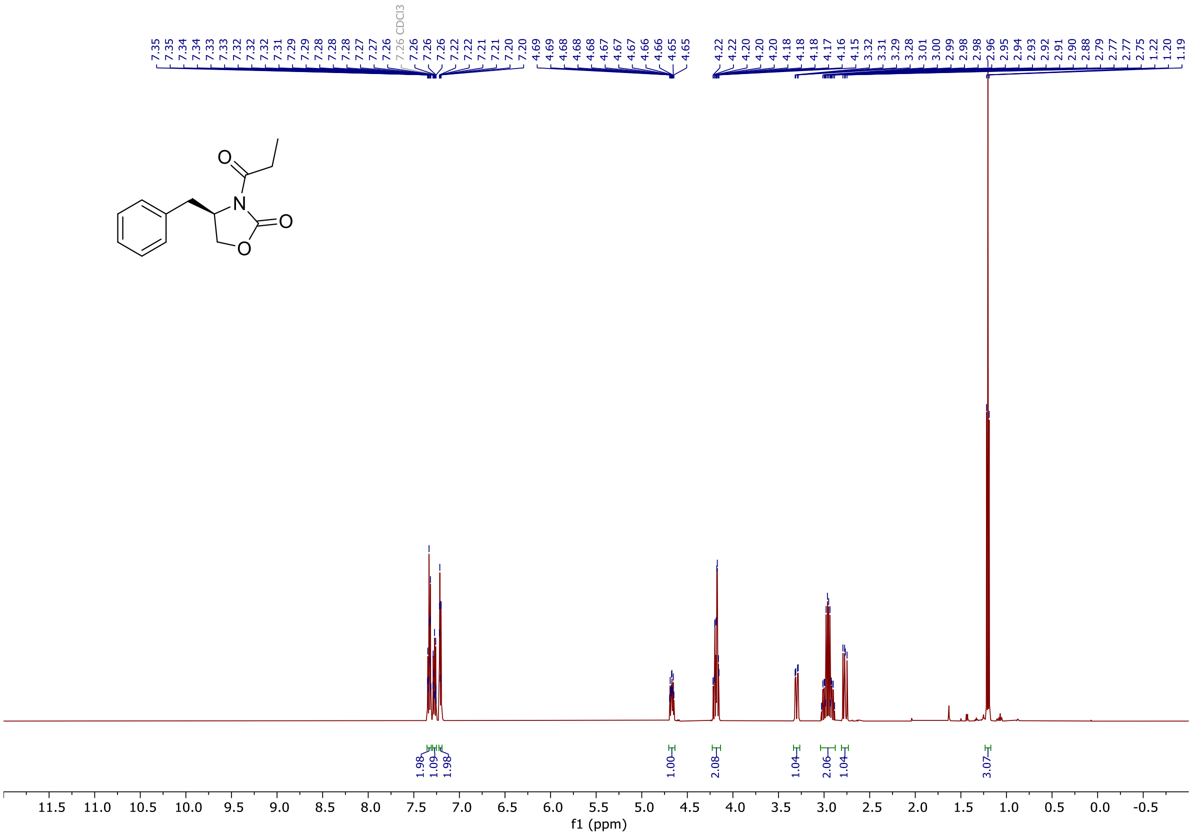

^{13}C NMR spectrum (126 MHz) in $CDCl_3$

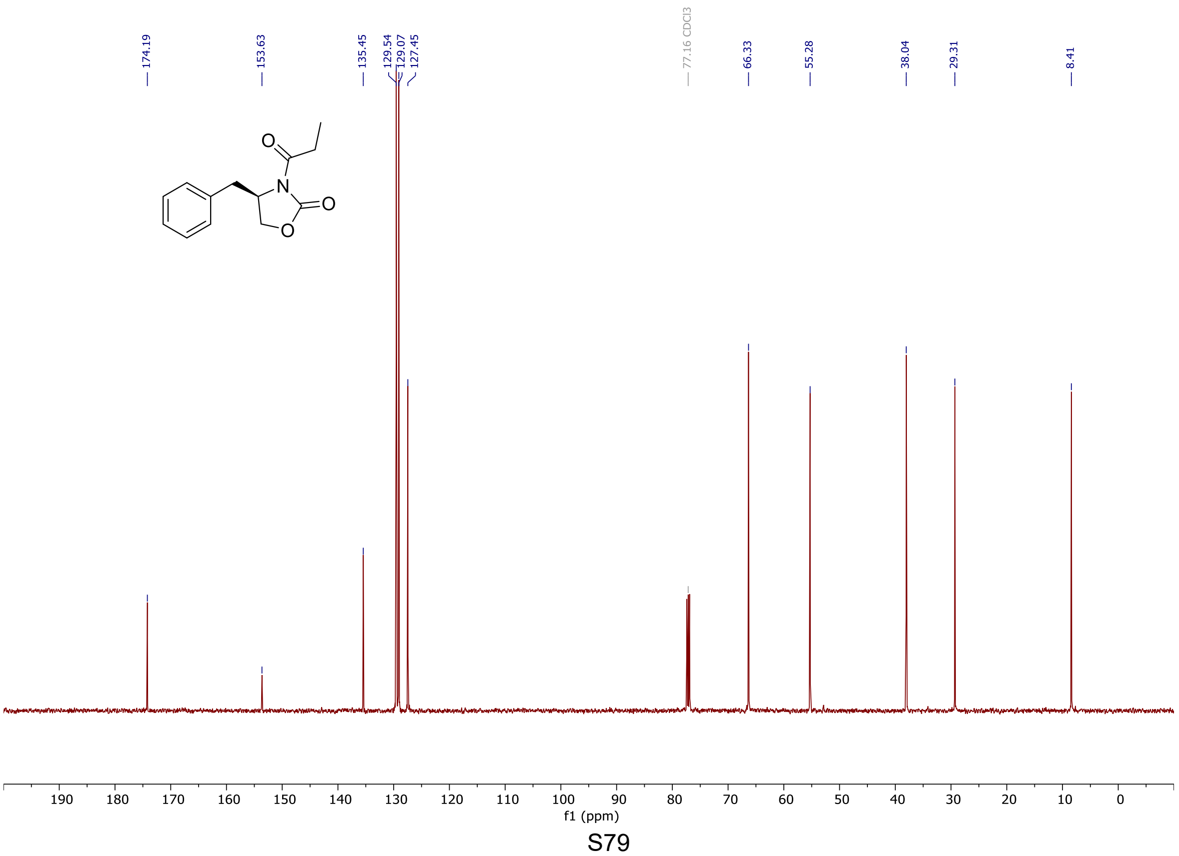

(*R*)-methyl 2-((1*s*,4*S*)-4-isopropylcyclohexanecarboxamido)-3-phenylpropanoate (1j):

^{1}H NMR spectrum (500 MHz) in $CDCl_3$

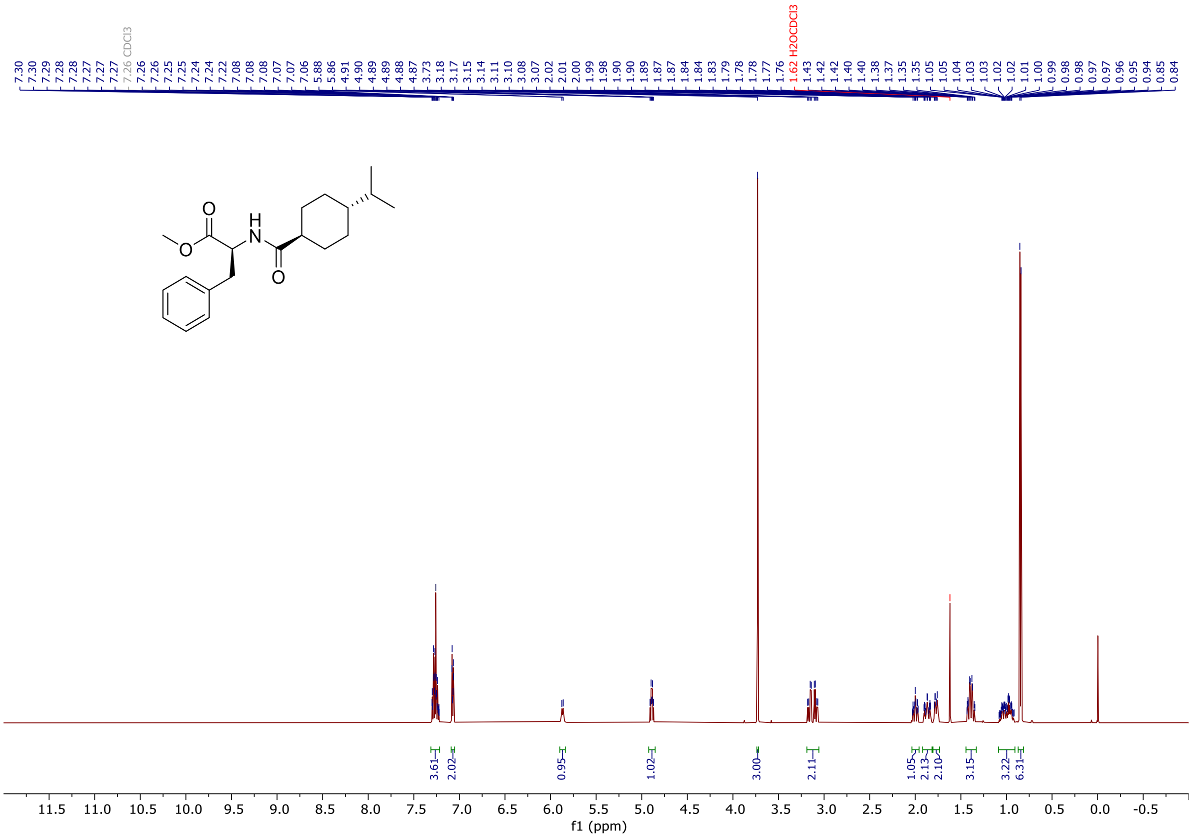

^{13}C NMR spectrum (126 MHz) in $CDCl_3$

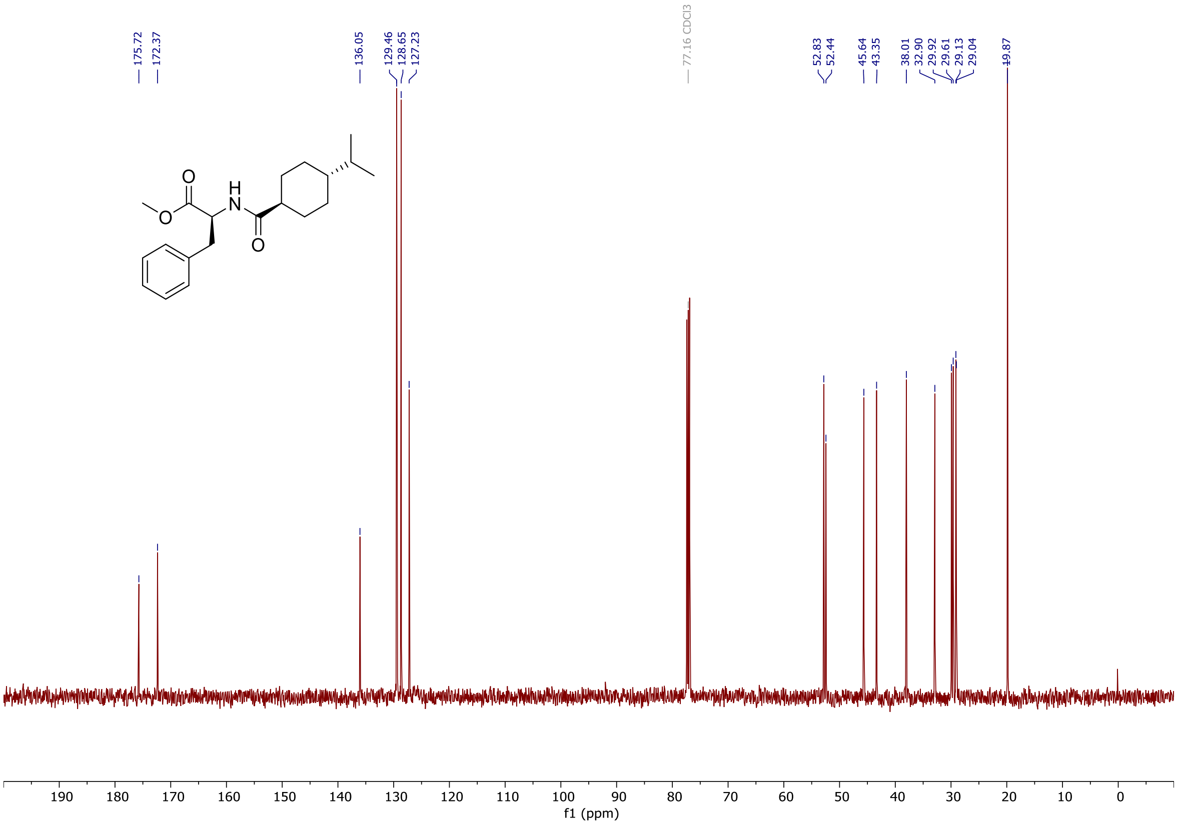

Methyl 2-(2-fluoro-[1,1'-biphenyl]-4-yl)propanoate (1k):

^{1}H NMR spectrum (500 MHz) in $CDCl_3$

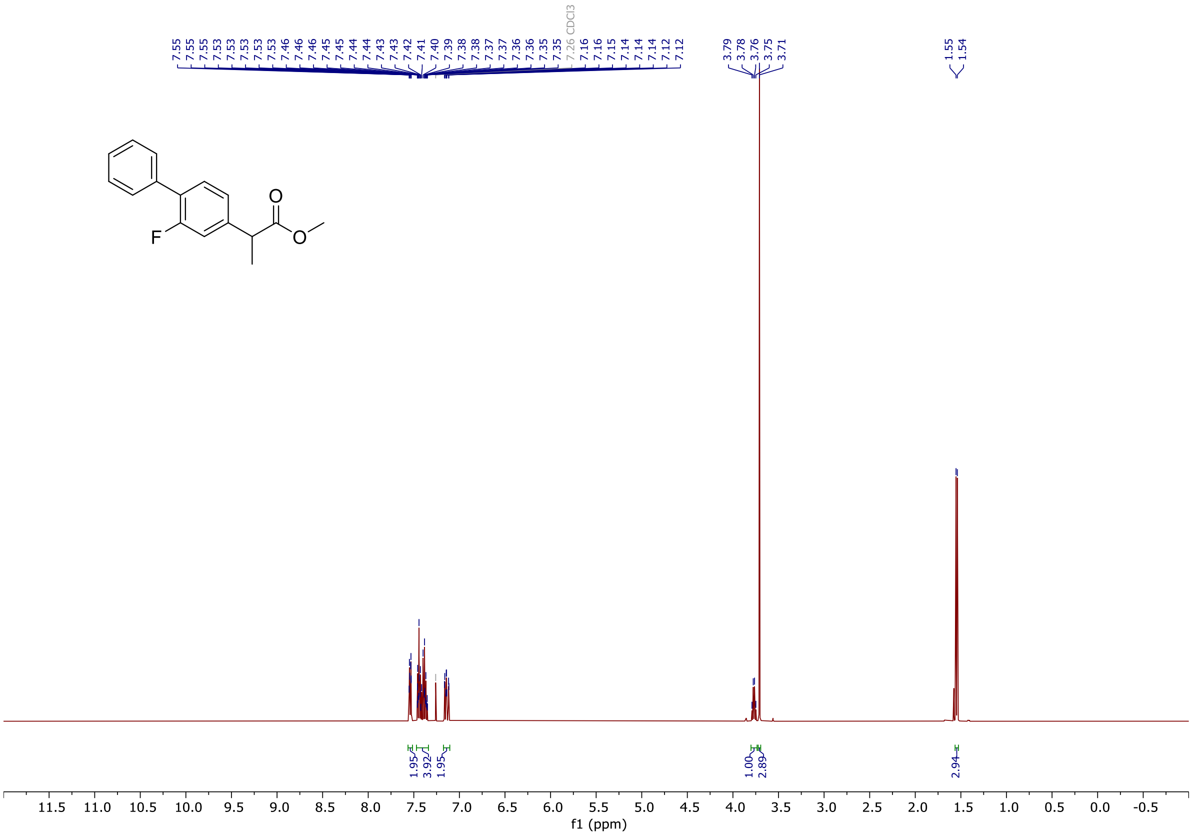

^{13}C NMR spectrum (126 MHz) in $CDCl_3$

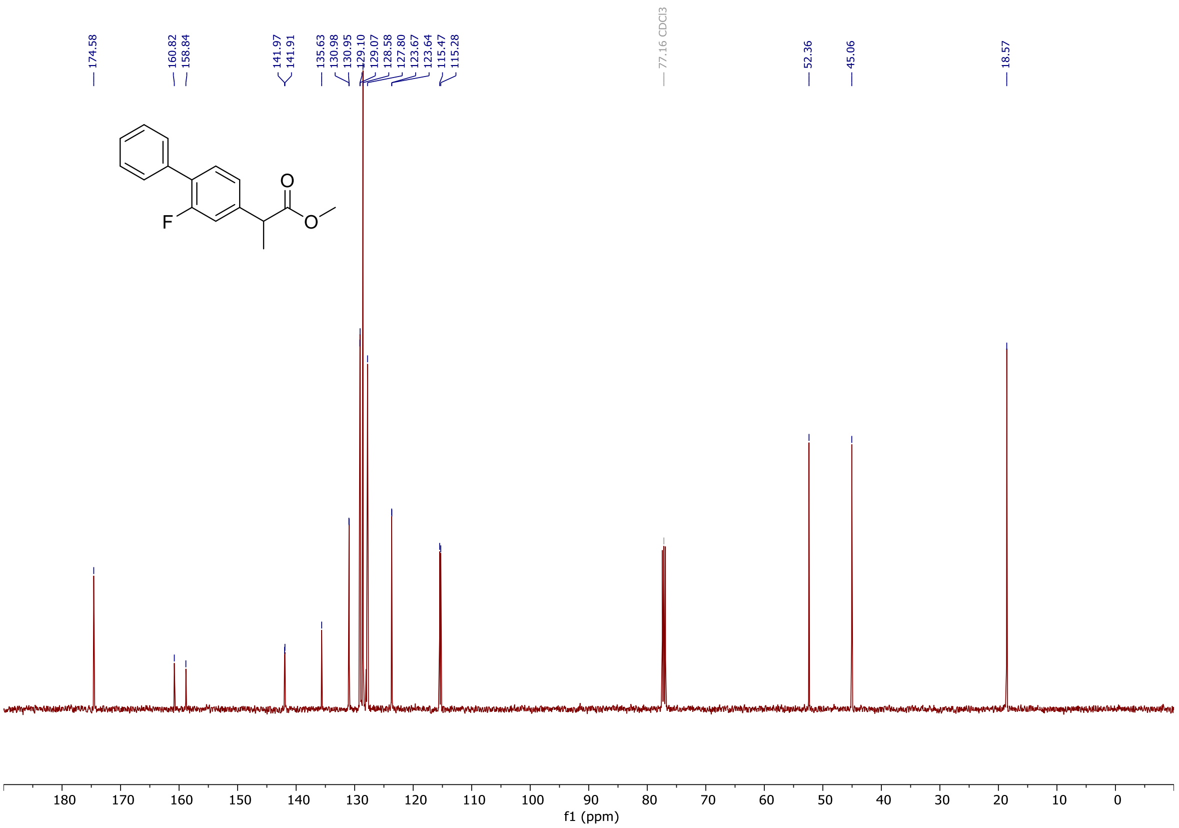

^{19}F NMR spectrum (471 MHz) in $CDCl_3$

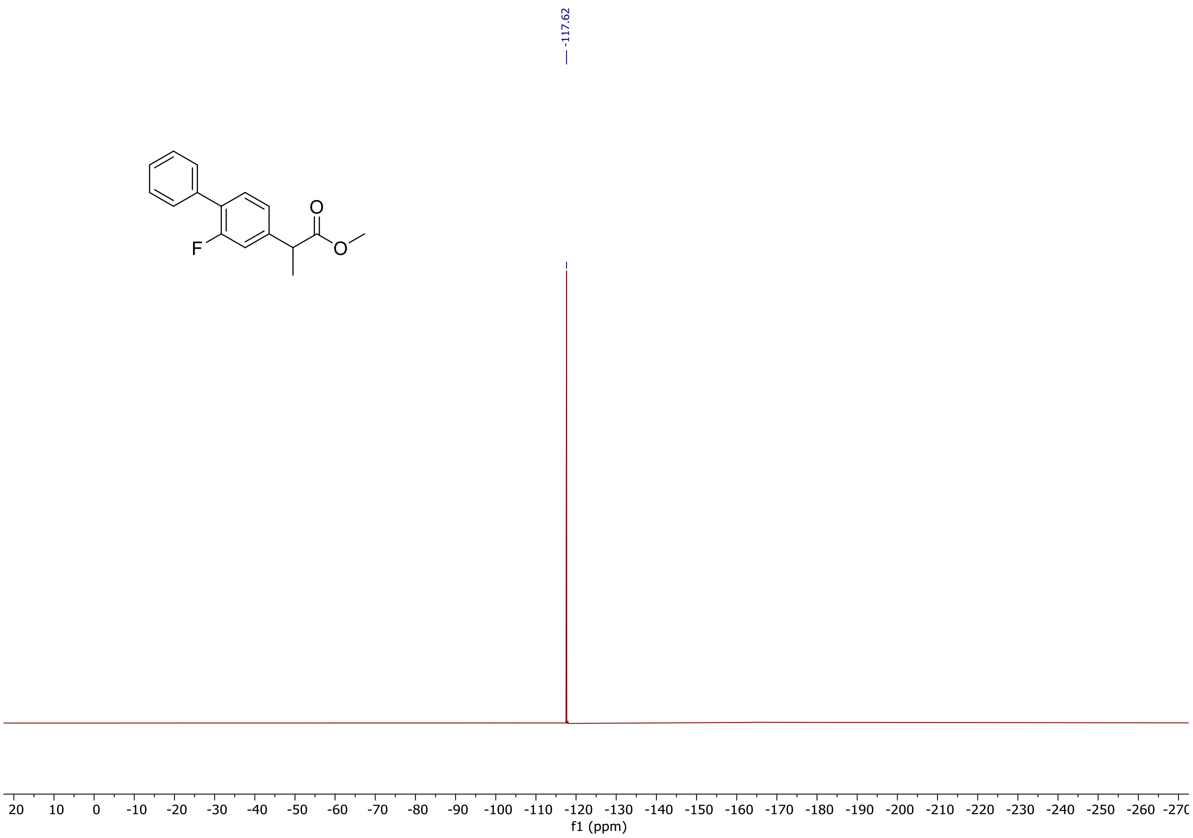

Methyl 2-(3-benzoylphenyl)propanoate (1l):

^1H NMR spectrum (600 MHz) in $CDCl_3$

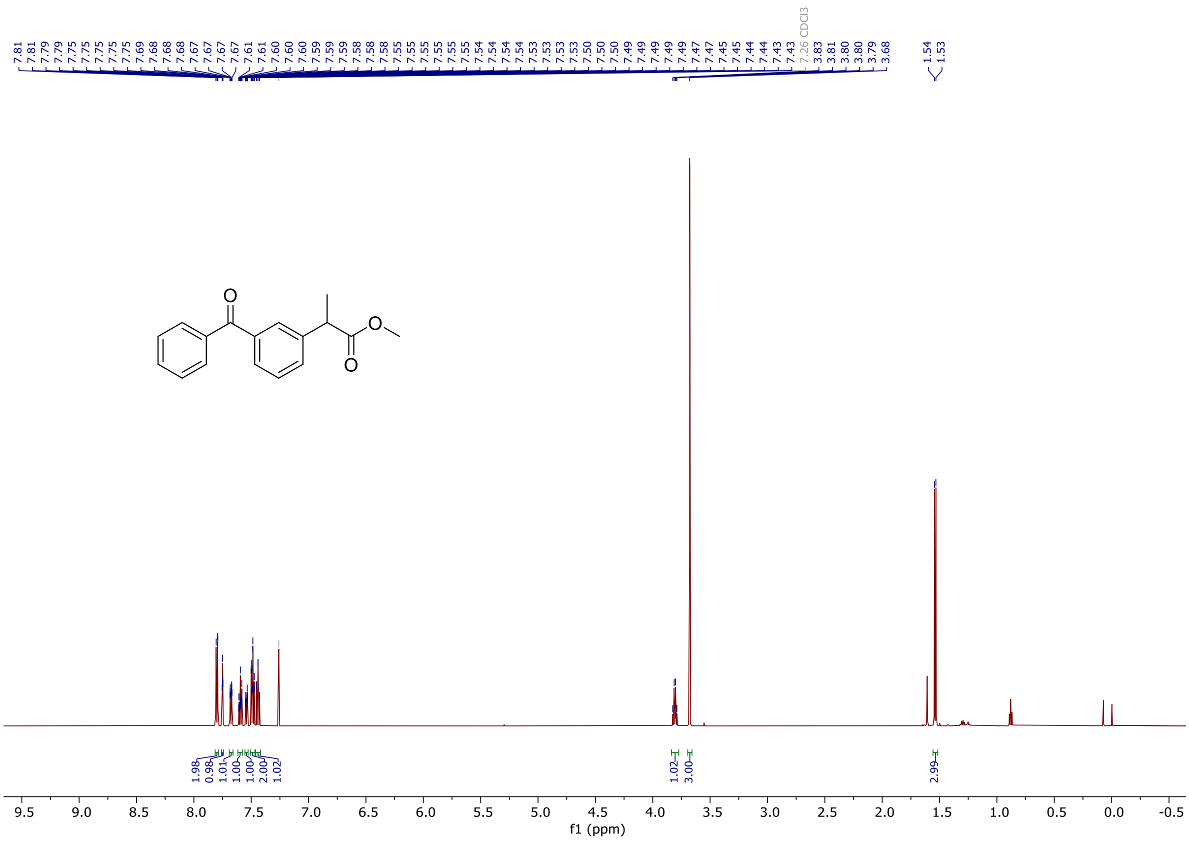

^{13}C NMR spectrum (151 MHz) in $CDCl_3$

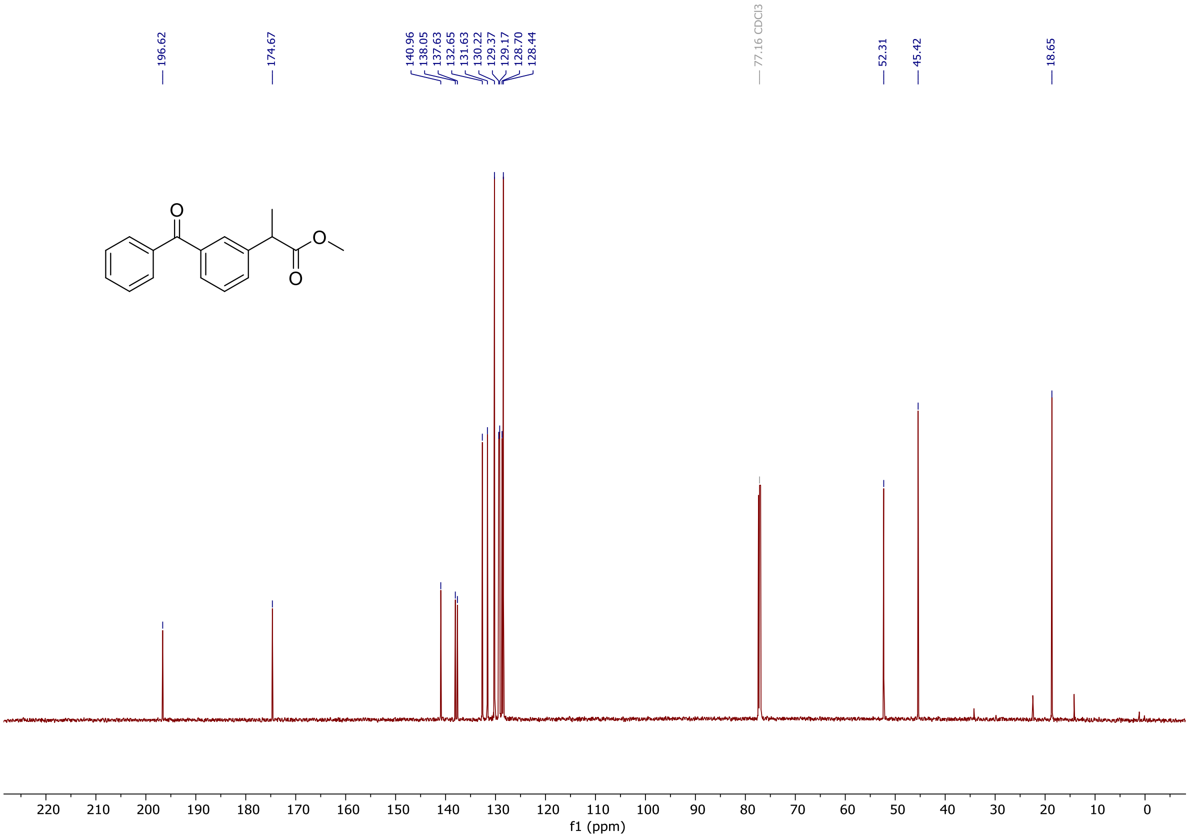

Methyl 3-(*m*-tolyl)propanoate (1m):

^{1}H NMR spectrum (500 MHz) in CDCl$_3$

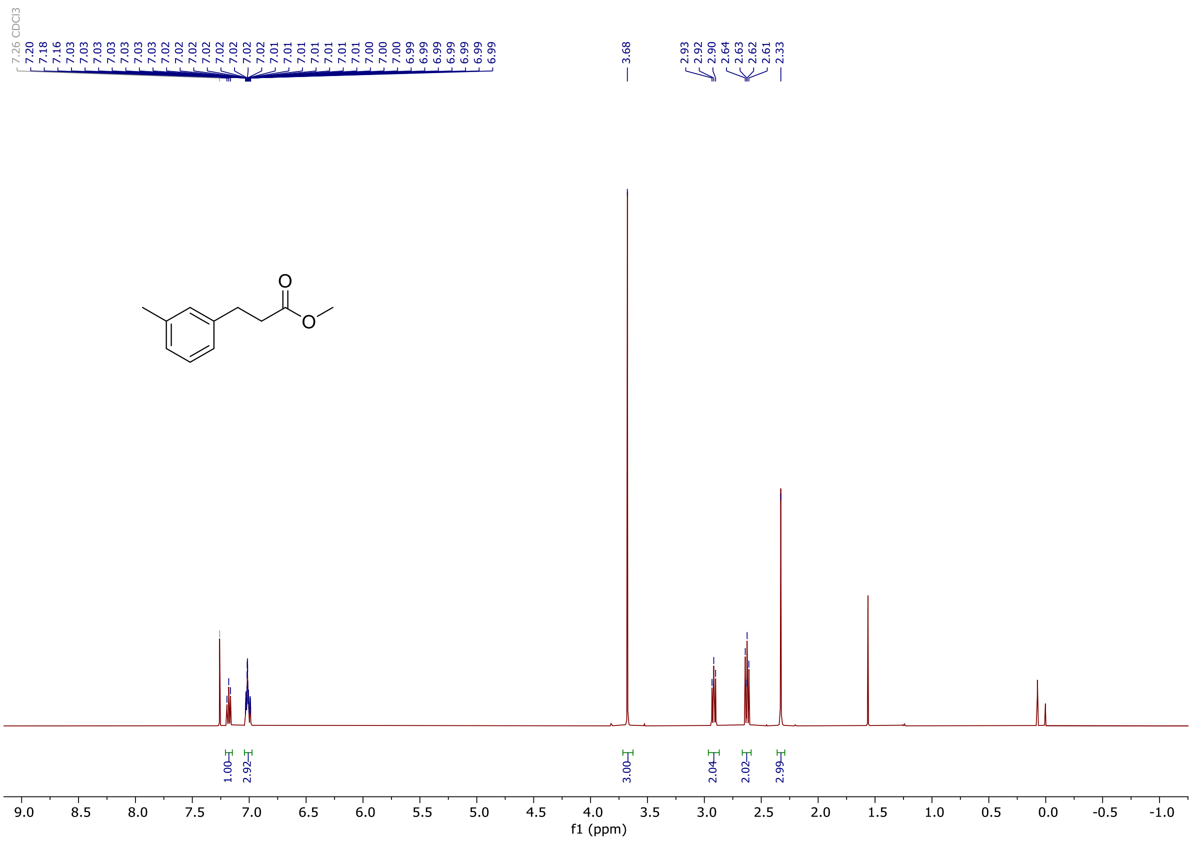

^{13}C NMR spectrum (126 MHz) in CDCl$_3$

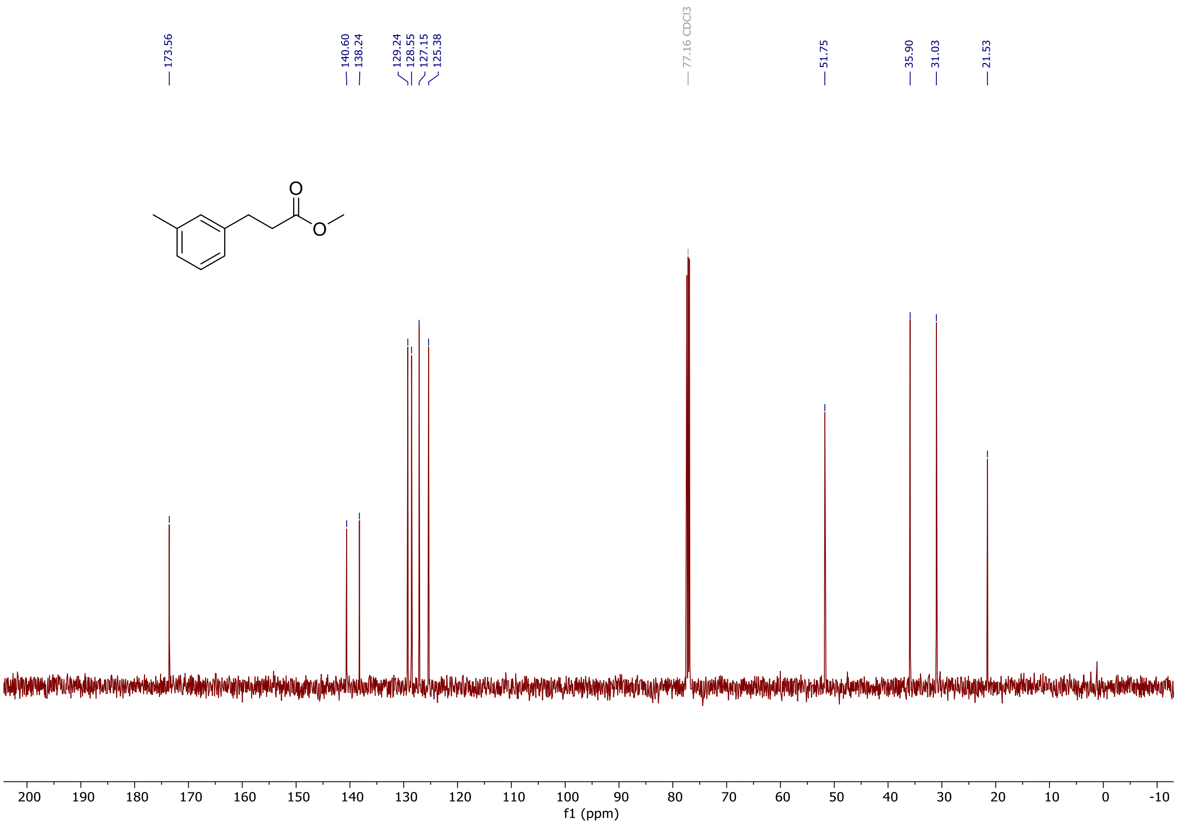

Methyl 5,6,7,8-tetrahydronaphthalene-1-carboxylate (1n):

^{1}H NMR spectrum (600 MHz) in $CDCl_3$

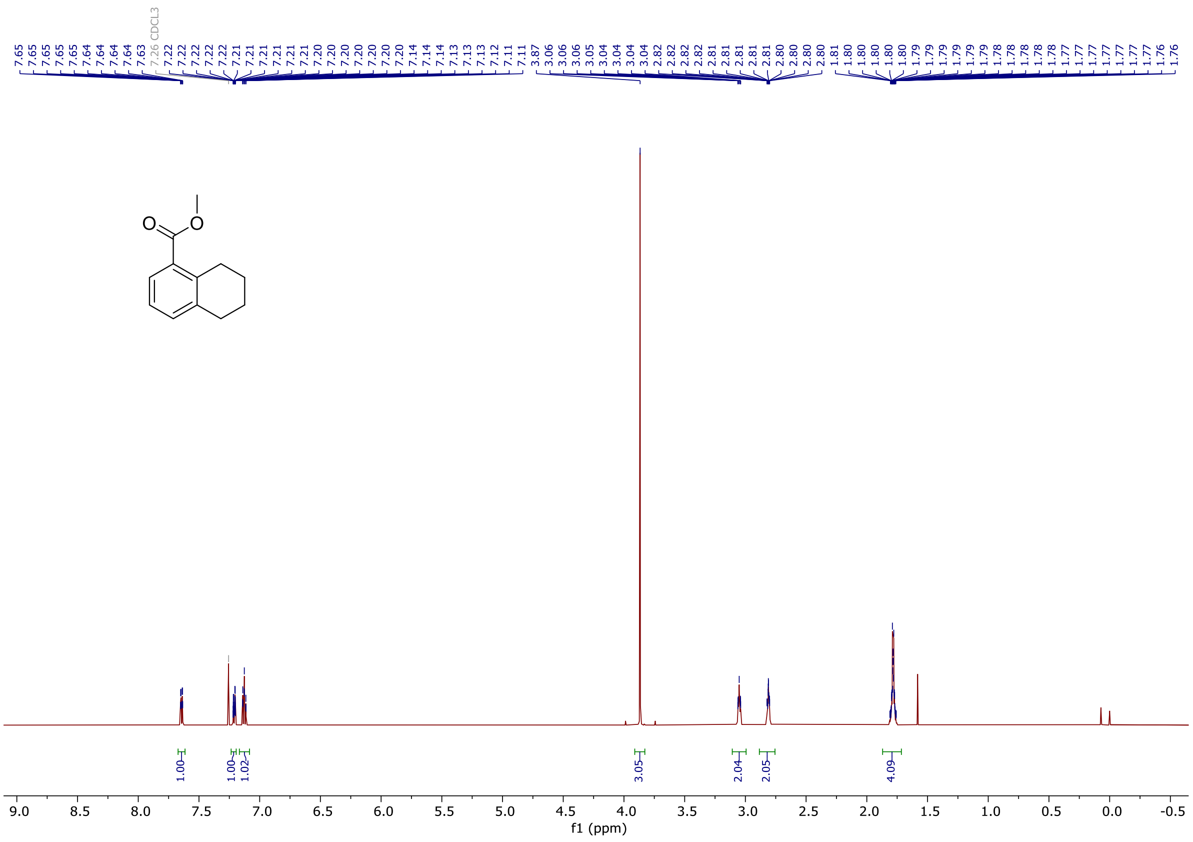

^{13}C NMR spectrum (151 MHz) in $CDCl_3$

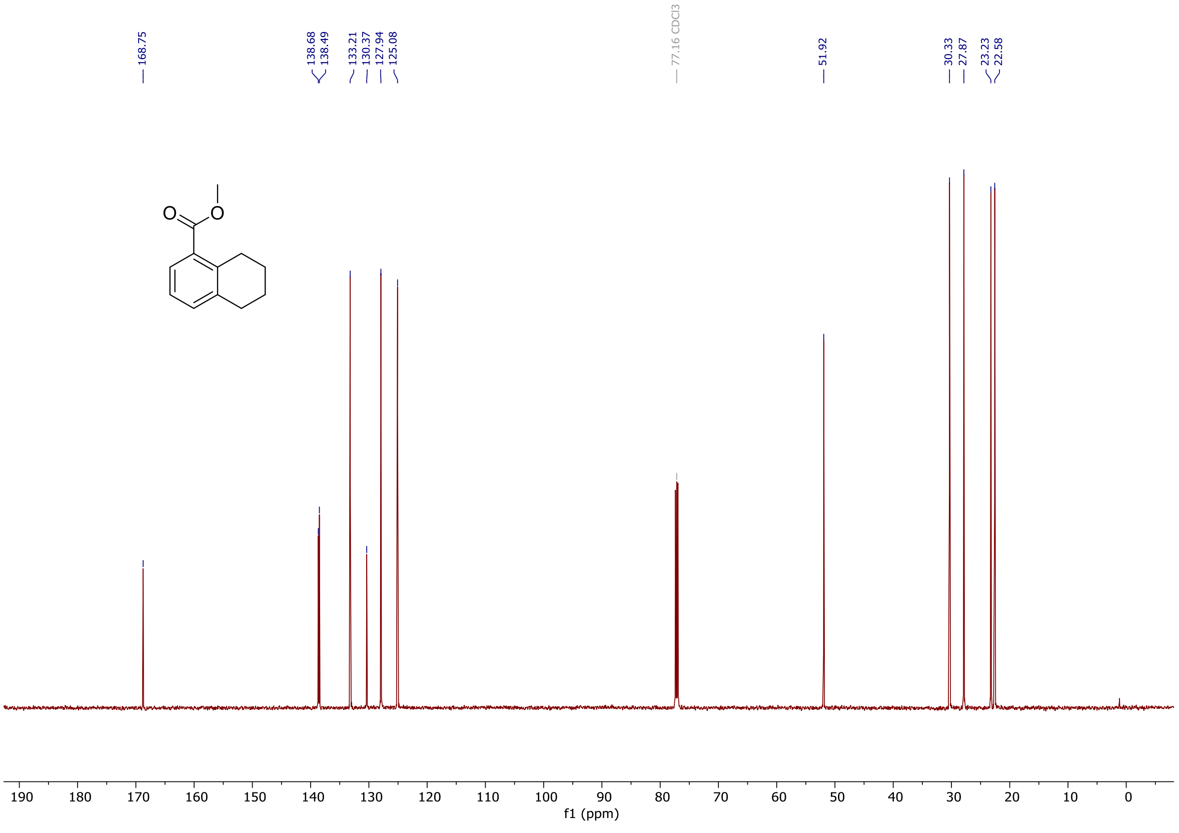

Methyl 2-methyl-4'-(trifluoromethoxy)-[1,1'-biphenyl]-3-carboxylate (1p):

^{1}H NMR spectrum (500 MHz) in Acetone-d_6

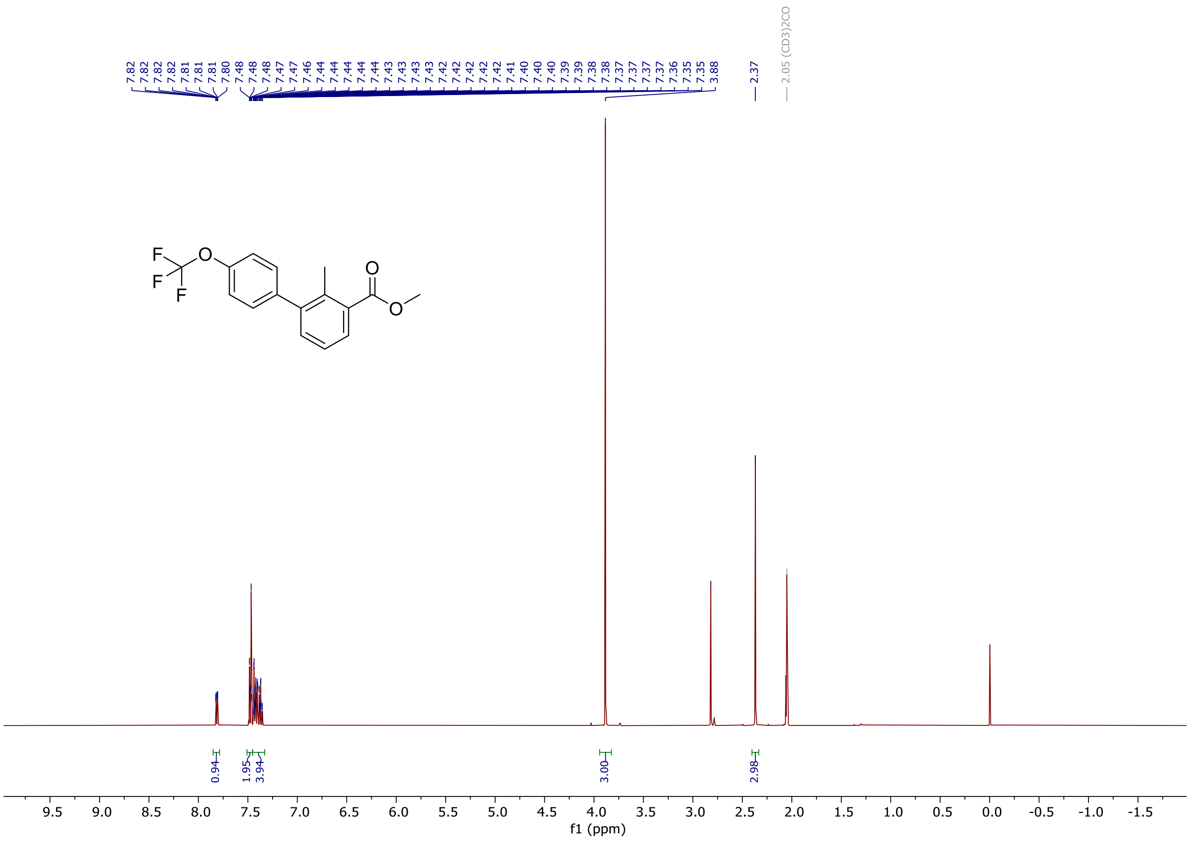

^{13}C NMR spectrum (126 MHz) in Acetone-d_6

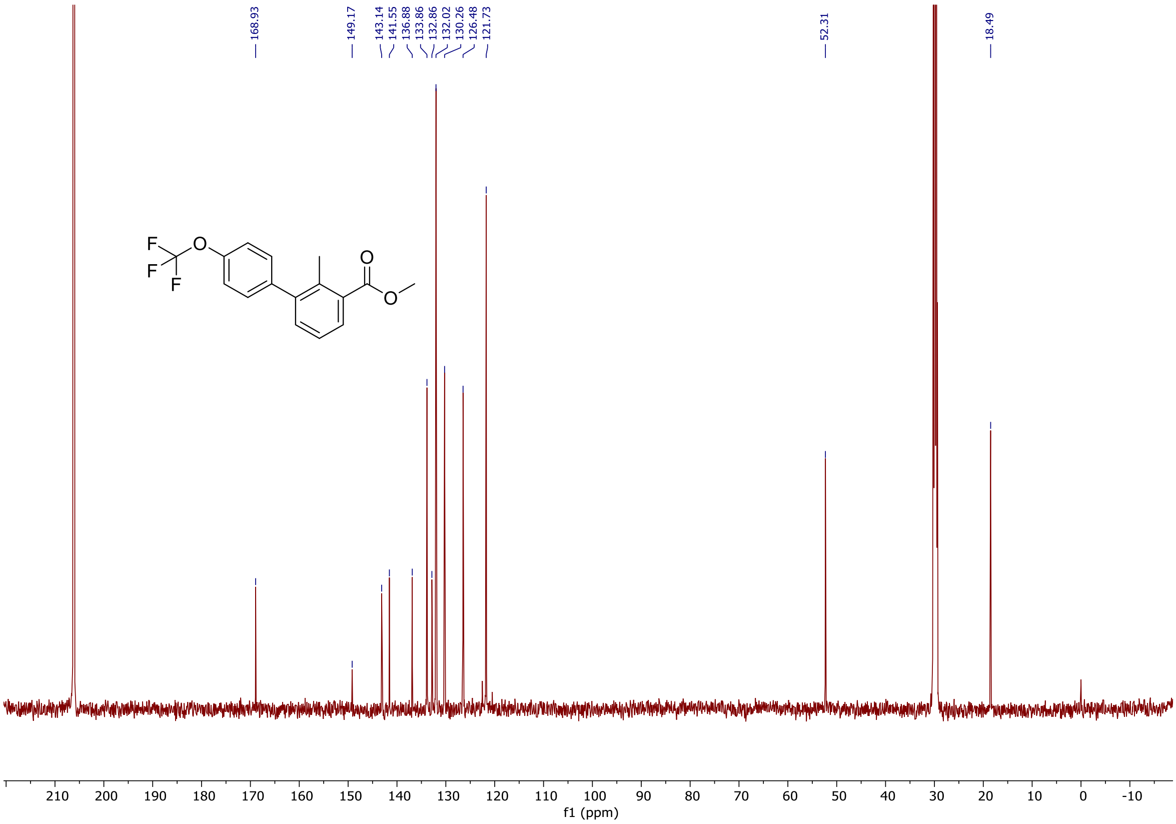

^{19}F NMR spectrum (471 MHz) in Acetone-d_6

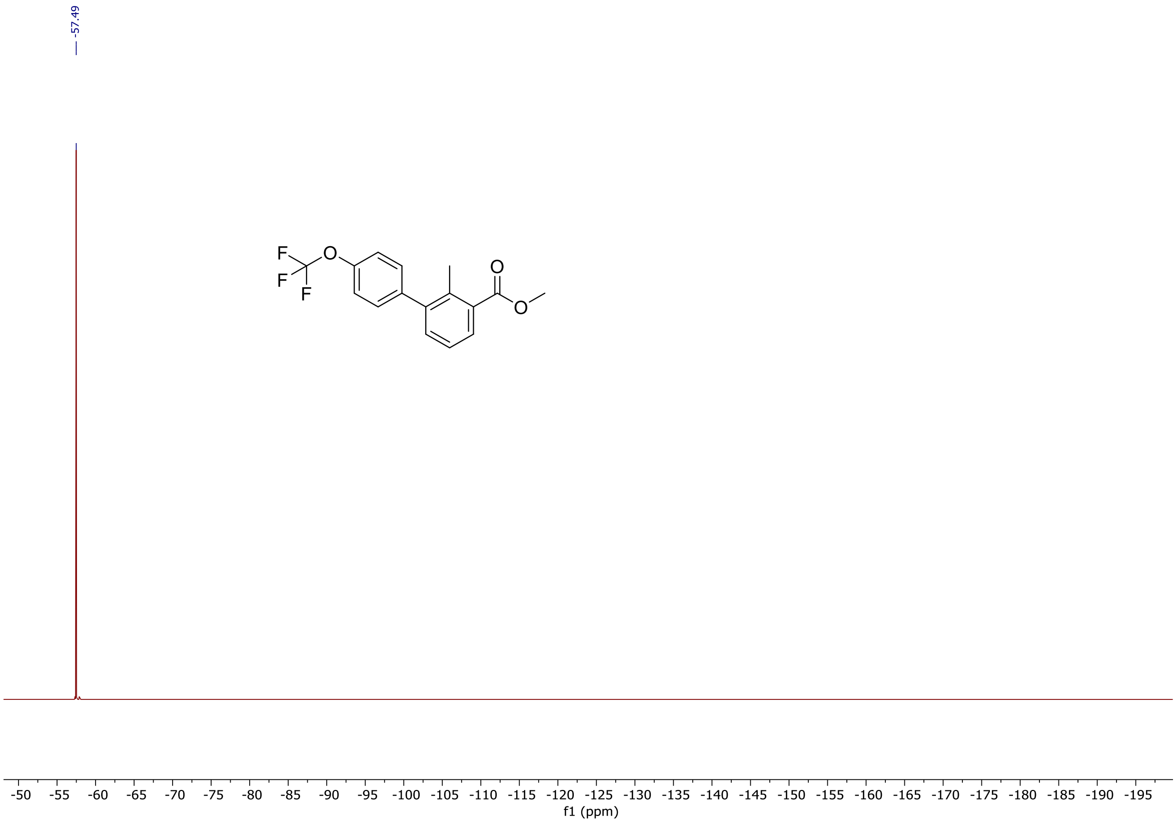
-57.49
-50 -55 -60 -65 -70 -75 -80 -85 -90 -95 -100 -105 -110 -115 -120 -125 -130 -135 -140 -145 -150 -155 -160 -165 -170 -175 -180 -185 -190 -195
f1 (ppm)

Methyl 5-(2,5-dimethylphenoxy)-2,2-dimethylpentanoate (1q):

^{1}H NMR spectrum (600 MHz) in CDCl$_3$

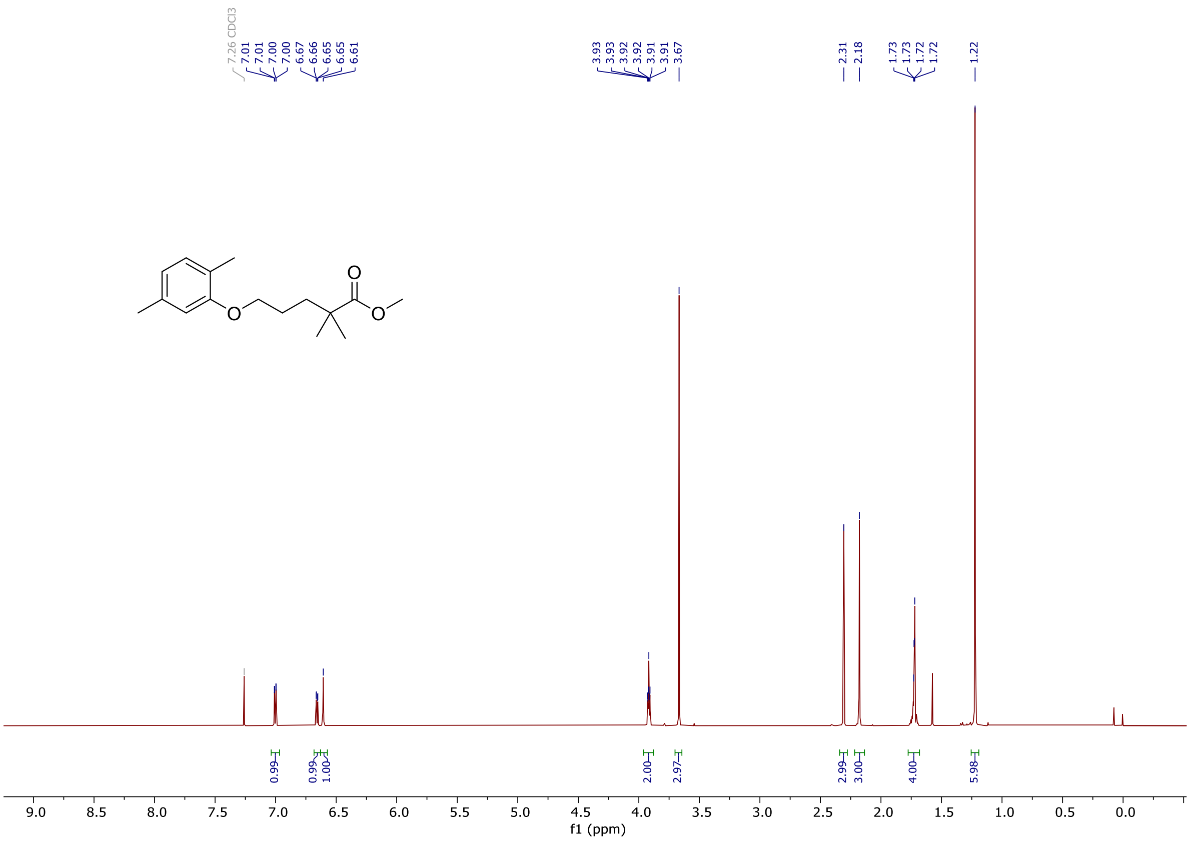

^{13}C NMR spectrum (151 MHz) in CDCl$_3$

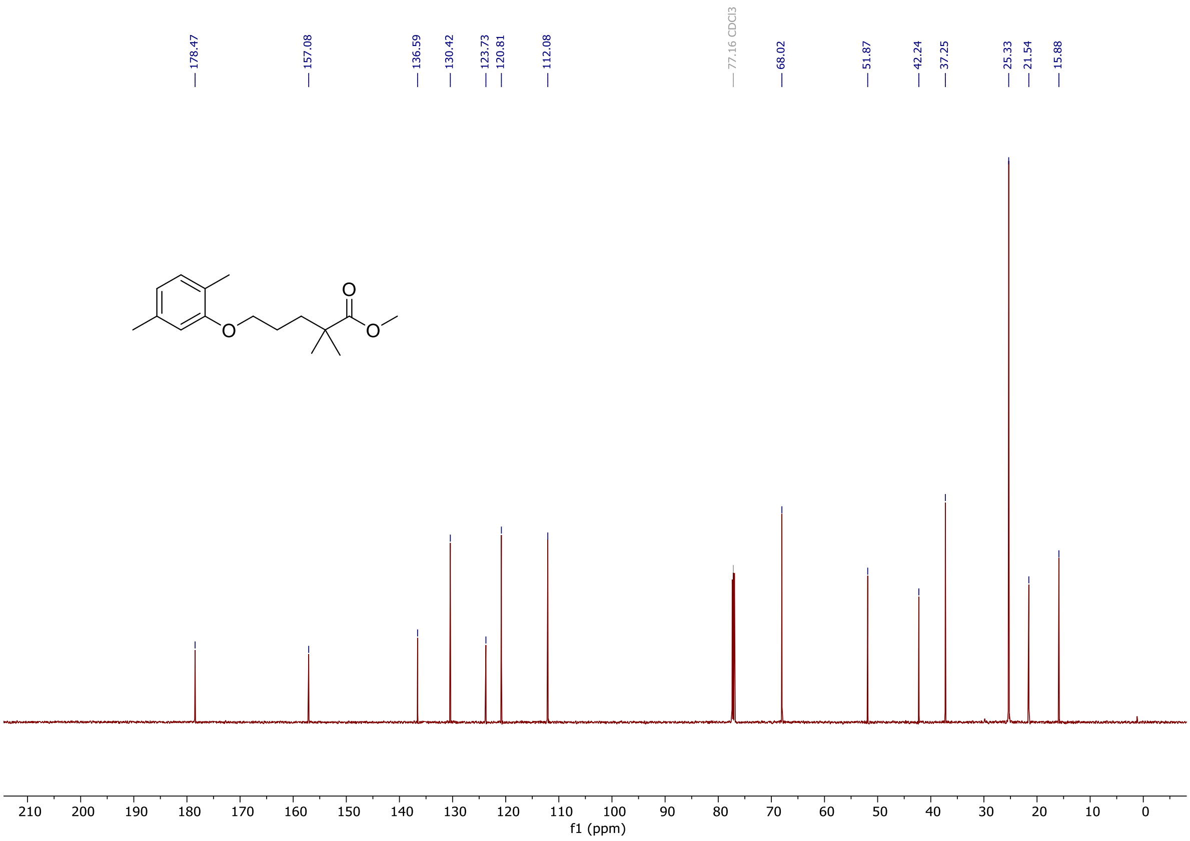

2-acetamido-*N*-((perfluorophenyl)sulfonyl)acetamide (BL1):

^{1}H NMR spectrum (500 MHz) in DMSO-d$_6$

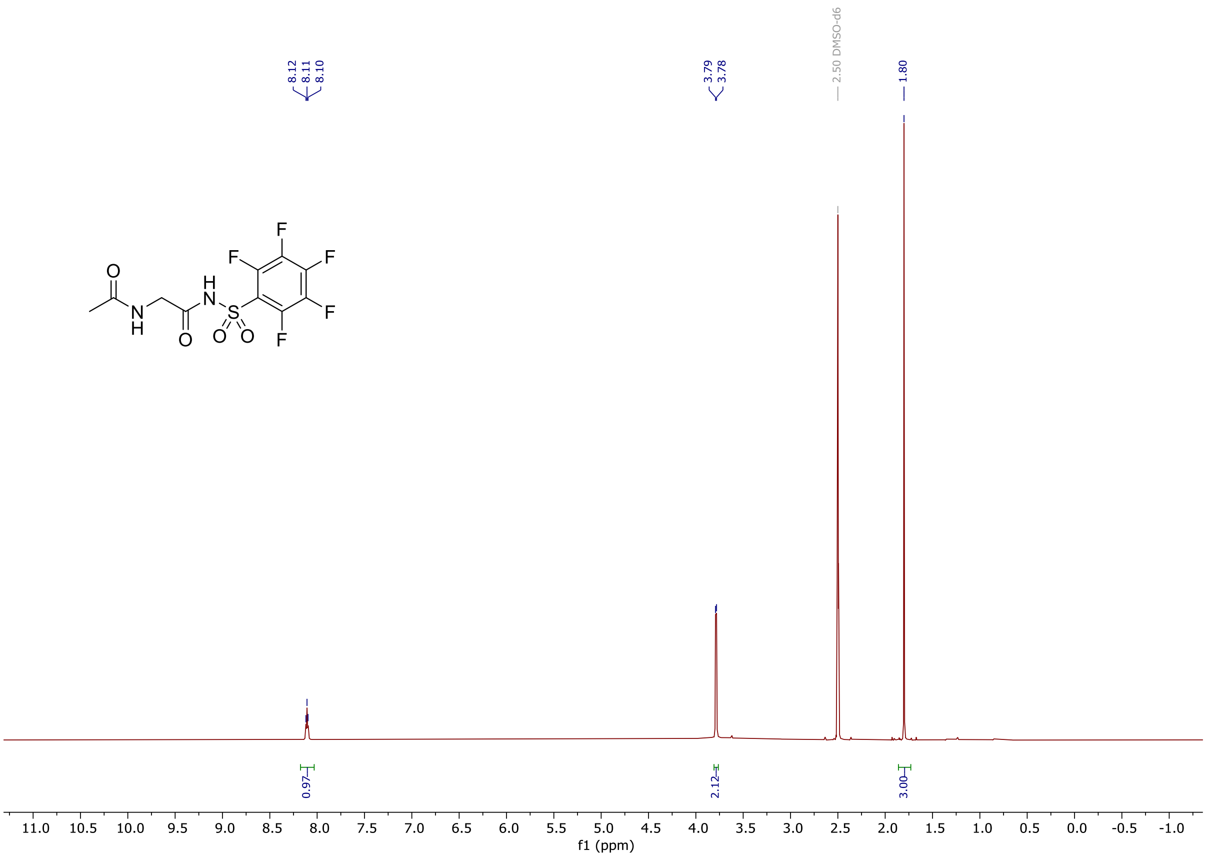

^{13}C NMR spectrum (126 MHz) in DMSO-d$_6$

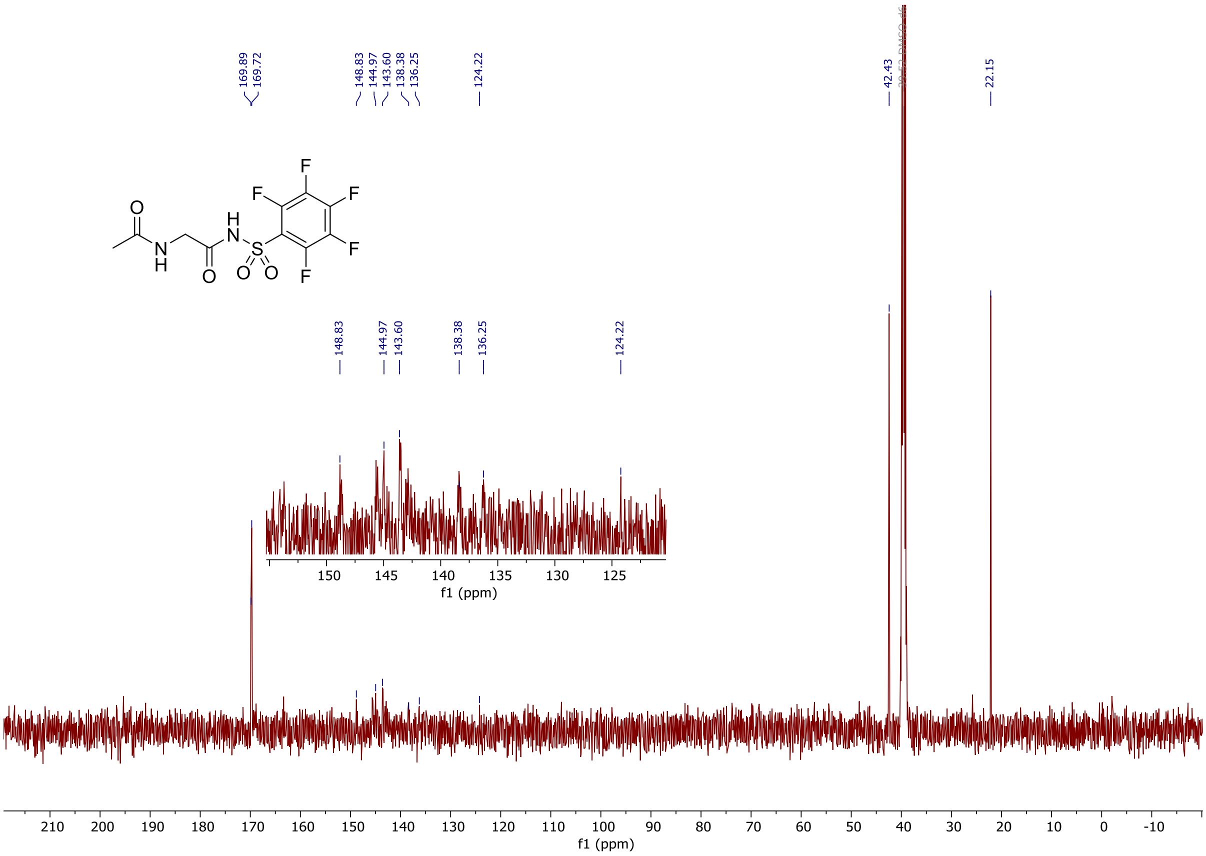

^{19}F NMR spectrum (471 MHz) in DMSO-d_6

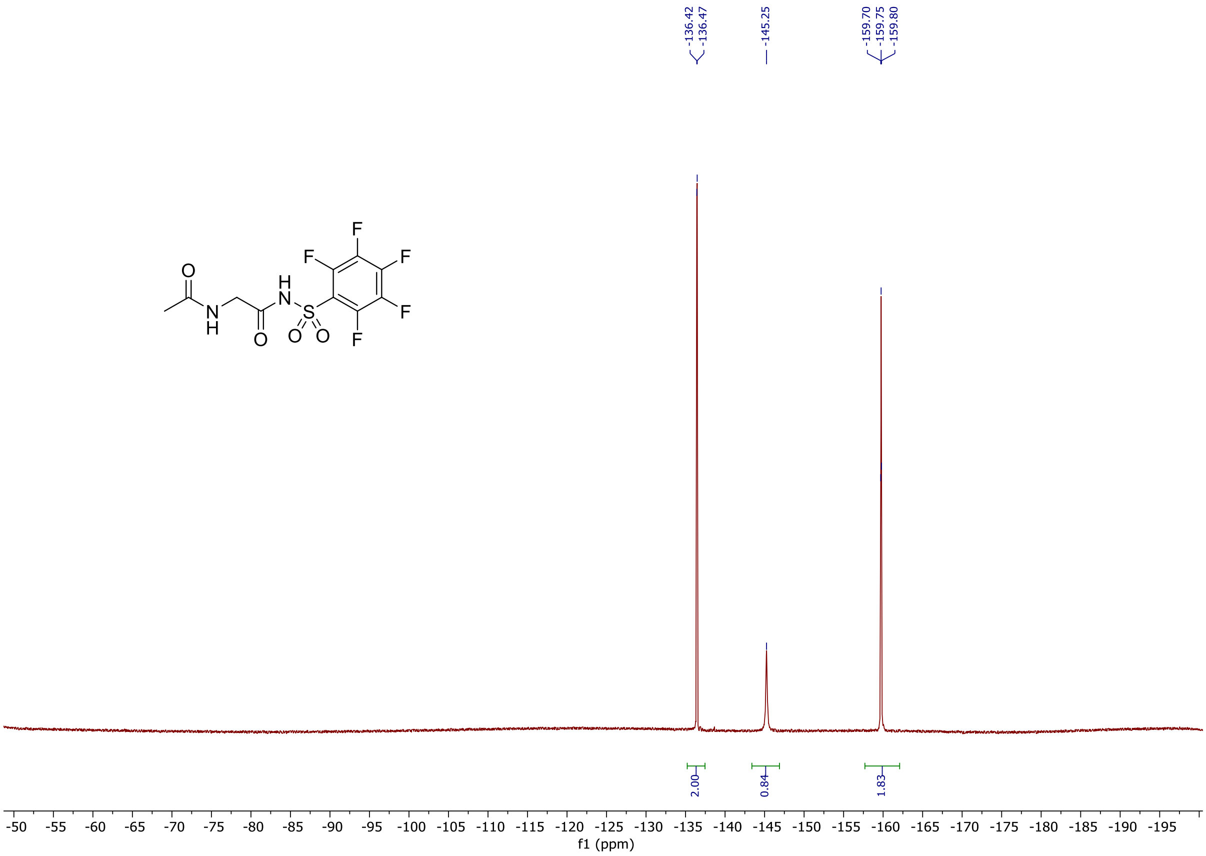

2,4,6-trimethoxy-N-(2-oxo-2-(perfluorophenylsulfonamido)ethyl)benzamide (BL13):

^{1}H NMR spectrum (500 MHz) in DMSO-d$_6$

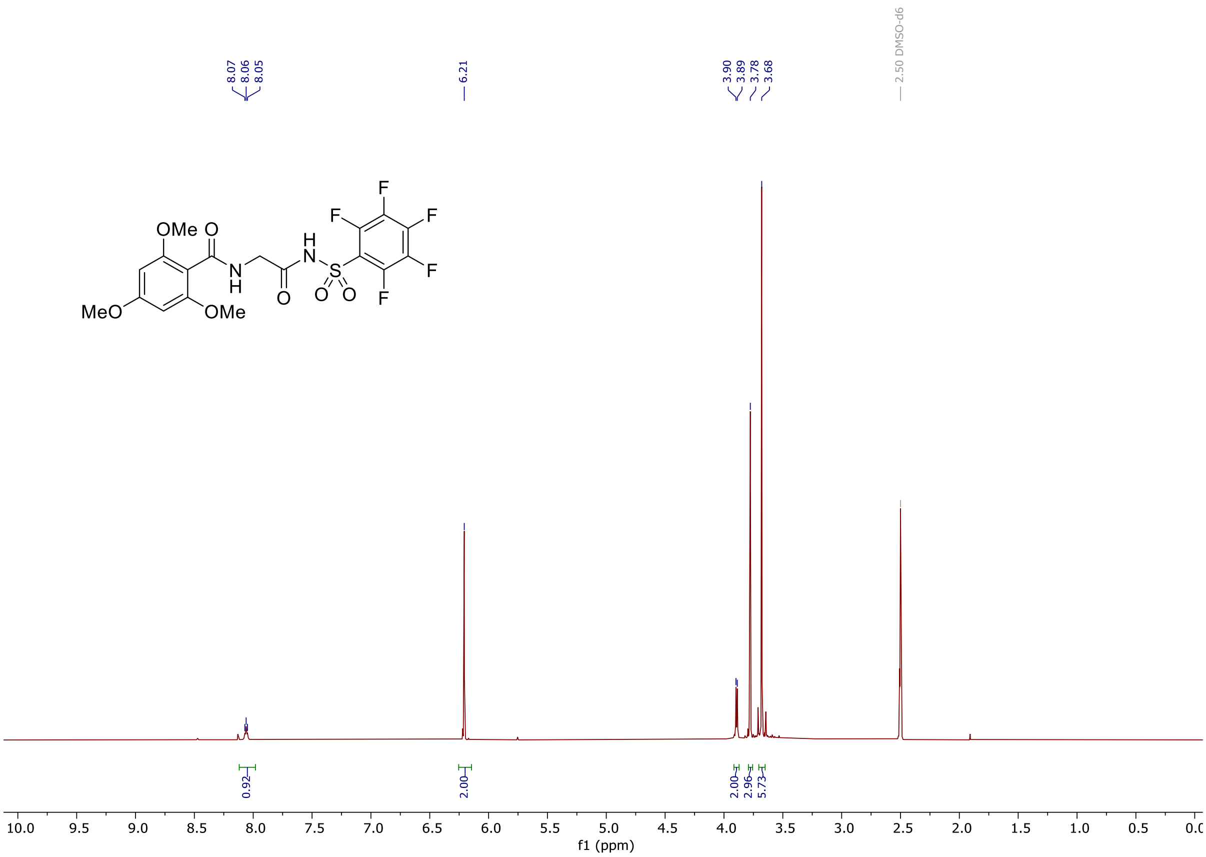

^{13}C NMR spectrum (126 MHz) in DMSO-d$_6$

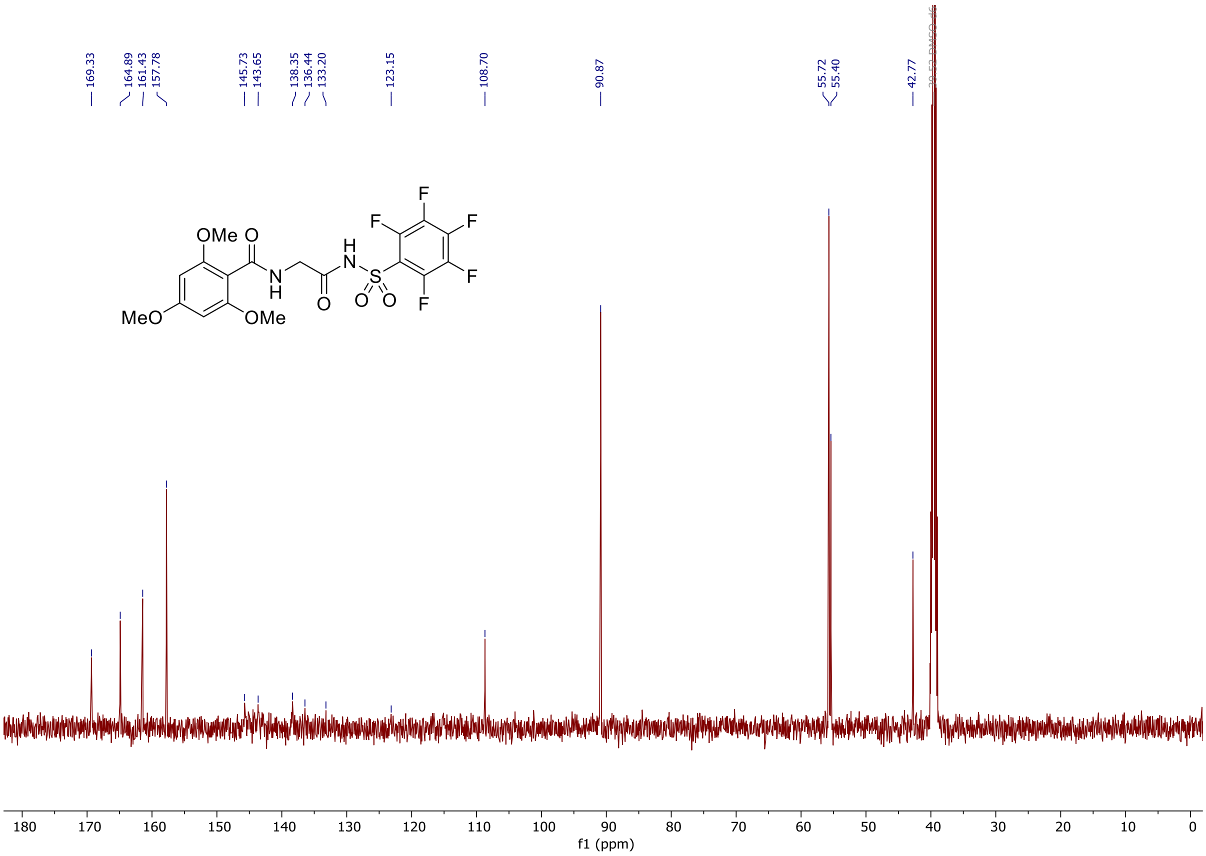

^{19}F NMR spectrum (471 MHz) in DMSO-d_6

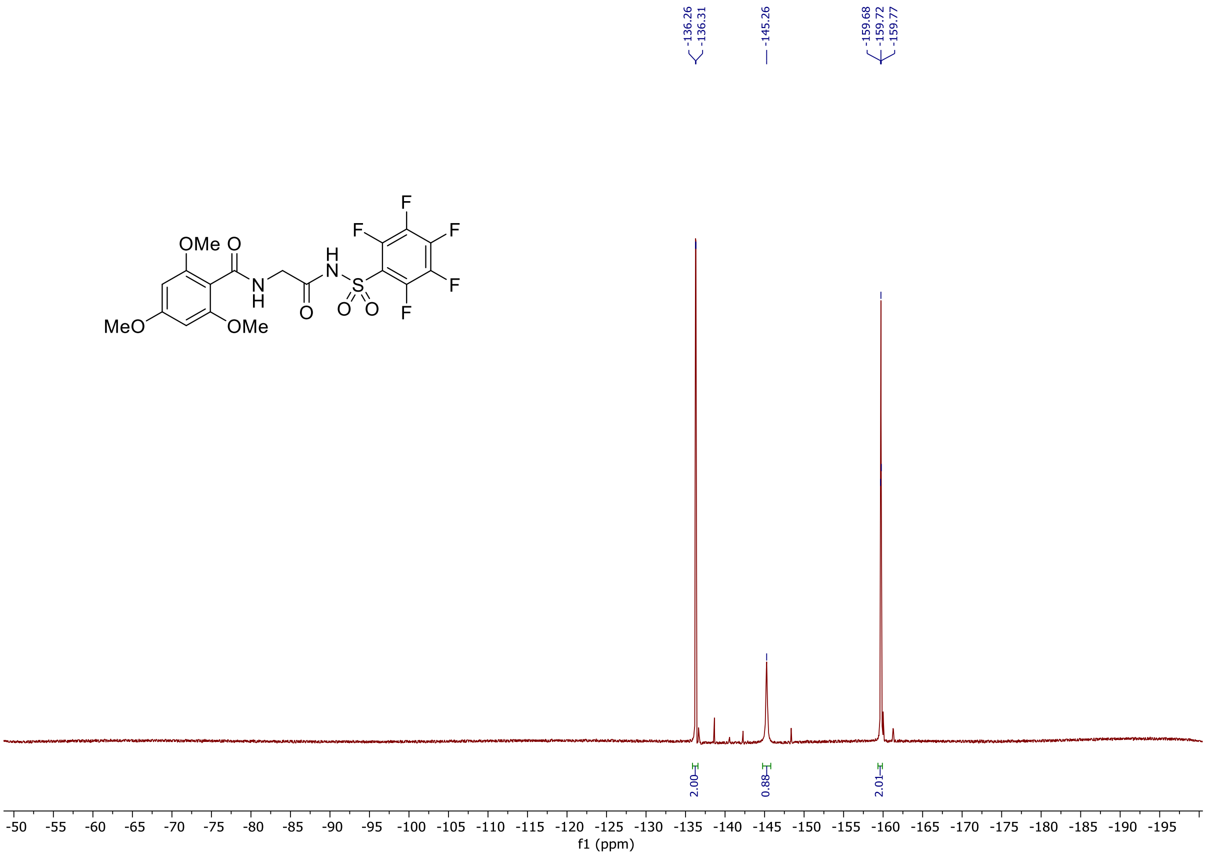

(*E*)-ethyl 3-(3-methyl-5-((triisopropylsilyl)oxy)phenyl)acrylate (2d):

^{1}H NMR spectrum (600 MHz) in $CDCl_3$

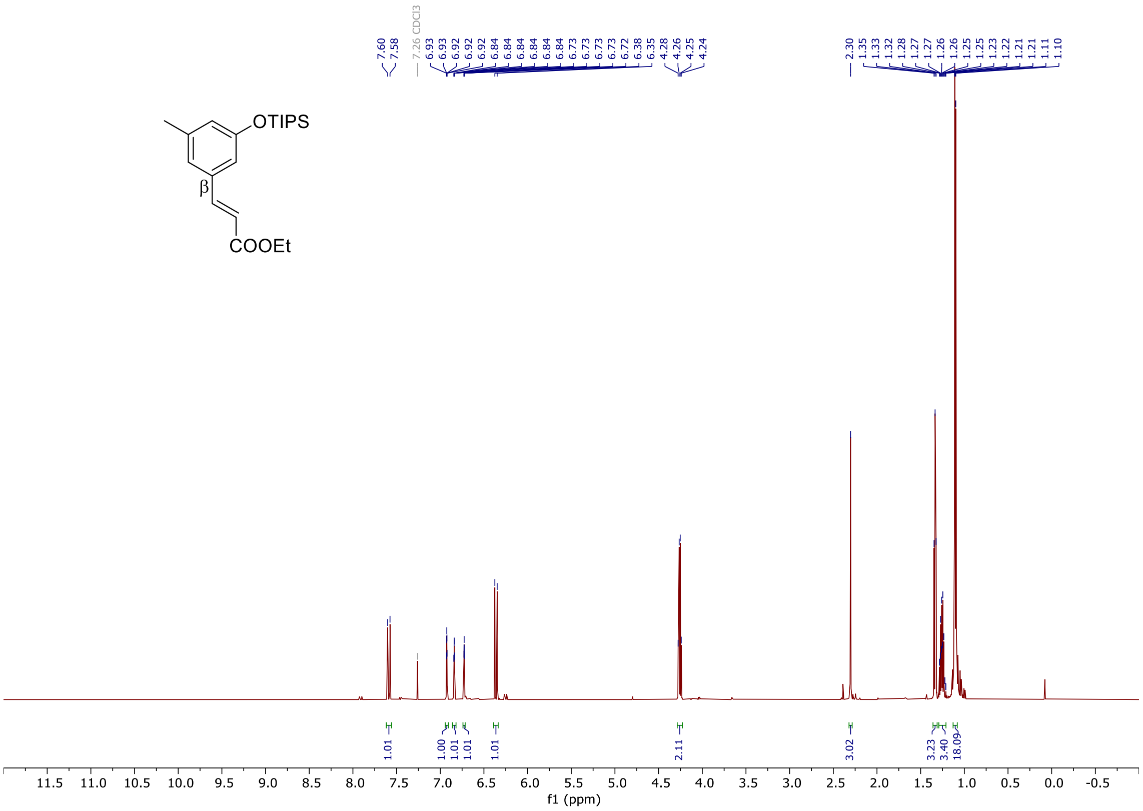

^{13}C NMR spectrum (151 MHz) in $CDCl_3$

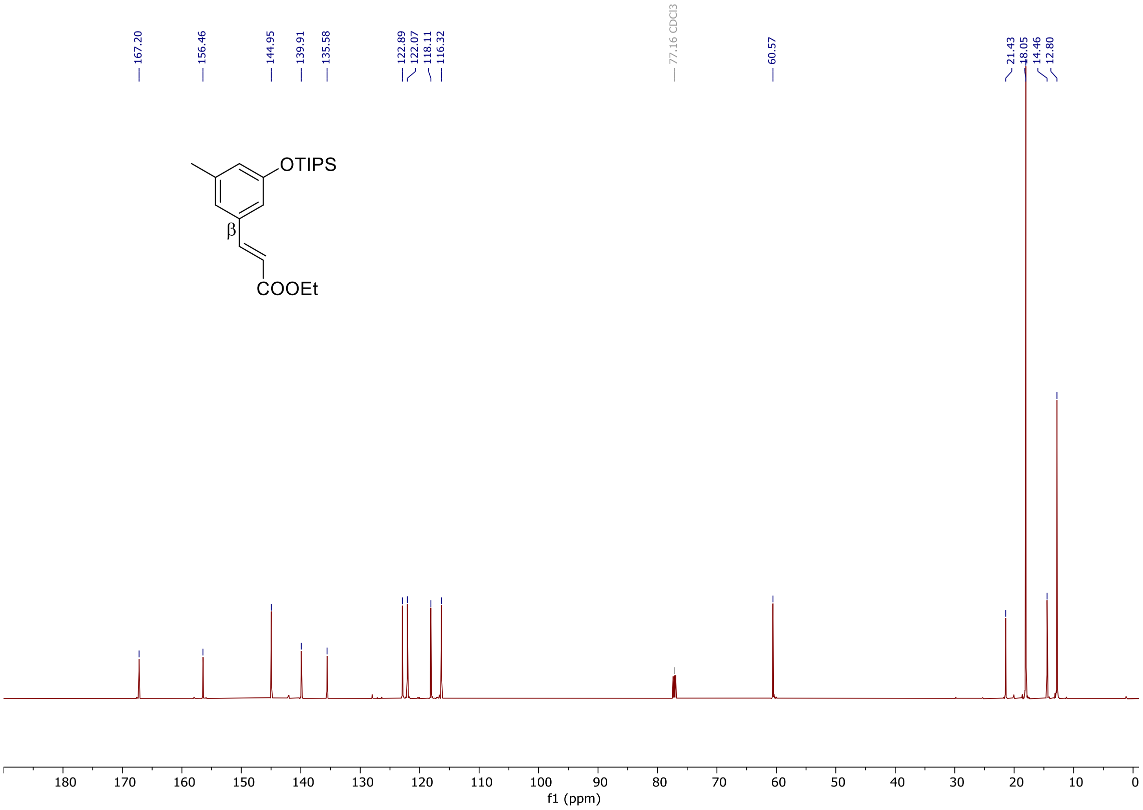

(*E*)-ethyl 3-(4-acetyl-3,5-dimethylphenyl)acrylate (2e):

^{1}H NMR spectrum (600 MHz) in $CDCl_3$

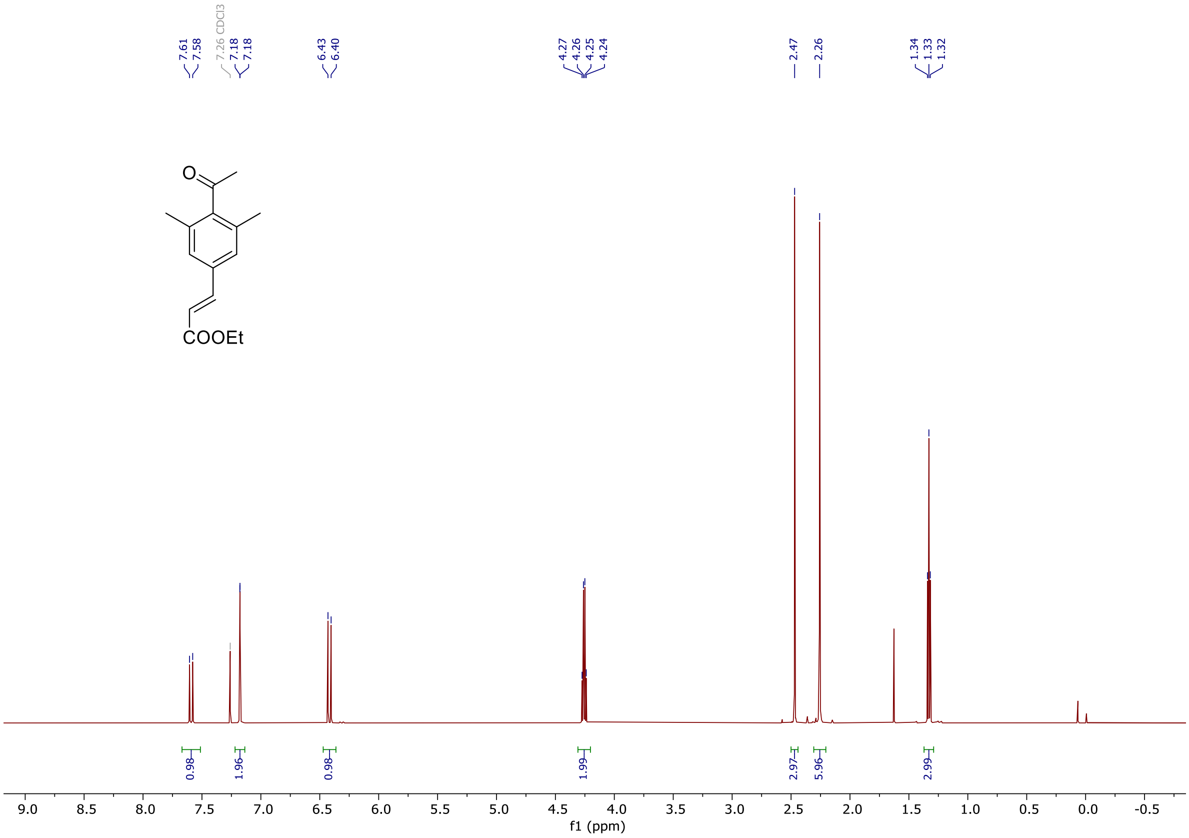

^{13}C NMR spectrum (151 MHz) in $CDCl_3$

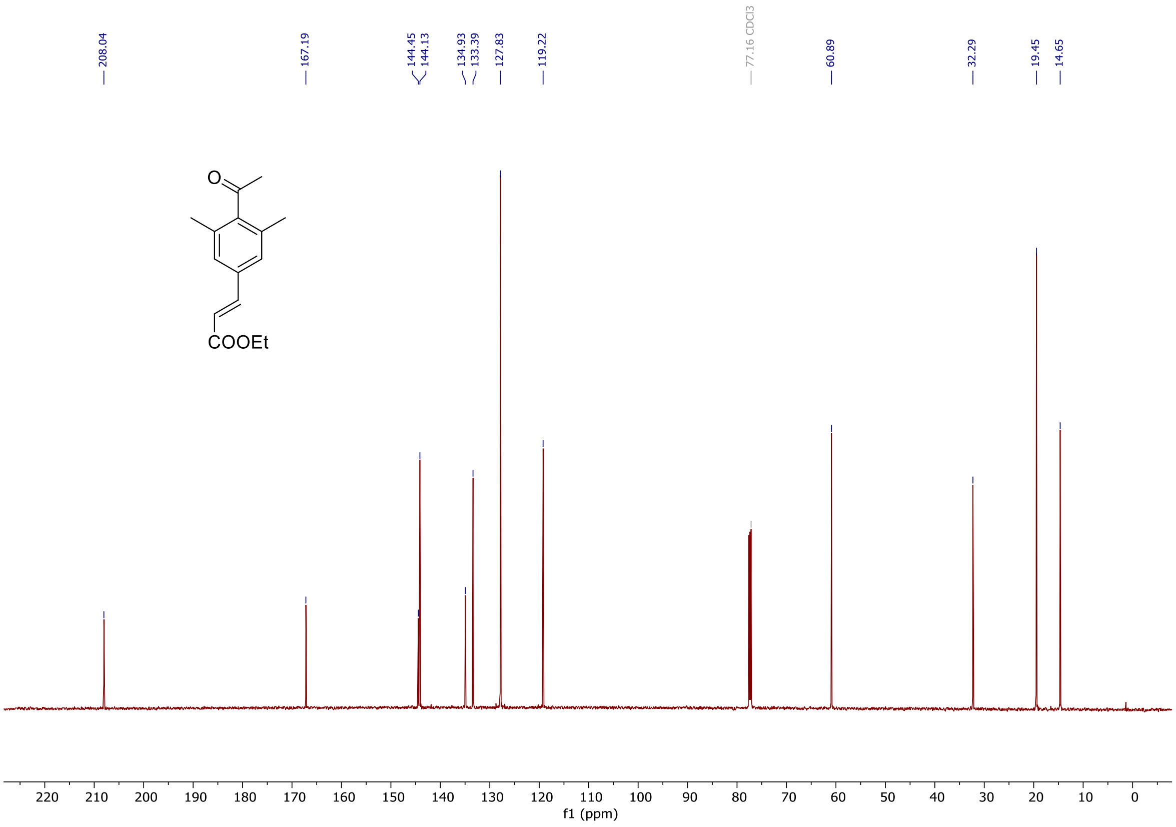

(*E*)-ethyl 3-(2-chloro-3-methoxy-5-methylphenyl)acrylate (2g):

^{1}H NMR spectrum (600 MHz) in $CDCl_3$

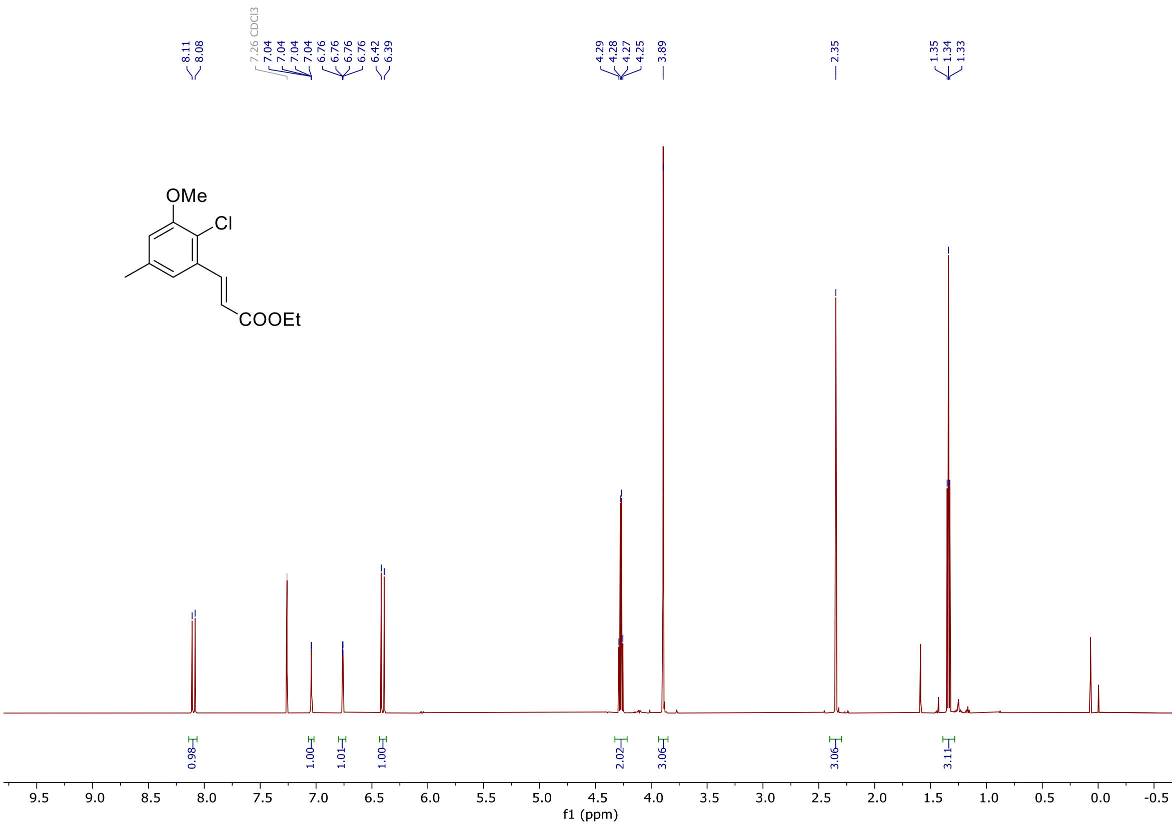

^{13}C NMR spectrum (151 MHz) in $CDCl_3$

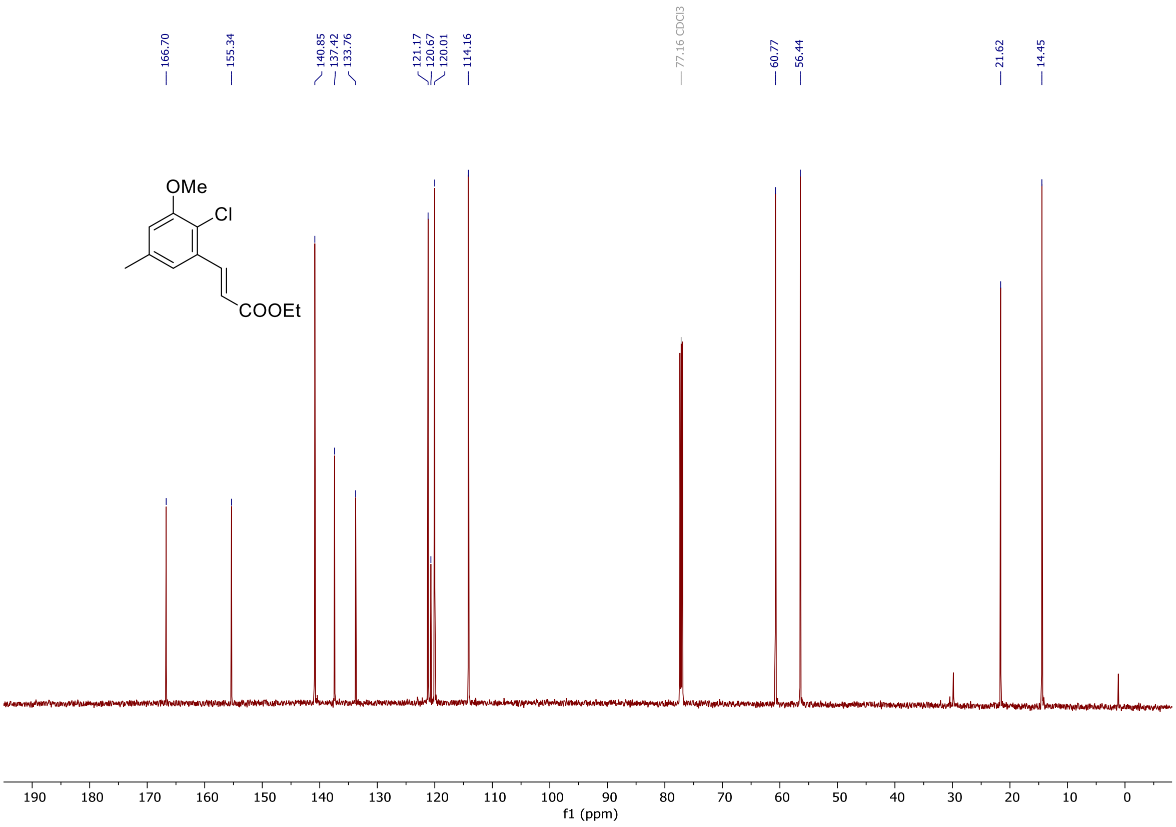

(*E*)-ethyl 3-(2'-fluoro-4'-(1-methoxy-1-oxopropan-2-yl)-[1,1'-biphenyl]-3-yl)acrylate (2k-β) and (*E*)-ethyl 3-(2'-fluoro-4'-(1-methoxy-1-oxopropan-2-yl)-[1,1'-biphenyl]-4-yl)acrylate (2k-γ):

^{1}H NMR spectrum (600 MHz) in $CDCl_3$

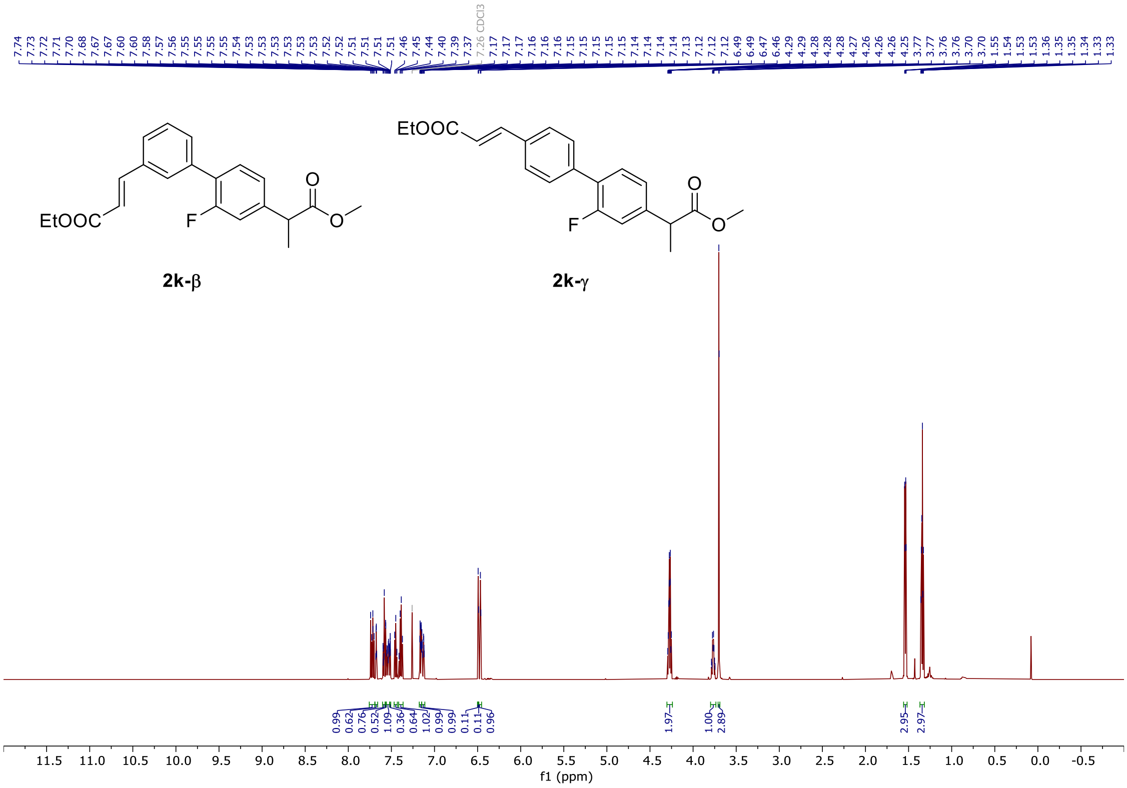

^{13}C NMR spectrum (151 MHz) in $CDCl_3$

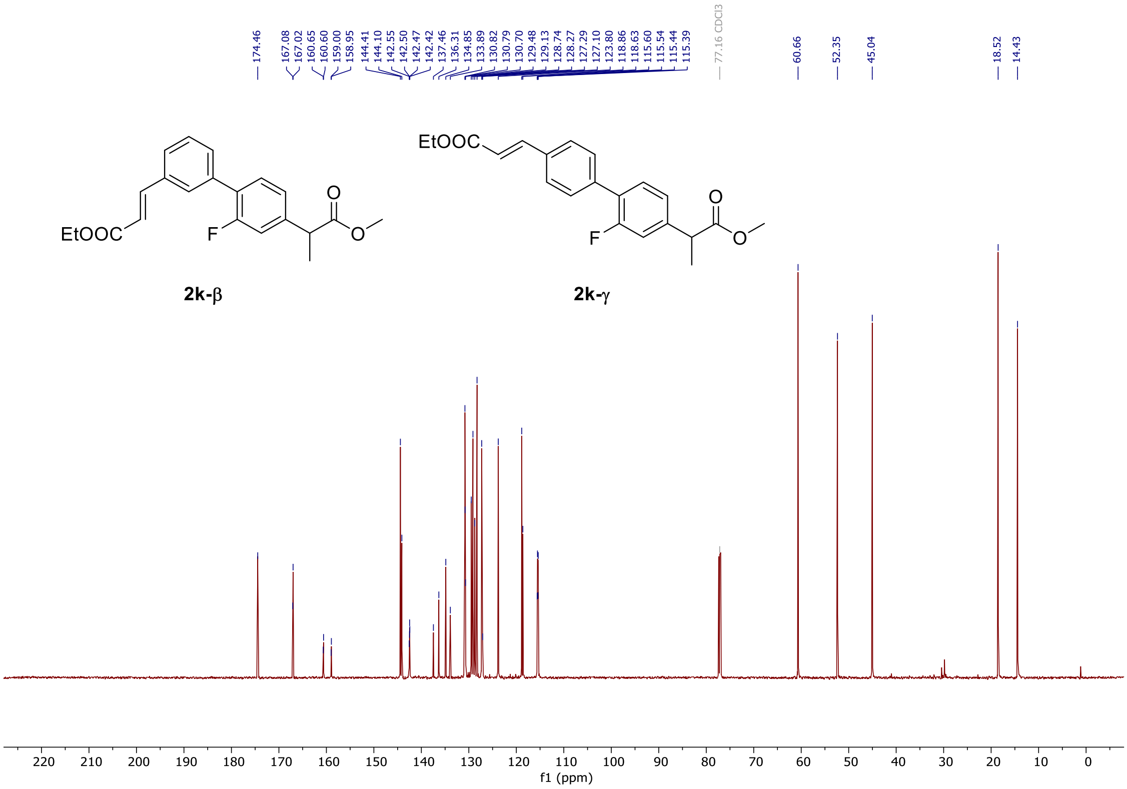

^{19}F NMR spectrum (471 MHz) in $CDCl_3$

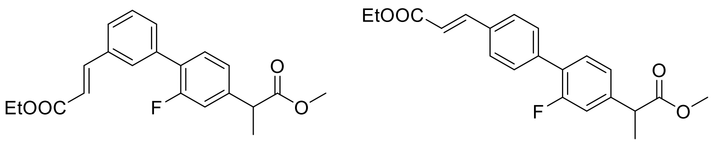

2k-β **2k-γ**

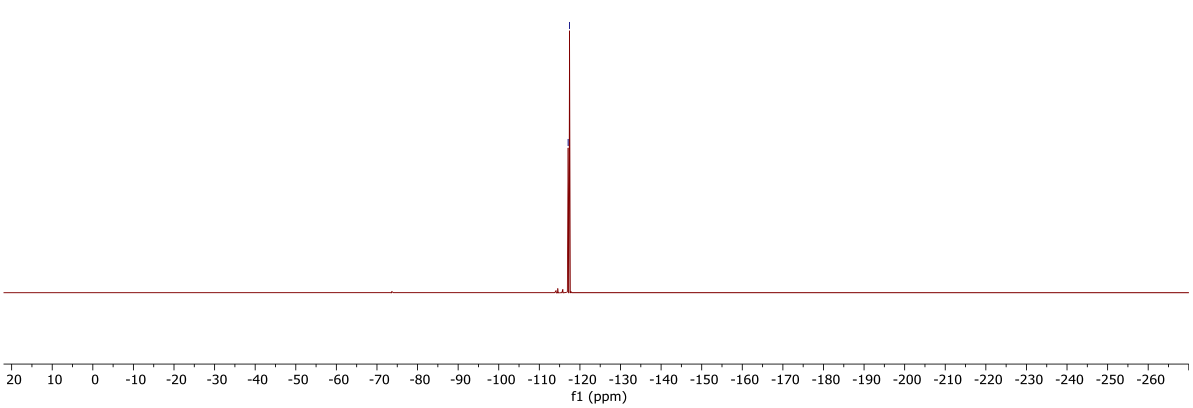

(*E*)-ethyl 3-(3-(3-(1-methoxy-1-oxopropan-2-yl)benzoyl)phenyl)acrylate (2l-β), (*E*)-ethyl 3-(4-(3-(1-methoxy-1-oxopropan-2-yl)benzoyl)phenyl)acrylate (2l-γ) and (*E*)-ethyl 3-(3-benzoyl-5-(1-methoxy-1-oxopropan-2-yl)phenyl)acrylate (2l-β') :

^1H NMR spectrum (600 MHz) in C_6D_6

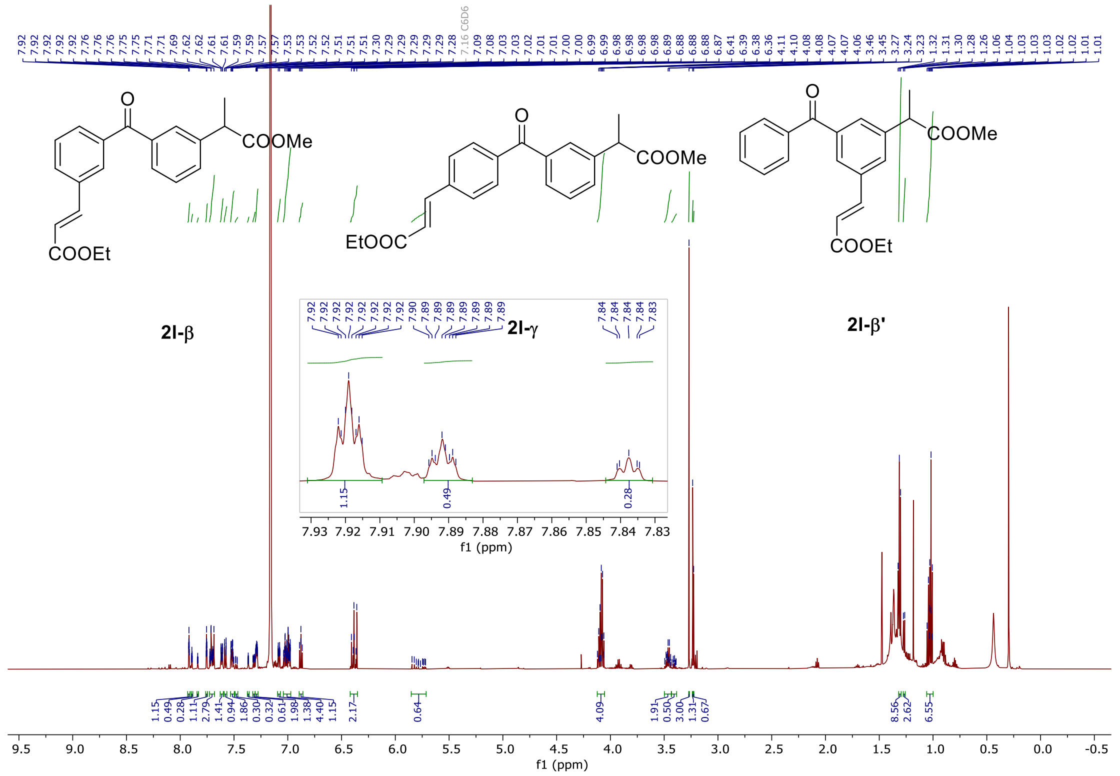

^{13}C NMR spectrum (151 MHz) in C_6D_6

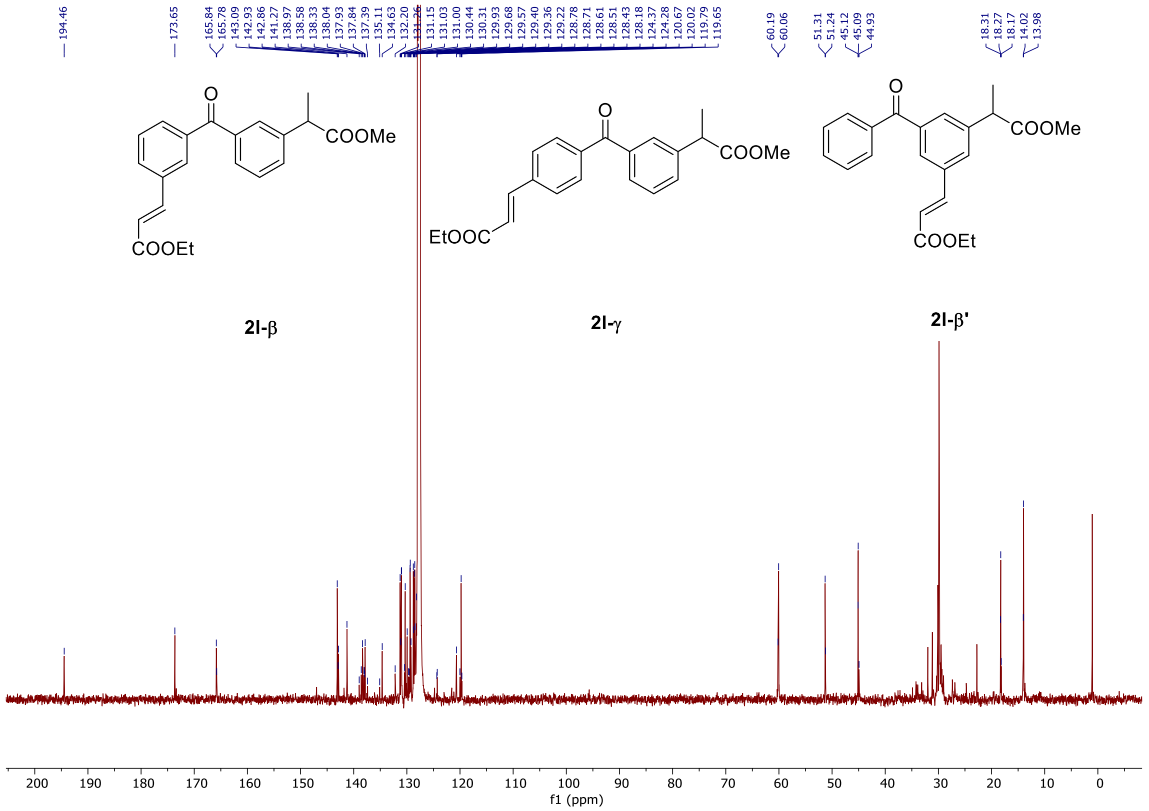

(*E*)-ethyl 3-(2-(3-(1-methoxy-1-oxopropan-2-yl)benzoyl)phenyl)acrylate (2l-α) and (*E*)-ethyl 3-(4-benzoyl-2-(1-methoxy-1-oxopropan-2-yl)phenyl)acrylate (2l-α') :

^{1}H NMR spectrum (600 MHz) in C_6D_6

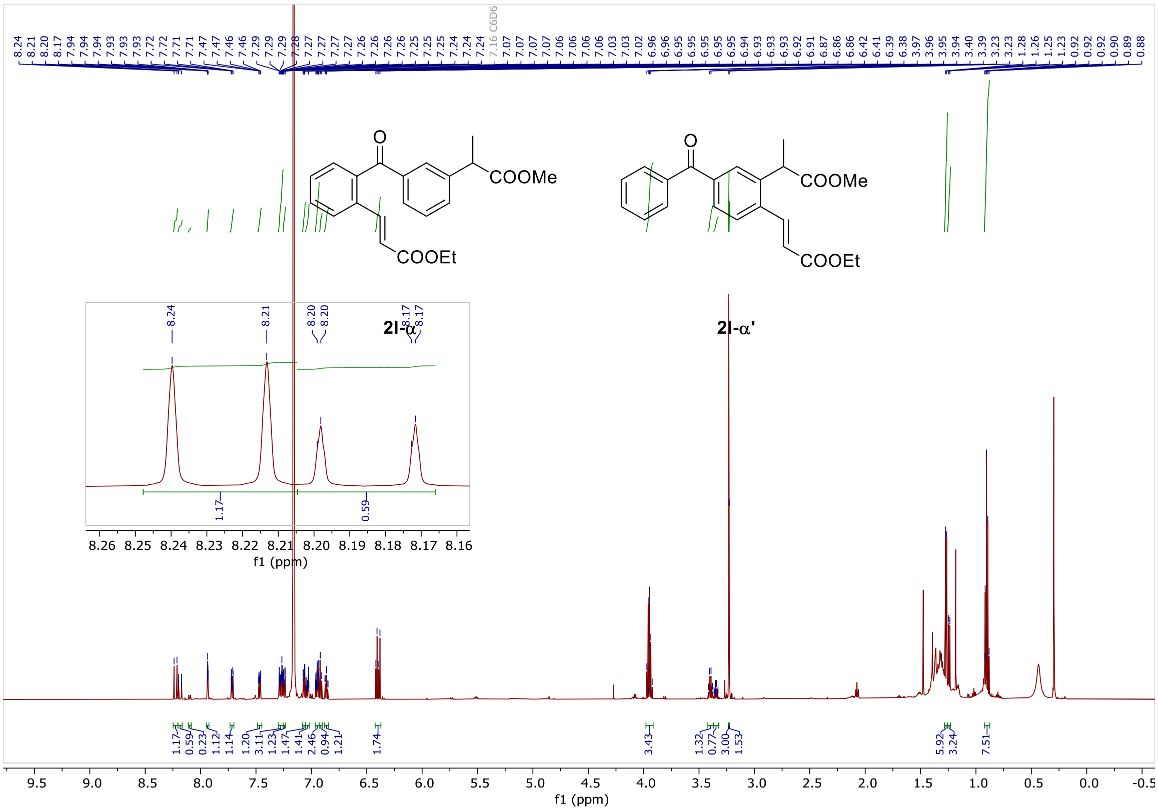

^{13}C NMR spectrum (151 MHz) in C_6D_6

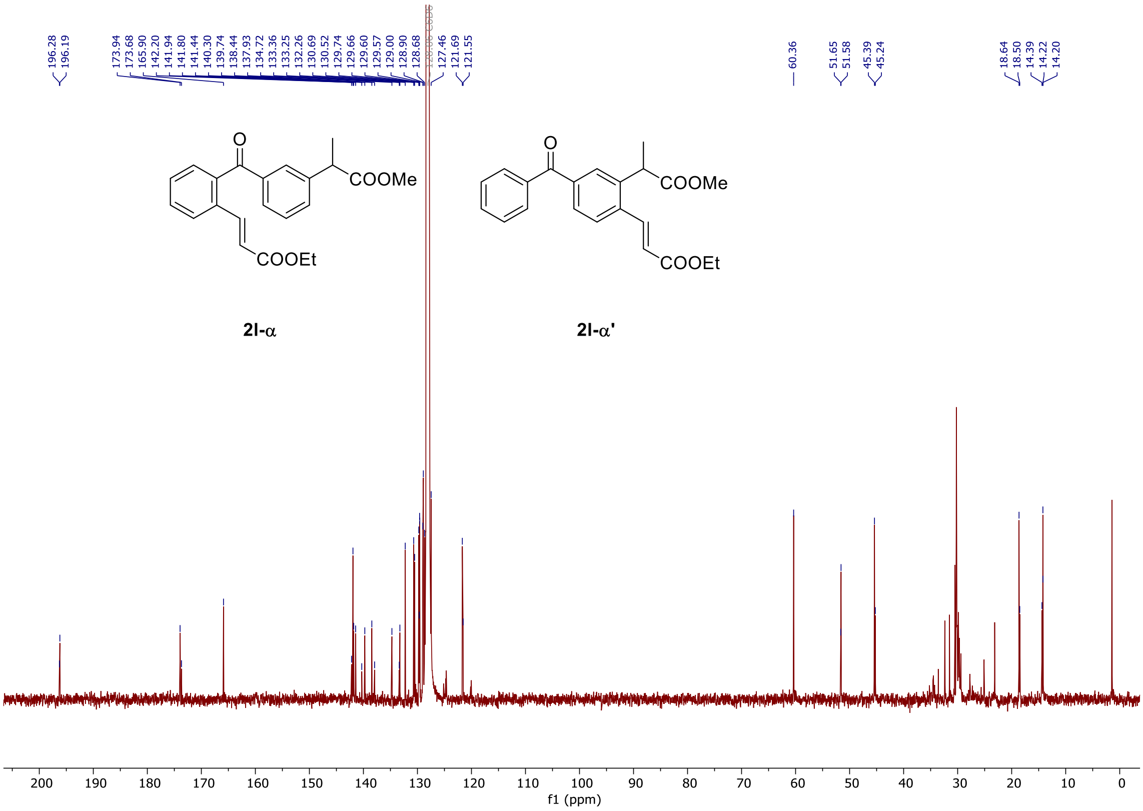

(*E*)-ethyl 3-(3-(3-methoxy-3-oxopropyl)-5-methylphenyl)acrylate (2m):

^{1}H NMR spectrum (600 MHz) in $CDCl_3$

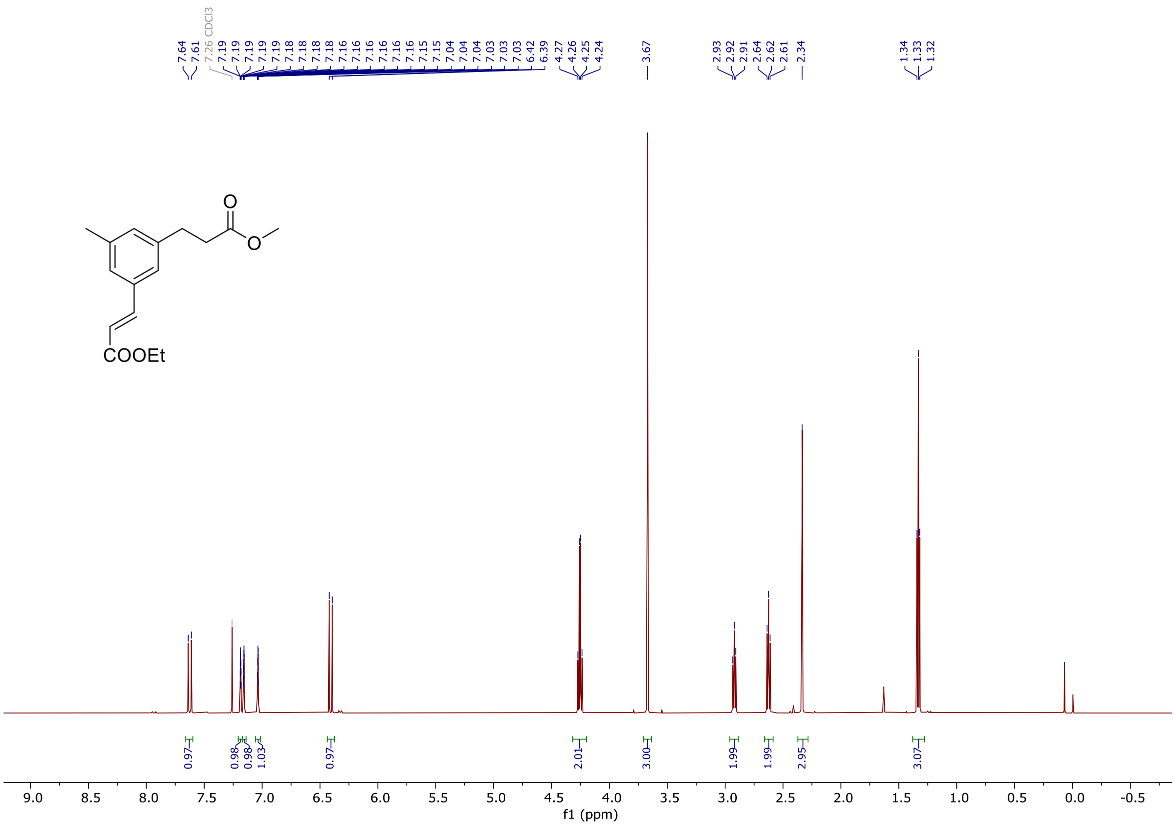

^{13}C NMR spectrum (151 MHz) in $CDCl_3$

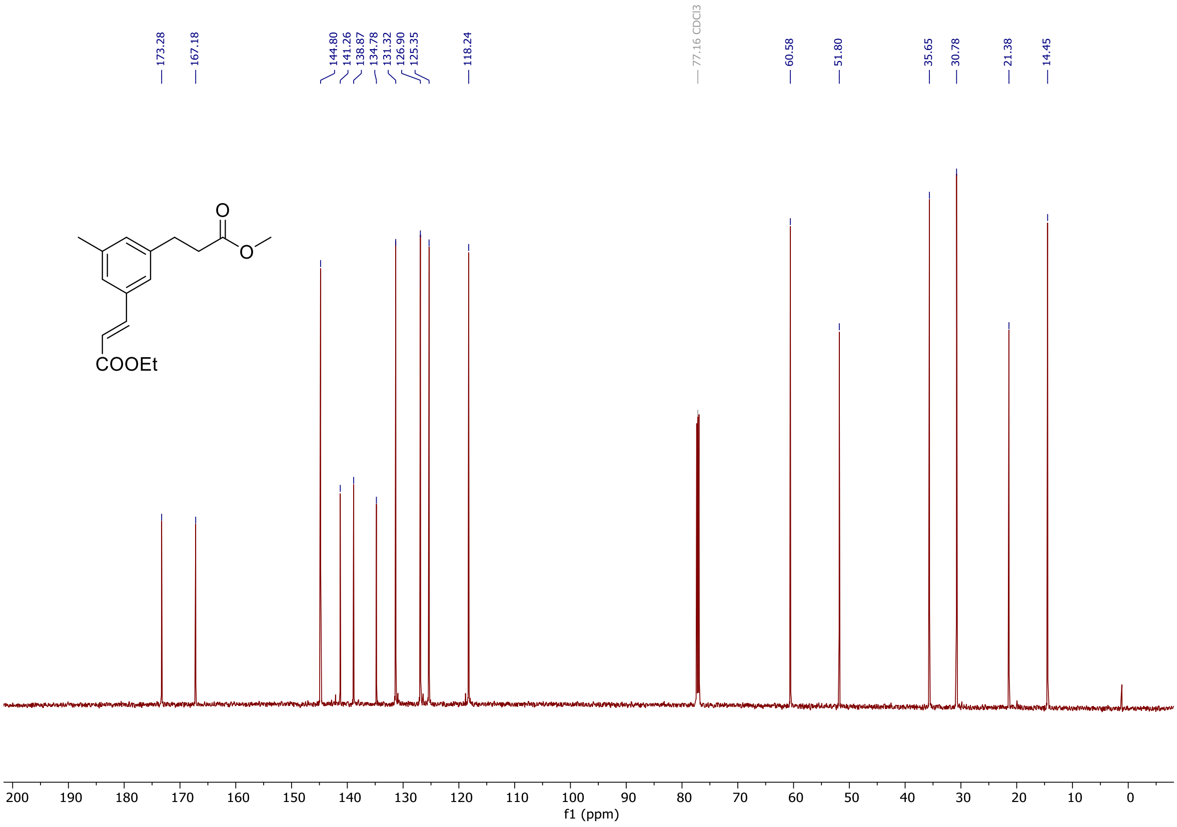

(*E*)-ethyl 3-(3-((2-isopropyl-5-methylphenoxy)methyl)-5-methylphenyl)acrylate (2o-β) and (*E*)-ethyl 3-(2-((2-isopropyl-5-methylphenoxy)methyl)-4-methylphenyl)acrylate (2o-α):

^{1}H NMR spectrum (600 MHz) in $CDCl_3$

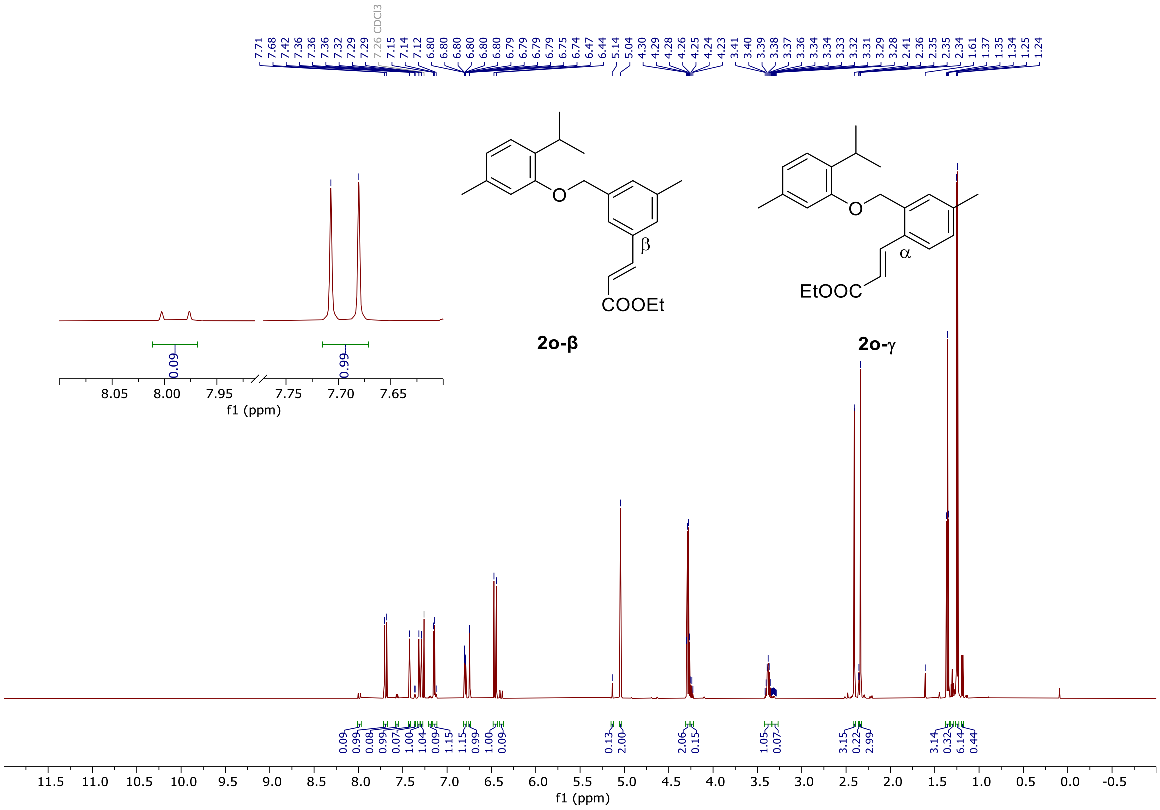

^{13}C NMR spectrum (151 MHz) in $CDCl_3$

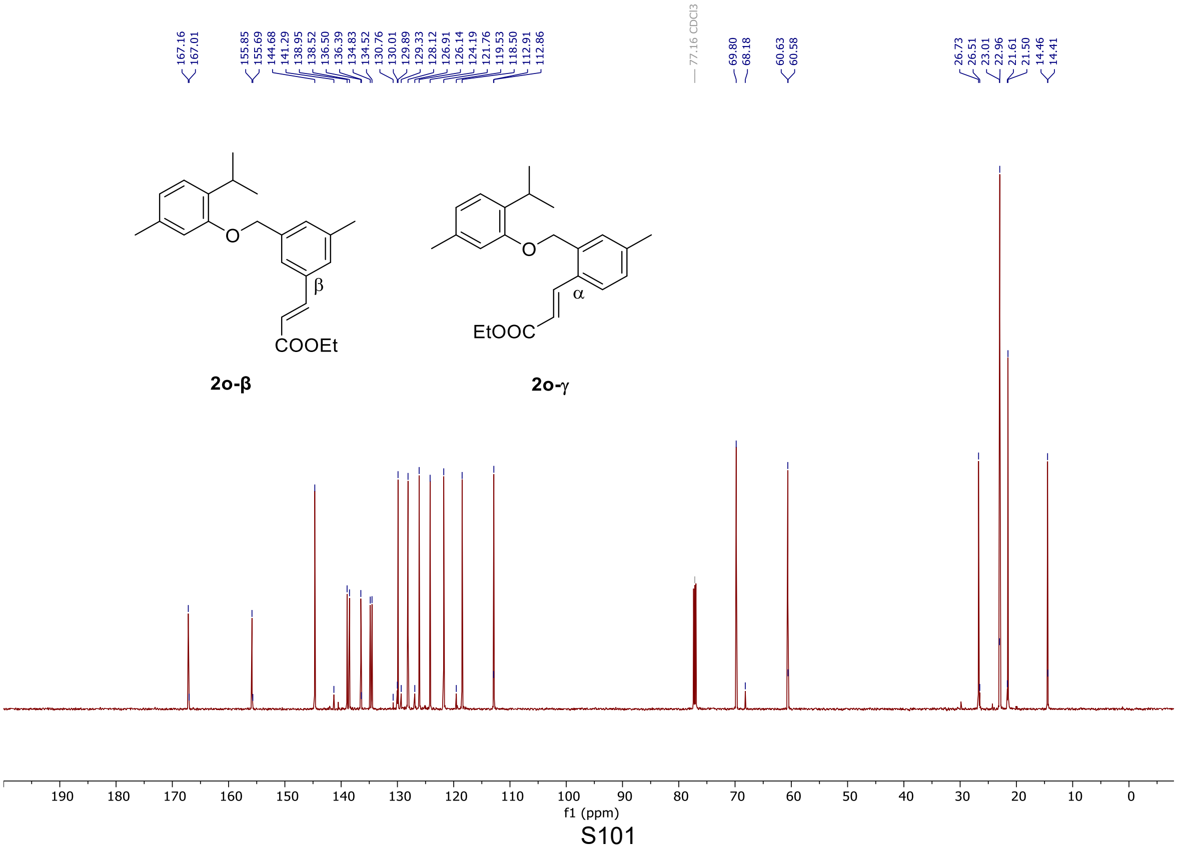

3-(*tert*-butyl)benzoic acid (3a-*meta*) and 4-(*tert*-butyl)benzoic acid (3a-*para*):

^{1}H NMR spectrum (500 MHz) in $CDCl_3$

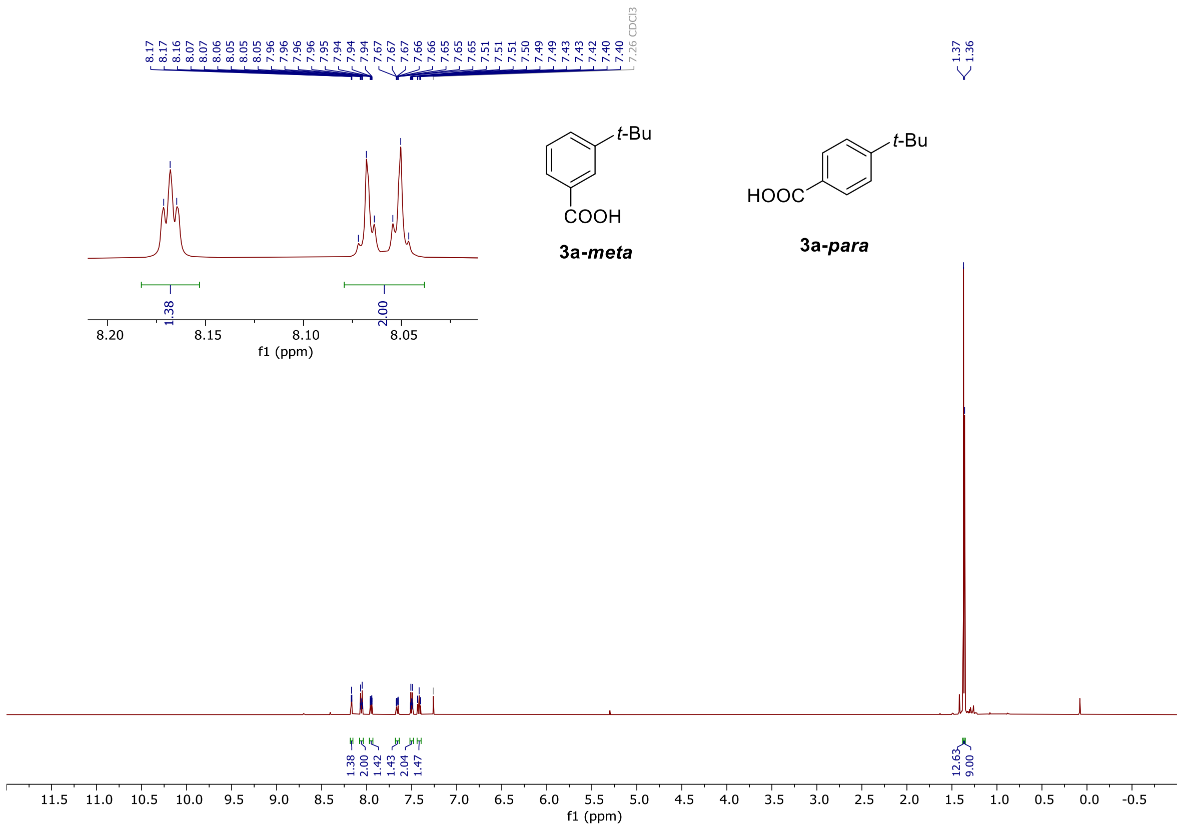

^{13}C NMR spectrum (126 MHz) in $CDCl_3$

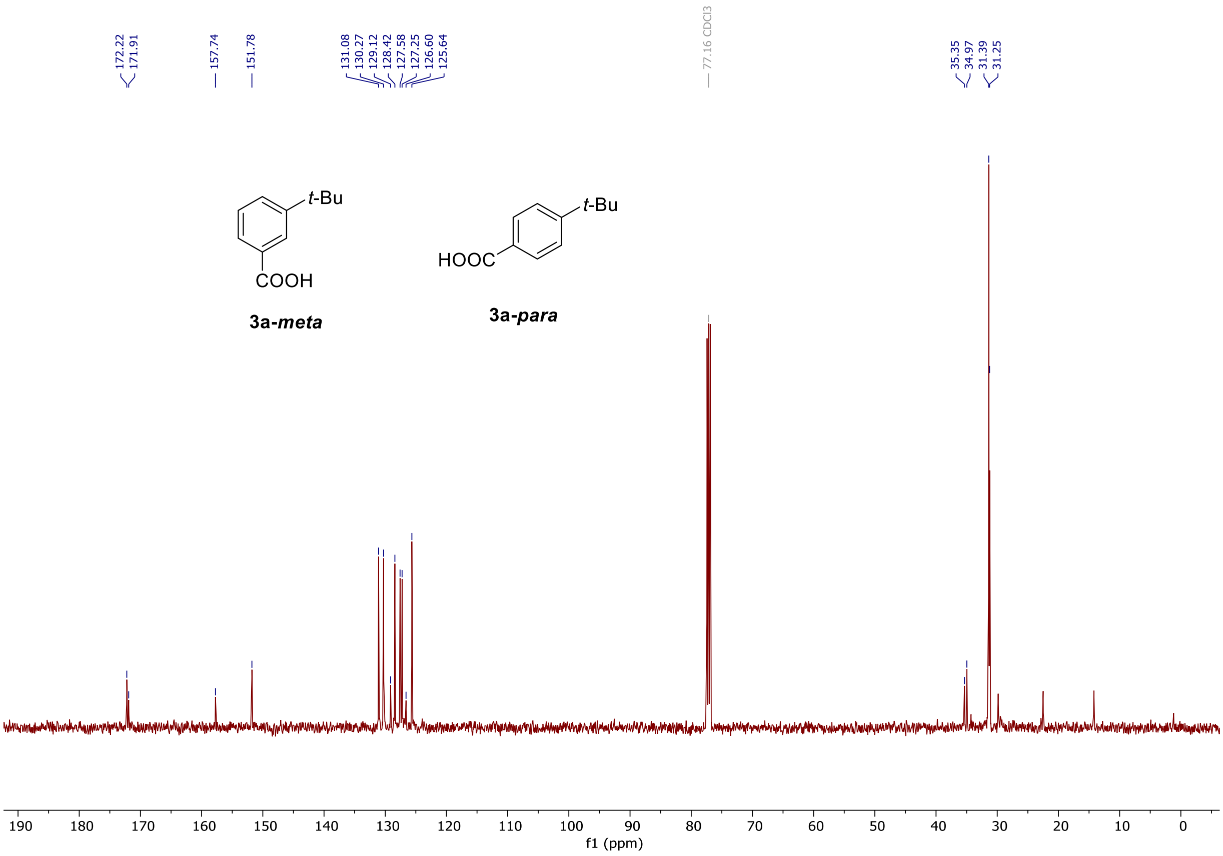

3,5-dimethylbenzoic acid (3b):

^{1}H NMR spectrum (500 MHz) in $CDCl_3$

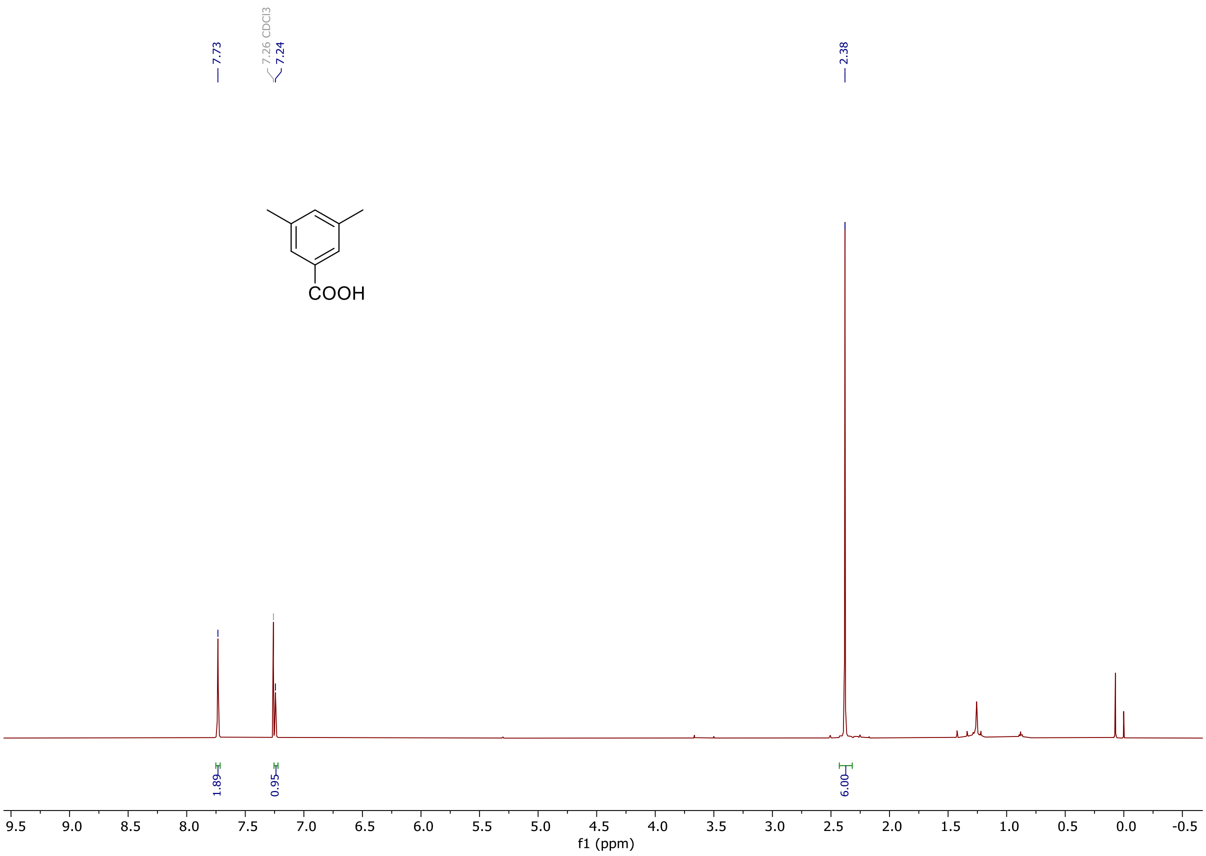

^{13}C NMR spectrum (126 MHz) in $CDCl_3$

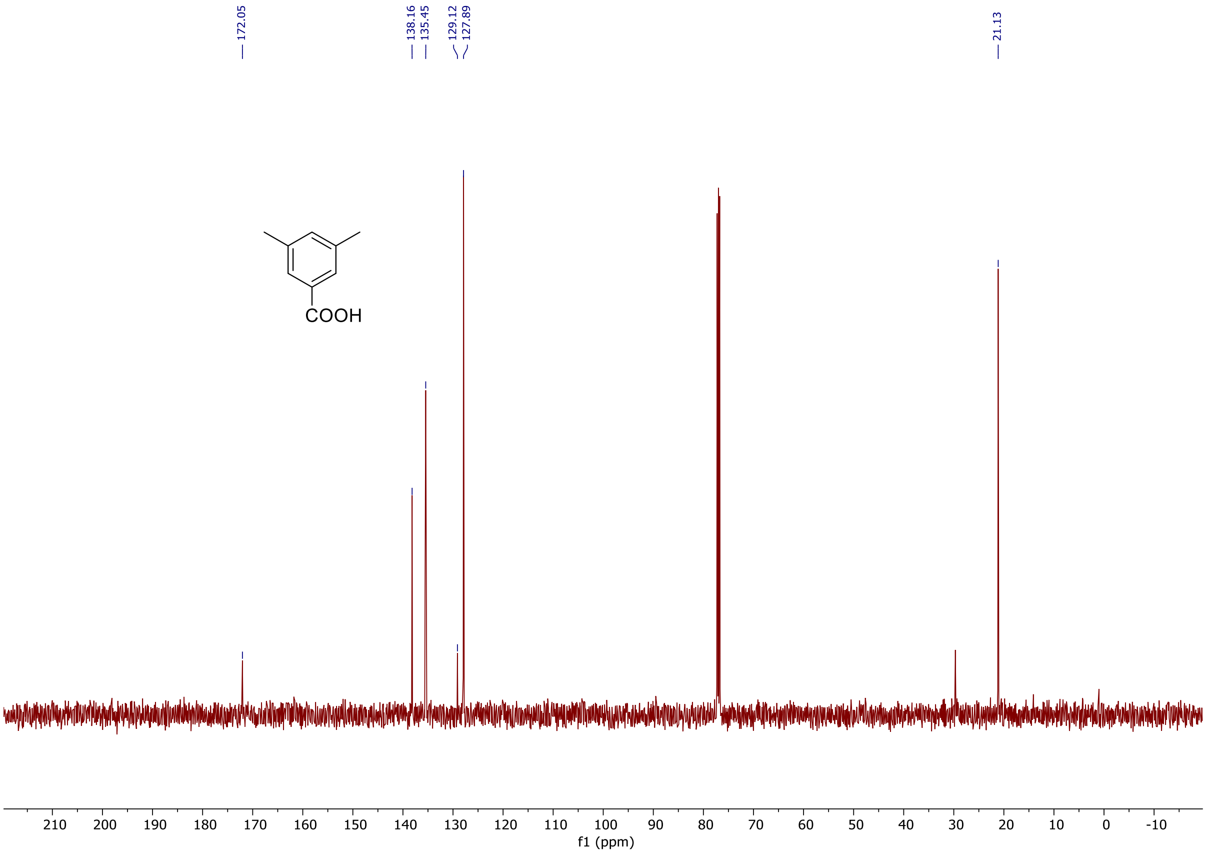

3-methoxy-5-methylbenzoic acid (3c)

^{1}H NMR spectrum (500 MHz) in $CDCl_3$

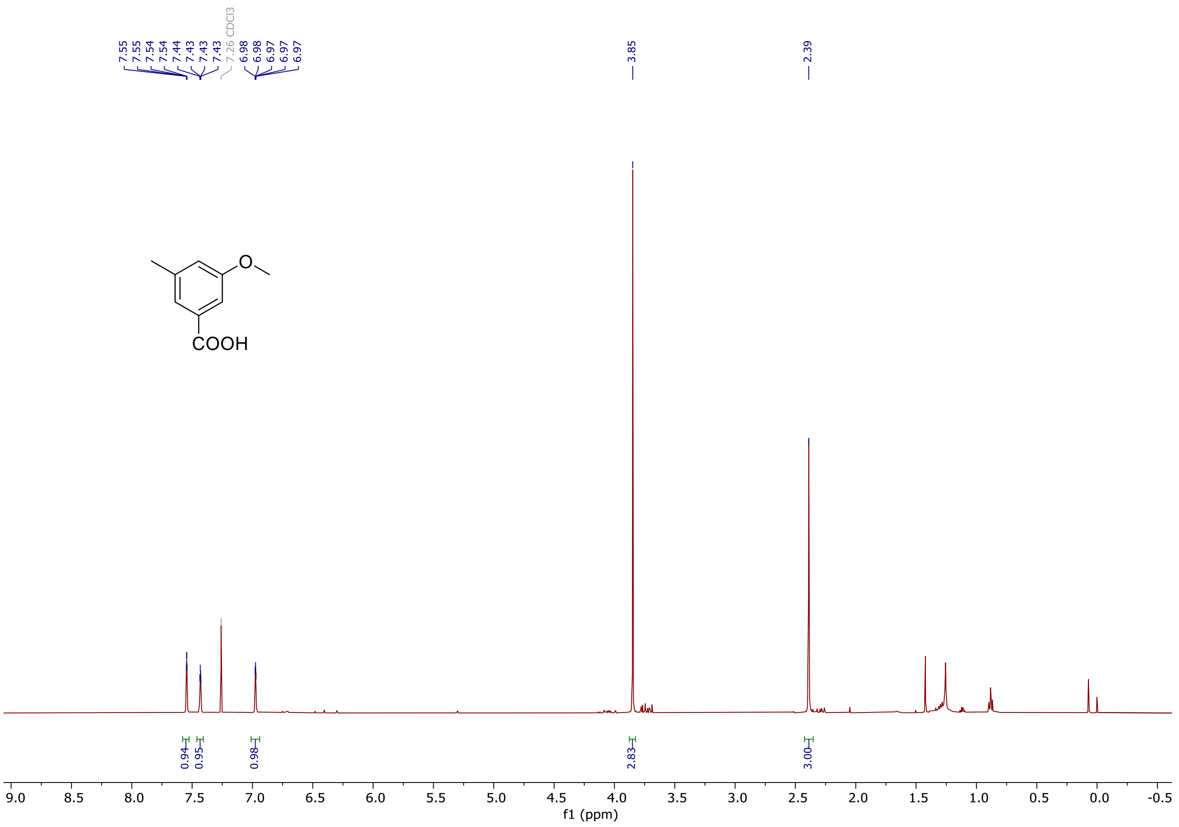

^{13}C NMR spectrum (126 MHz) in $CDCl_3$

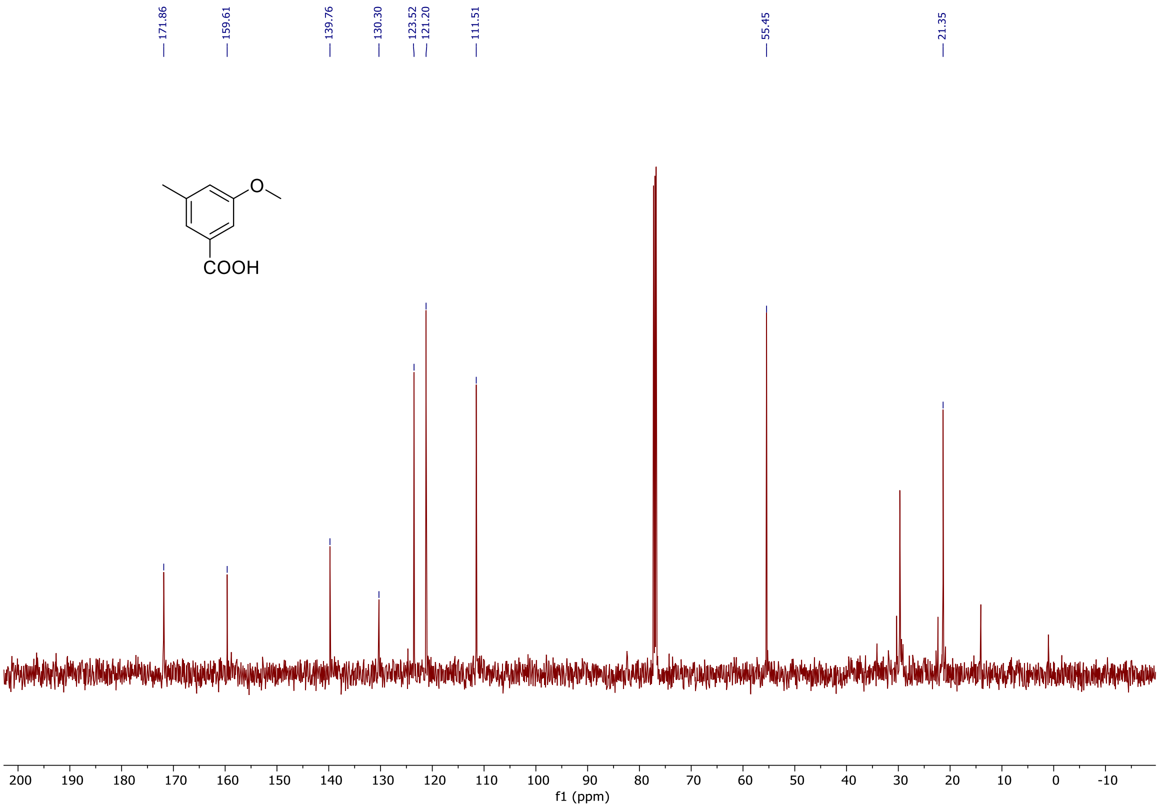

Methyl 3-methyl-5-((triisopropylsilyl)oxy)benzoate (3d):

^{1}H NMR spectrum (600 MHz) in $CDCl_3$

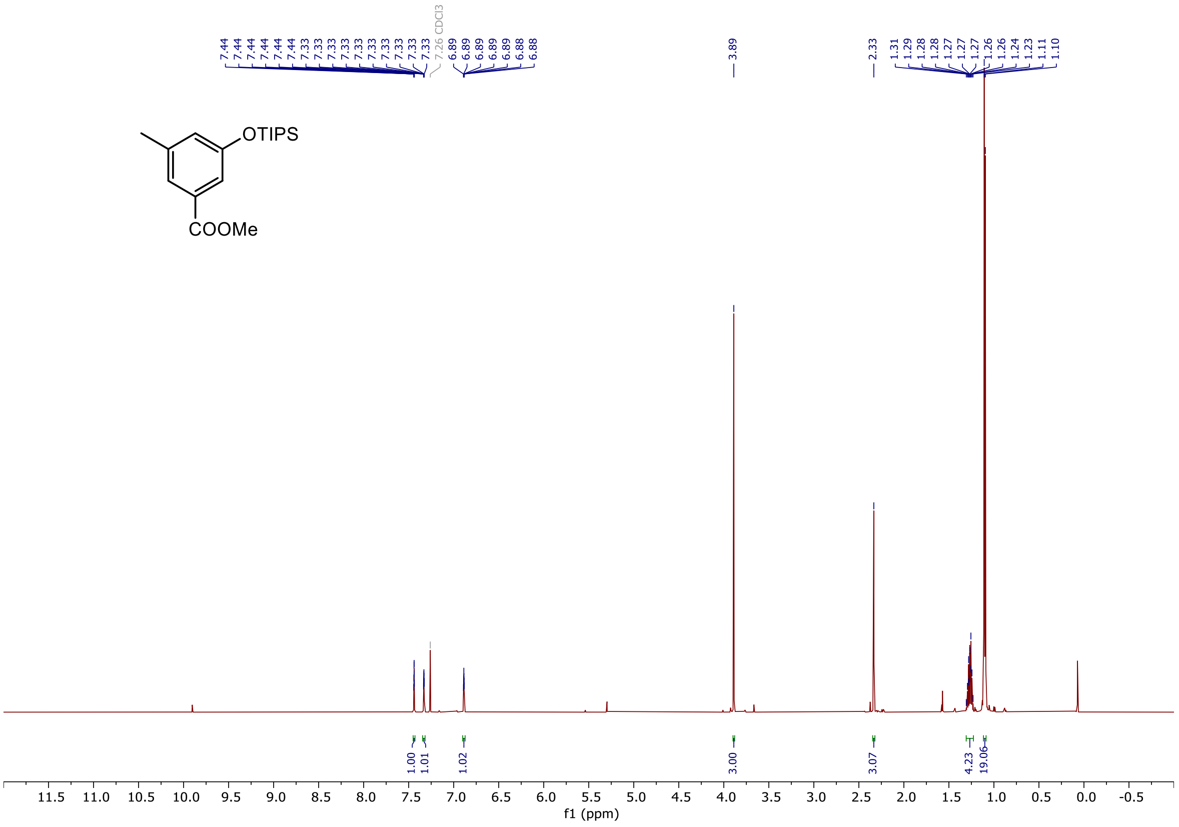

^{13}C NMR spectrum (151 MHz) in $CDCl_3$

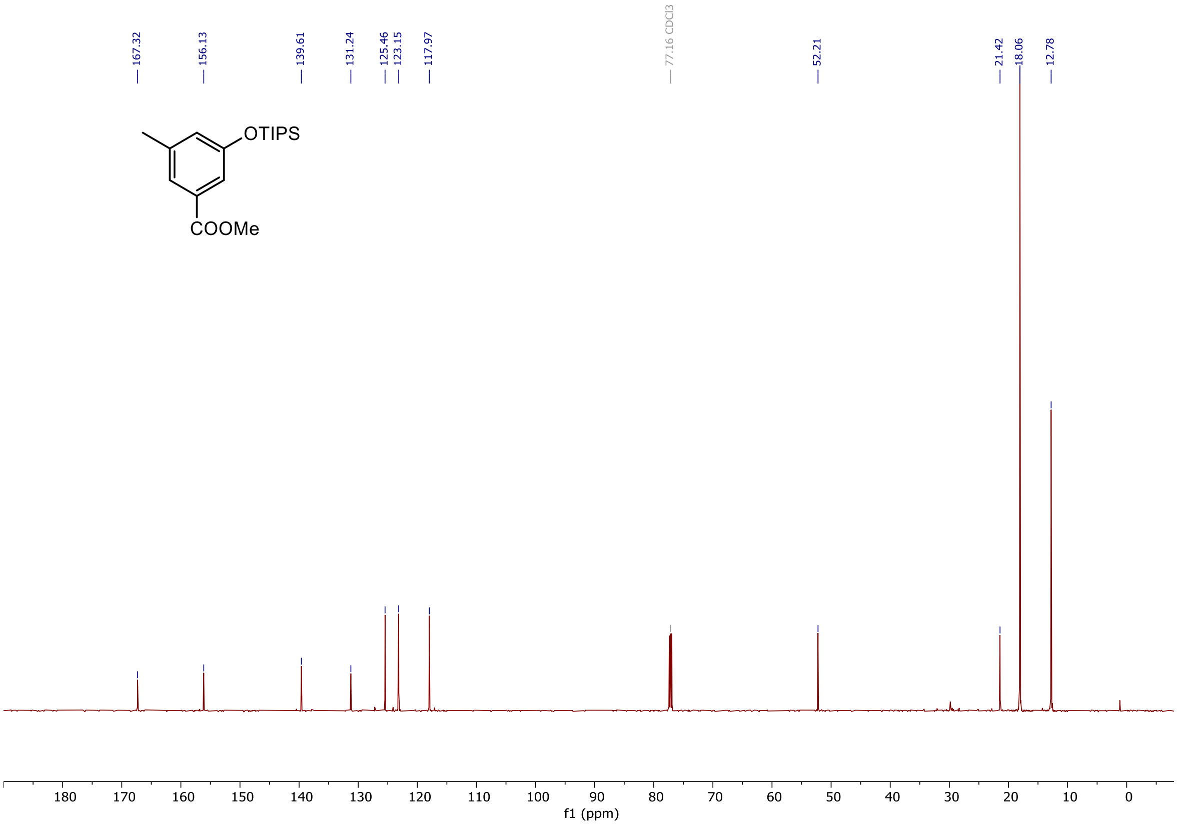

4-acetyl-3,5-dimethylbenzoic acid (3e):

^{1}H NMR spectrum (500 MHz) in $CDCl_3$

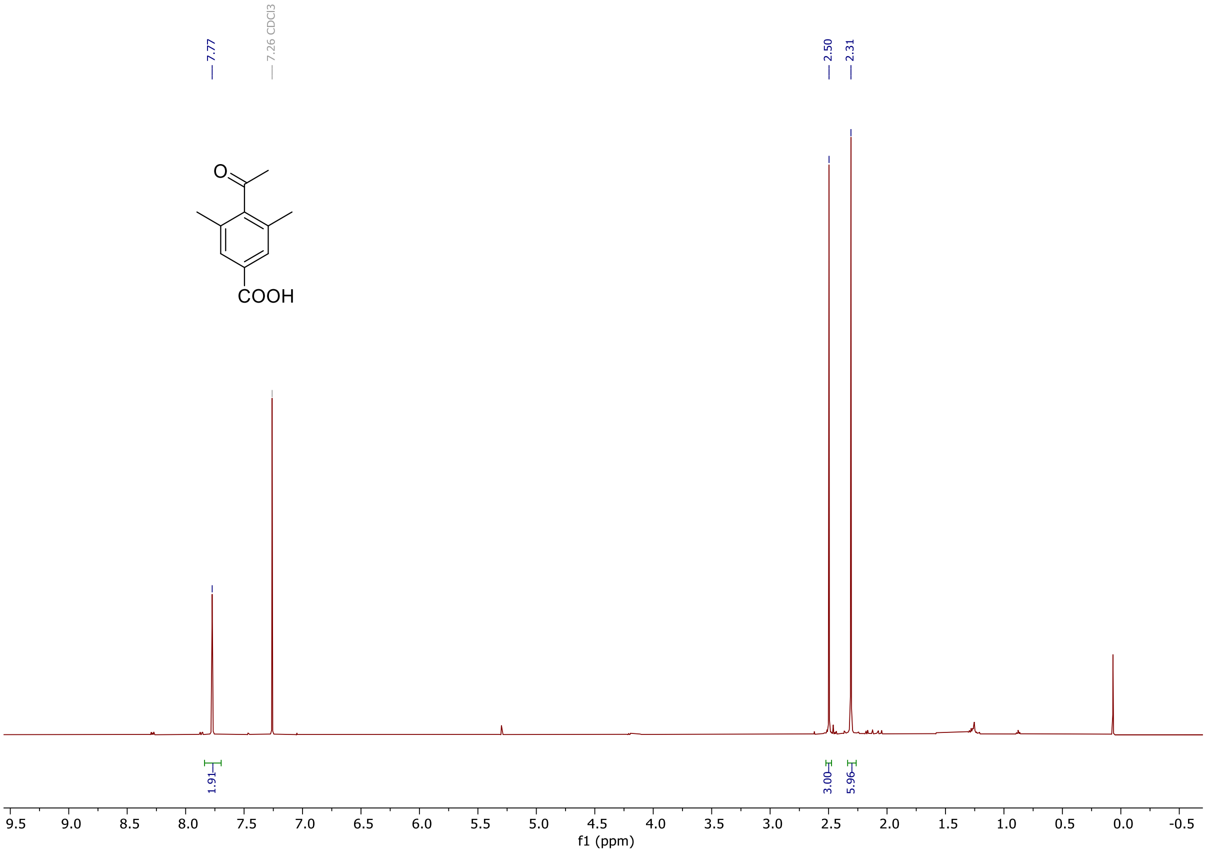

^{13}C NMR spectrum (126 MHz) in $CDCl_3$

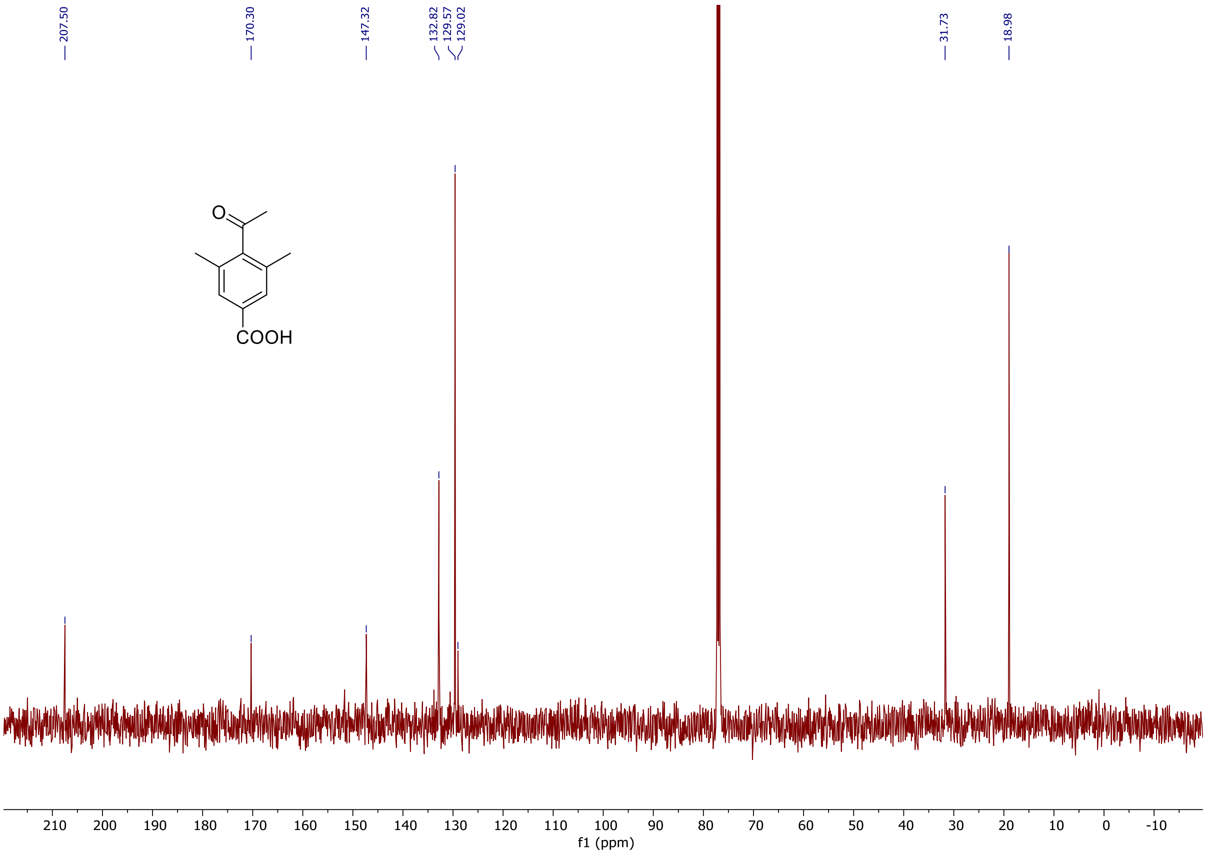

2-chloro-5-methylbenzoic acid (3f):

^1H NMR spectrum (500 MHz) in $CDCl_3$

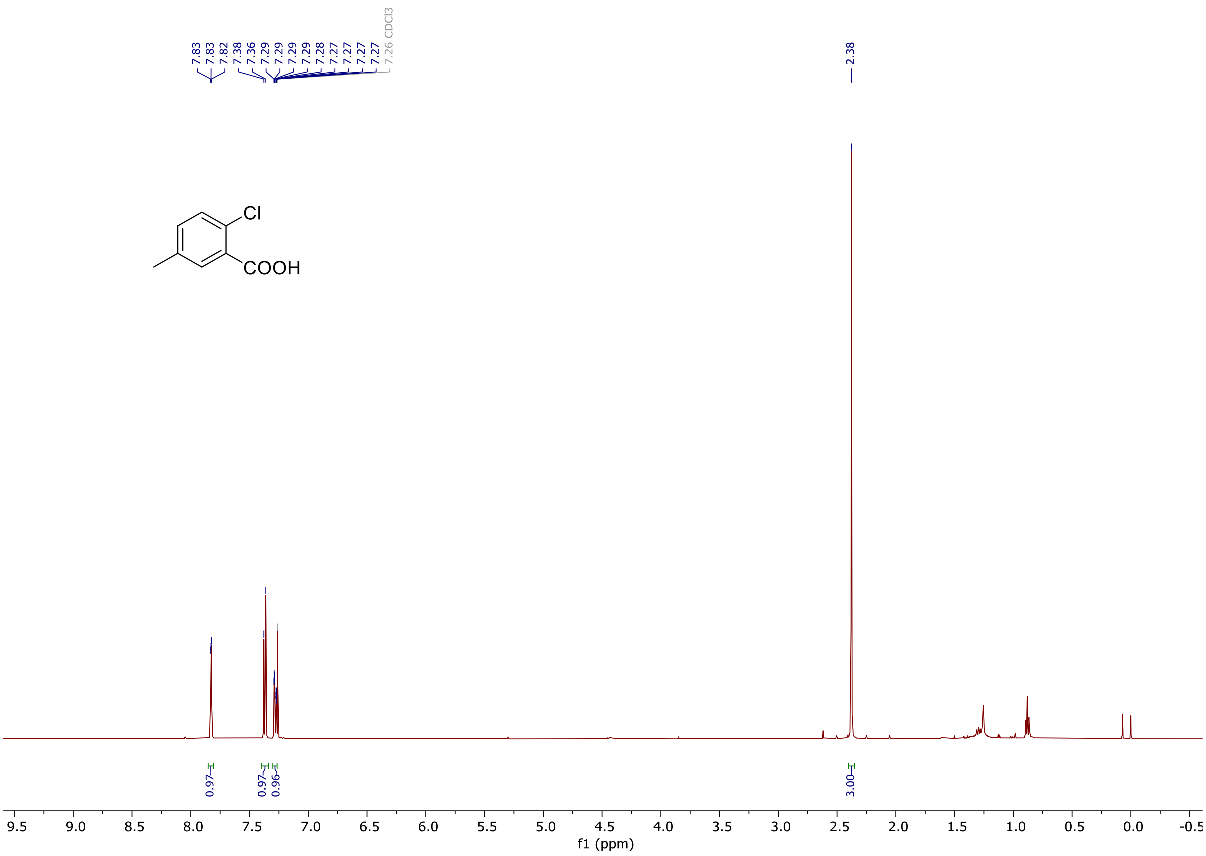

^{13}C NMR spectrum (126 MHz) in $CDCl_3$

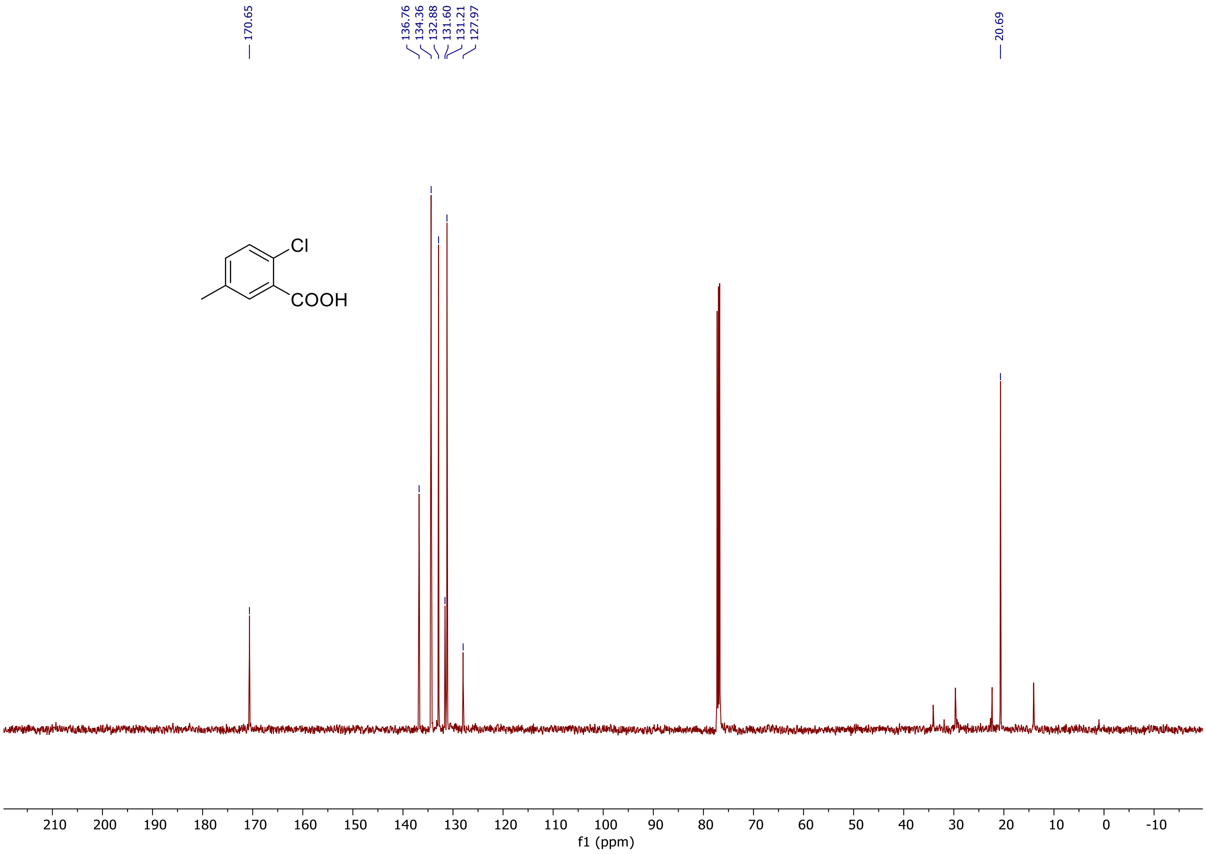

2-chloro-3-methoxy-5-methylbenzoic acid (3g):

^{1}H NMR spectrum (500 MHz) in CDCl$_3$

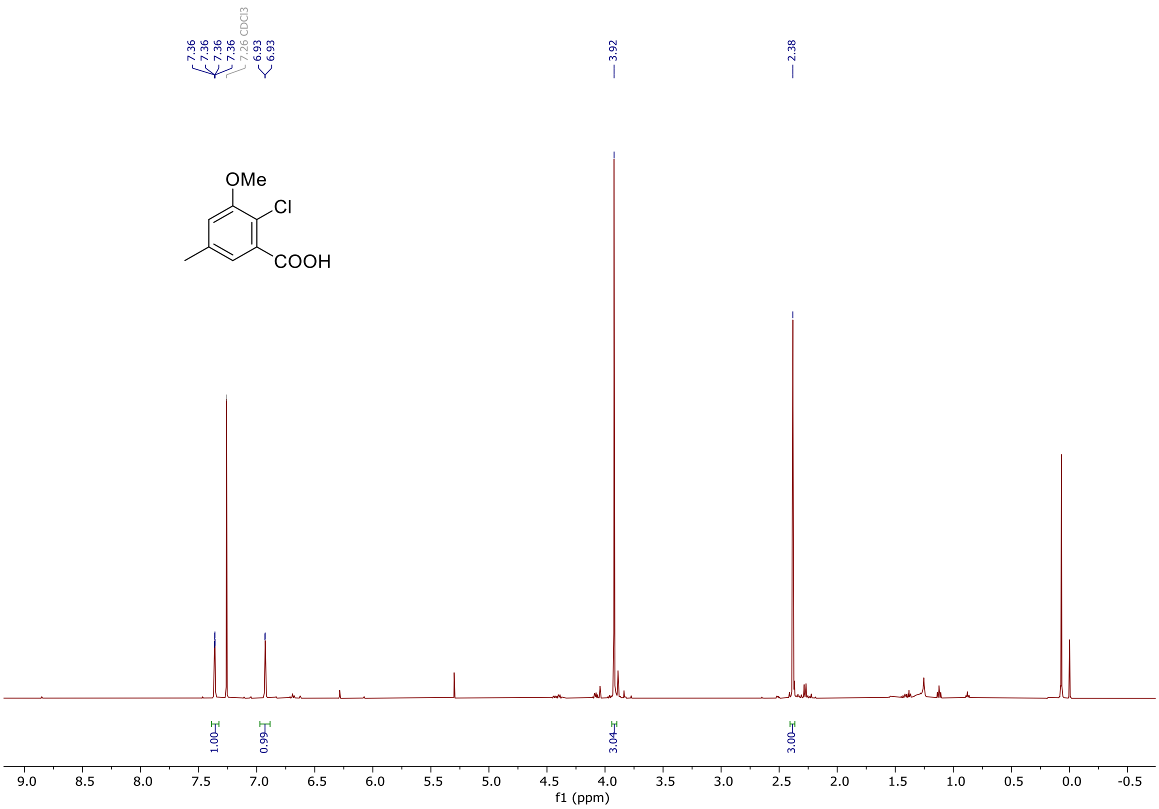

^{13}C NMR spectrum (126 MHz) in CDCl$_3$

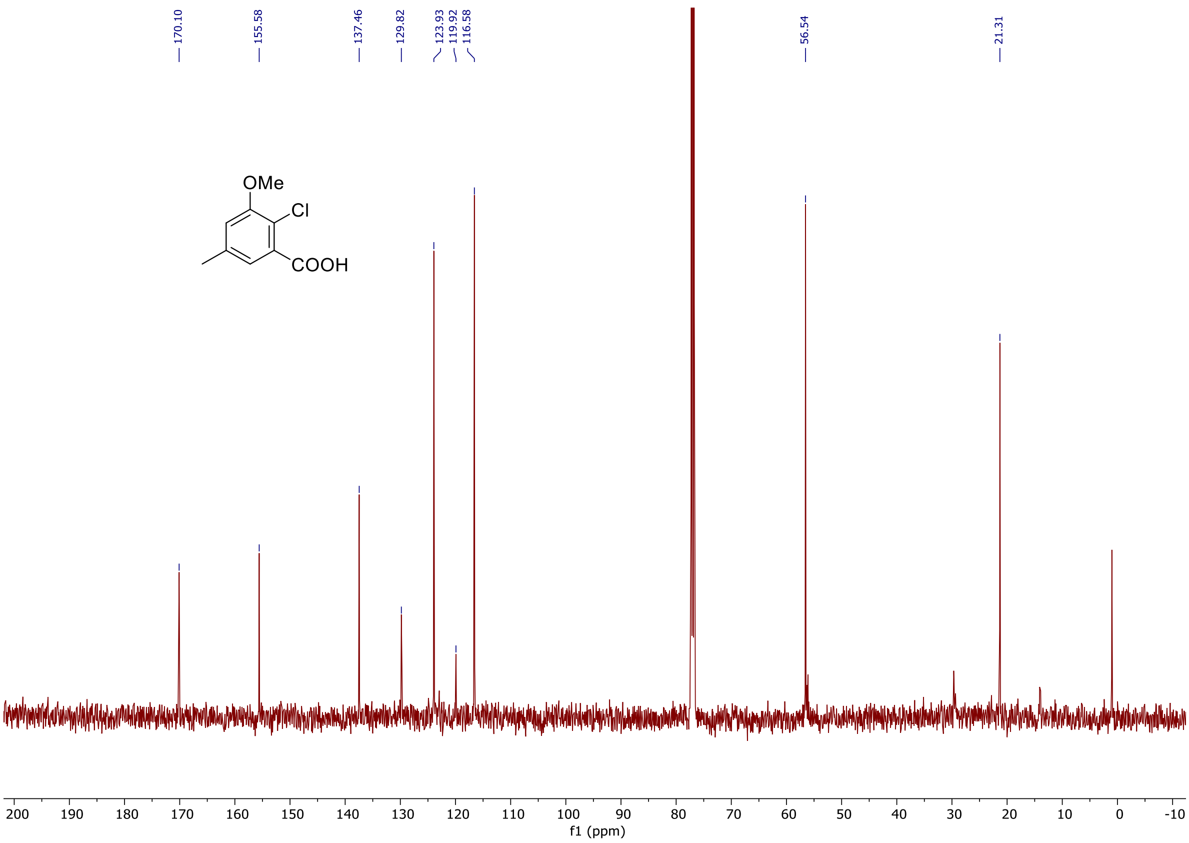

(*S*)-methyl 3-(2-(1,3-dioxoisoindolin-2-yl)-3-methoxy-3-oxopropyl)benzoate (3h-*meta*) and (*S*)-methyl 4-(2-(1,3-dioxoisoindolin-2-yl)-3-methoxy-3-oxopropyl)benzoate (3h-*para*)

^{1}H NMR spectrum (600 MHz) in $CDCl_3$

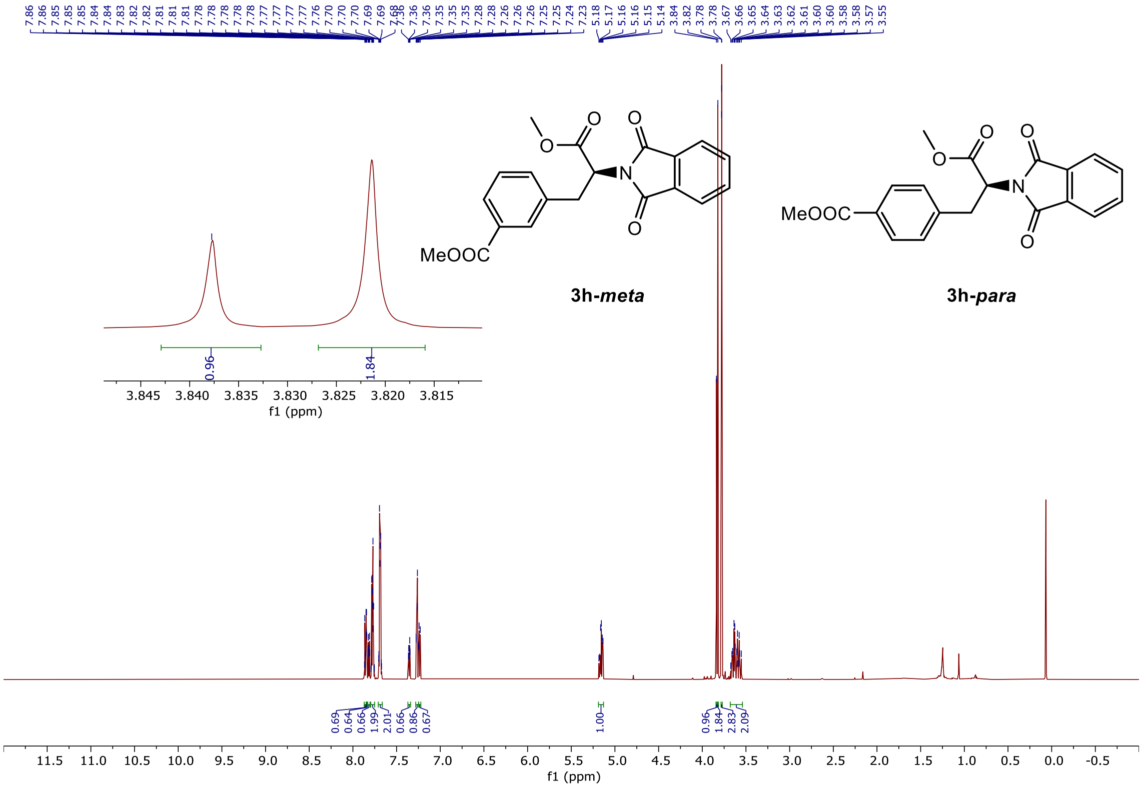

^{13}C NMR spectrum (151 MHz) in $CDCl_3$

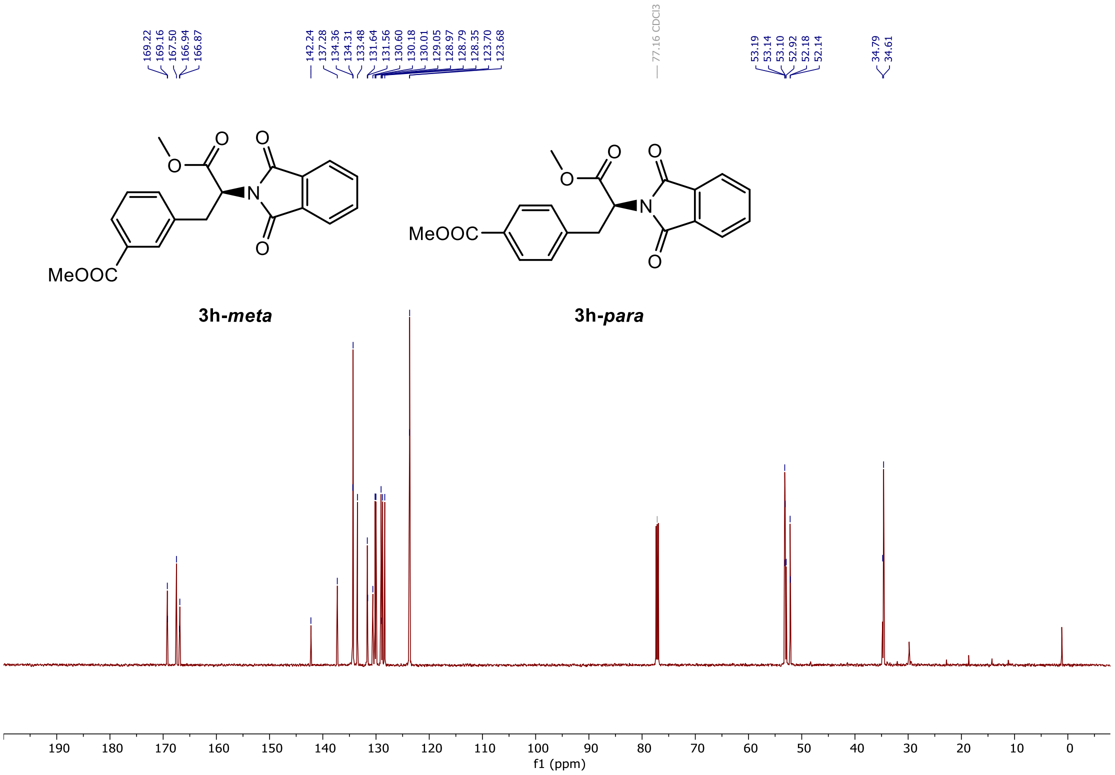

(*R*)-3-((2-oxo-3-propionyloxazolidin-4-yl)methyl)benzoic acid (3i-*meta*) and (*R*)-4-((2-oxo-3-propionyloxazolidin-4-yl)methyl)benzoic acid (3i-*para*)

^{1}H NMR spectrum (600 MHz) in $CDCl_3$

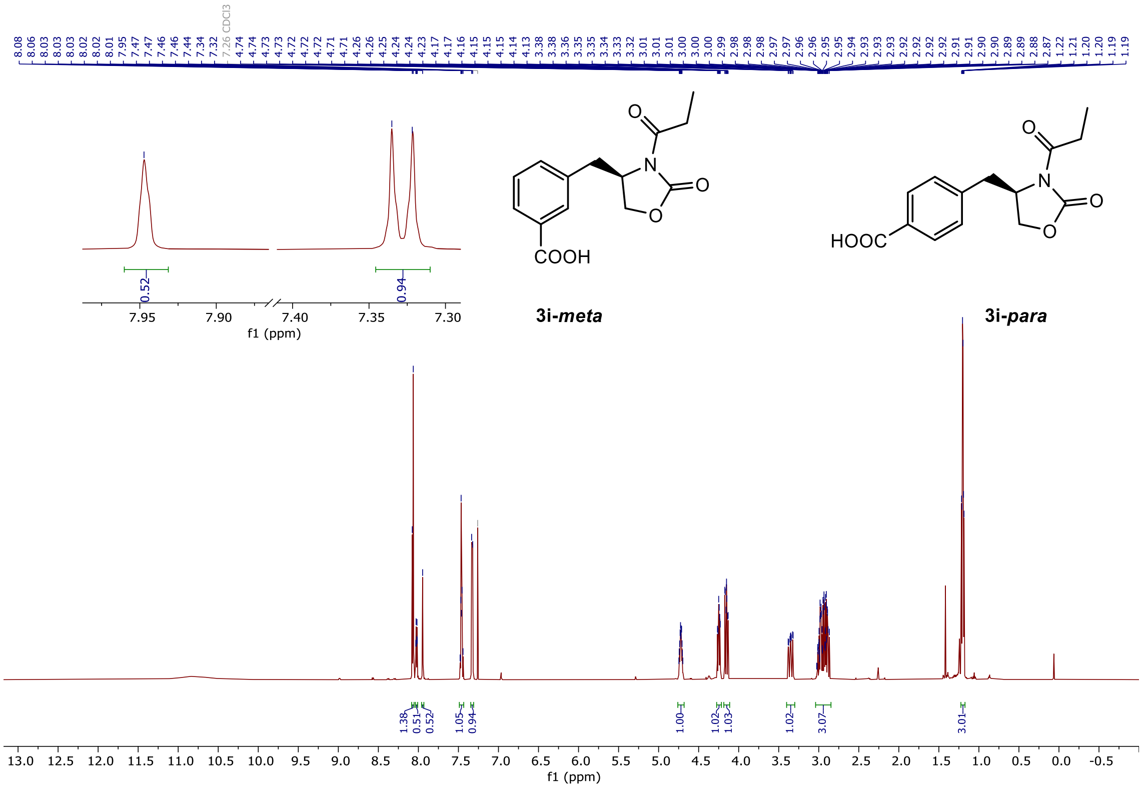

^{13}C NMR spectrum (151 MHz) in $CDCl_3$

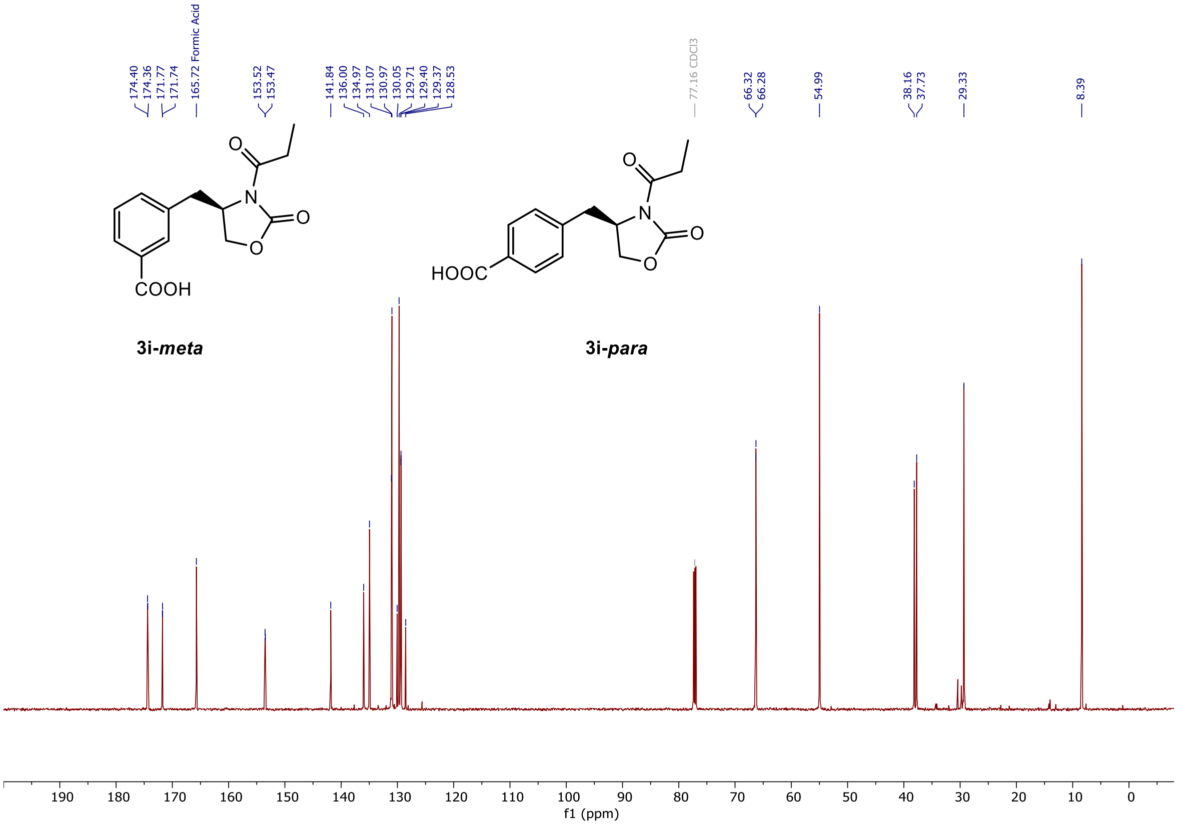

3-((*R*)-2-((1*r*,4*R*)-4-isopropylcyclohexanecarboxamido)-3-methoxy-3-oxopropyl)benzoic acid (3j-*meta*) and 4-((*R*)-2-((1*r*,4*R*)-4-isopropylcyclohexanecarboxamido)-3-methoxy-3-oxopropyl)benzoic acid (3j-*para*)

^{1}H NMR spectrum (600 MHz) in CD_3OD

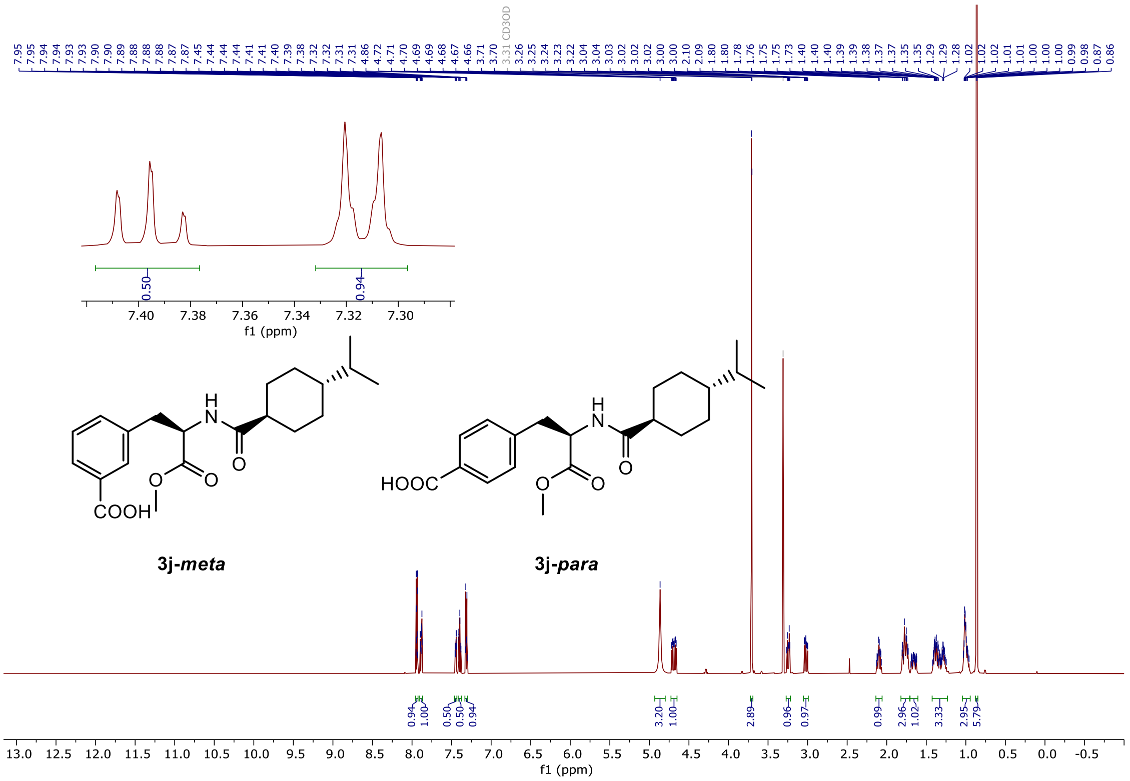

^{13}C NMR spectrum (151 MHz) in CD_3OD

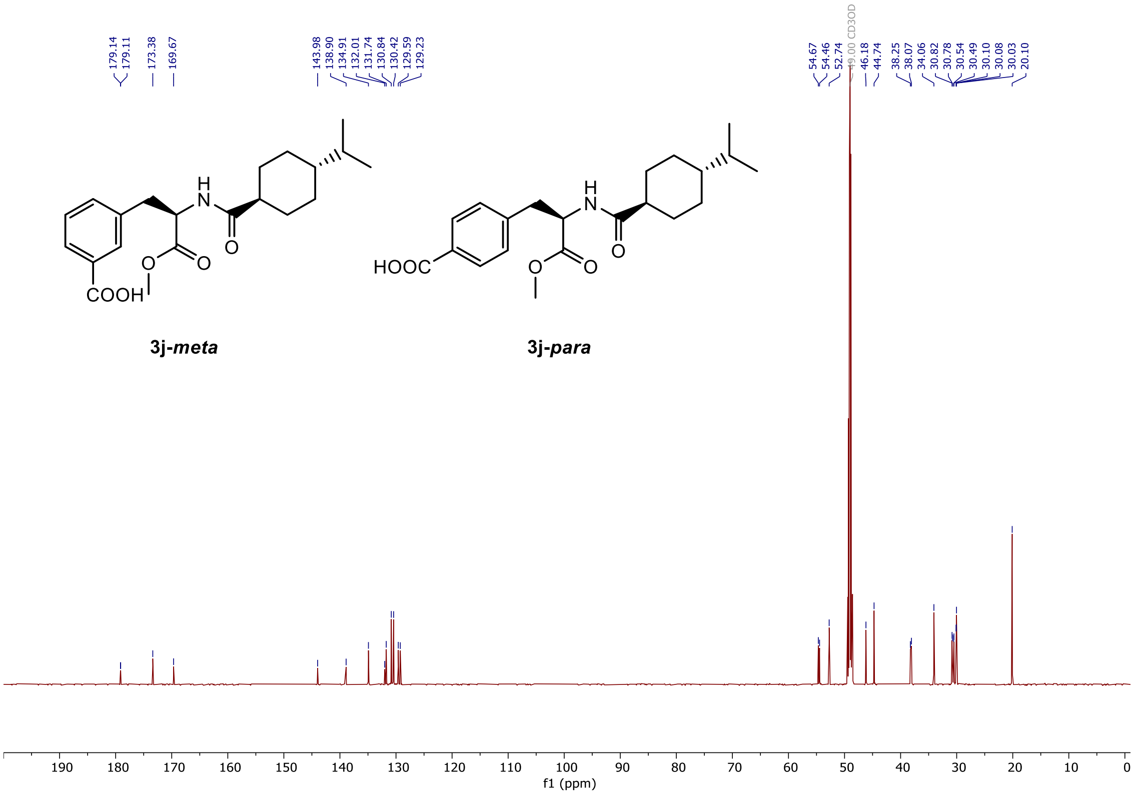

2'-fluoro-4'-(1-methoxy-1-oxopropan-2-yl)-[1,1'-biphenyl]-3-carboxylic acid (3k-β) and 2'-fluoro-4'-(1-methoxy-1-oxopropan-2-yl)-[1,1'-biphenyl]-4-carboxylic acid (3k-γ)

^{1}H NMR spectrum (600 MHz) in $CDCl_3$

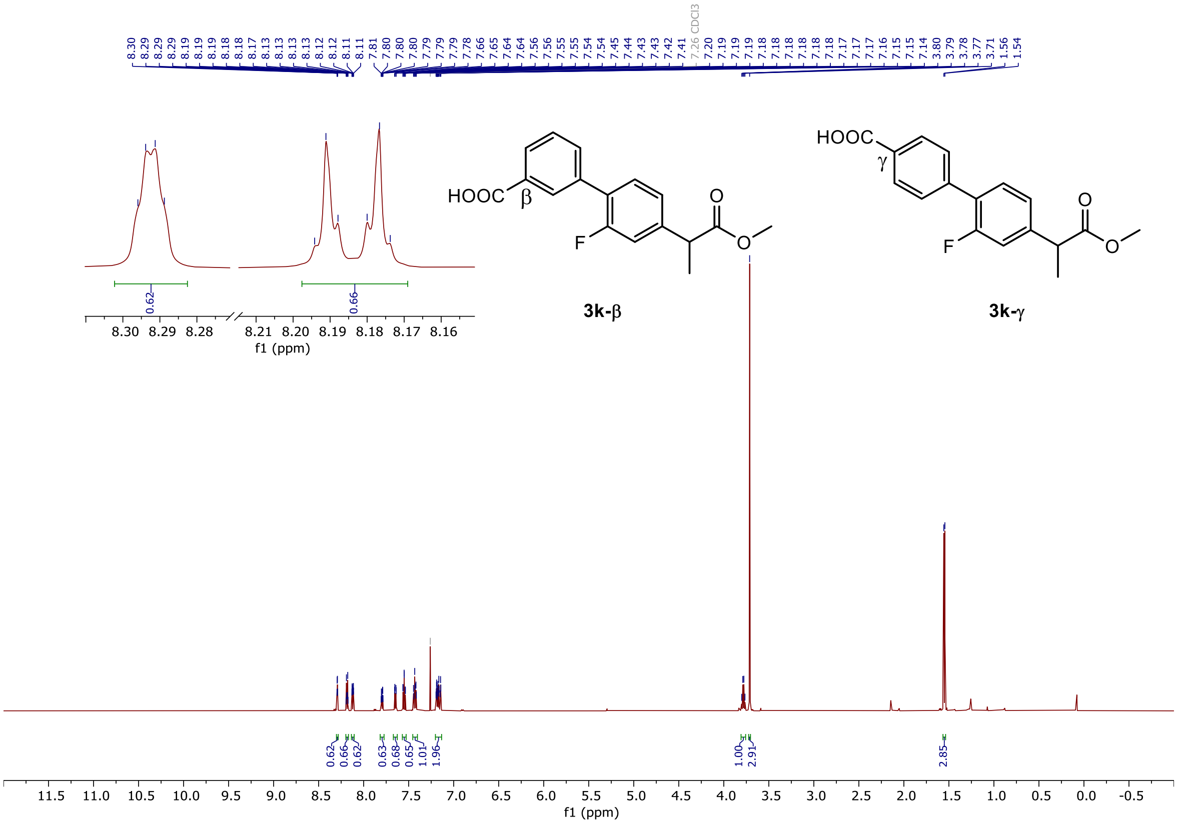

^{13}C NMR spectrum (151 MHz) in $CDCl_3$

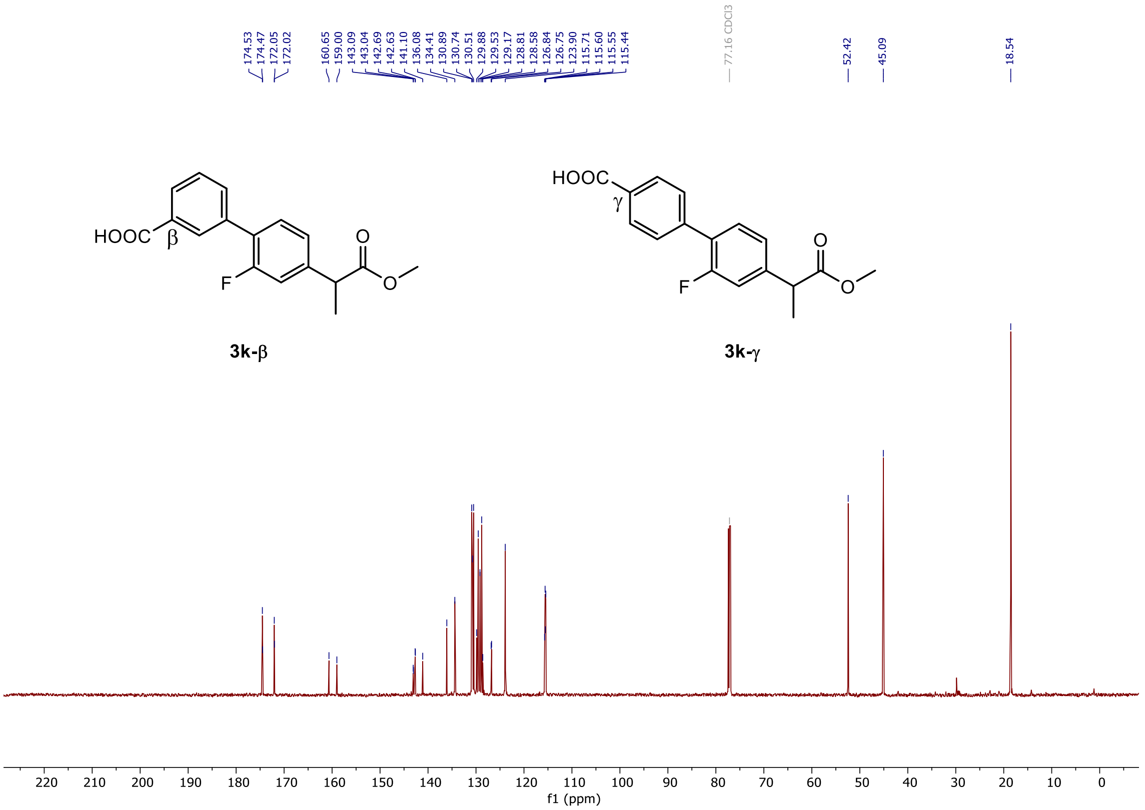

^{19}F NMR spectrum (471 MHz) in $CDCl_3$

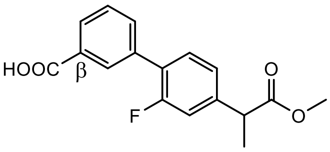

3k-β

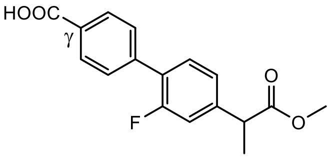

3k-γ

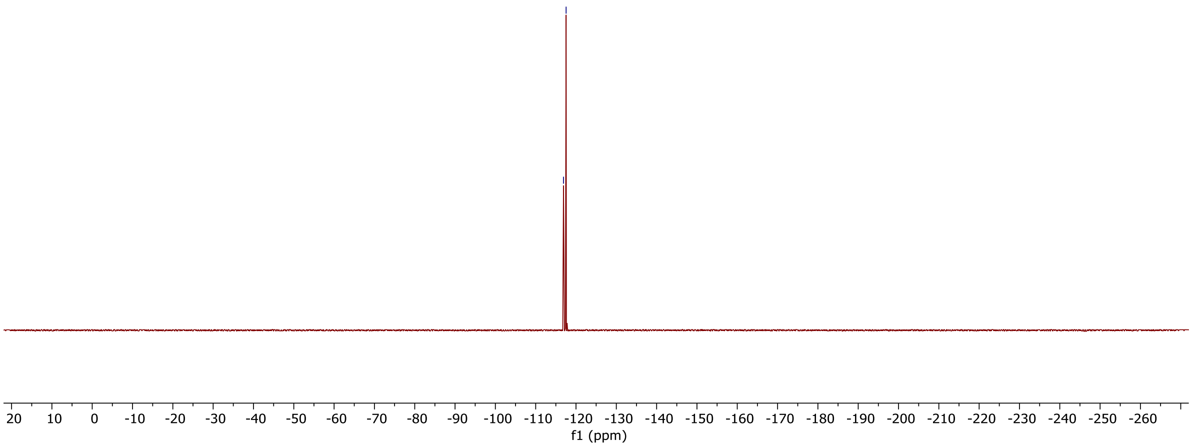

3-(3-(1-methoxy-1-oxopropan-2-yl)benzoyl)benzoic acid (3l-β), 3-benzoyl-5-(1-methoxy-1-oxopropan-2-yl)benzoic acid (3l-β') and 4-(3-(1-methoxy-1-oxopropan-2-yl)benzoyl)benzoic acid (3l-γ):

^{1}H NMR spectrum (600 MHz) in $CDCl_3$

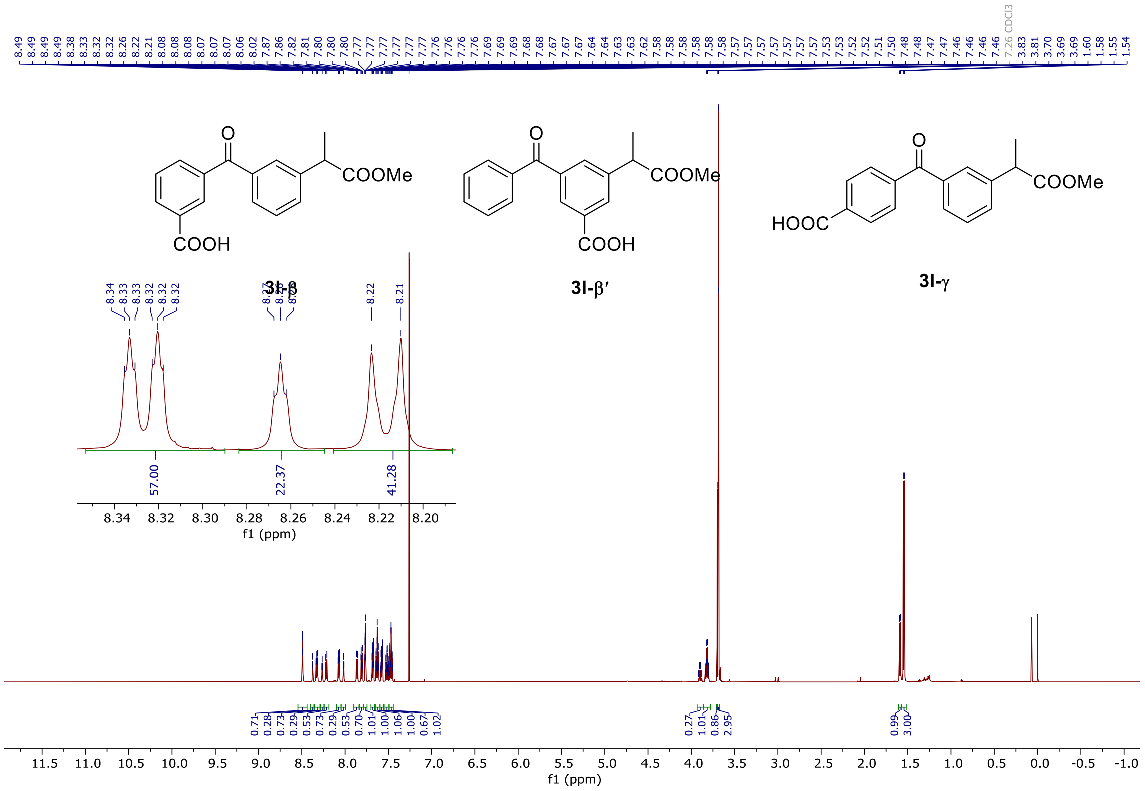

^{13}C NMR spectrum (151 MHz) in $CDCl_3$

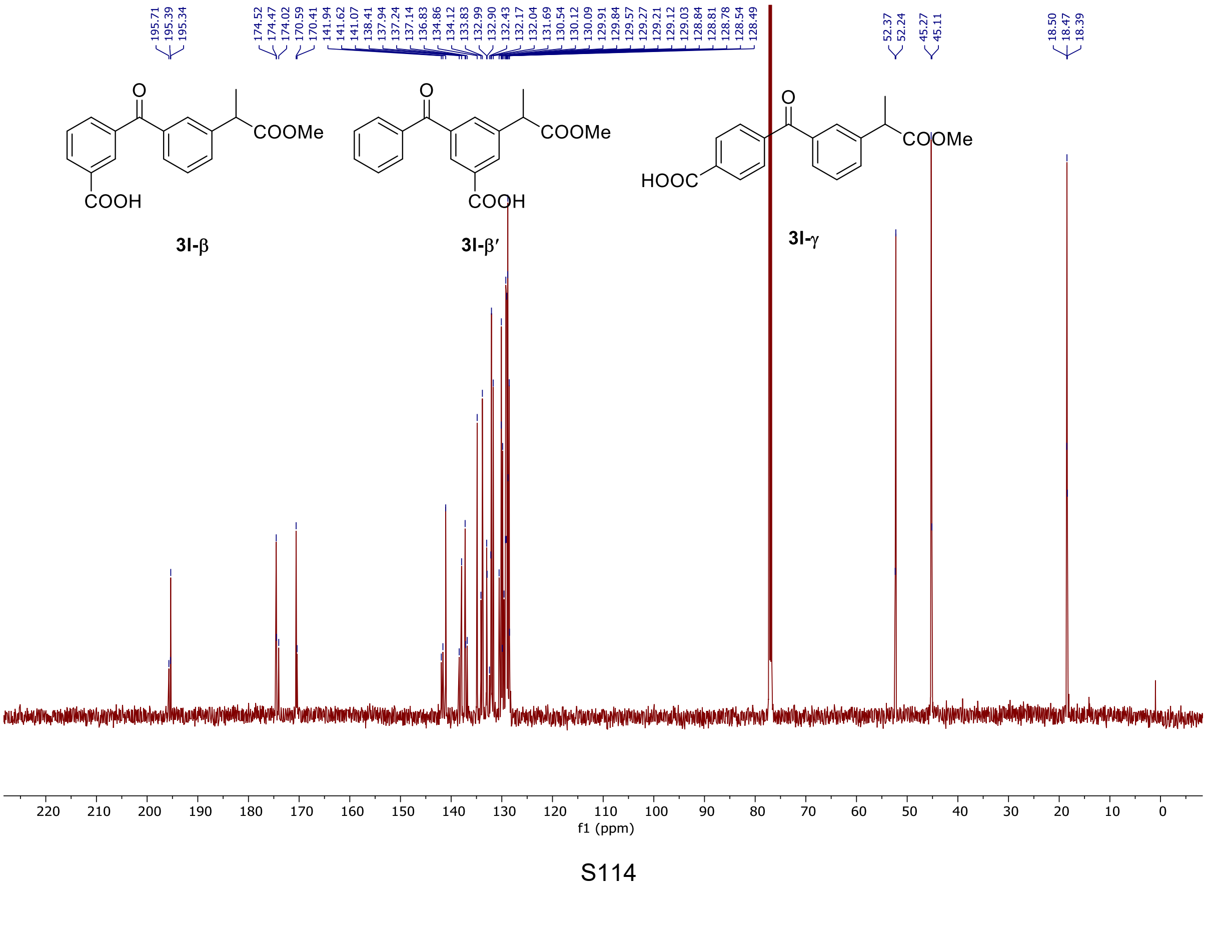

3-(3-methoxy-3-oxopropyl)-5-methylbenzoic acid (3m):

^{1}H NMR spectrum (500 MHz) in $CDCl_3$

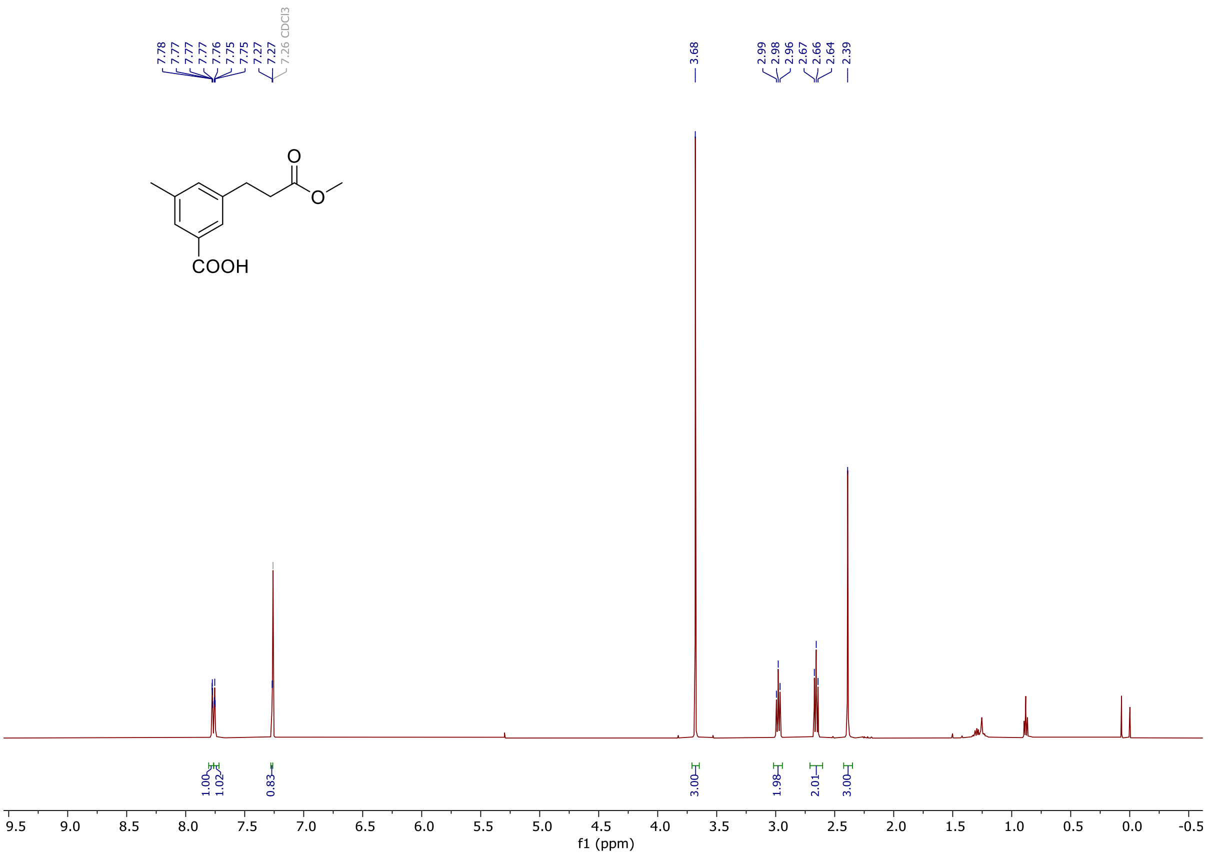

^{13}C NMR spectrum (126 MHz) in $CDCl_3$

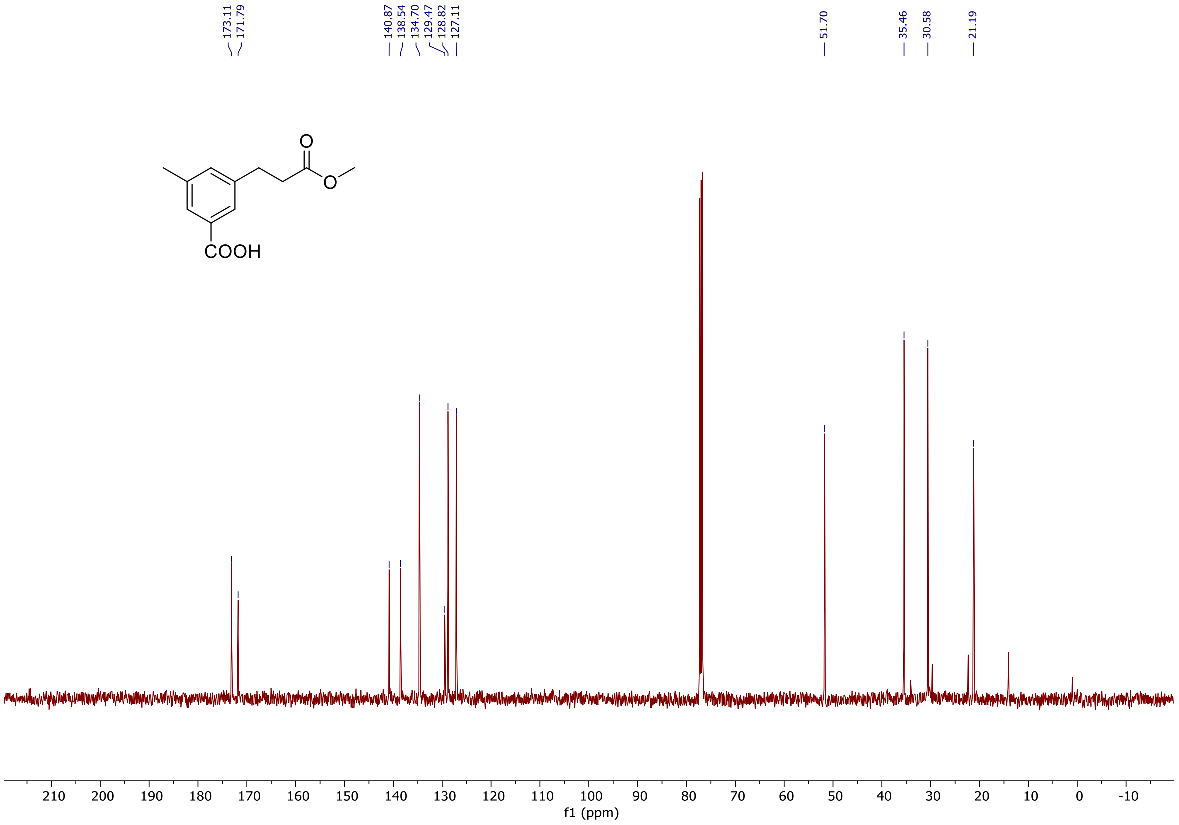

4-(methoxycarbonyl)-5,6,7,8-tetrahydronaphthalene-2-carboxylic acid (3n):

^{1}H NMR spectrum (600 MHz) in $CDCl_3$

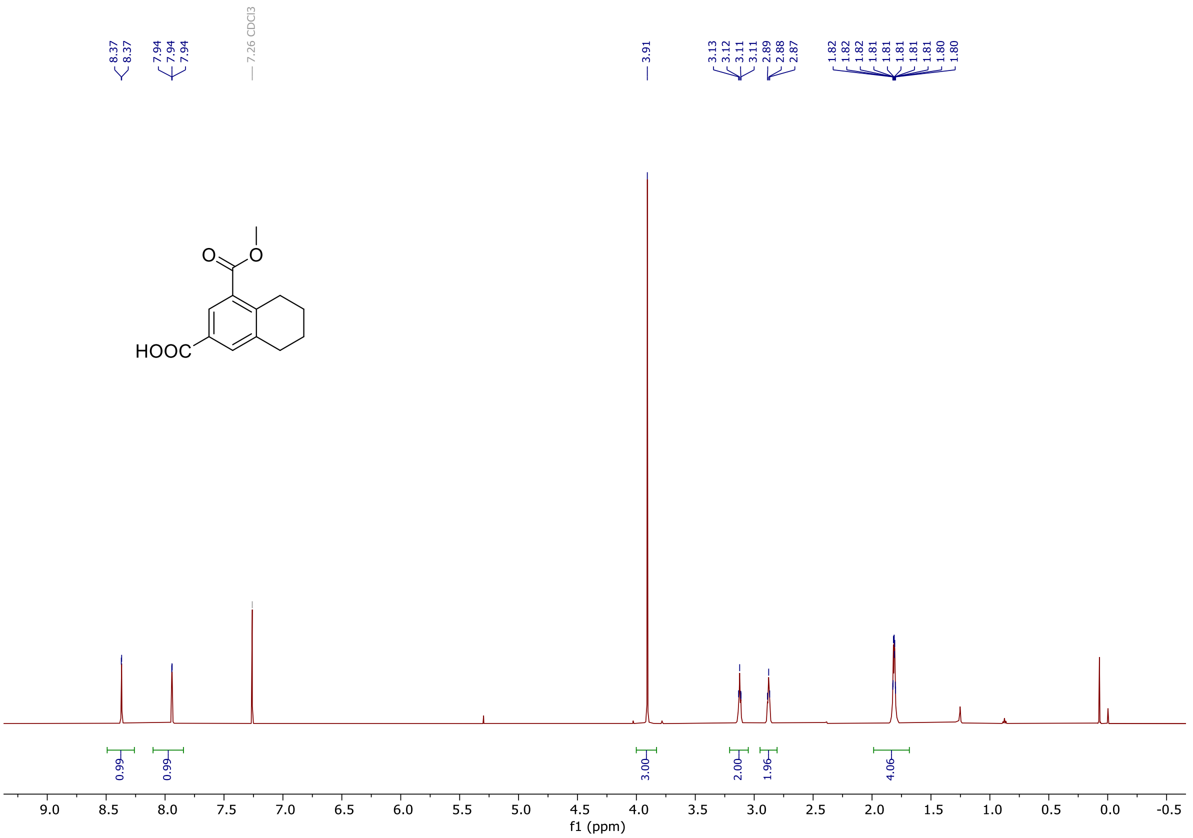

^{13}C NMR spectrum (151 MHz) in $CDCl_3$

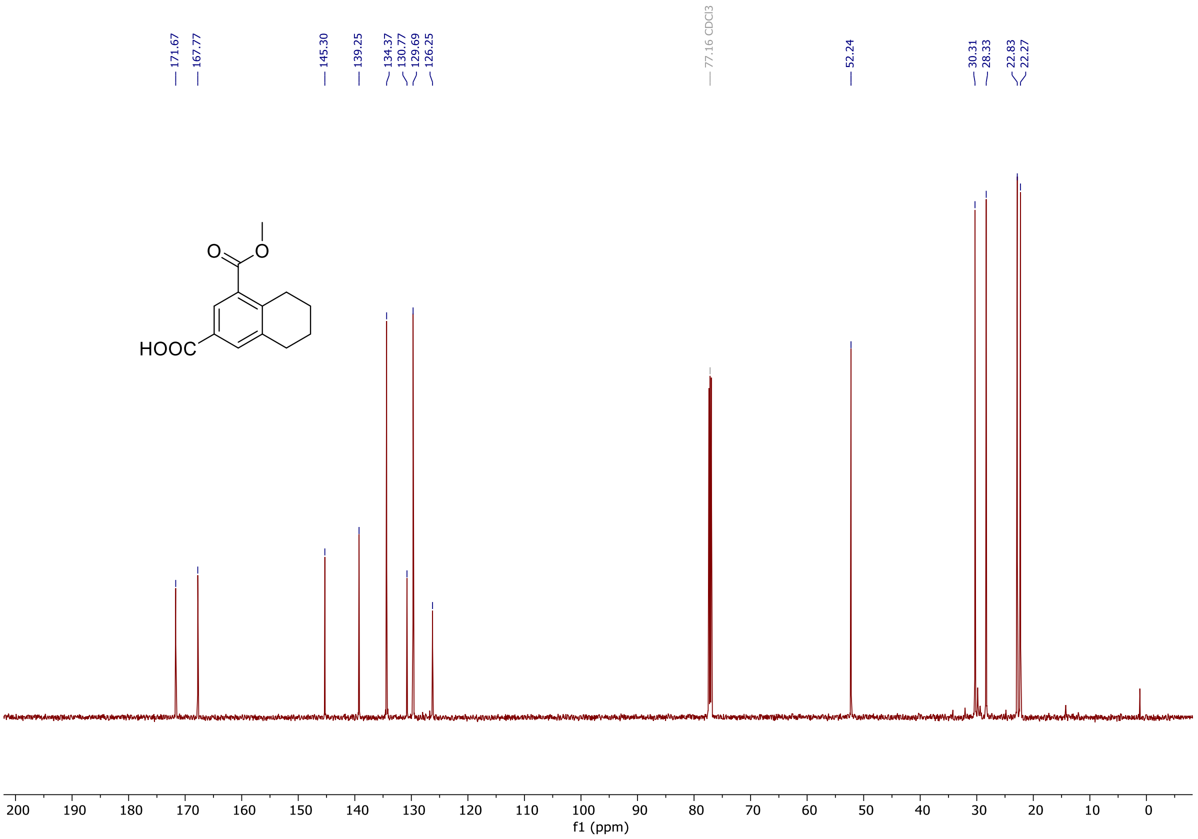

3-((2-isopropyl-5-methylphenoxy)methyl)-5-methylbenzoic acid (3o)

¹H NMR spectrum (600 MHz) in $CDCl_3$

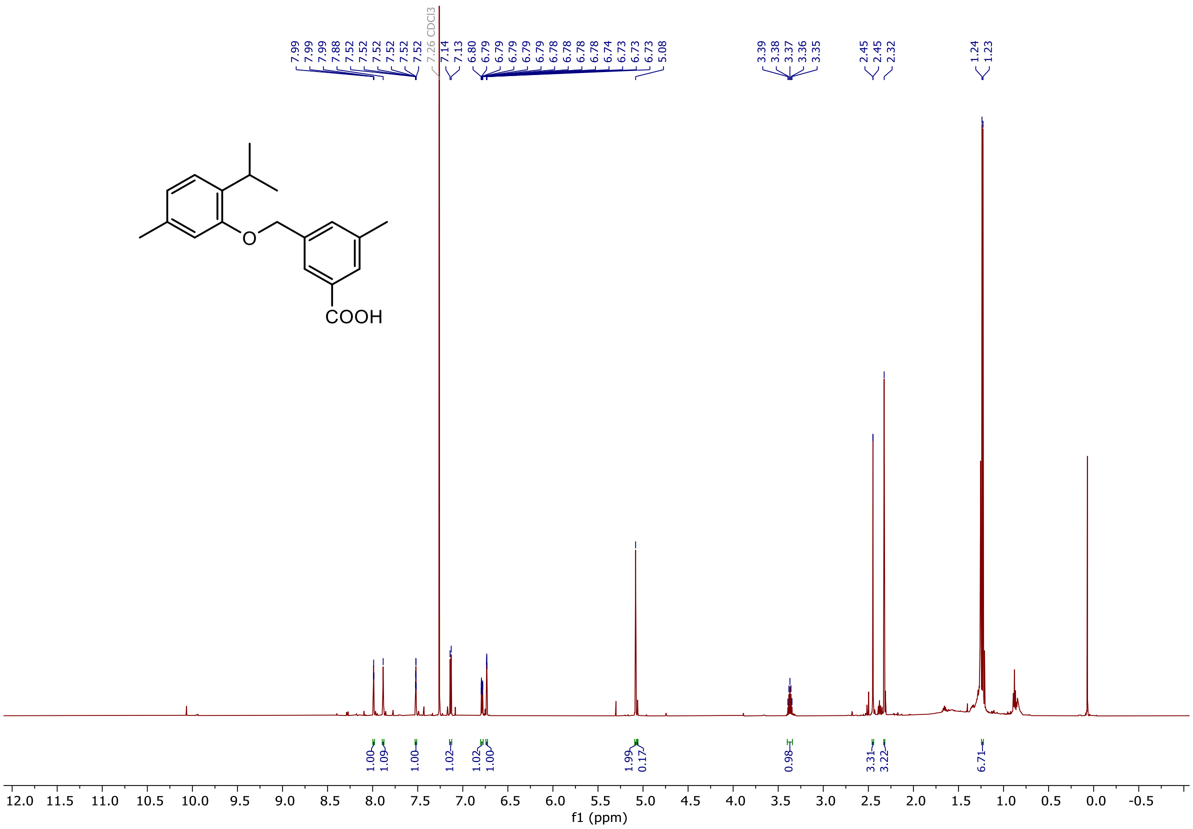

¹³C NMR spectrum (151 MHz) in $CDCl_3$

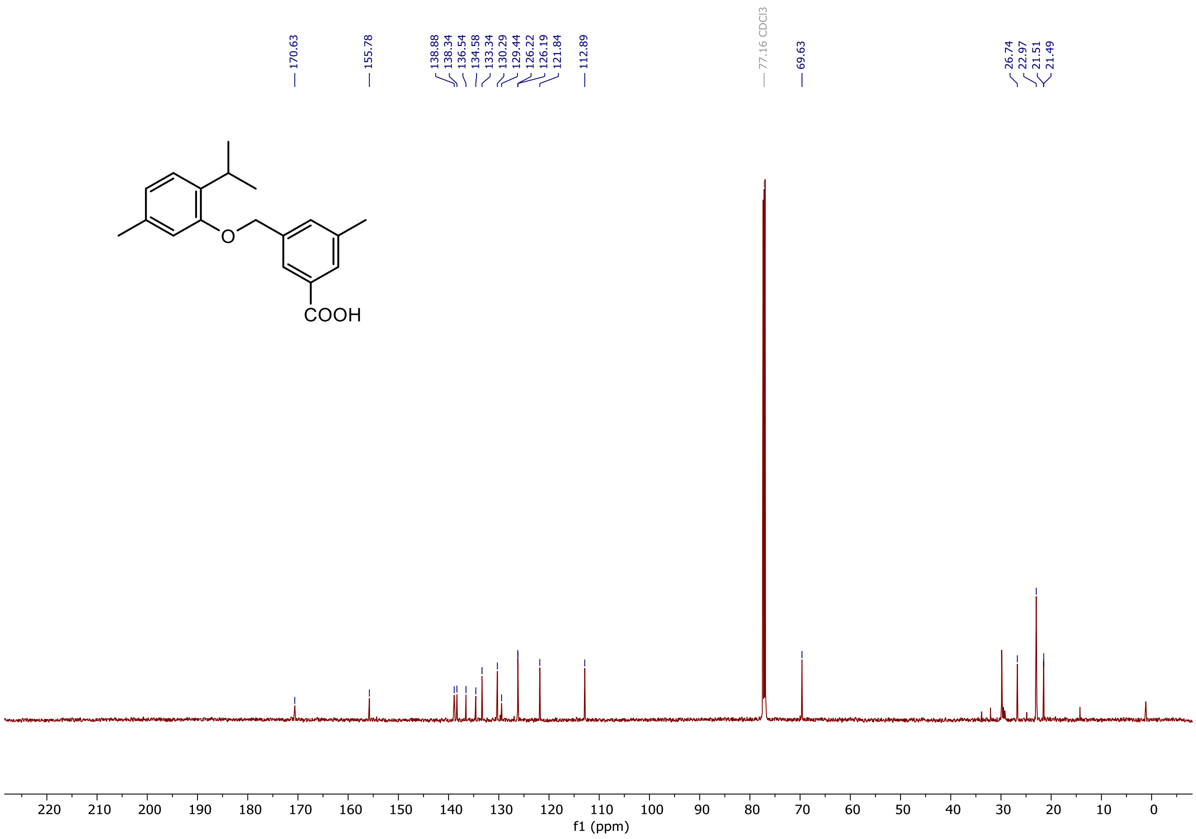

5-(methoxycarbonyl)-6-methyl-4'-(trifluoromethoxy)-[1,1'-biphenyl]-3-carboxylic acid (3p)

^{1}H NMR spectrum (500 MHz) in $CDCl_3$

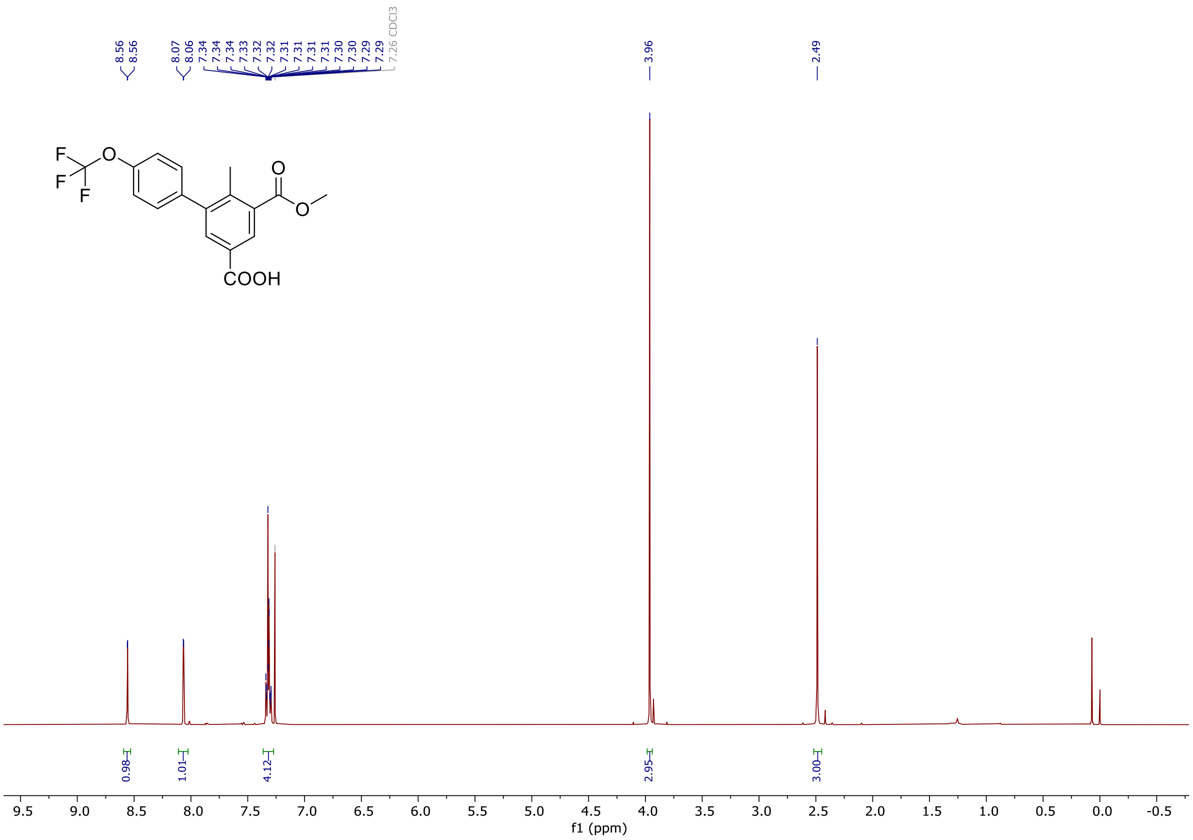

^{13}C NMR spectrum (126 MHz) in $CDCl_3$

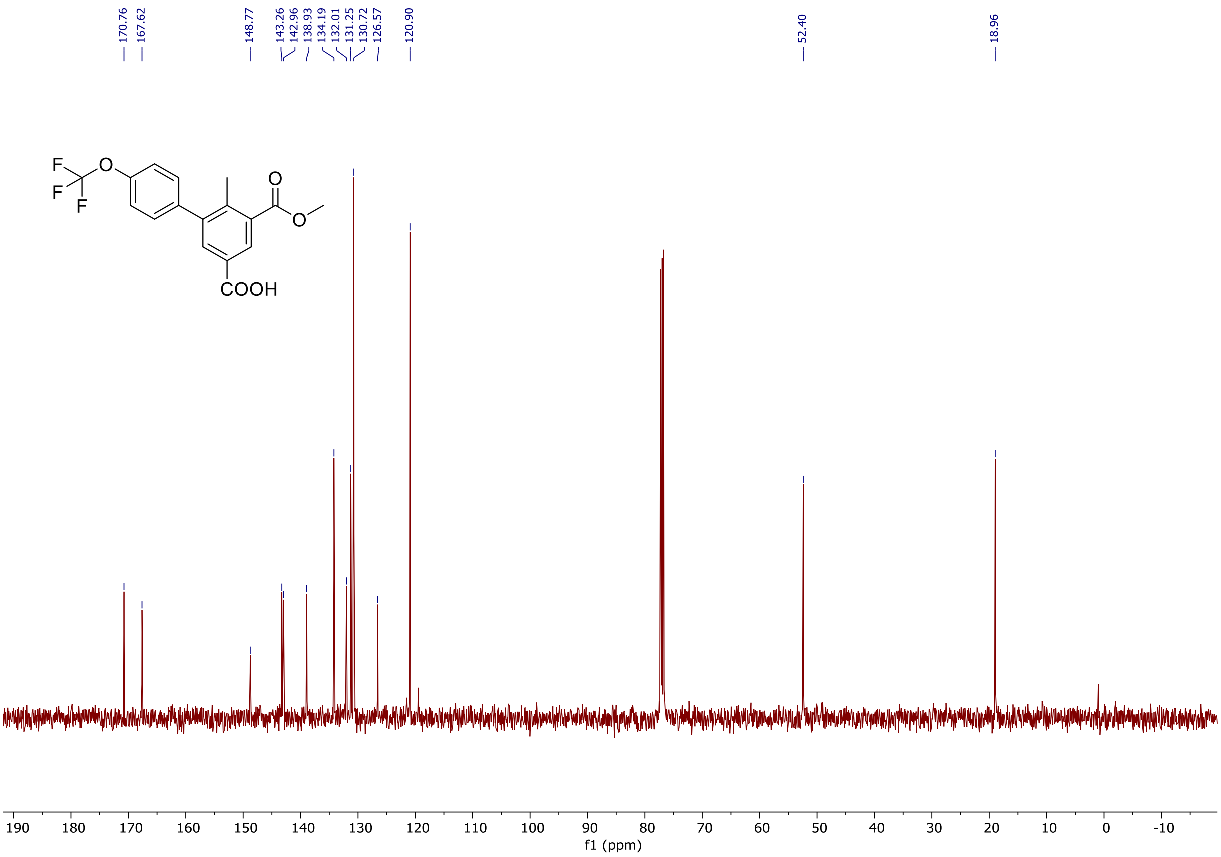

^{19}F NMR spectrum (471 MHz) in $CDCl_3$

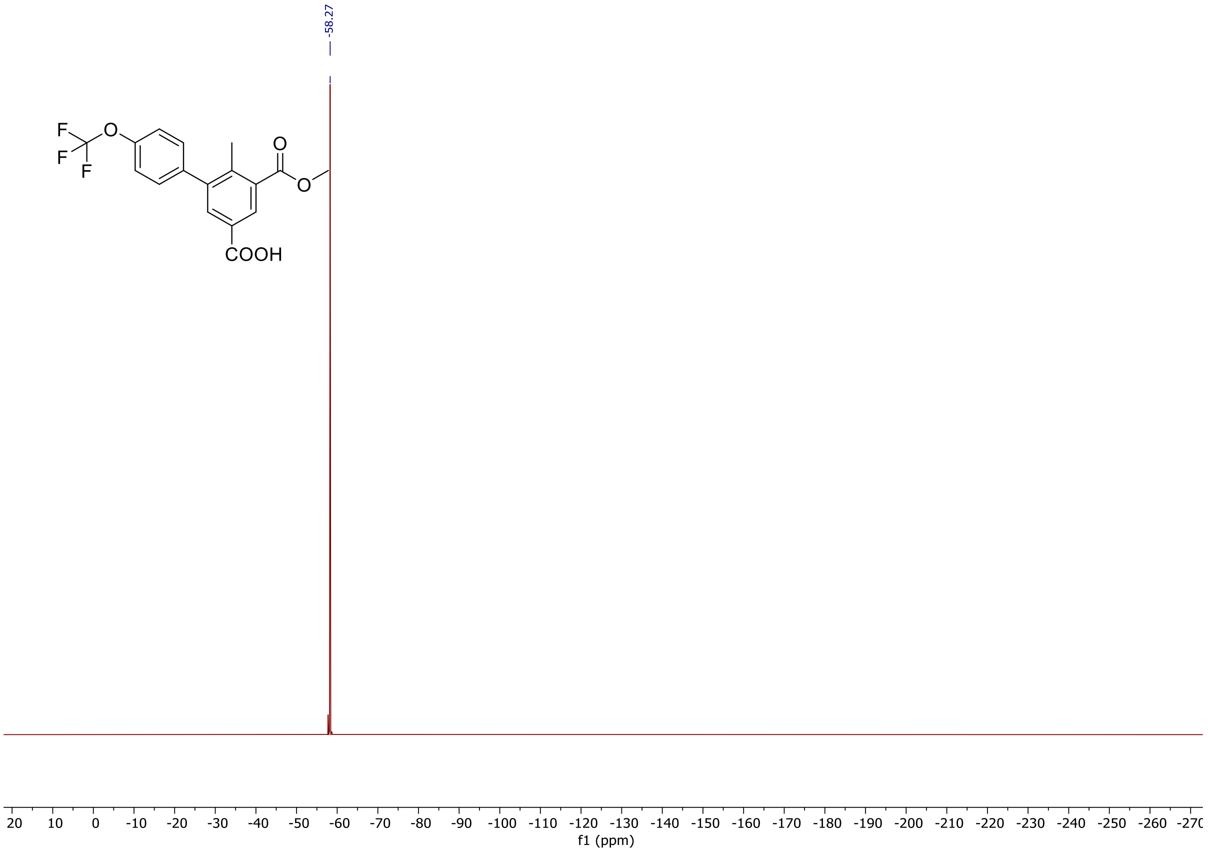

4-((5-methoxy-4,4-dimethyl-5-oxopentyl)oxy)-2,5-dimethylbenzoic acid (3q):

^{1}H NMR spectrum (600 MHz) in $CDCl_3$

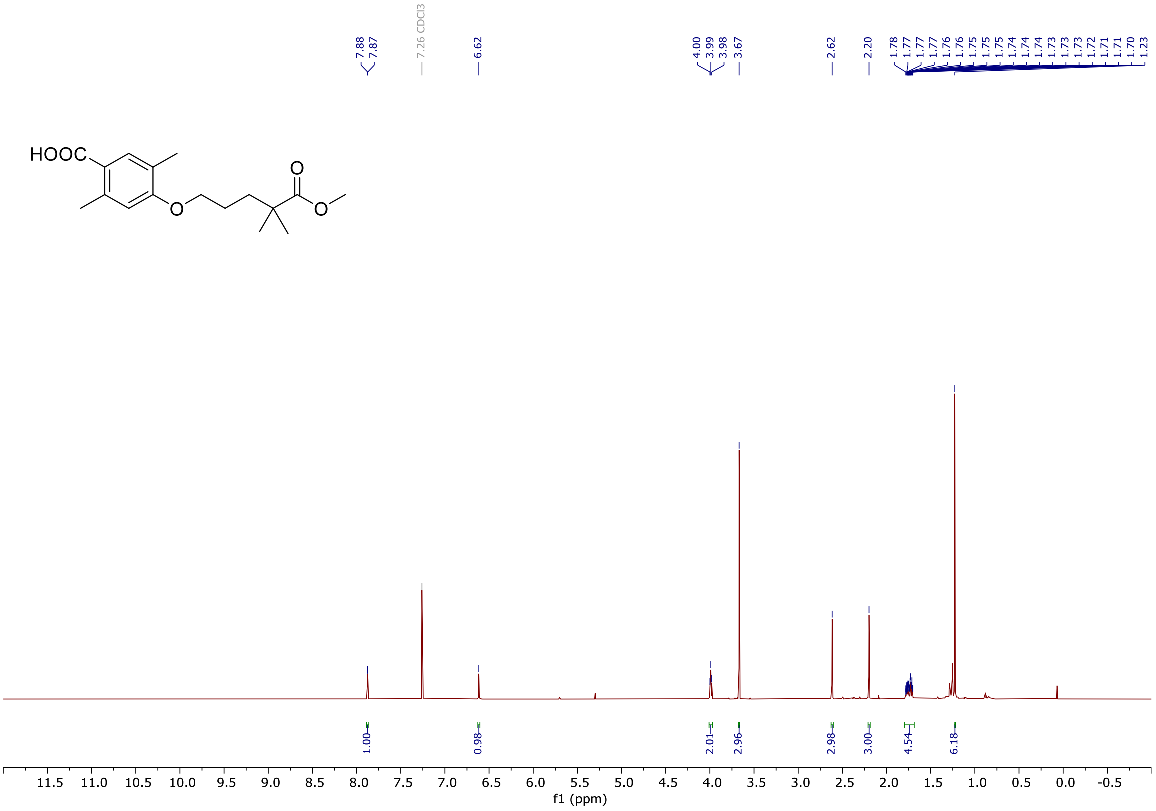

^{13}C NMR spectrum (151 MHz) in $CDCl_3$

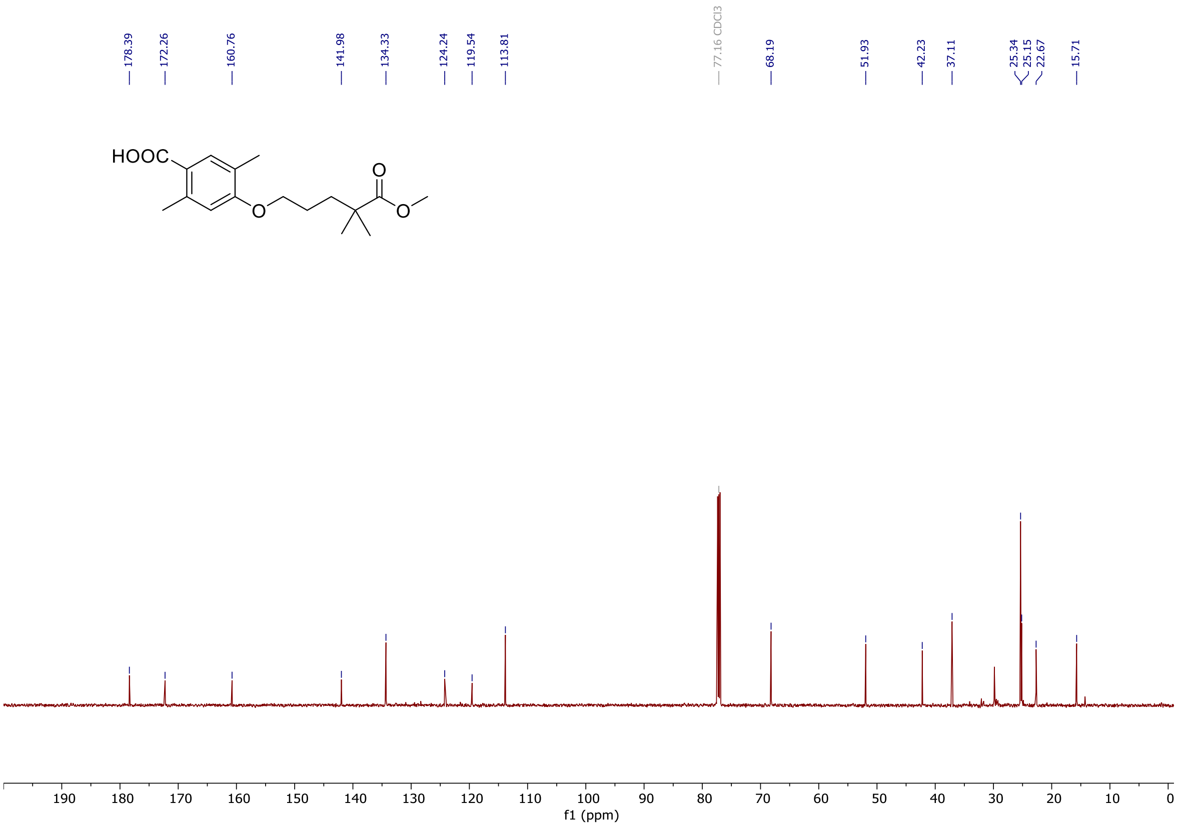

3-(*tert*-butyl)benzaldehyde (4a-*meta*) and 4-(*tert*-butyl)benzaldehyde (4a-*para*)

^{1}H NMR spectrum (600 MHz) in $CDCl_3$

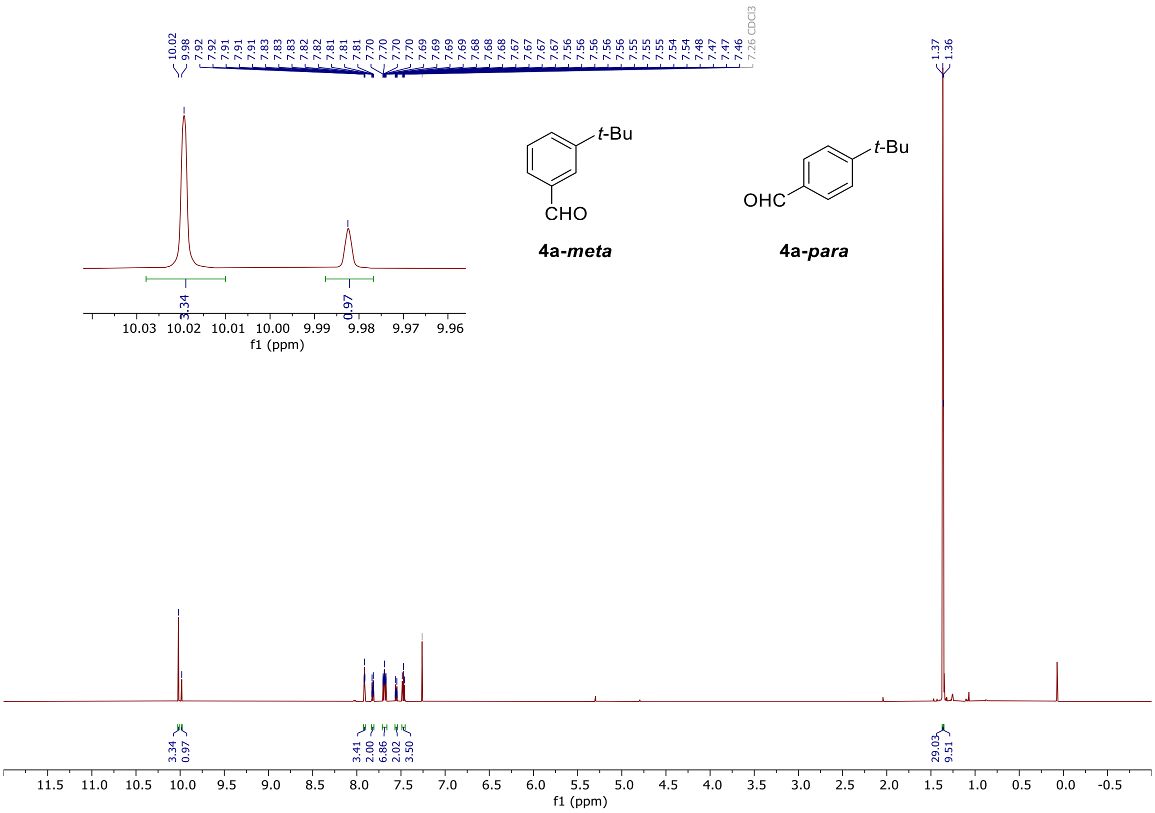

^{13}C NMR spectrum (151 MHz) in $CDCl_3$

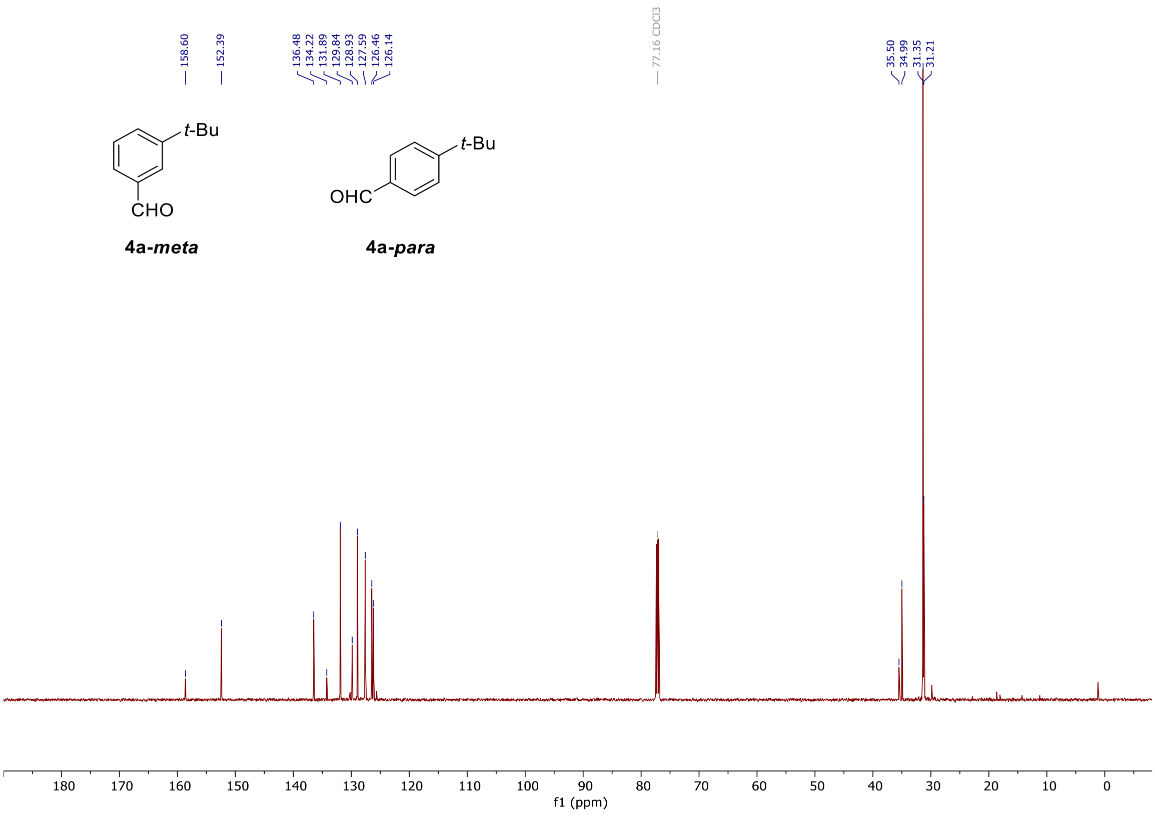

2,4-dimethylbenzaldehyde (4b-α) and 3,5-dimethylbenzaldehyde (4b-β):

^{1}H NMR spectrum (500 MHz) in $CDCl_3$

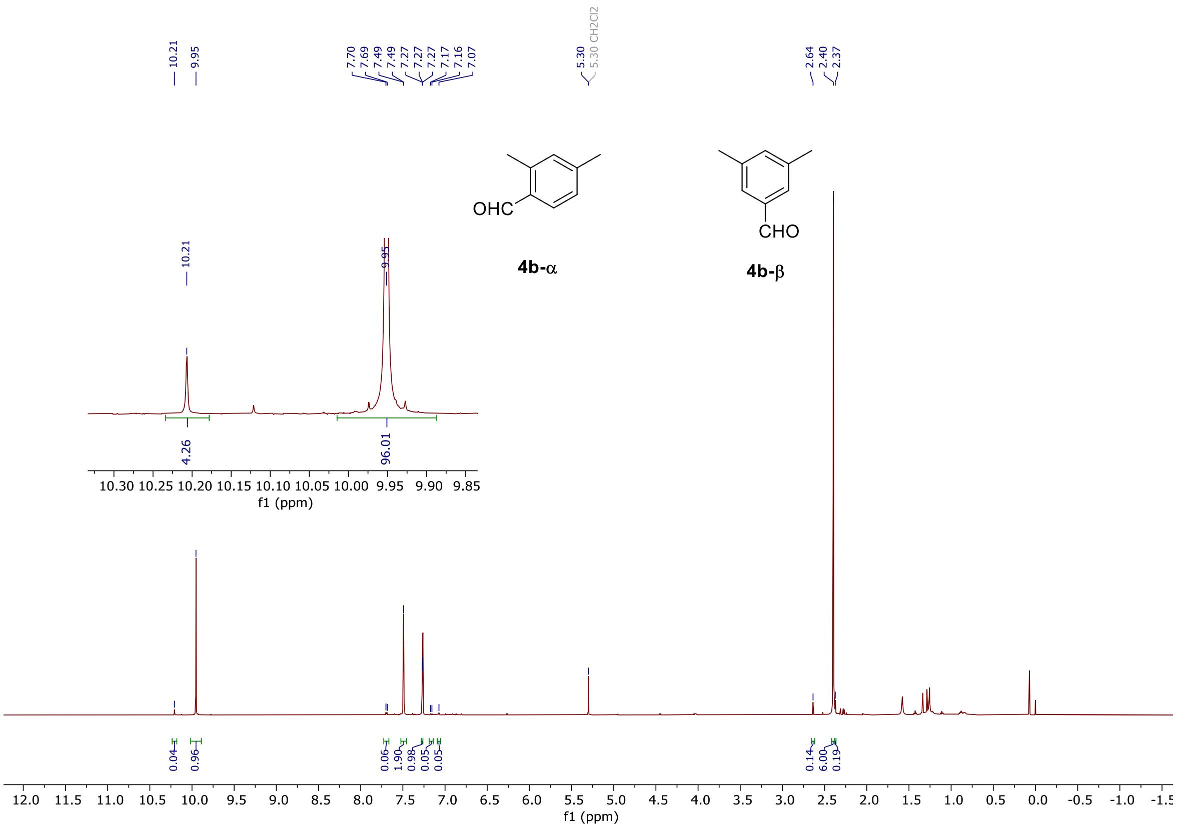

^{13}C NMR spectrum (126 MHz) in $CDCl_3$

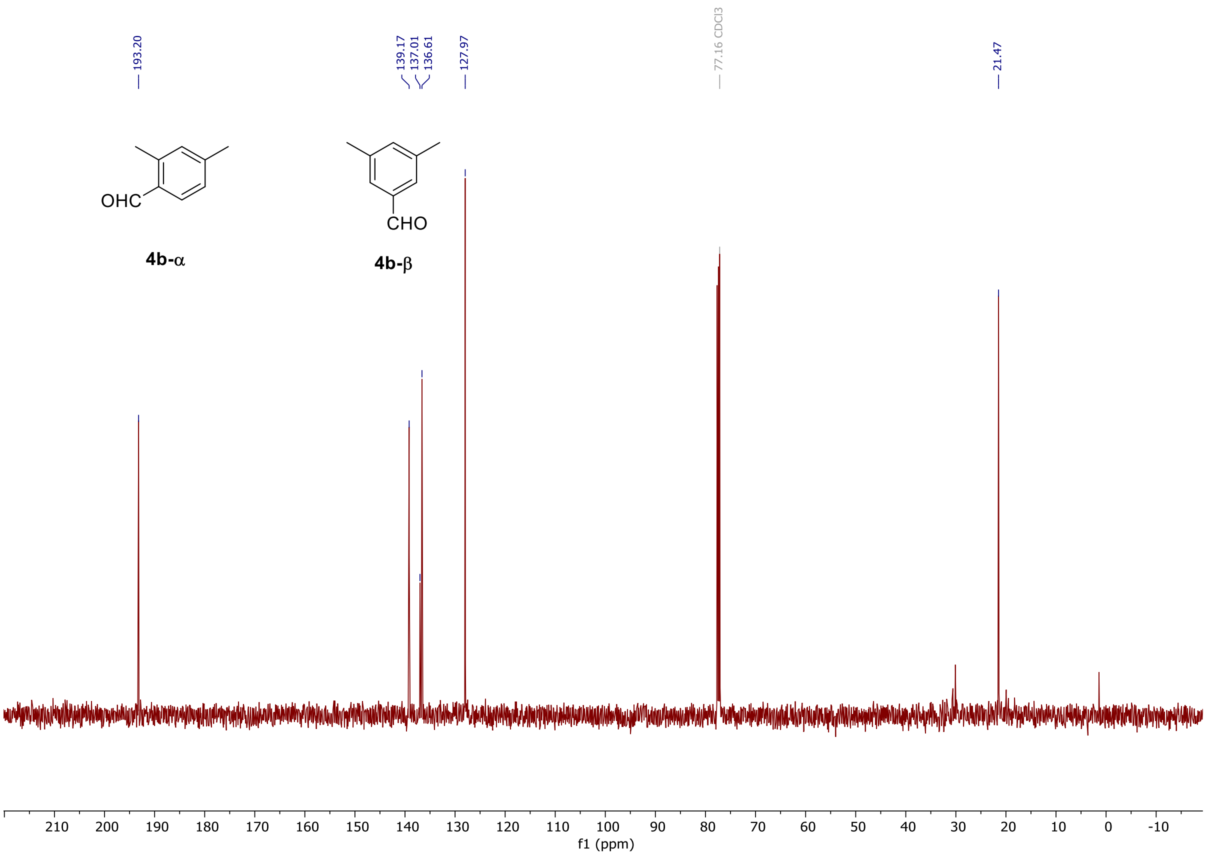

3-methoxy-5-methylbenzaldehyde (4c-β):

^{1}H NMR spectrum (500 MHz) in $CDCl_3$

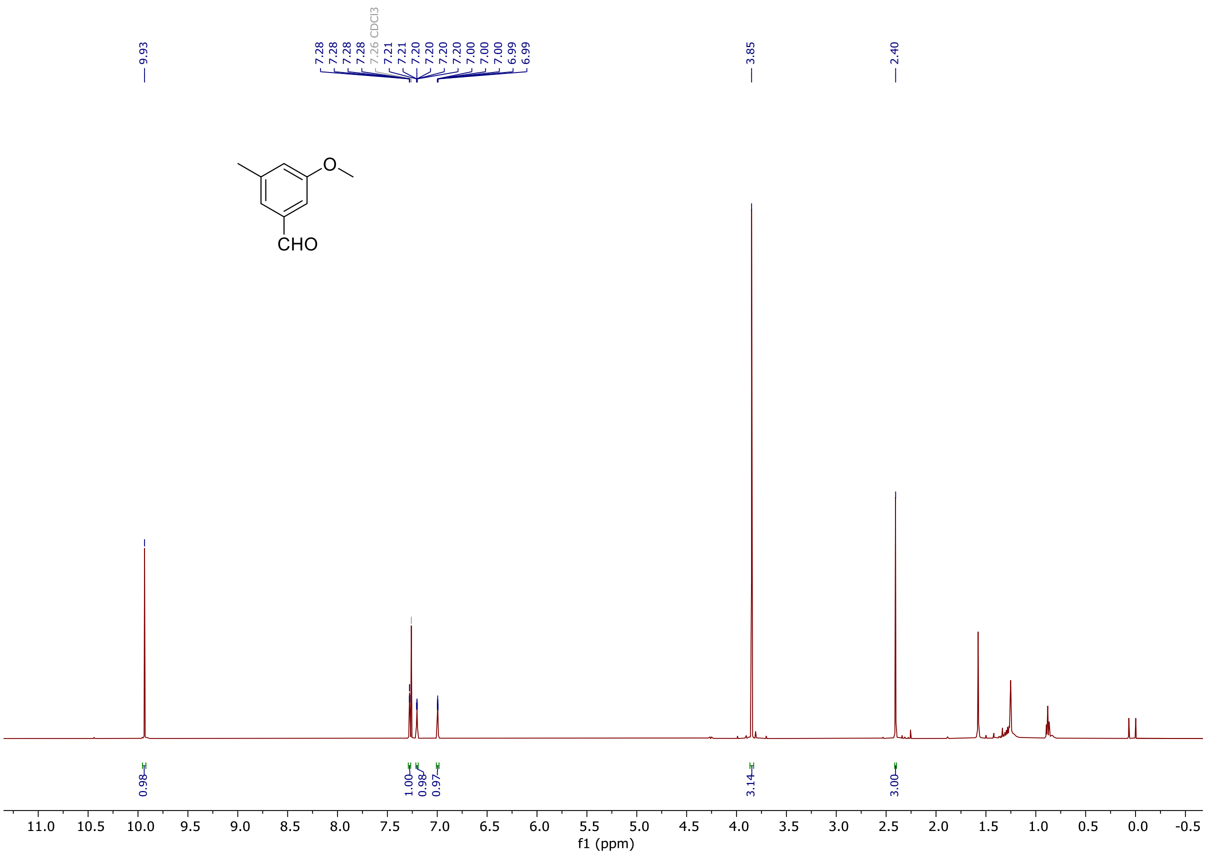

^{13}C NMR spectrum (126 MHz) in $CDCl_3$

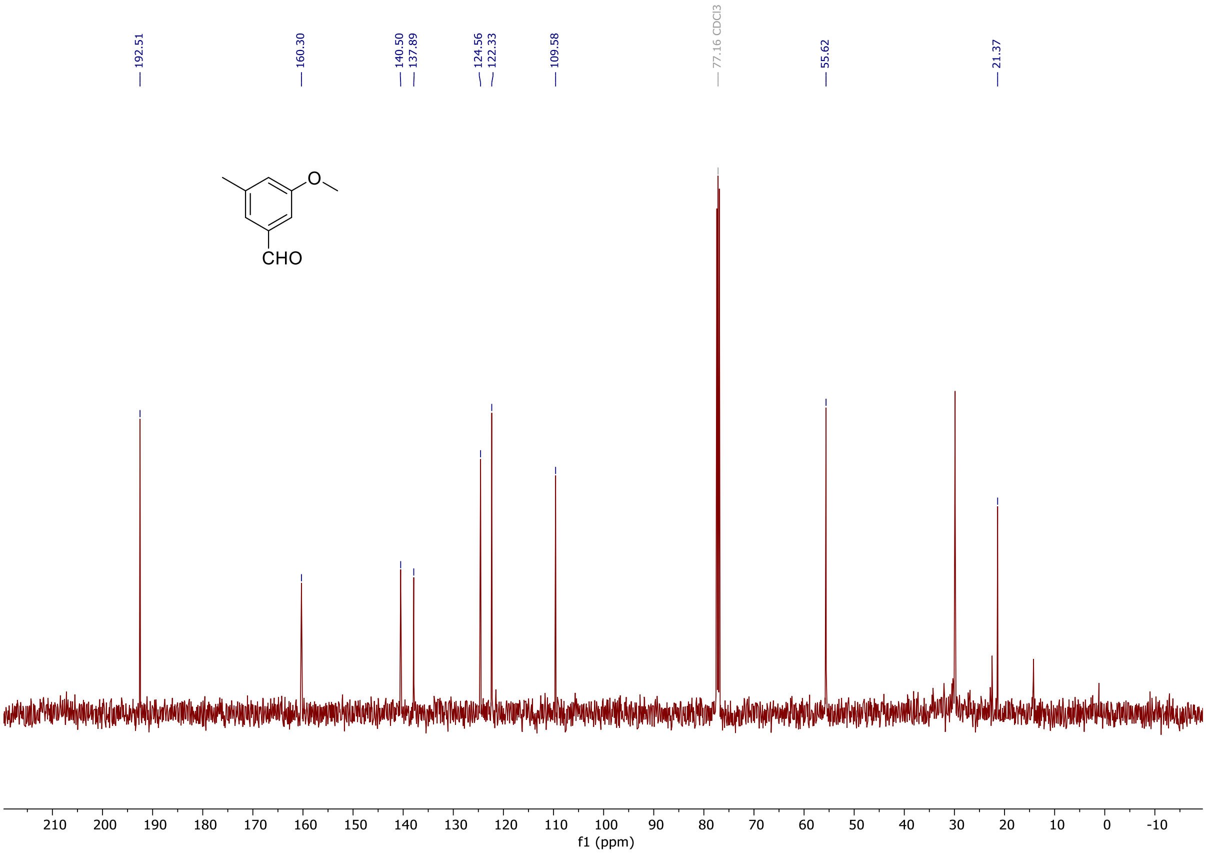

2-methoxy-4-methylbenzaldehyde (4c-α):

^{1}H NMR spectrum (500 MHz) in $CDCl_3$

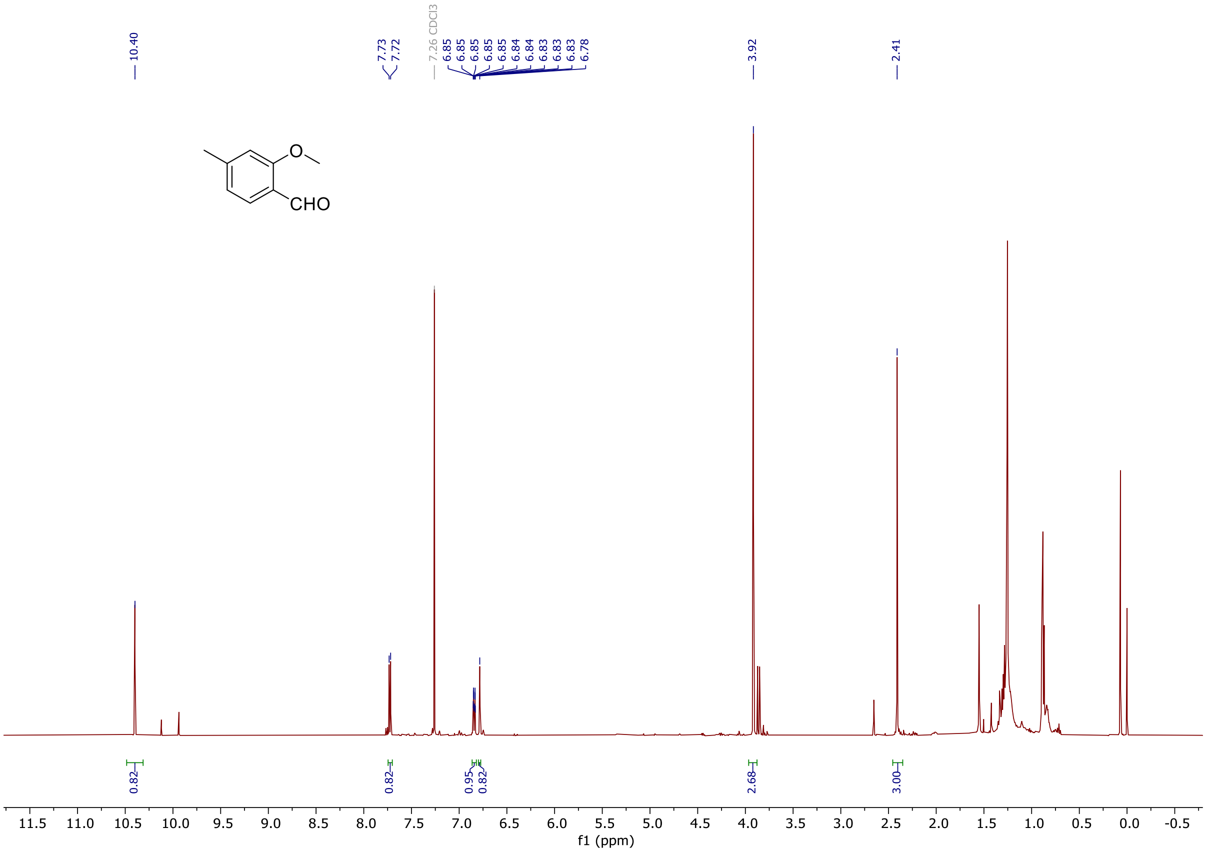

^{13}C NMR spectrum (126 MHz) in $CDCl_3$

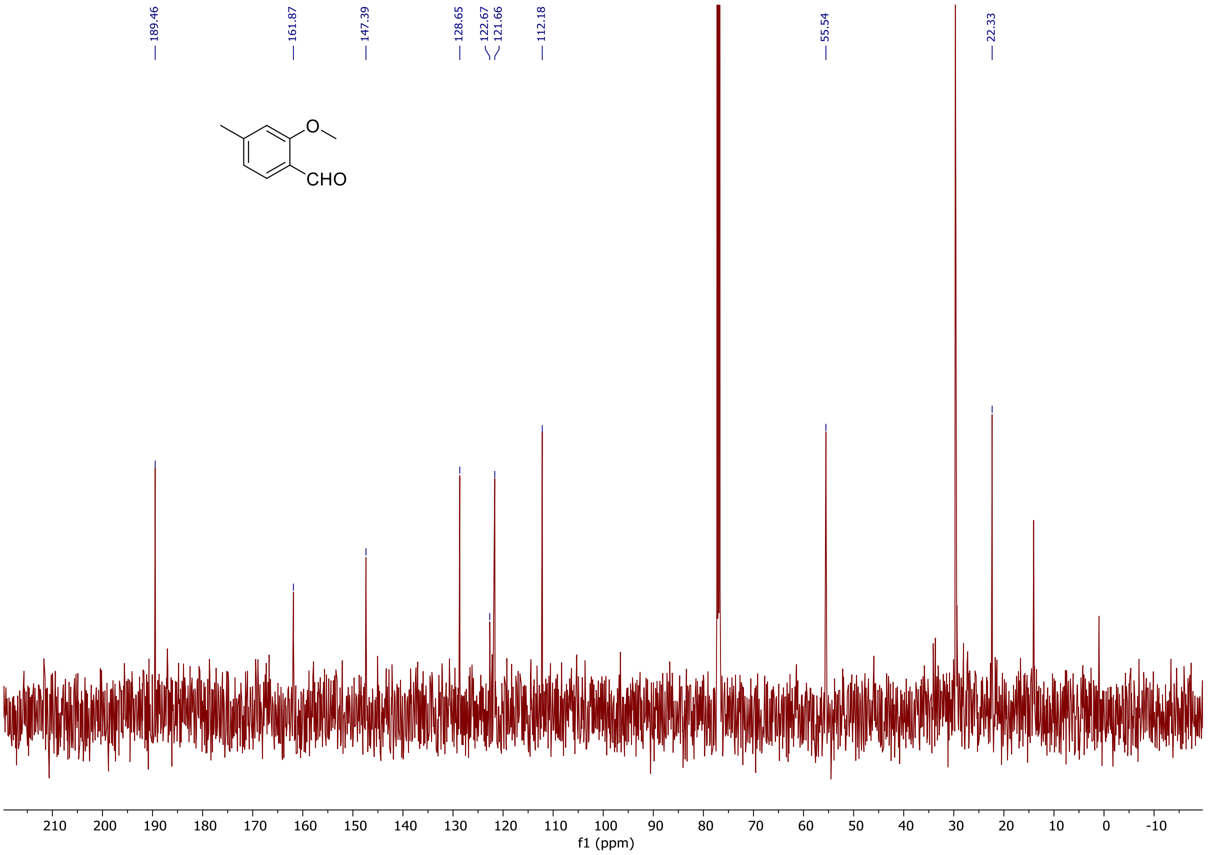

3-methyl-5-((triisopropylsilyl)oxy)benzaldehyde (4d):

¹H NMR spectrum (600 MHz) in $CDCl_3$

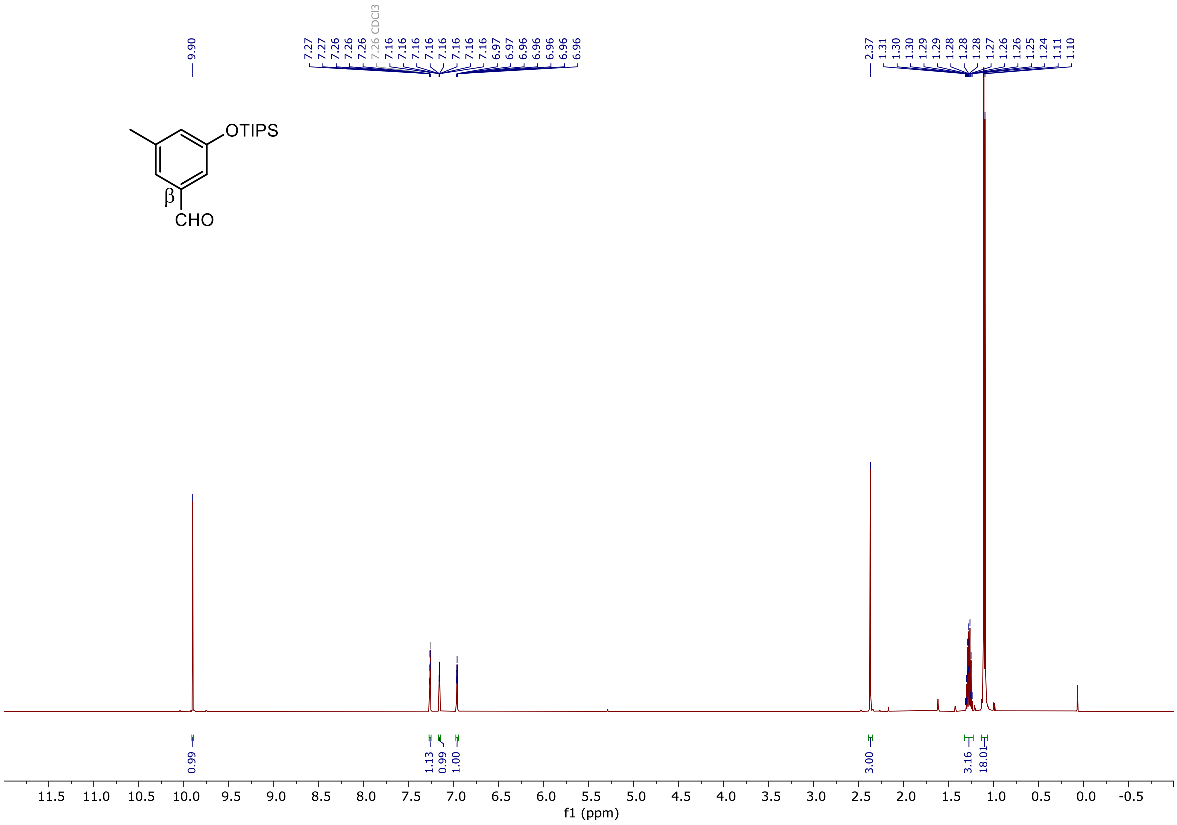

¹³C NMR spectrum (151 MHz) in $CDCl_3$

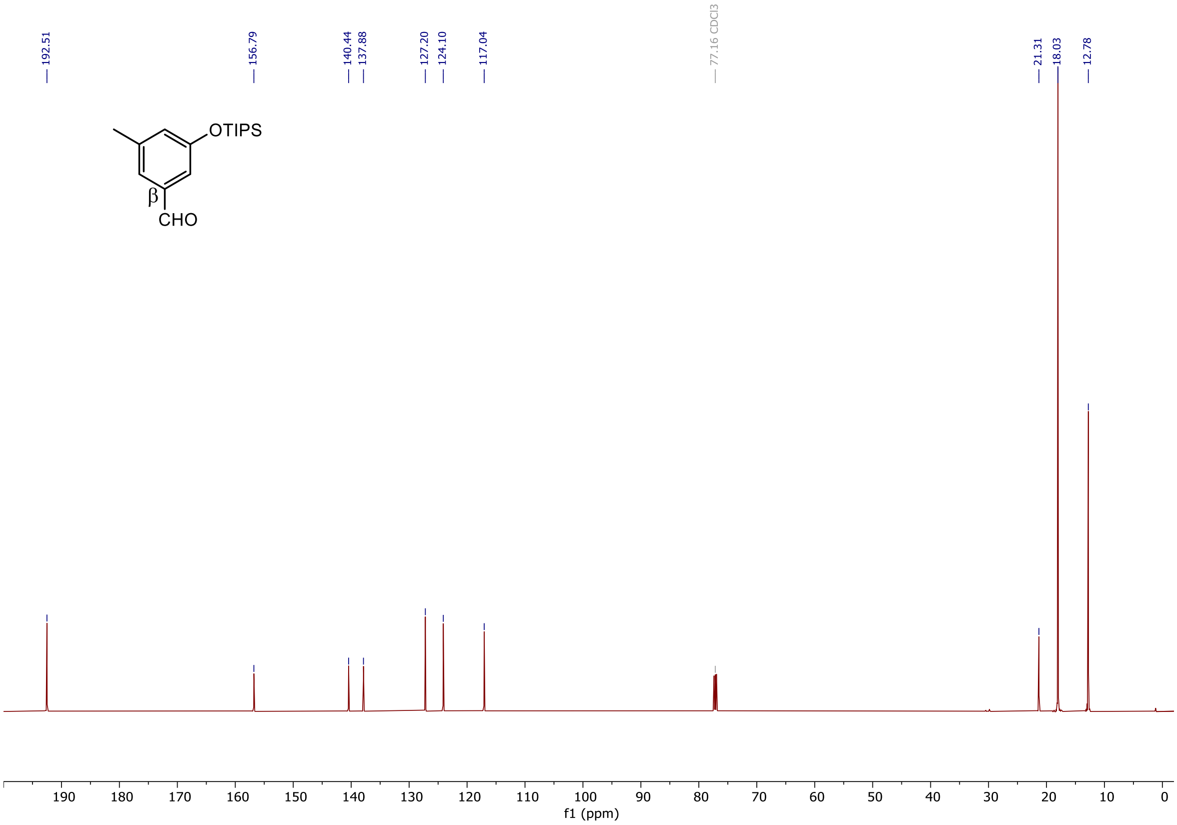

4-acetyl-3,5-dimethylbenzaldehyde (4e):

^{1}H NMR spectrum (500 MHz) in $CDCl_3$

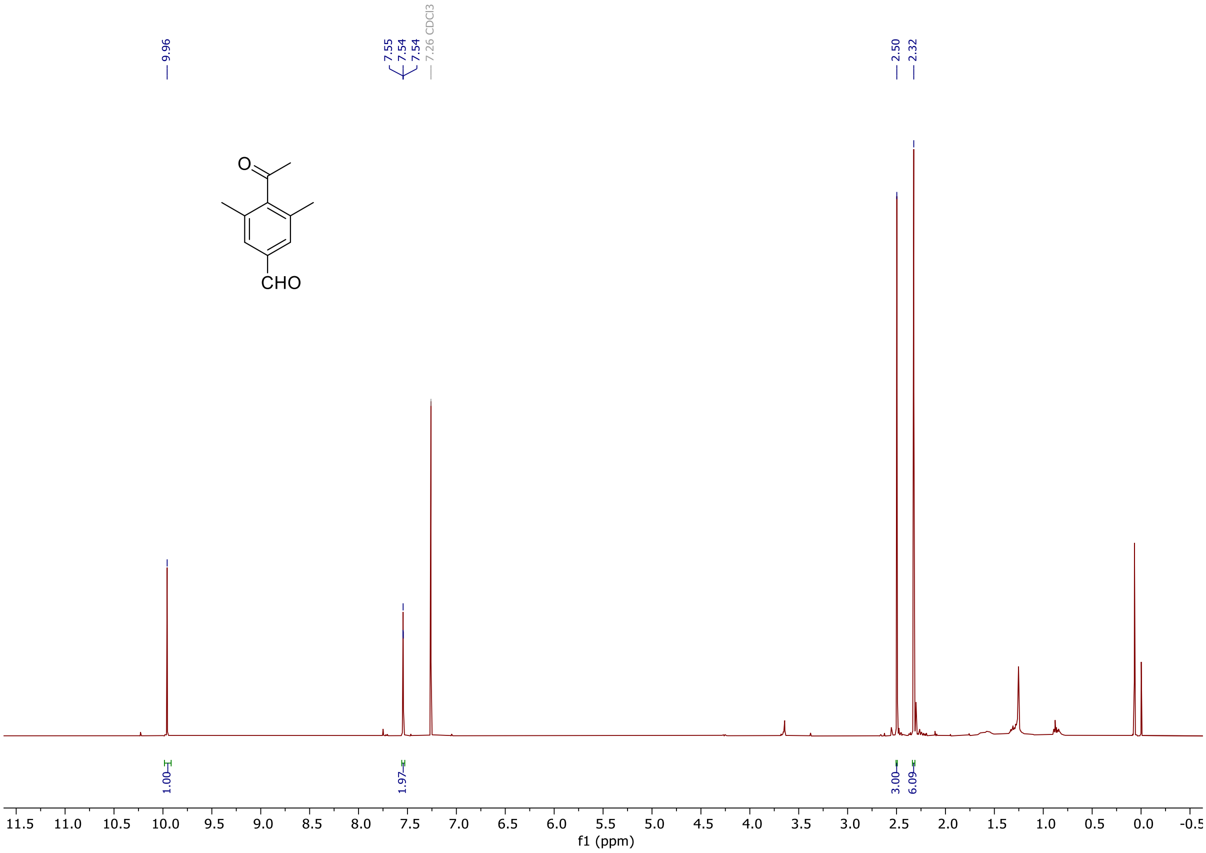

^{13}C NMR spectrum (126 MHz) in $CDCl_3$

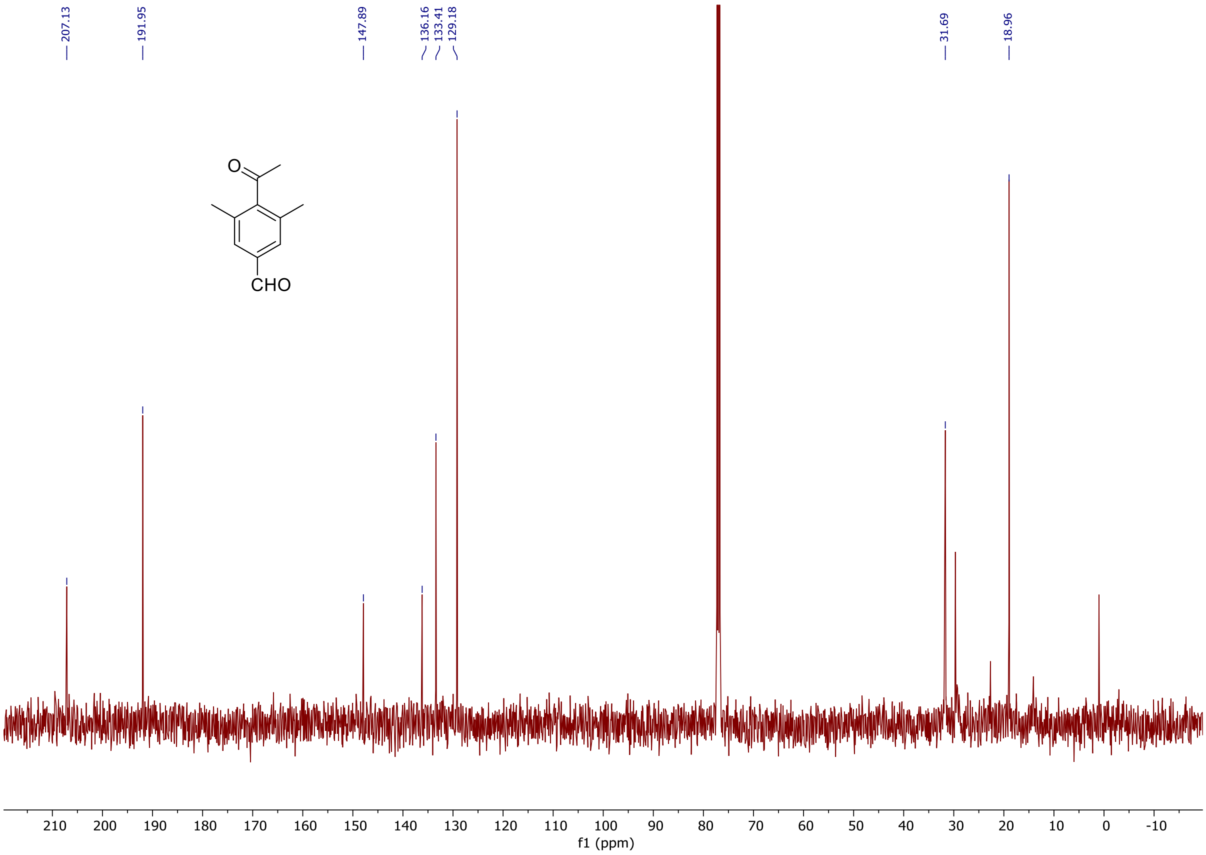

2-chloro-5-methylbenzaldehyde (4f-β)

^{1}H NMR spectrum (600 MHz) in $CDCl_3$

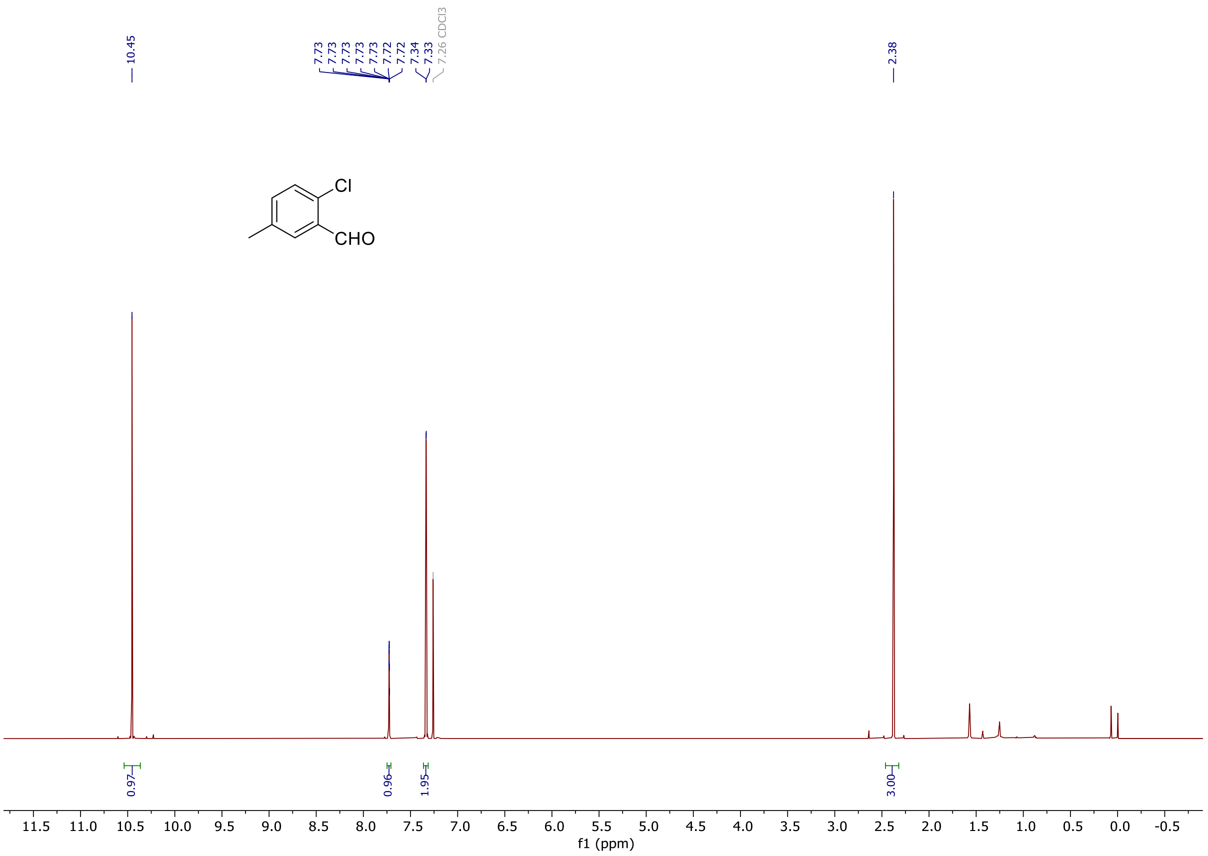

^{13}C NMR spectrum (151 MHz) in $CDCl_3$

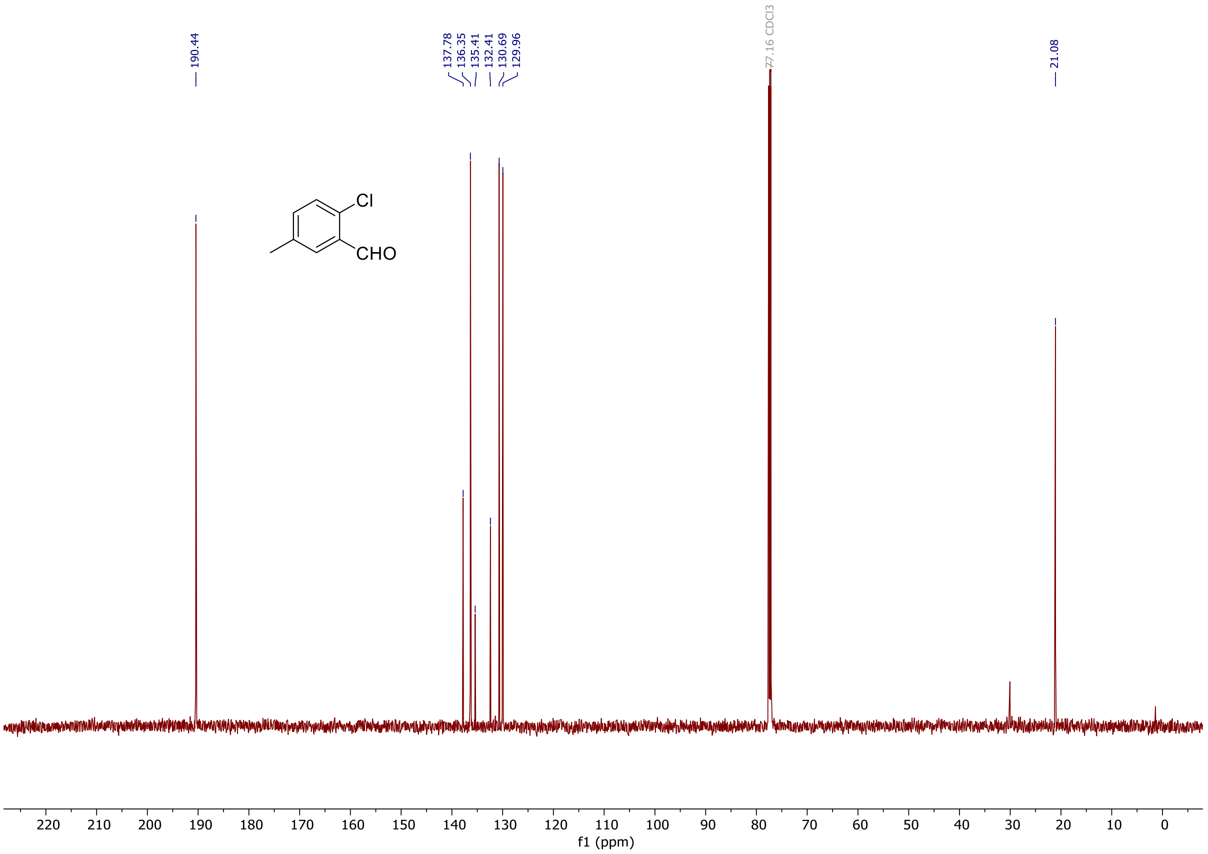

2-chloro-5-methylisophthalaldehyde (4f-di):

^1H NMR spectrum (500 MHz) in $CDCl_3$

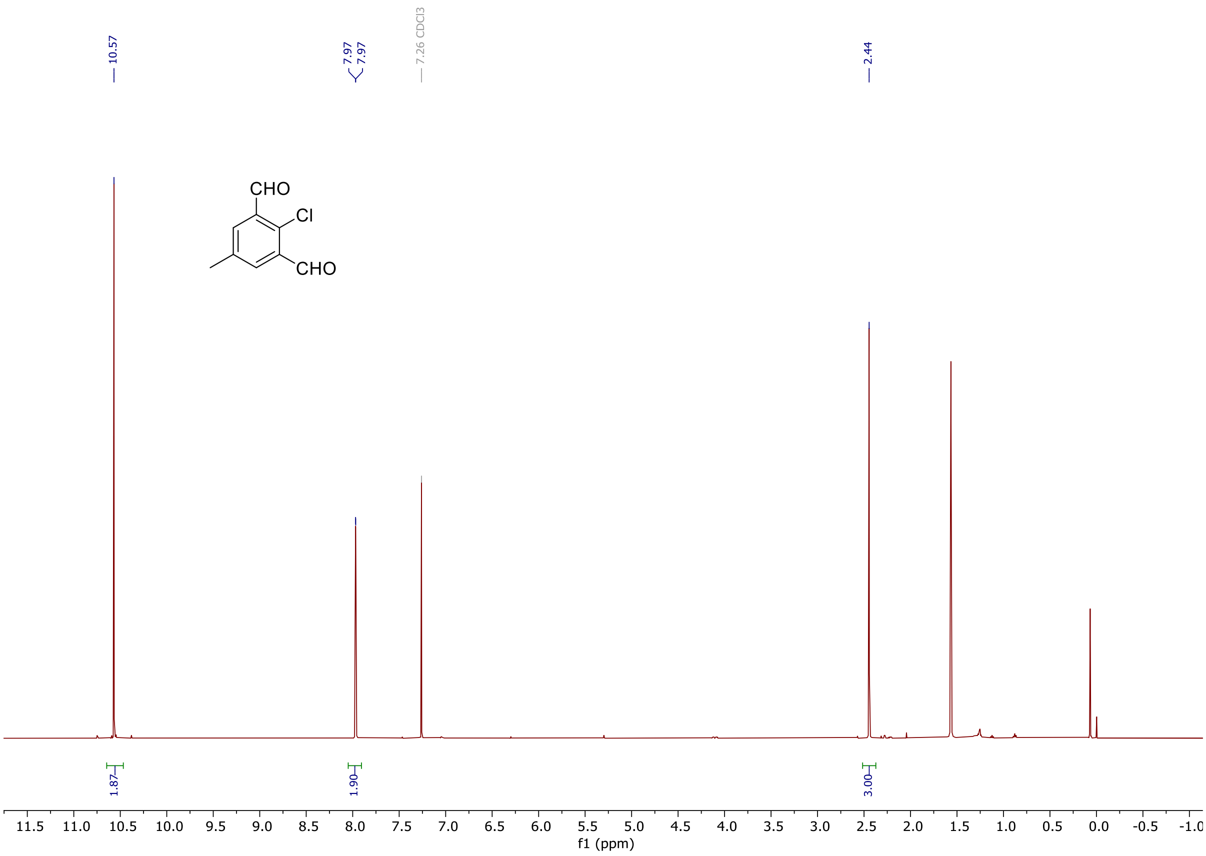

^{13}C NMR spectrum (126 MHz) in $CDCl_3$

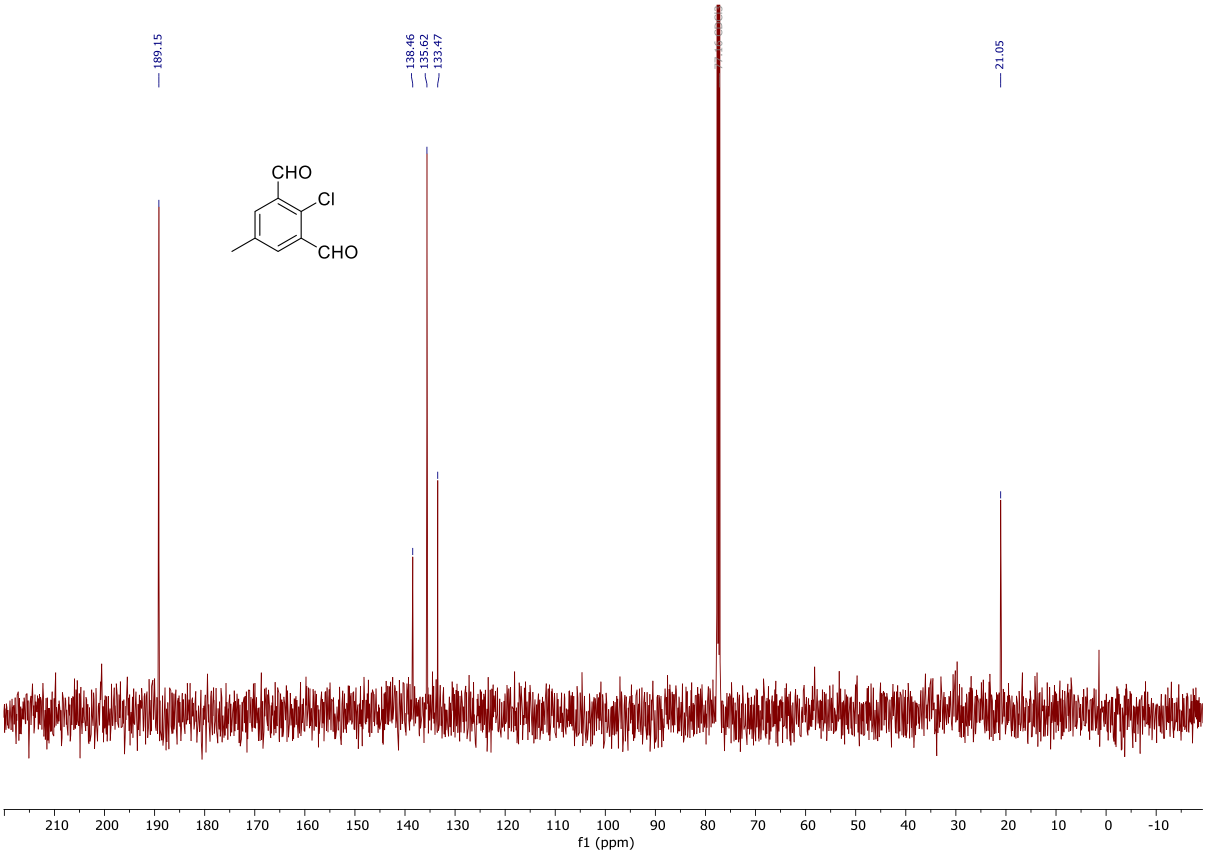

2-chloro-3-methoxy-5-methylbenzaldehyde (4g):

^{1}H NMR spectrum (500 MHz) in CDCl₃

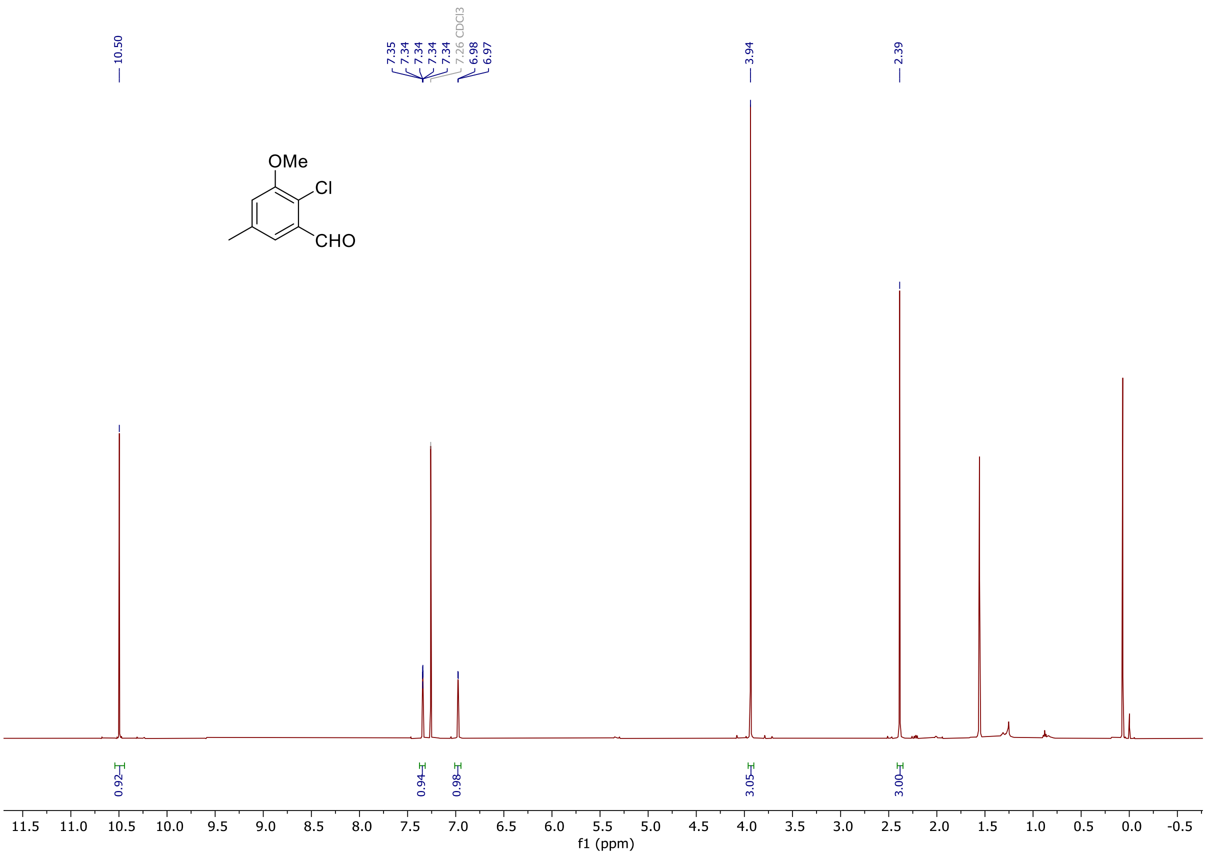

^{13}C NMR spectrum (126 MHz) in CDCl₃

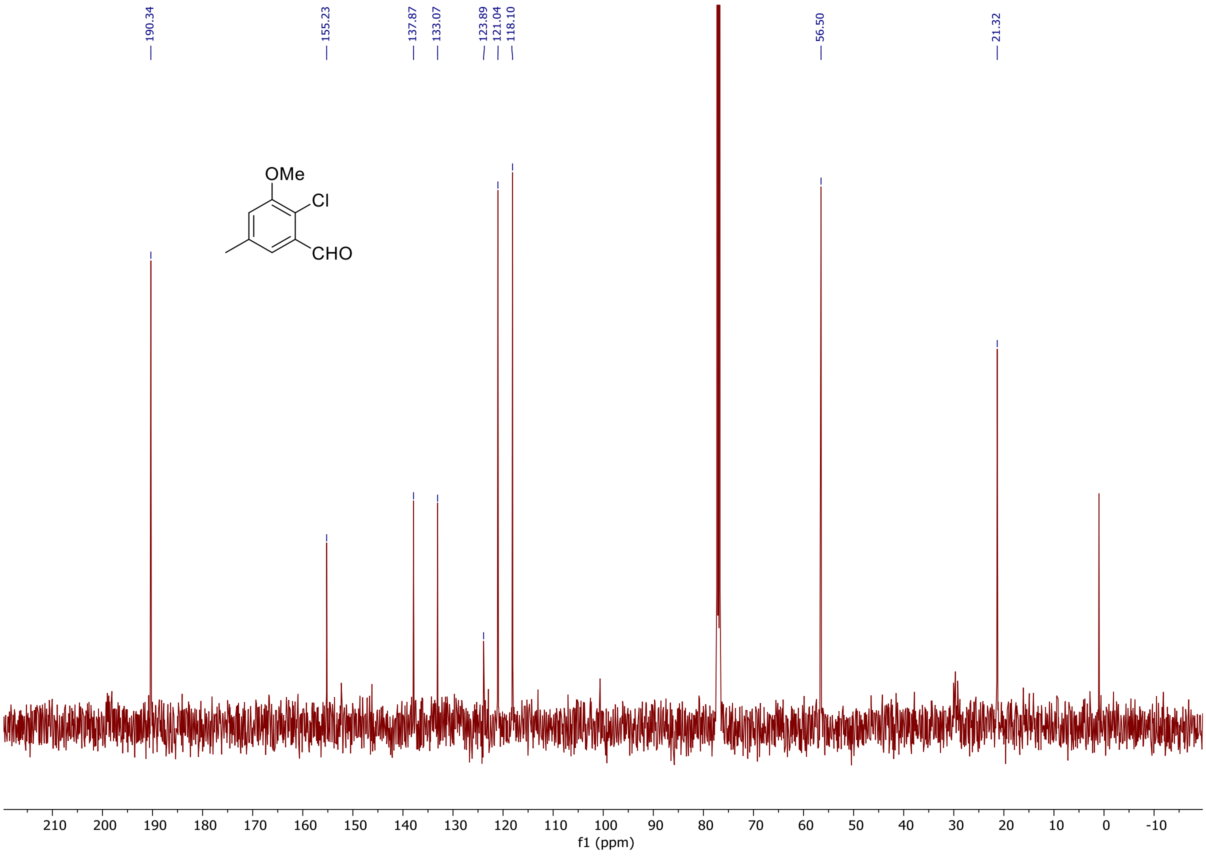

(*S*)-methyl 2-(1,3-dioxoisoindolin-2-yl)-3-(3-formylphenyl)propanoate (4h-*meta*) and (*S*)-methyl 2-(1,3-dioxoisoindolin-2-yl)-3-(4-formylphenyl)propanoate (4h-*para*):

^{1}H NMR spectrum (500 MHz) in $CDCl_3$

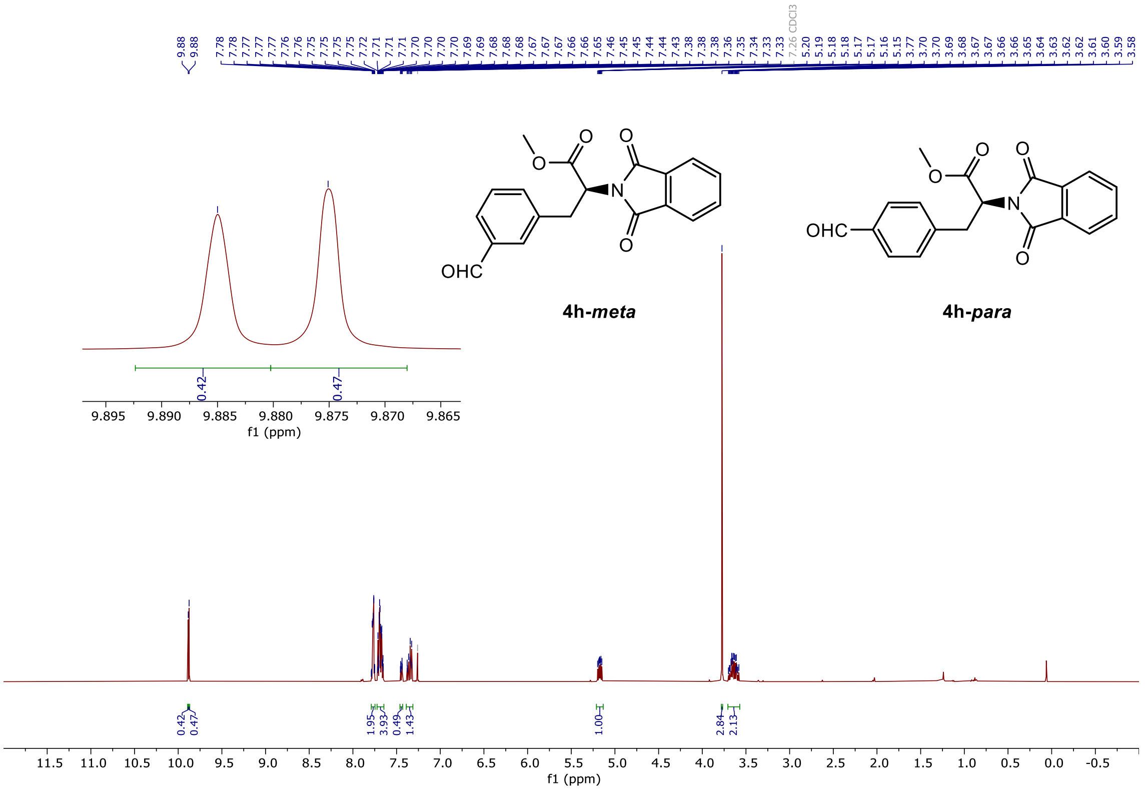

^{13}C NMR spectrum (126 MHz) in $CDCl_3$

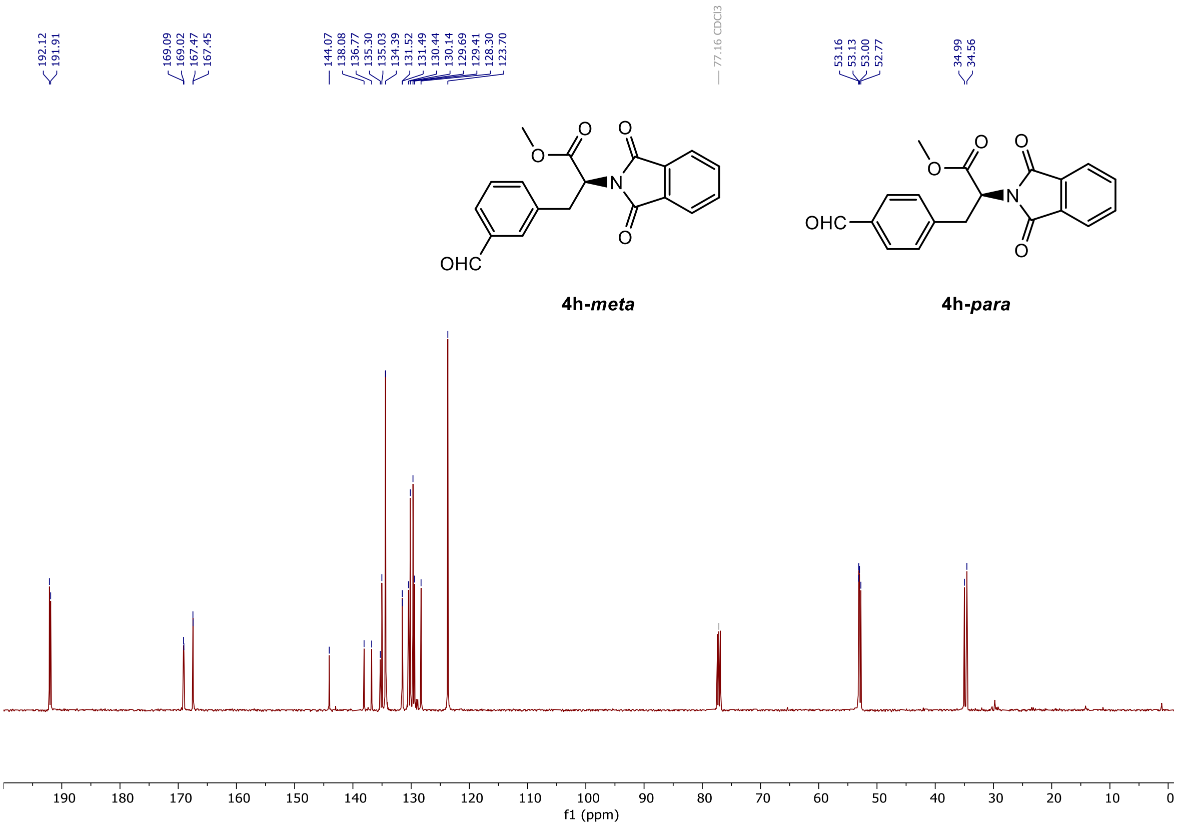

(*R*)-3-((2-oxo-3-propionyloxazolidin-4-yl)methyl)benzaldehyde (4i-*meta*) and (*R*)-4-((2-oxo-3-propionyloxazolidin-4-yl)methyl)benzaldehyde (4i-*para*):

^{1}H NMR spectrum (600 MHz) in $CDCl_3$

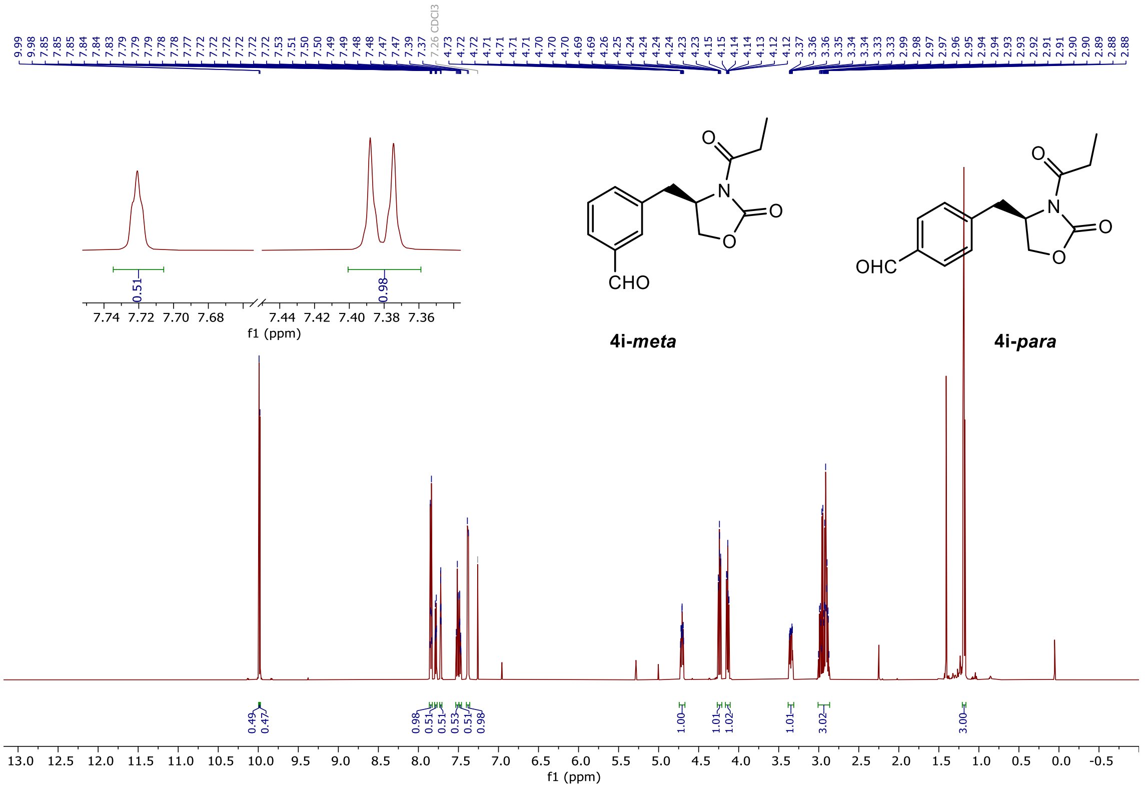

^{13}C NMR spectrum (151 MHz) in $CDCl_3$

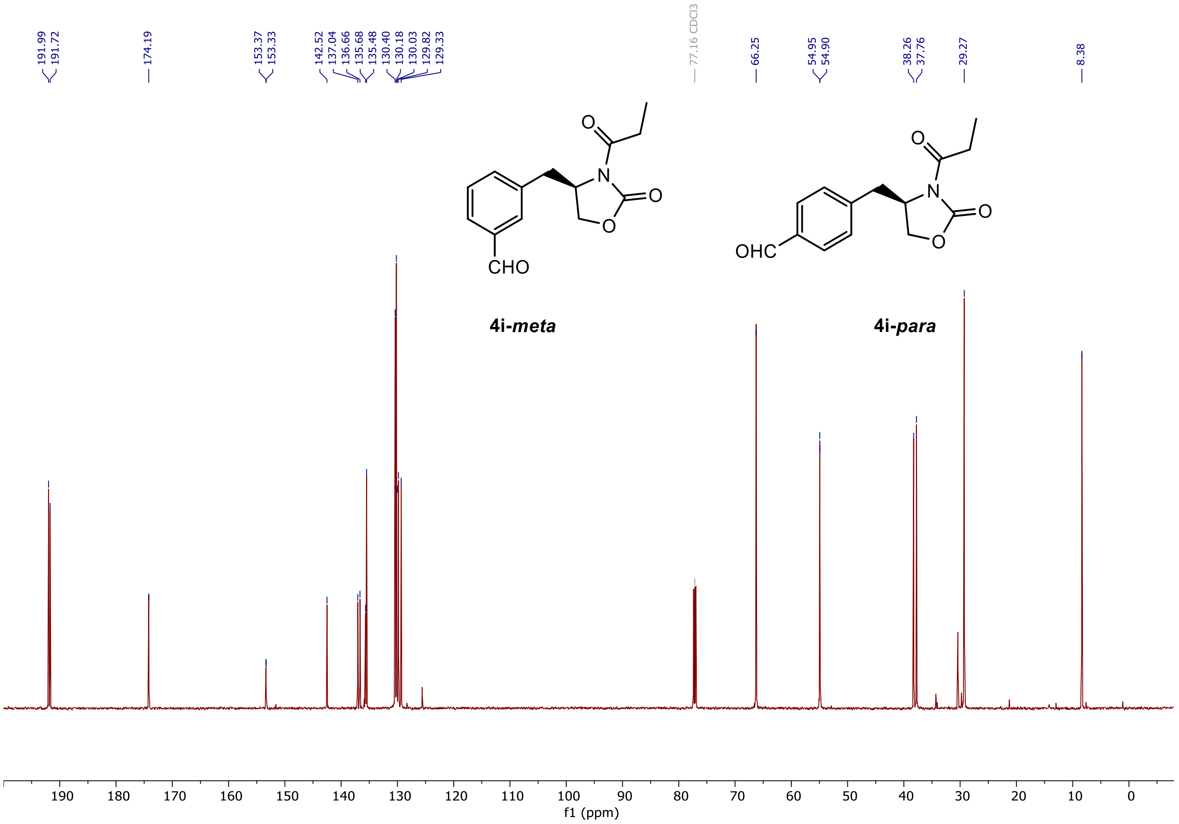

(*R*)-methyl 3-(3-formylphenyl)-2-((1r,4R)-4-isopropylcyclohexanecarboxamido) propanoate (4j-*meta*) and (R)-methyl 3-(4-formylphenyl)-2-((1r,4R)-4-isopropylcyclohexanecarboxamido)propanoate (4j-*para*):

^{1}H NMR spectrum (600 MHz) in $CDCl_3$

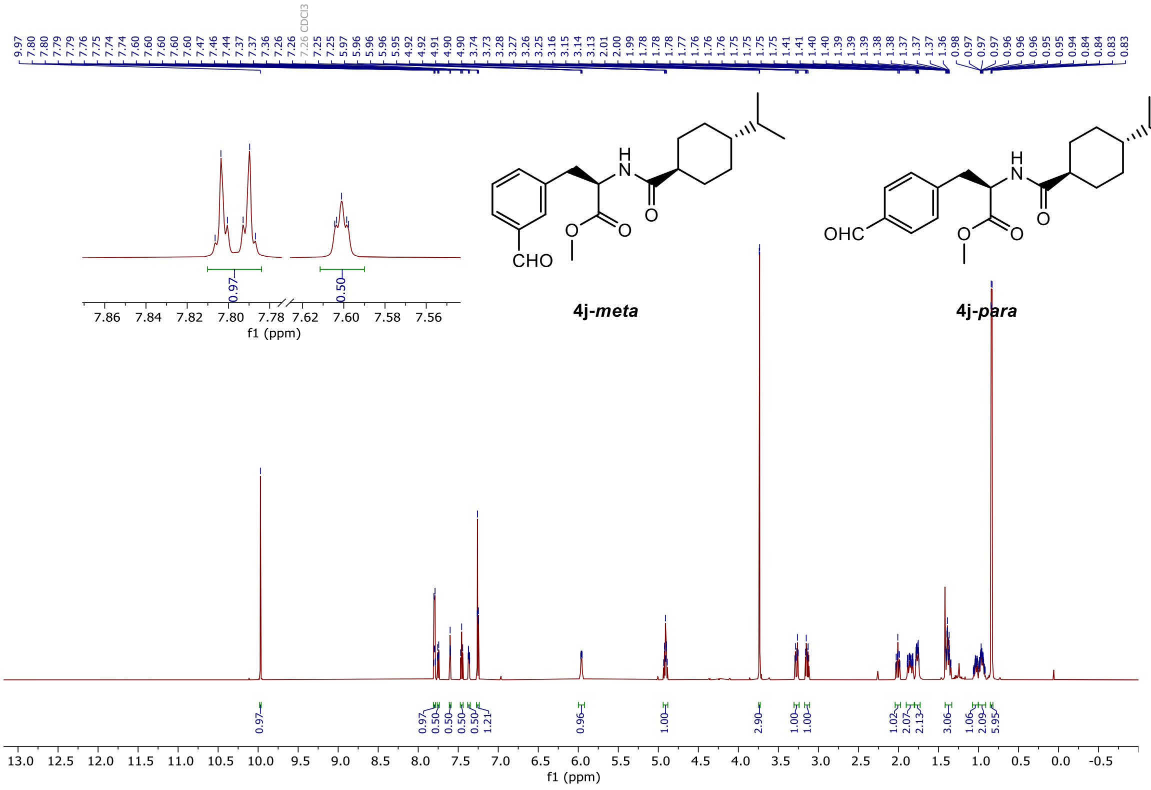

^{13}C NMR spectrum (151 MHz) in $CDCl_3$

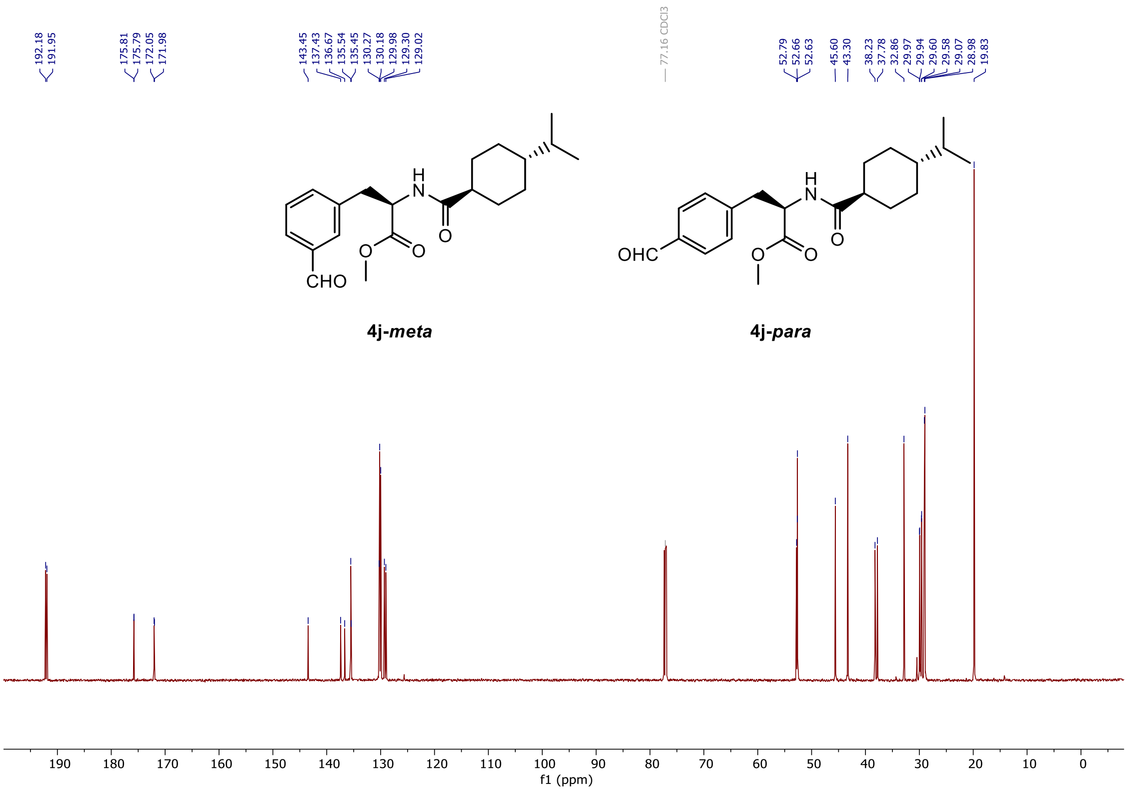

Methyl 2-(2-fluoro-3'-formyl-[1,1'-biphenyl]-4-yl)propanoate (4k-β) and Methyl 2-(2-fluoro-4'-formyl-[1,1'-biphenyl]-4-yl)propanoate (4k-γ):

Characterization of the 4k-β:

^{1}H NMR spectrum (600 MHz) in $CDCl_3$

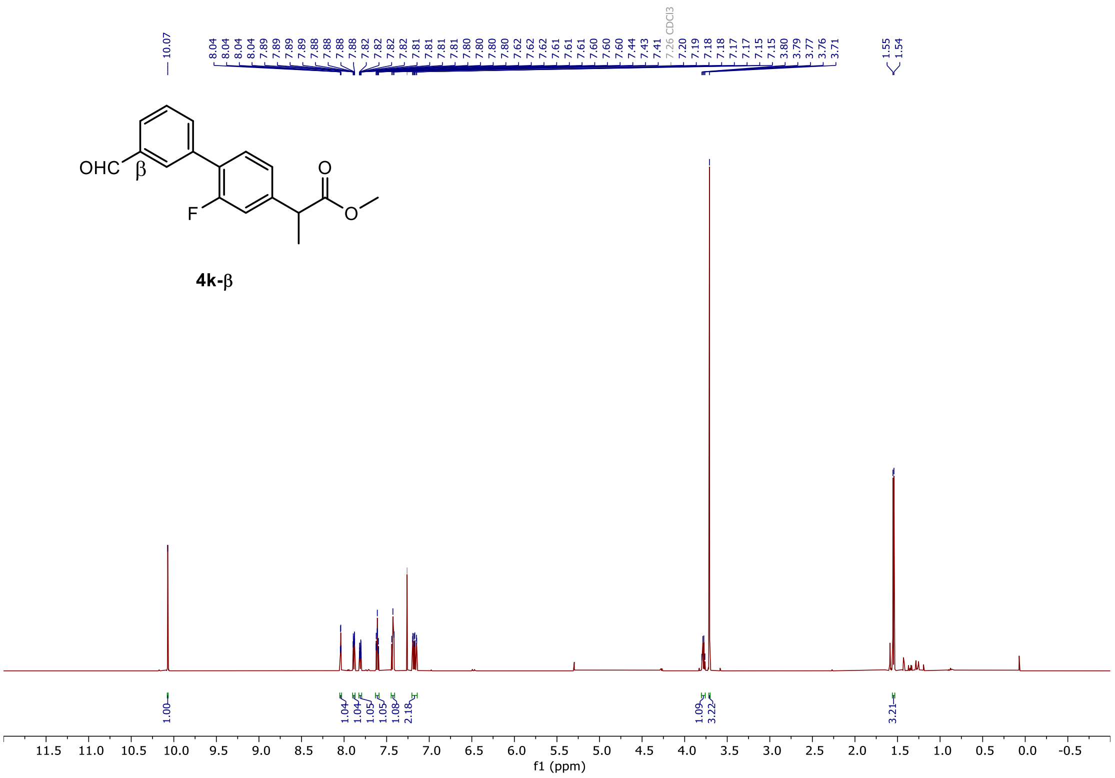

^{13}C NMR spectrum (151 MHz) in $CDCl_3$

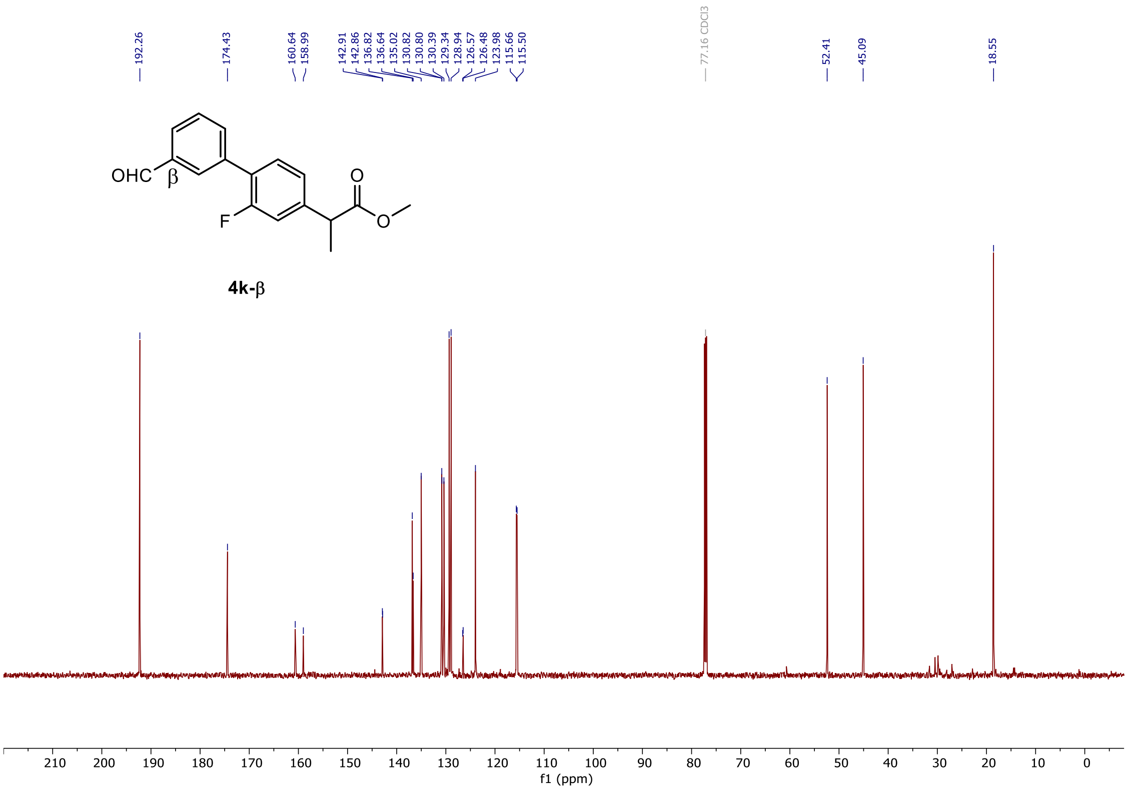

^{19}F NMR spectrum (471 MHz) in $CDCl_3$

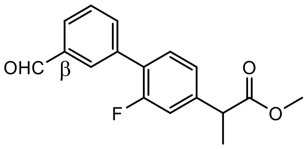

4k-β

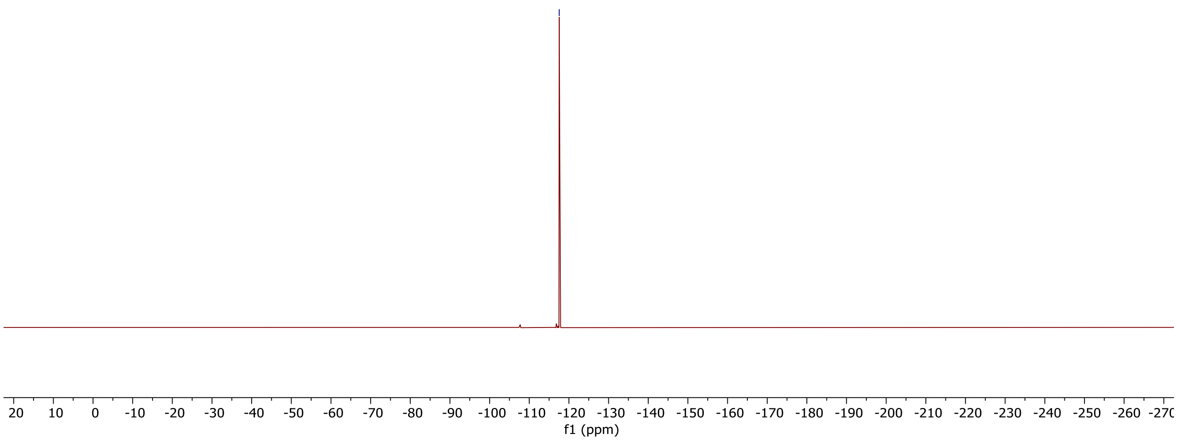

Characterization of the 4k-β and 4k-γ mixture:

^{1}H NMR spectrum (600 MHz) in $CDCl_3$

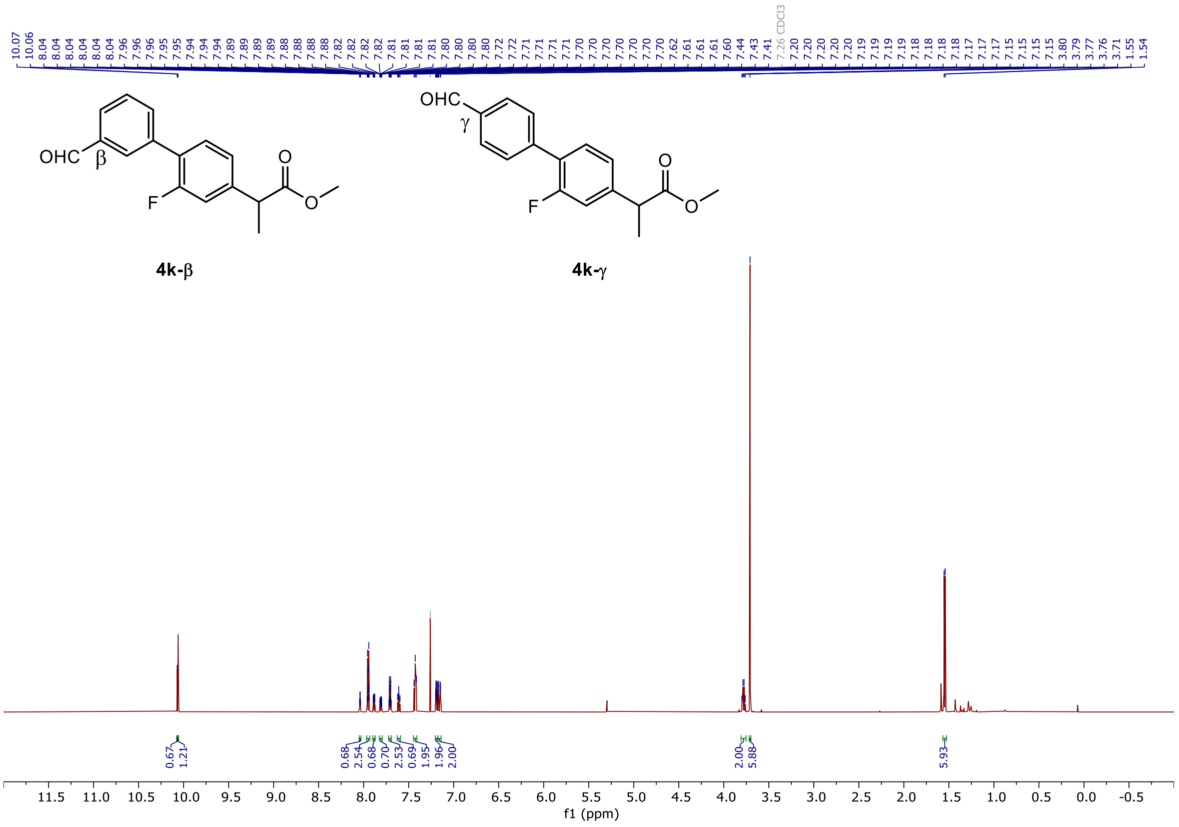

^{13}C NMR spectrum (151 MHz) in $CDCl_3$

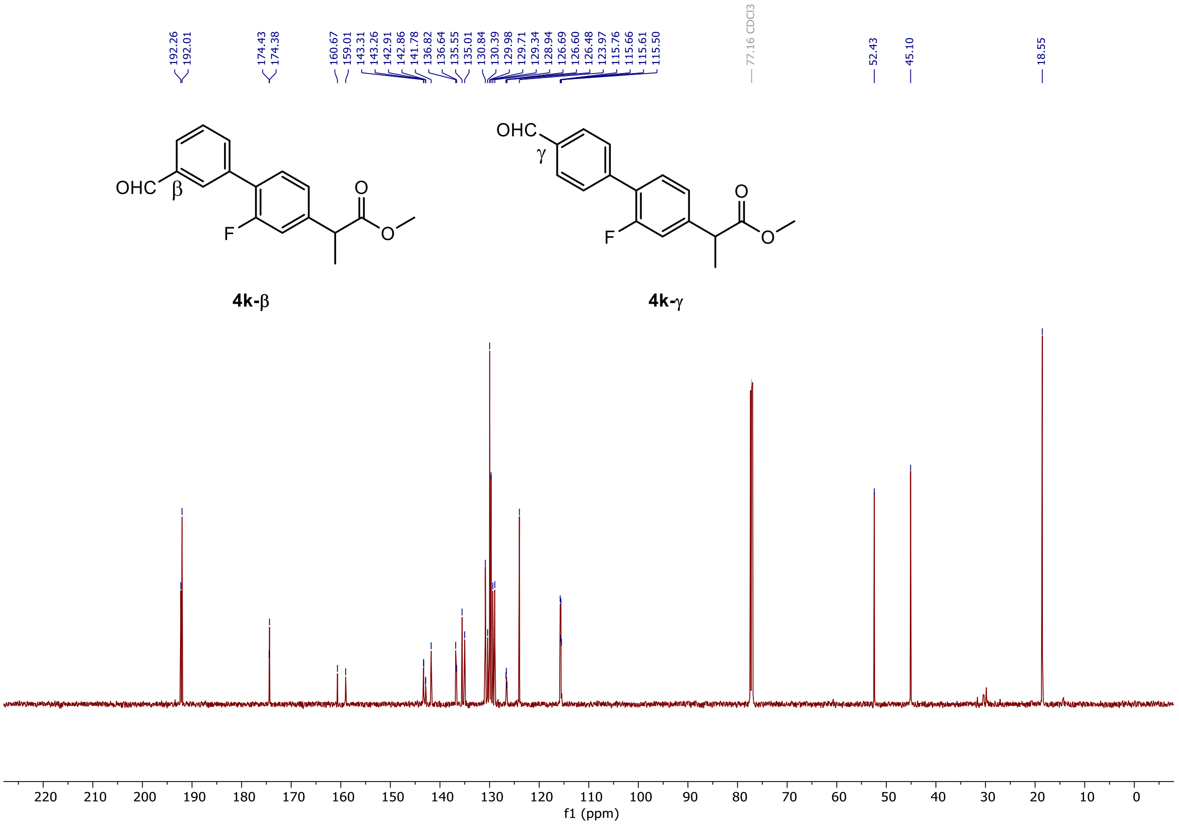

^{19}F NMR spectrum (471 MHz) in $CDCl_3$

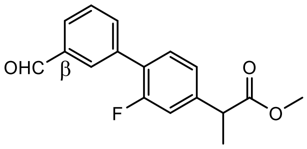

4k-β

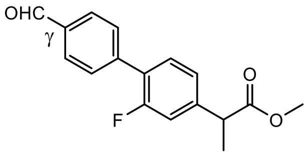

4k-γ

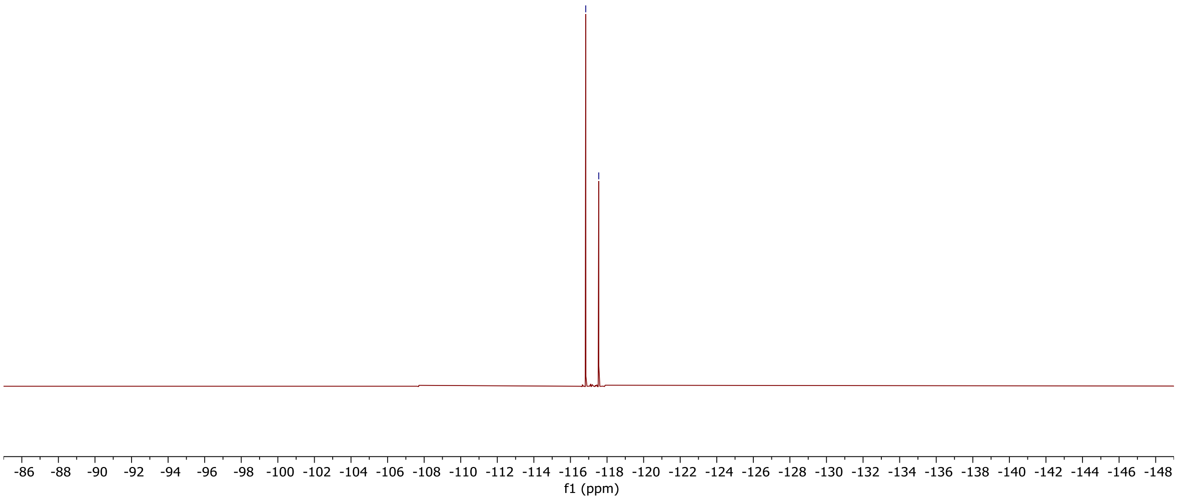

Methyl 2-(3-(3-formylbenzoyl)phenyl)propanoate (4l-β), methyl 2-(3-benzoyl-5-formylphenyl)propanoate (4l-β′) and methyl 2-(3-(4-formylbenzoyl)phenyl)propanoate (4l-γ):

^{1}H NMR spectrum (600 MHz) in $CDCl_3$

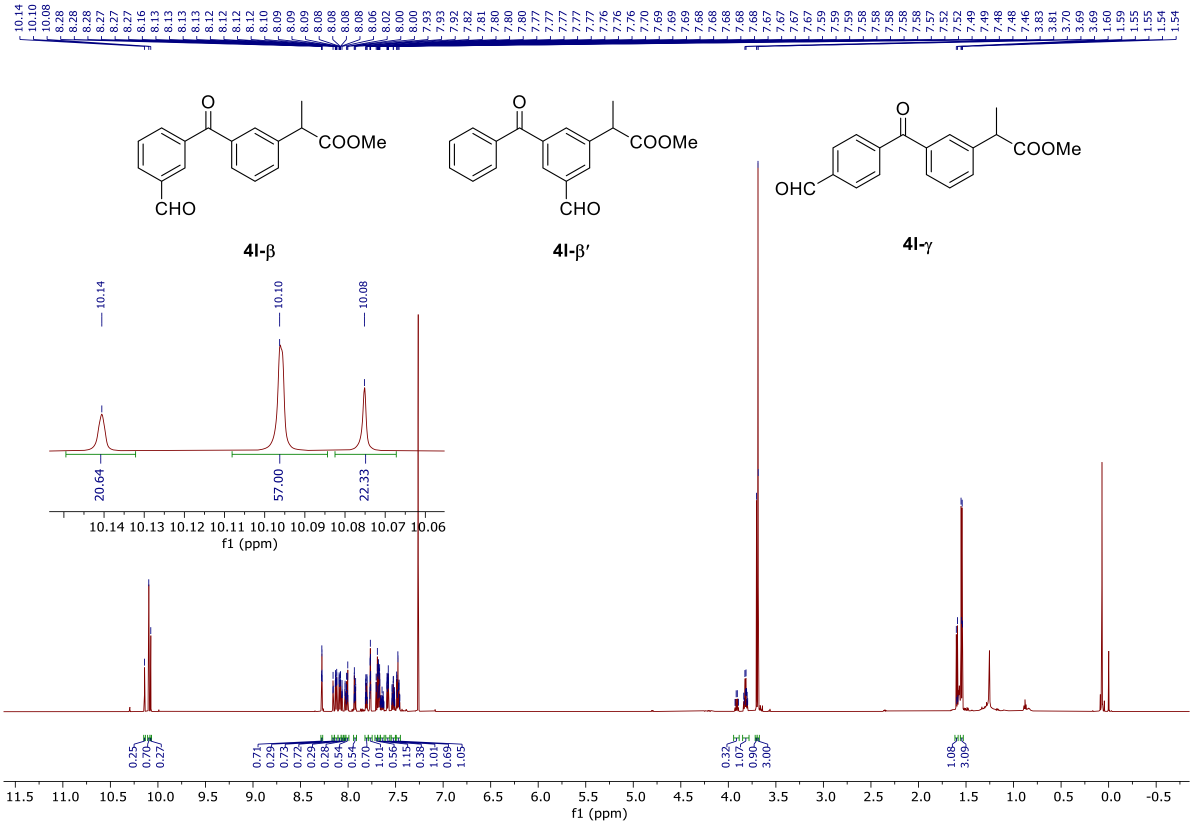

^{13}C NMR spectrum (151 MHz) in $CDCl_3$

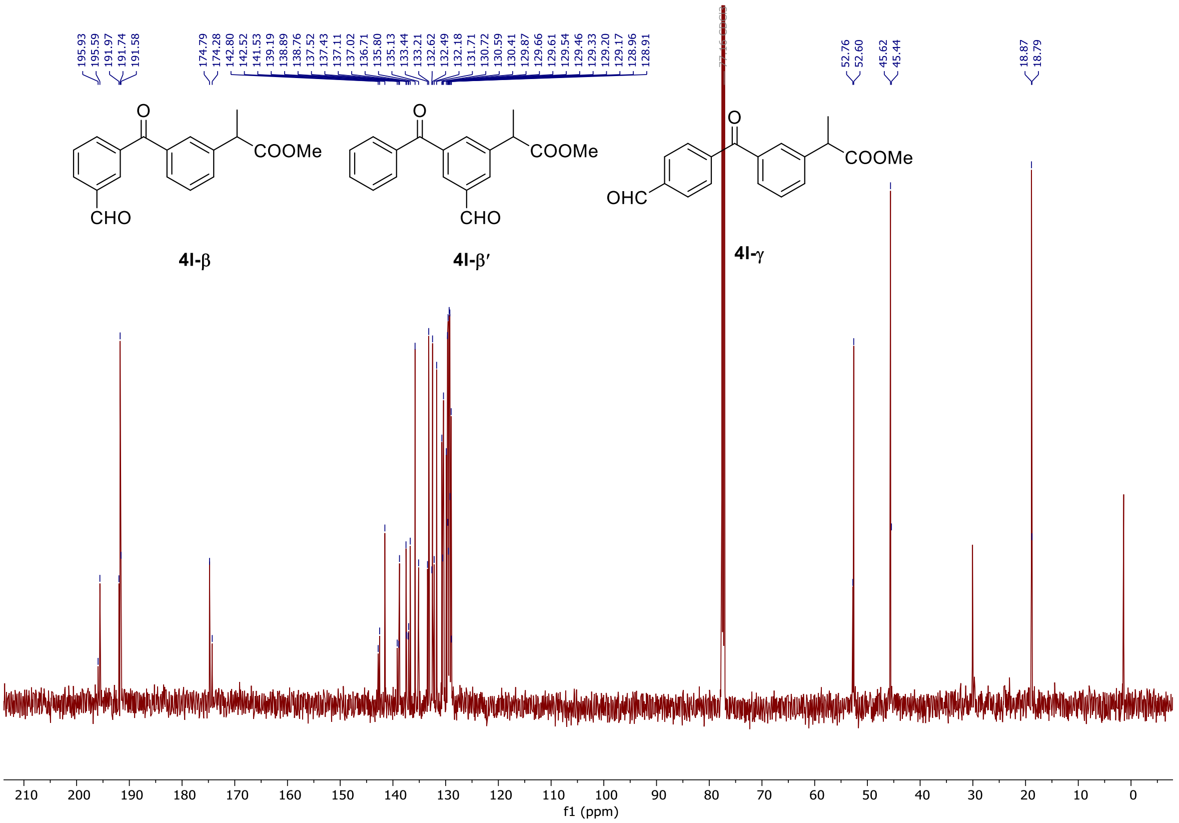

Methyl 3-(3-formyl-5-methylphenyl)propanoate (4m):

^{1}H NMR spectrum (500 MHz) in $CDCl_3$

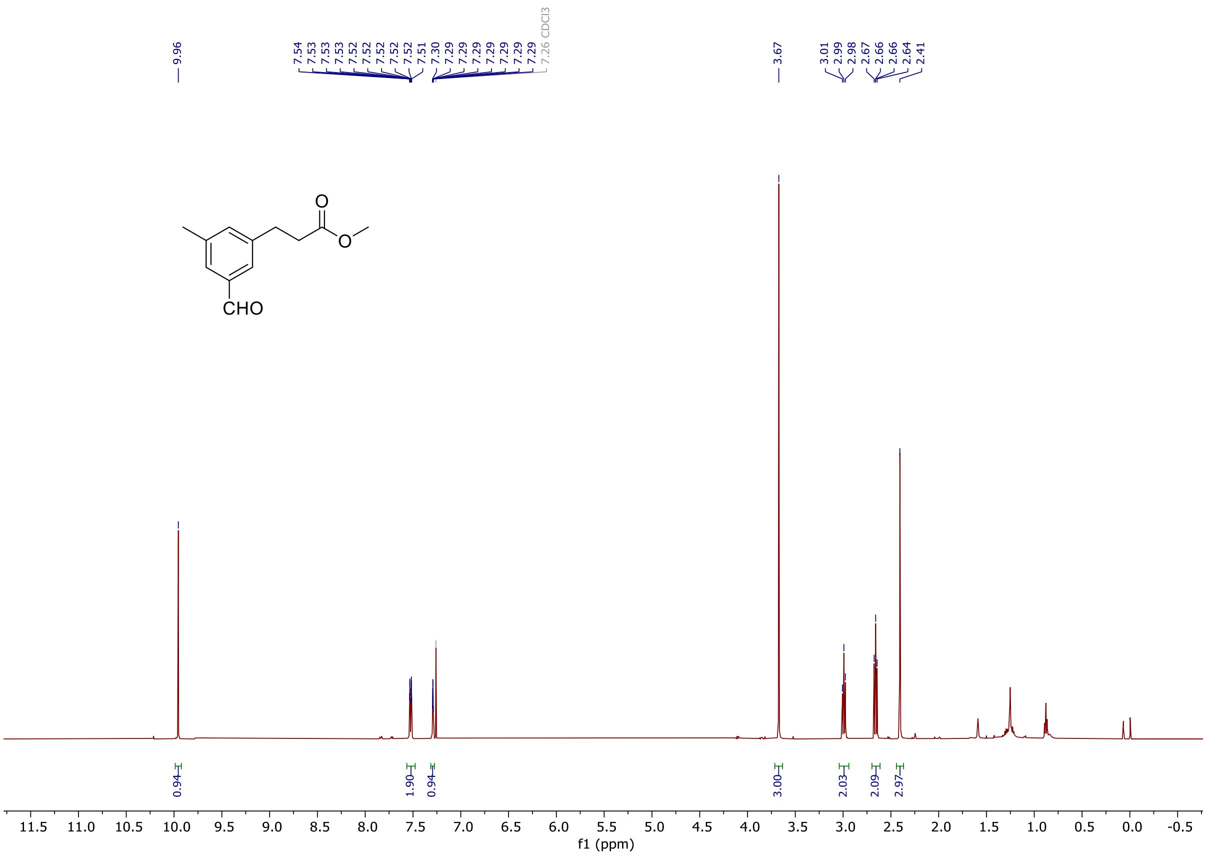

^{13}C NMR spectrum (126 MHz) in $CDCl_3$

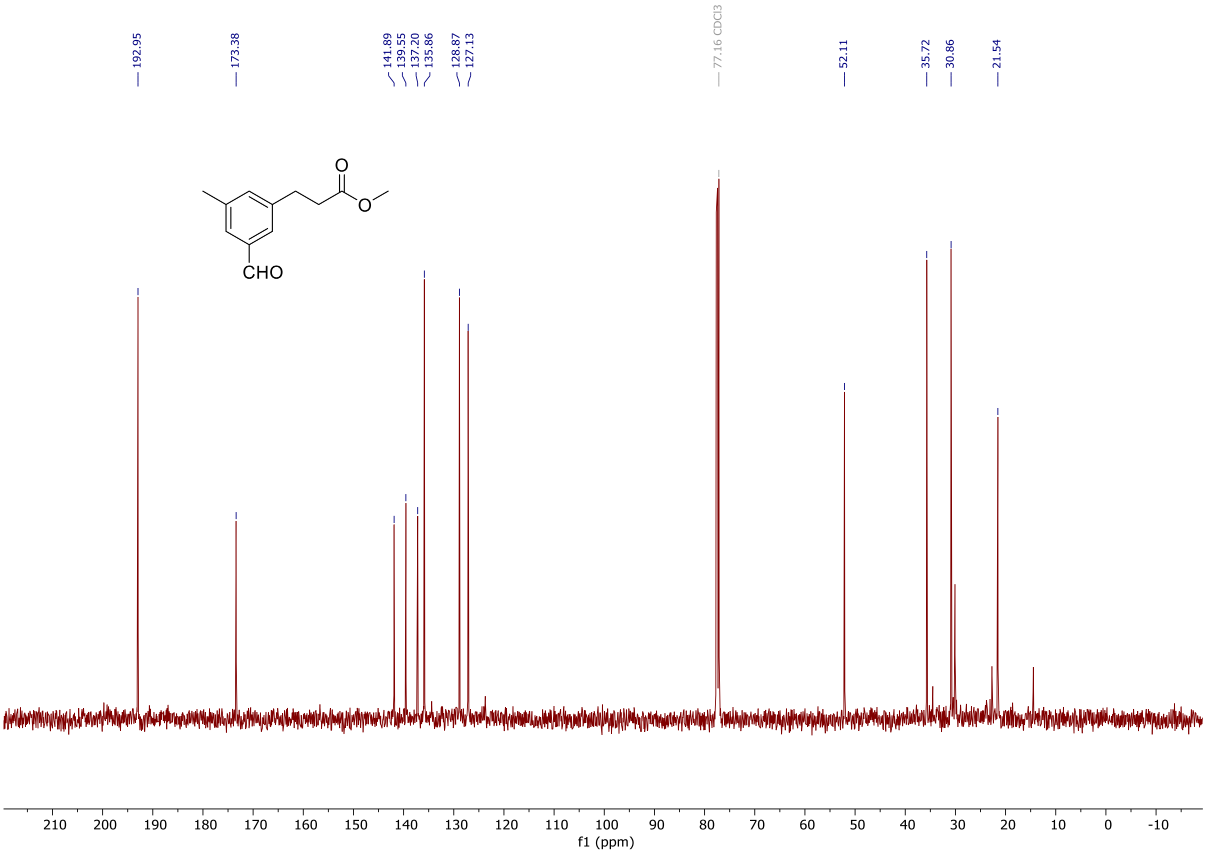

Methyl 3-formyl-5,6,7,8-tetrahydronaphthalene-1-carboxylate (4n):

^{1}H NMR spectrum (600 MHz) in $CDCl_3$

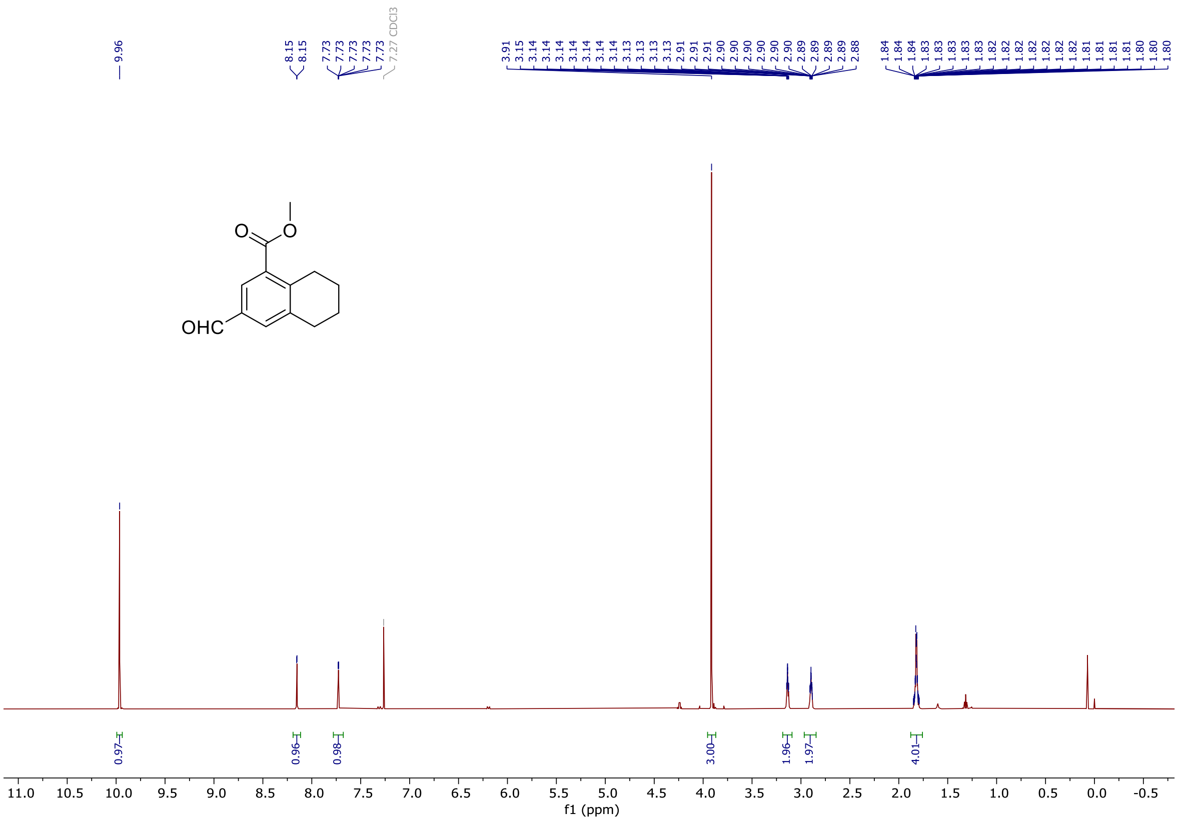

^{13}C NMR spectrum (151 MHz) in $CDCl_3$

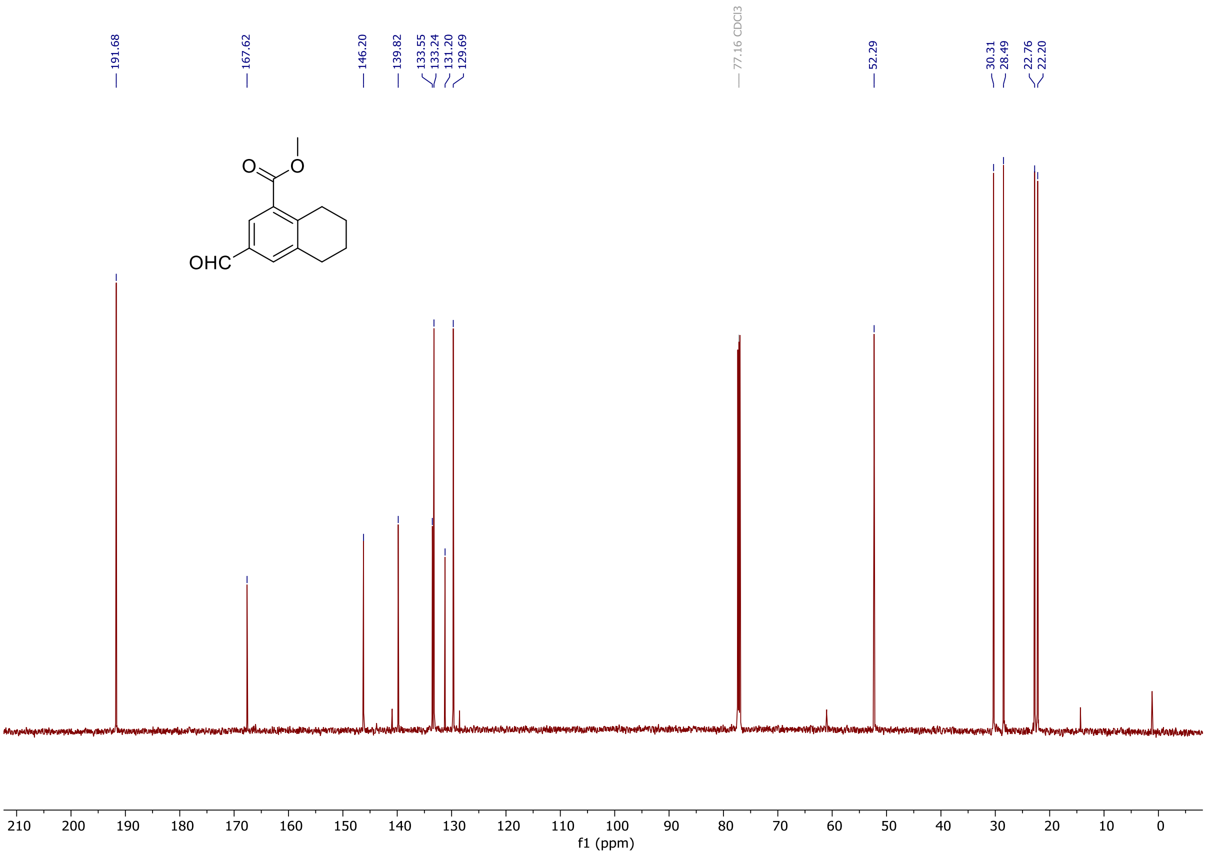

3-((2-isopropyl-5-methylphenoxy)methyl)-5-methylbenzaldehyde (4o)

^{1}H NMR spectrum (600 MHz) in $CDCl_3$

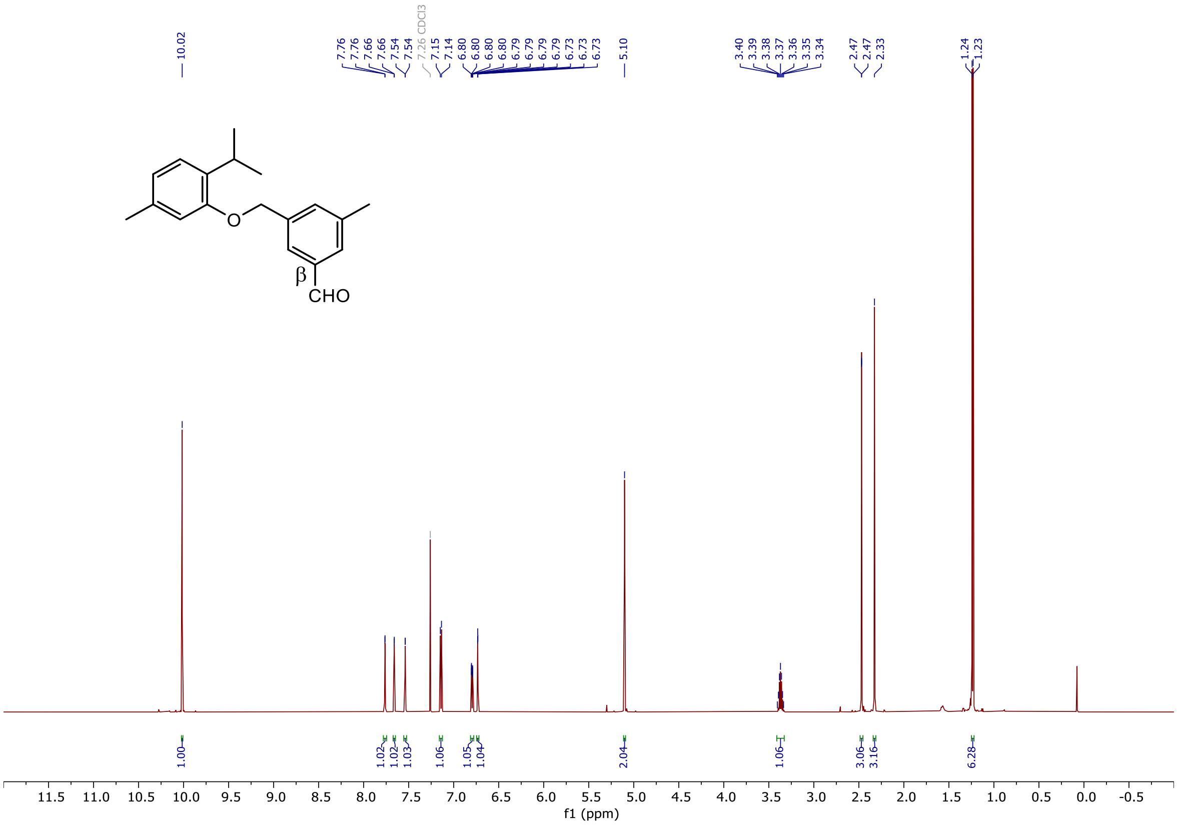

^{13}C NMR spectrum (151 MHz) in $CDCl_3$

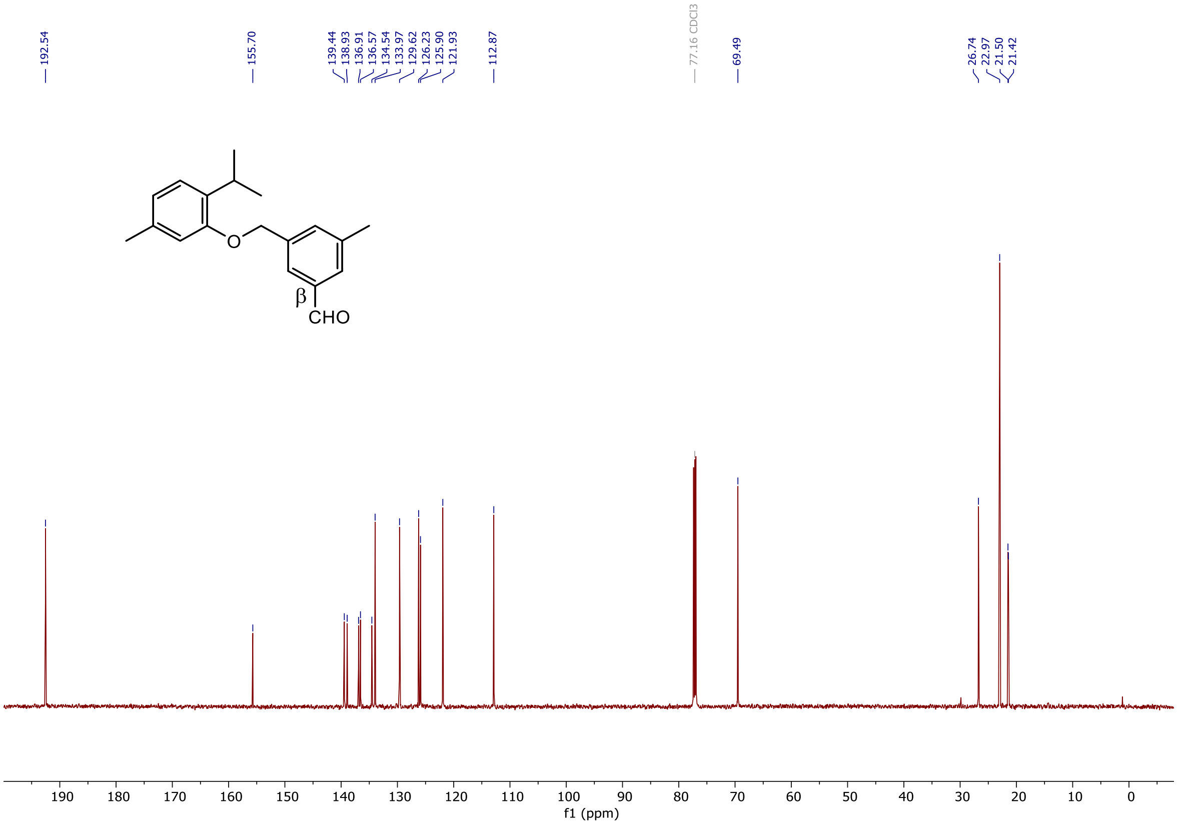

Methyl 5-formyl-2-methyl-4'-(trifluoromethoxy)-[1,1'-biphenyl]-3-carboxylate (4p):

^{1}H NMR spectrum (500 MHz) in CDCl$_3$

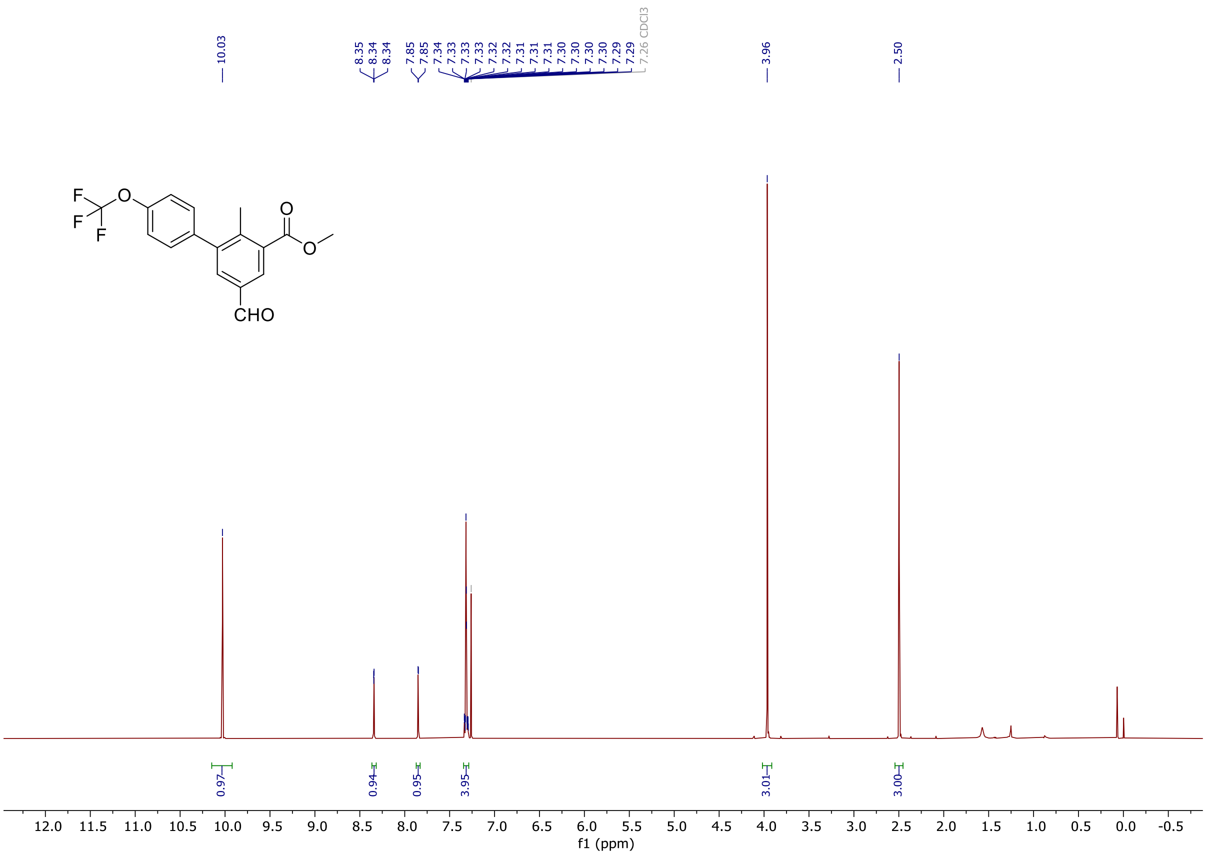

^{13}C NMR spectrum (151 MHz) in CDCl$_3$

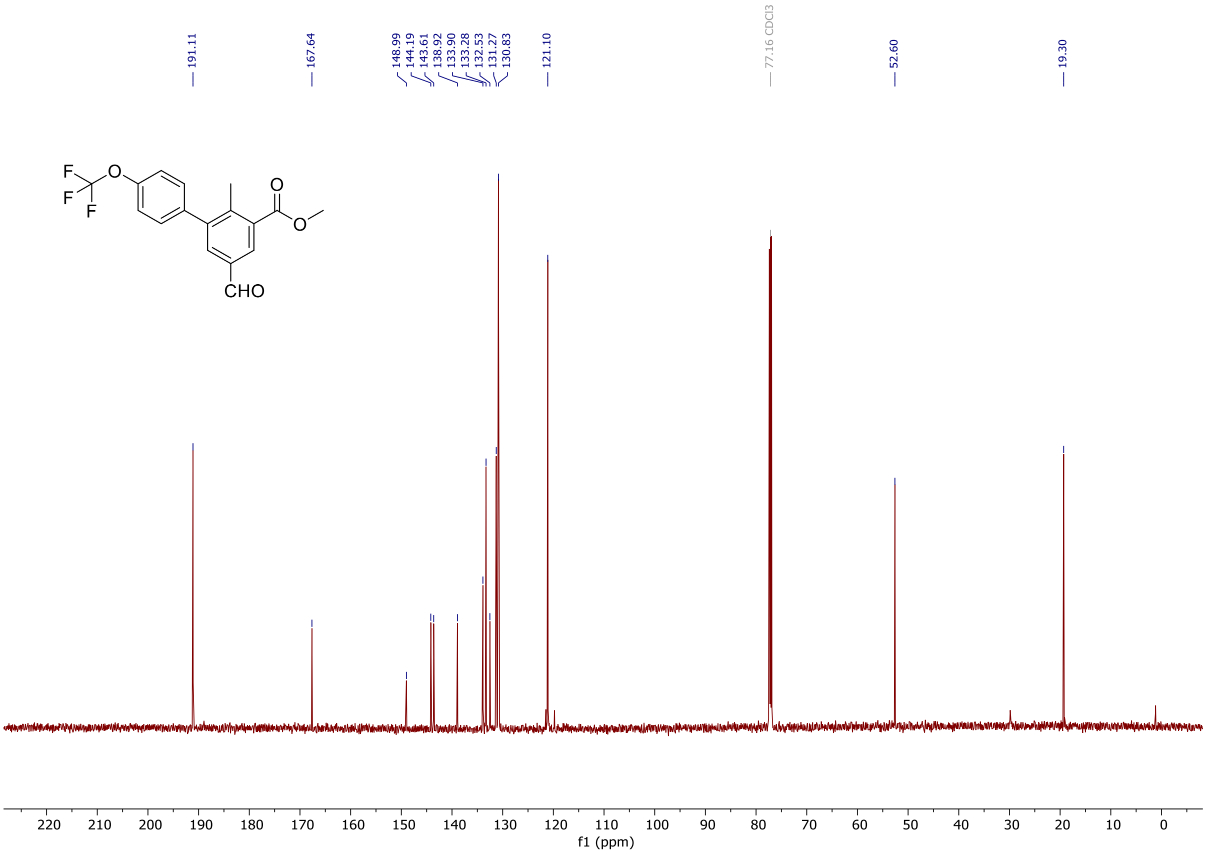

^{19}F NMR spectrum (471 MHz) in $CDCl_3$

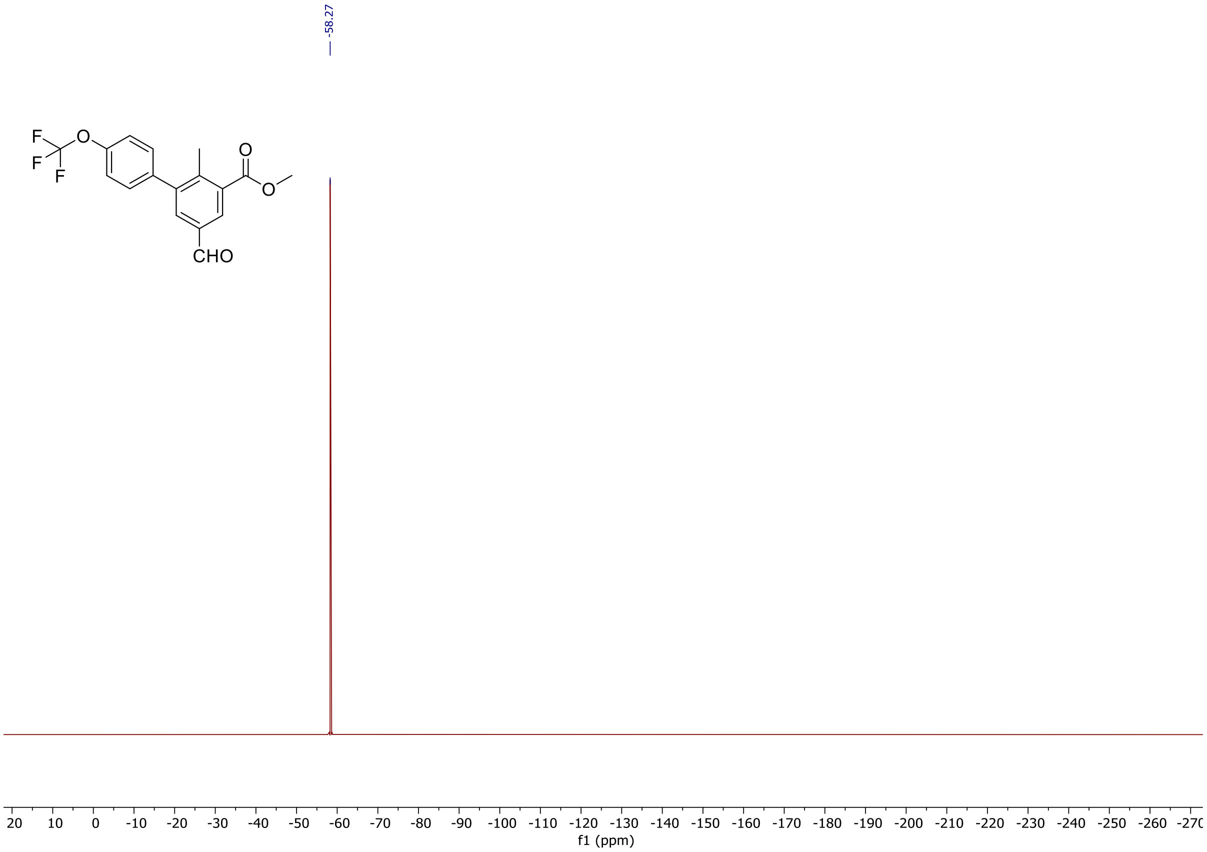

Methyl 5-(4-formyl-2,5-dimethylphenoxy)-2,2-dimethylpentanoate (4q):

^{1}H NMR spectrum (500 MHz) in $CDCl_3$

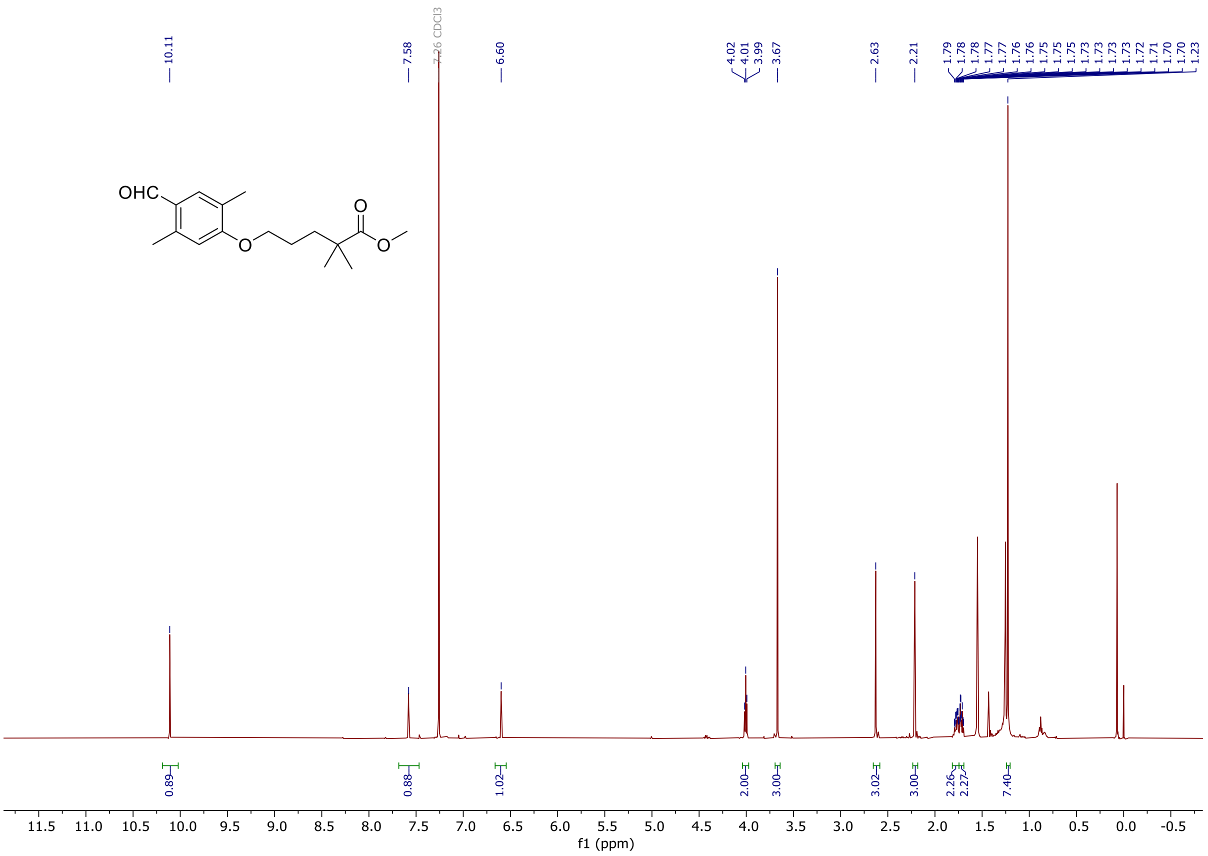

^{13}C NMR spectrum (151 MHz) in $CDCl_3$

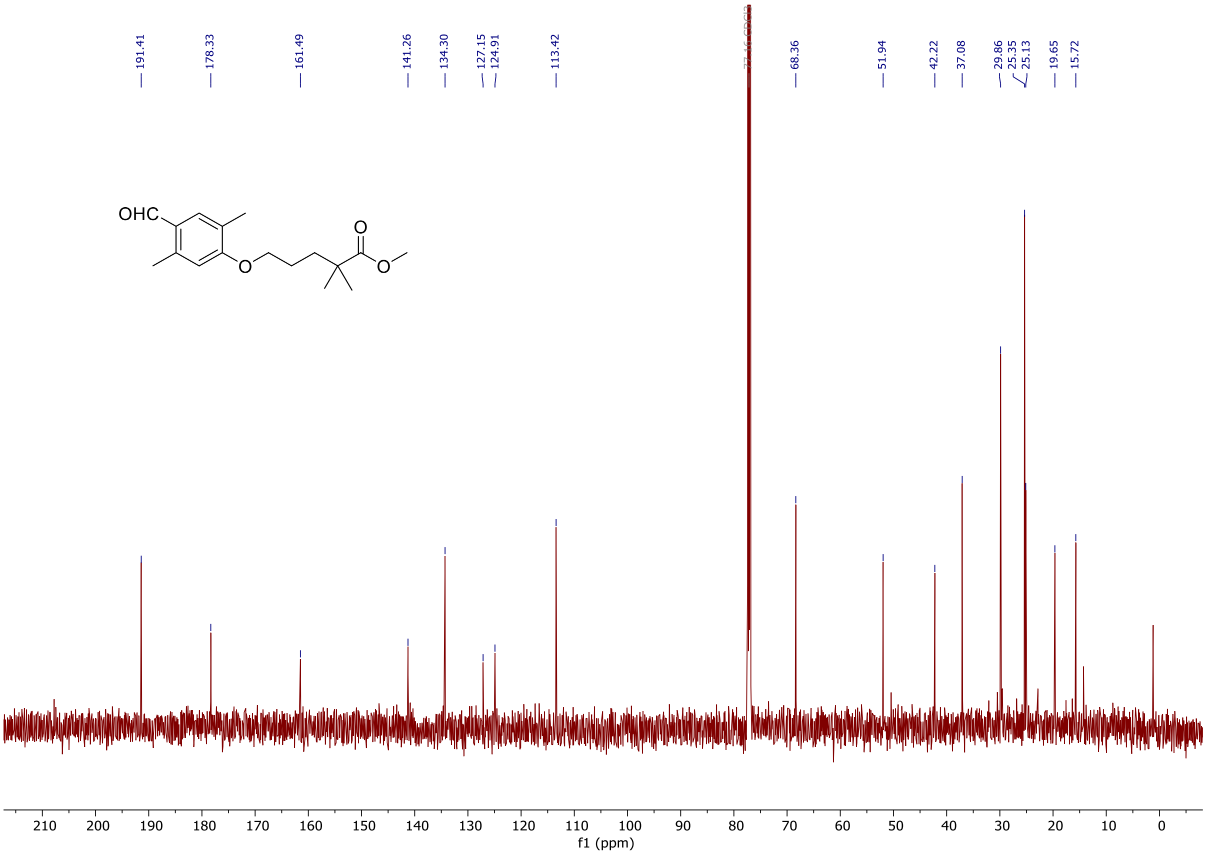

Supplement: Supplementary file 1 [file ol5c03474_si_001.pdf]
